# Chiral Self-Assembly of Zinc and Magnesium Porphyrins with Enantiopure Cyclohexanohemicucurbiturils in Solution and in Solid State

**DOI:** 10.1021/acs.inorgchem.5c04969

**Published:** 2025-11-24

**Authors:** Marko Šakarašvili, Khai-Nghi Truong, Lukáš Ustrnul, Nele Konrad, Kristjan Siilak, Tatsiana Burankova, Reiko Kuroda, Mathias O. Senge, Victor Borovkov, Jas S. Ward, Kari Rissanen, Riina Aav

**Affiliations:** † Department of Chemistry and Biotechnology, 54561 Tallinn University of Technology, Akadeemia tee 15, 12618 Tallinn, Estonia; ‡ Department of Chemistry, 4168 University of Jyväskylä, Survontie 9B, 40014 Jyväskylä, Finland; § Process Analytics, 706742 Hamilton Bonaduz AG, Via Crusch 8, 7402 Bonaduz, Switzerland; ∥ 12740 Chubu University, 1200 Matsumoto-cho, Kasugai-shi, Aichi 487-8501, Japan; ⊥ School of Chemistry, Trinity Biomedical Sciences Institute, 8809 Trinity College Dublin, The University of Dublin, 152-160 Pearse Street, Dublin D02 R590, Ireland; # Institute for Advanced Study (TUM-IAS), Focus Group—Molecular and Interfacial Engineering of Organic Nanosystems, Technical University of Munich, Lichtenberg Str. 2a, 85748 Garching, Germany

## Abstract

Chiral self-assembled supramolecular networks offer great potential for new chiral materials. We report the predictable self-assembly of metalloporphyrins with chiral cyclohexanohemicucurbit­[*n*]­urils (cycHC[6] and cycHC[8]), where cycHC­[*n*] chirality is transferred to achiral porphyrins in solution and the solid state. Solvent and porphyrin substituents directed the formation of 31 novel complexes characterized by single-crystal X-ray diffraction. All complexes feature metal–urea coordination centers, with architectures ranging from discrete complexes to 1D, helical 1D, and 2D square-grid coordination polymers, governed by the shape and binding strength of cycHC­[*n*]­s. Noncovalent interaction analysis highlighted van der Waals interactions alongside metal–oxygen coordination. In solution, binding strengths ranged from 690 to 1,840,000 M^–1^, while electronic circular dichroism (ECD) *g*-factors remained consistent. Solid-state measurements revealed a 10-fold increase in induced ECD intensity in the Soret region, attributed to chiral aggregation and interporphyrin exciton coupling. Vibrational circular dichroism showed chirality induction in the infrared range, with the *g*-factor of cycHC[6] carbonyl signals increasing 10-fold upon complexation. These results demonstrate the robustness of hemicucurbituril–metalloporphyrin supramolecular systems for developing advanced chiral sensing materials.

## Graphical abstract

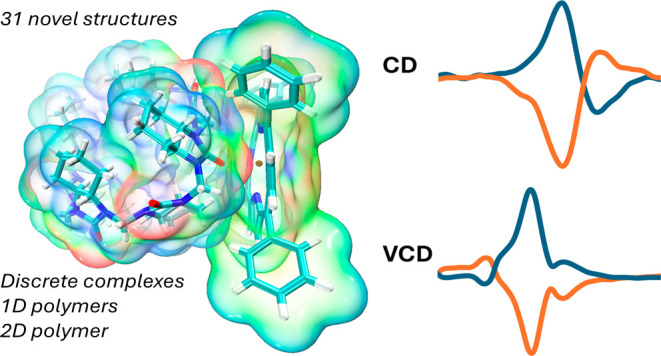

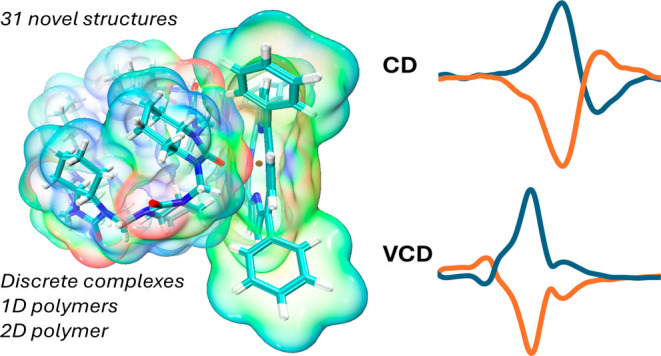

## Introduction

Porphyrins, with their highly tunable ring systems and metal centers,^1,2^ offer remarkable structural and electronic versatility, enabling diverse applications in catalysis,^3,4^ photodynamic therapy,^5,6^ solar energy conversion,^7^ and, notably, chemical sensing.^8–12^ Their conjugated core gives rise to distinctive UV–vis absorption properties, while the chelated metal ion often governs the chemical reactivity. Inducing chirality in porphyrins through supramolecular complexation expands their utility to include the recognition of chiral molecules—such as pharmaceuticals,^13^ environmental pollutants,^14,15^ and food ingredients,^16^ as well as absolute configuration determination.^17–19^ Recently, the induction of chirality in supramolecular host–guest systems has gained significant attention, with studies demonstrating efficient chiroptical responses through the encapsulation of achiral guests such as fullerenes and fluorescent dyes in chiral nanocapsules.^20,21^ Supramolecular control over the self-assembly of metalloporphyrins with a chiral molecular entity enables the efficient formation of tunable chemical and chiroptical sensors. This requires the presence of complementary interaction sites and steric compatibility between the individual molecules. Porphyrins and cyclohexanohemicucurbit­[*n*]­urils^22–24^ (cycHC­[*n*]­s) possess these features (Figure 1). The size of the porphyrin core unit is 0.84 nm, and in the case of 5,10,15,20-tetraphenylporphyrin, the distance between phenyl *meso*-substituents is about 1.5 nm. CycHC[6] and cycHC[8] have comparable dimensions, with a height of roughly 1 nm and diameters of 1.4–1.7 nm.

**Figure 1:**
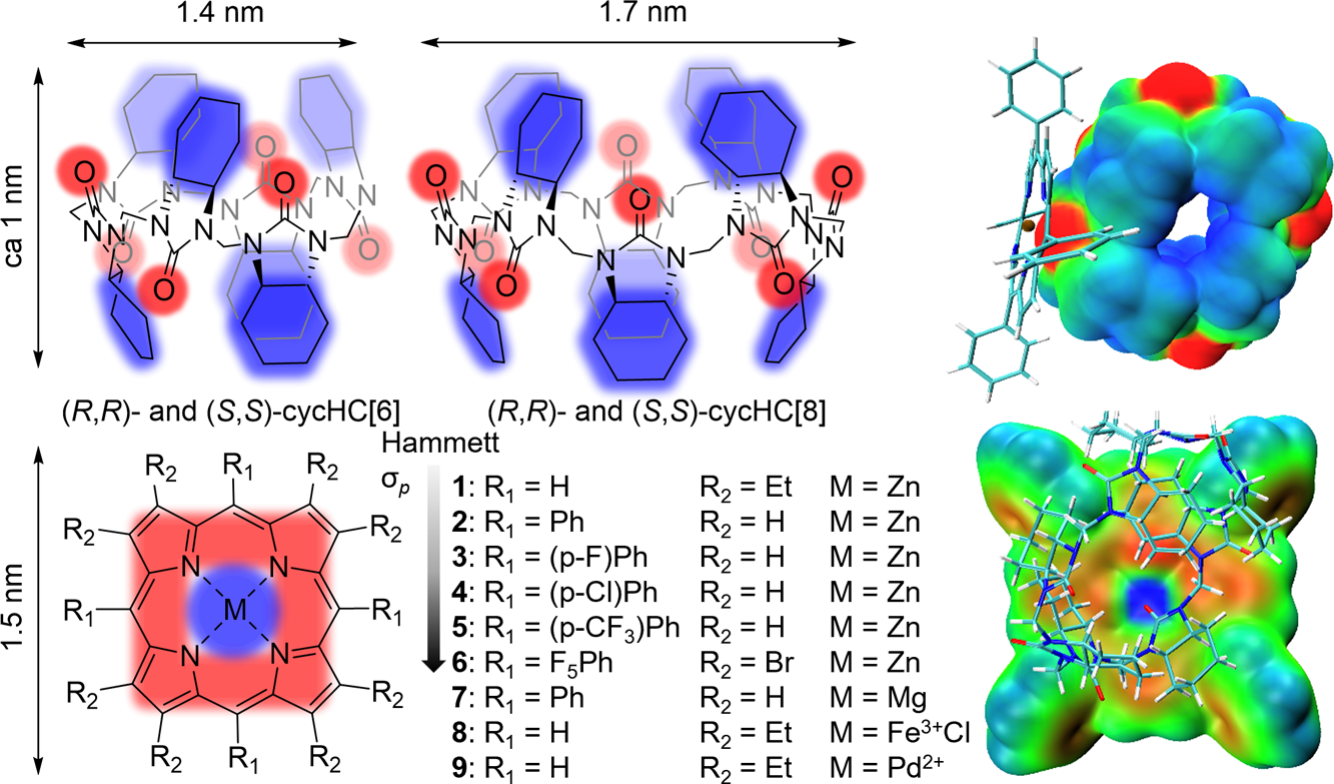
Structures of hemicucurbiturils and metalloporphyrins studied on the left and electrostatic potential colored molecular van der Waals surfaces of (*S*,*S*)-cycHC­[8]·**7** on the right. Blue color corresponds to electron-donating and red color corresponds to electron-accepting capabilities. The Hammett constant σ_*p*_ for *para*-substituents increases from porphyrins **1** to **6**.

Metalloporphyrins and cycHC­[*n*]­s also feature complementary apolar organic regions and Lewis acid–base interaction sites. This can be seen on the electrostatic potential surfaces of the (*S*,*S*)-cycHC­[8]·**7** complex (Figure 1, SI Figures S116 and S117), where the main interaction between the electron-rich carbonyl oxygen (red) and the electron-deficient central metal (blue) is accompanied by a secondary interaction between the cycHC­[*n*]’s hydrophobic parts (blue) and the porphyrin’s π-ring (red). We have reported that the urea groups of enantiomeric cycHC­[*n*]­s can externally bind zinc porphyrins through Lewis acid–base interactions, inducing chirality at both mono- and bis-porphyrin cores.^25,26^ This induces electronic circular dichroism (ECD) signals in the Soret region of inherently achiral (ECD-silent) zinc 2,3,7,8,12,13,17,18-octaethylporphyrin (**1**), zinc 5,10,15,20-tetraphenylporphyrin (**2**), and bis­(zinc 2,3,7,8,12,13,17,18-octaethylporphyrin).^25,26^ Furthermore, the first successful application of cycHC­[*n*] and metalloporphyrin complexes for chiral sensing of volatile organic compounds was demonstrated using an enantioselective electronic nose with a quartz crystal microbalance detector array.^27^

To develop a versatile sensing system and uncover new supramolecular architectures, we investigated how substituents at the Zn-porphyrin ring (**1–6**) and variations in metal cations (Mg^2+^, Fe^3+^, and Pd^2+^; **7–9**) affect their binding with cycHC[6] and cycHC [8], as well as their chiroptical properties. The choice of Mg- and Fe-porphyrins was motivated by their previously reported complexes with oxygen-bearing ligands, while the Pd-porphyrin was recently shown to sense oxygen gas.^12,17,28–33^ However, no information was available regarding their interactions with urea macrocycles. Exploration of new complexes for supramolecular chirogenesis in both solution and solid-state using ECD and vibrational circular dichroism (VCD) spectroscopies is essential for the further development of optical chiral sensing applications. Furthermore, single-crystal X-ray diffraction (SC-XRD) analysis of 31 novel crystal structures revealed the high structural versatility of cycHC­[*n*]–porphyrin complexes. The obtained results demonstrate coherent binding motifs and a broad range of chiral supramolecular architectures, offering a robust platform for next-generation sensing arrays driven by specific cycHC­[*n*]-porphyrin interactions.

### Experimental Section

Unless otherwise stated, all reagents and solvents were purchased from commercial suppliers of the highest grade available and used as received. Compounds prepared in our laboratories were cycHC­[*n*]­s^22,24^ and porphyrins **3**–**7**.^34–37^ All solutions were prepared using Hamilton Gastight syringes; those syringes were also used for all additions during UV–vis and NMR titrations. To ensure precise measurement in sample preparation, the mass of solvent and its density were used along with volumetric glassware. Samples were weighed on a microbalance with an accuracy of 6 μg (Radwag MYA 11.4Y, Poland). The online tool Bindfit (supramolecular.org)^38,39^ was used for evaluating the binding constants. See additionally SI p. S2–S36.

### ^1^H NMR Measurements

All the ^1^H NMR experiments were measured on a Bruker Avance III 400 MHz or a Bruker Avance III 800 MHz spectrometer at a temperature of 298 K. All NMR titrations used deuterium solvent to lock. Chemical shifts in ^1^H NMR were referenced to the deuterated solvent residual peak. The data was analyzed using the program MNova (Mestrelab) and MS excel. See additionally SI p. S7 and S33–S36.

### UV–Vis and CD Measurements

The UV–vis absorption spectra were recorded with a Jasco V-730 spectrophotometer or with a Varian Cary 50 UV–vis spectrophotometer. The CD spectra were recorded with a Jasco J-1500 circular dichroism spectrophotometer in the 370–430 nm range for **1**, the 420–520 nm range for **6**, and the 390–450 nm range for other metalloporphyrins. Measurement parameters were as follows: scanning speed 20 nm/min; data pitch 0.1 nm; digital integration time 4 s; bandwidth 2.00 nm. A total of 6 scans were recorded for each metalloporphyrin cycHC­[*n*] complex, and the average of them was taken; DCM spectra were subtracted for baseline correction. The thermostat (PTC-510) was used to keep the cell holder temperature at 20 °C. See additionally SI p. S37–S51.

Solid-state ECD, LD UV–vis, LB and CB spectra were recorded by a solid-state dedicated circular dichroism (CD) spectrophotometer (J-800 KCM) by mixing the sample with KBr and pressing into a pellet. See additionally SI p. S52–S56.

### IR and VCD Measurements

IR and VCD measurements were carried out with a Bruker Tensor27 spectrometer equipped with a VCD module PMA 50. For VCD measurement, a linear polarizer and a 50 kHz ZnSe photoelastic modulator (PEM) were used. The absorption signals were detected with a liquid nitrogen cooled MCT infrared detector from a sample of a KBr pellet with a resolution of 4 cm^–1^. Data collection time was set at 6 h using six blocks of 60 min. For the demodulation of the signals, a lock-in amplifier (Stanford Research Systems 830) was used. The PEM was adjusted for a maximum efficiency at 1600 or 900 cm^–1^, depending on the region under investigation. A multiple wavelength plate (CdS) combined with a wire grid linear polarizer was used to calibrate the phase of the lock-in amplifier. For the baseline of VCD, a pure KBr pellet was used. The baseline signal was subtracted from the original sample signal. See additionally SI p. S57–S62.

### Single-Crystal X-ray Diffraction

Single-crystal X-ray diffraction data were collected on Rigaku SuperNova and Bruker-Nonius Kappa CCD diffractometers using Cu K_α_ (λ = 1.54184 Å) and Mo K_α_ (λ = 0.71073 Å) radiation, respectively. Data collection and reduction employed CrysAlis^Pro^,^40^ COLLECT,^41^ and HKL DENZO/SCALEPACK,^42^ with absorption corrections applied via Gaussian and SADABS^43^ methods. Structures were solved by direct methods (SHELXS) and refined by full-matrix least-squares on *F* ^2^ using SHELXL-2015.^44–46^ Non-hydrogen atoms were refined anisotropically; hydrogen atoms were positioned geometrically or refined with restraints. Rigid-bond,^47,48^ distance (DFIX), and SIMU restraints were applied as needed. If no sensible disordered model could be formulated for the unknown solvates, the program SQUEEZE^49^ within PLATON^50^ was used to account for the residual electron density. Crystallographic and refinement details, including CCDC numbers, are provided below and in the Supporting Information. See additionally SI p. S63–S103.

### Computational Methods

For all the computations in this work, single-point energy calculations were performed with the ORCA^51–54^ program package using the B3LYP functional and the def2-SVP basis set. The crystallographic coordinates were used as input geometries without further optimization. Tight SCF convergence criteria and ORCA’s Grid5 integration grid were employed.

Multiwfn^55,56^ was used to perform quantitative molecular surface analysis for electrostatic potential surface coloring, noncovalent interactions (NCI, a.k.a reduced density gradient) analysis^57^ and Interaction region indicator (IRI) analysis.^58^ VMD^59^ software was used for visualization of the NCI and IRI color-filled isosurface graphs. The Gnuplot program (http://www.gnuplot.info) was used for generating color mapped NCI and IRI scatter maps. See additionally SI p. S104–S123.

## Results and Discussion

### Variability of Binding Strength of Metalloporphyrins to Hemicucurbiturils

CycHC­[*n*]­s have been shown to form inclusion complexes with anions and electron-rich heterocycles only in protic media, such as methanol and water, whereas Lewis and Bro̷nsted acids form external interactions in nonprotic solvents, such as dichloromethane (DCM).^25,60–62^ In the present work, it was found that nonpolar solvents (toluene, chloroform, and DCM) facilitate the formation of the **2**·cycHC­[*n*] complex with minor differences in affinity, the strongest being observed in DCM (SI Table S1). In contrast, oxygen-containing solvents (e.g., MeOH, THF, and DMSO) suppressed the formation of complexes with cycHC[8] due to competition with abundant solvent molecules (SI p. S3–S8).^63,64^

The influence of substituents on the porphyrin core **1**–**9** was explored by conducting UV–vis titrations in DCM with cycHC­[*n*]­s (*n* = 6, 8), and corresponding affinity constants *K* were determined by evaluating the data with a 2:1 statistical binding model from the online tool BindFit,^38,39^ where *K*_1_ indicates 1:1 and subsequently *K*_2_ 2:1 complex formation equilibria (Table 1 and Table S1, SI p. S8–S36). To highlight the differences in binding strengths of the studied complexes, the *K*_1_ values are compared in Table 1.

**Table 1:** Association Constants of the First Step *K*_1_ for Metalloporphyrin and cycHC­[*n*] Complexes Obtained with Bindfit^38,39^ 2:1 Statistical Model (2:1 Full Model in Case of **7**) Are Listed in the Table (Choice of Binding Models and Acquiring of Constants Is Elaborated in SI)

**metallo-porphyrin**	***K***_**1**_ with **cycHC[6], ×10** ^**3**^ **M** ^**–1**^	***K***_**1**_ with **cycHC[8], ×10** ^**3**^ **M** ^**–1**^
**1**	2.9 ± 0.2	0.69 ± 0.01
**2**	5.34 ± 0.06^a^	2.07 ± 0.02^a^
**3**	4.8 ± 0.3	2.1 ± 0.3
**4**	6.01 ± 0.07	2.87 ± 0.05
**5**	7.4 ± 0.5	3.34 ± 0.02
**6**	409 ± 4	n.d.
**7**	1840 ± 70^b^	1040 ± 40^b^

a Constants from ref 25.

b Apparent *K* in the presence of 10 equiv of water. All constants were determined from at least two independent measurements with enantiomeric cycHC­[*n*]­s, deviations are standard deviation from independently measured *K* values. n.d., not determined (No satisfactory fit could be obtained, SI p. S26–S29).

In agreement with our previous studies on the binding of Lewis basic thioureas,^65^ the binding strength of porphyrins **1**–**6** to cycHC­[*n*]­s correlates with the electron deficiency at the central metal. Porphyrin **1** bearing weak electron-donating ethyl substituents showed the weakest binding, whereas for Zn^2+^ tetraarylporphyrins decorated with chloro-, fluoro-, or trifluoromethyl groups at *para*-position **2**–**5** (Table 1, lines 1–5) the binding strength increases along with the Hammett σ_*p*_ values^66^ of these substituents. A two-order-of-magnitude larger *K*_1_ was obtained for a more extreme example of the electron-deficient Zn-porphyrin **6**, bearing four pentafluorophenyl groups and eight bromine substituents (Table 1, line 6). Strong affinity between cycHC­[*n*]­s and **6** was manifested by full complexation and a 1000-fold lower excess of cycHC­[*n*], compared to titrations of porphyrins **1–5** in the same conditions (SI p. S26–S29).

Variation in the metal cations Mg^2+^ **7**, Fe^3+^ **8**, and Pd^2+^ **9** in the porphyrin coordination center demonstrated that Mg^2+^ is an outstanding alternative to the previously known Zn-porphyrin-cycHC­[*n*] complexes.^25^ No binding to cycHC[8] was observed with **8** and **9** (SI Figures S55 and S56), probably due to an unfavorable interaction between the metal and urea oxygen of cycHC­[*n*]­s. Complexation of **7** with various ligands via nitrogen and oxygen coordination with magnesium cation has been previously described.^33,67–69^ However, the interaction between cycHC­[*n*]­s and Mg-porphyrin **7** uncovered in this study prevailed even in the presence of competing water molecules. It was found that **7** binds cycHC­[*n*] by 2–3 orders of magnitude more strongly than the structurally equivalent zinc porphyrin **2** (Table 1, lines 2 and 7). The binding of **7** with cycHC­[*n*]­s was evaluated as an apparent association constant, as roughly 10 equiv of water per **7** was in the titration solution due to the high oxophilicity of Mg-porphyrin at ambient conditions (SI Figure S60).

Overall, binding constants for porphyrins **1–7** cover the range from 690 to 1,840,000 M^–1^ in DCM and follow the Lewis acidity of porphyrins. The results revealed that the smaller cycHC[6], compared to the larger cycHC[8] macrocycle, has slightly stronger binding for all of the studied Zn^2+^ and Mg^2+^ porphyrins.

### Versatility of Solid-State Supramolecular Architectures

Self-assembly of cycHC­[*n*]­s and metalloporphyrins is affected by the solvent, crystallization temperature, and additives. Crystallization experiments with equimolar mixtures of seven achiral metalloporphyrins (**1–7**) and four chiral cycHC­[*n*]­s produced 31 novel structures: six with (*R,R*)-cycHC­[6], seven with (*S,S*)-cycHC­[6], ten with (*R,R*)-cycHC­[8], and eight with (*S,S*)-cycHC­[8]. All structures display electrostatic M···O interactions, with assemblies ranging from discrete penta- or hexa-coordinated complexes to coordination polymers (Figures 2 and 3, Table S6 and Figures S85–S115).

**Figure 2:**
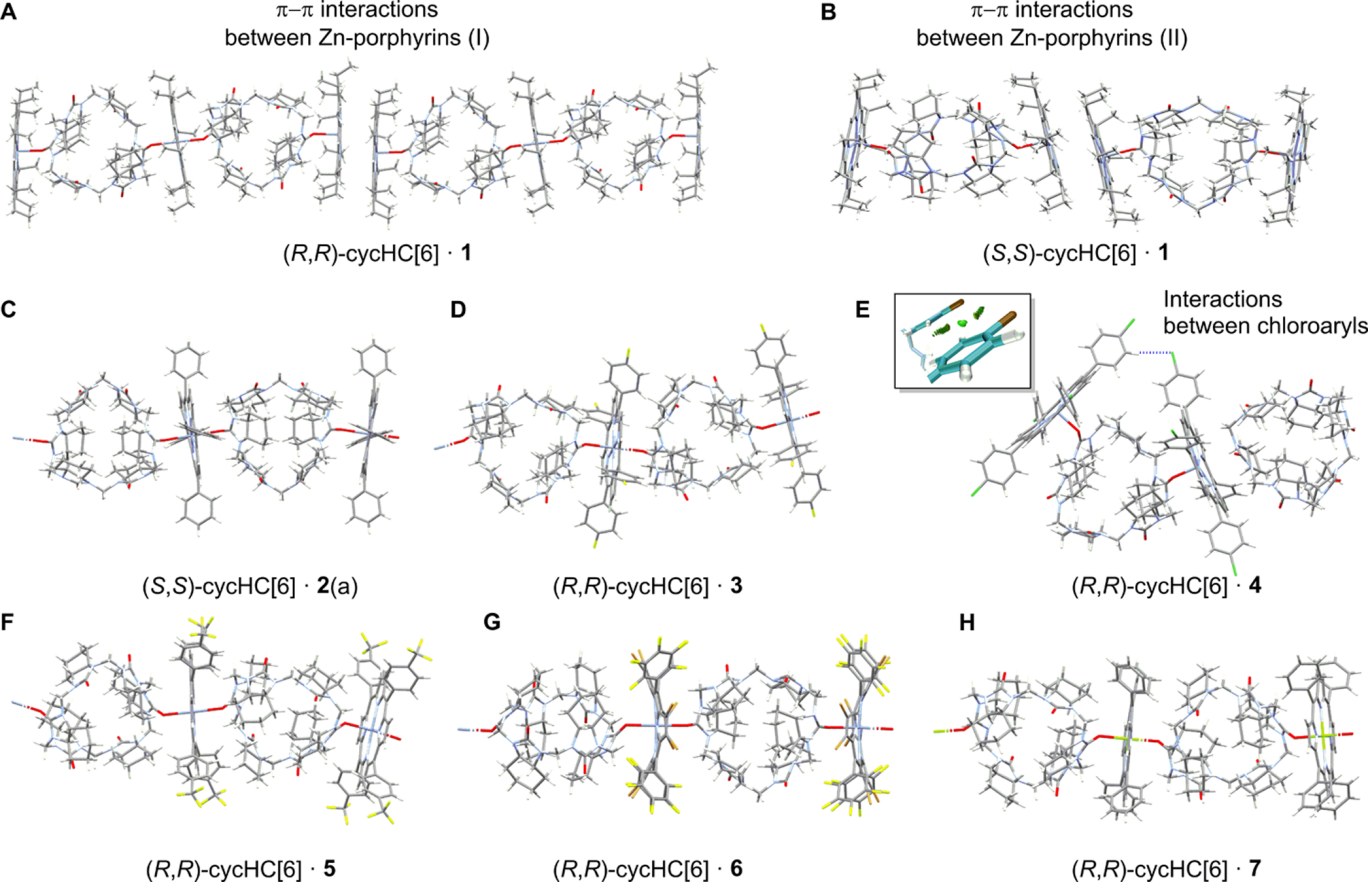
View of the molecular structures of cycHC[6] complexes with porphyrins **1** (A and SI p. S73; B and SI p. S74), **2** (C and SI p. S75), **3** (D and SI p. S77), **4** (E and SI p. S79 and C–H···Cl noncovalent interaction isosurface SI p. S110), **5** (F and Si p. S81), **6** (G and SI p. S83), and **7** (H and SI p. S84). In C, D, F, G, and H, crystals are 1D coordination polymers; see additional details in SI. Atom sites with minor occupancies as well as cocrystallized solvent molecules have been omitted for clarity. Atoms are colored according to the CPK code.

**Figure 3:**
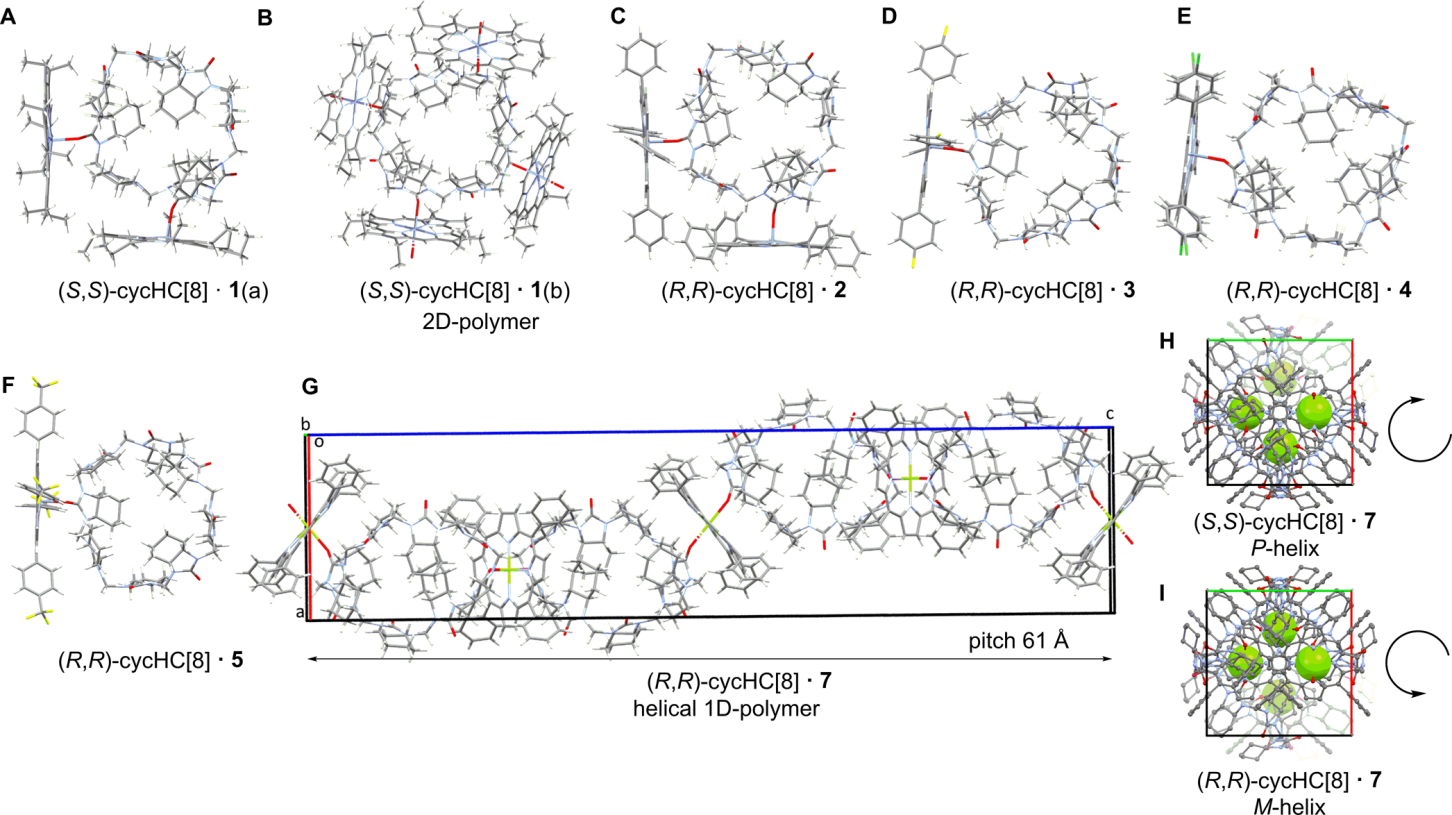
View of the crystal structures in the cycHC[8] complexes with porphyrins **1**(a) (A and SI p. S86), **1**(b) (B and SI p. S87), **2**(a) (C and SI p. S88), and **3**(a) (D and SI p. S94; the enantiomer complex is isostructural SI p. S96, also isostructural to E, SI p. S97), **4**(a) (E and SI p. S97; isostructural to D), **5** (F and SI p. S100), and **7** (G, H, I, and SI p. S102–S103), where B is a 2D square-grid coordination polymer and G is a 1D helical coordination polymer of which H shows a *P*-helix formed with (*S,S*)-cycHC­[8] and I shows an *M*-helix with (*R*,*R*)-cycHC­[8], see additional details in SI p. S102–S103. Atom sites with minor occupancies as well as cocrystallized solvent molecules have been omitted for clarity. Atoms are colored according to the CPK code.

Assembly of (*R*,*R*)-cycHC­[6] with porphyrin **1** at −20 °C in DCM/MeOH yielded a discrete 2:3 complex in which cycHC[6] links two terminal porphyrins in a double-metalloporphyrin sandwich. While the bridging porphyrin coordinates two cycHC[6] molecules via hexa-coordinated Zn···O interactions, terminal Zn centers are penta-coordinated and stabilized by π–π stacking between two porphyrins. This interaction is evidenced by the close proximity (3.3 Å) of planes passing through the porphyrin cores, resulting in a Zn—Zn distance of 4.83 Å (Figure 2A, SI p. S73).

Porphyrin-porphyrin interaction with a distance of 3.4 Å between the porphyrin cores and the Zn—Zn distance of 4.87 Å was also seen in the (*S*,*S*)-cycHC­[6]·**1** polymorph (Figure 2B), the latter was crystallized under the same conditions as its enantiomeric congener; however, in this case a discrete 1:2 complex with solely penta-coordinated Zn centers was formed.

Porphyrin **2** was reported to form a 1D-chain polymer with (*R*,*R*)-cycHC­[6].^25^ In this study, using (*S*,*S*)-cycHC­[6], crystallization at 3 °C also yielded a 1D-chain polymer (Figure 2C, (*S*,*S*)-cycHC­[6]·**2**(a), SI p. S75), that was isomorphous and isostructural to complexes forming with the Mg-porphyrin **7** (Figure 2H, SI p. S84–S85). Crystallization at ambient temperature produces a discrete 1:1 complex with an uncoordinated cycHC[6] molecule ((*S*,*S*)-cycHC­[6]·**2**(b), SI p. S76). This change from a polymer to a discrete complex is accompanied by a 0.2 Å decrease in the Zn···O distance (SI Table S7).

Porphyrin **3** exhibits a strong temperature-dependent behavior: (*R*,*R*)-cycHC­[6]·**3** forms a 1D-chain polymer at ambient temperature (Figure 2D, SI p. S77), whereas crystallization of (*S,S*)-cycHC­[6]·**3** at 3 °C yields a discrete 2:2 complex in which the porphyrins coordinate to two carbonyl oxygens of the one (*S*,*S*)-cycHC­[6] (SI p. S78 while only a single Zn-porphyrin is attached to the second (*S*,*S*)-cycHC­[6]. A similar binding pattern is observed for porphyrin **4**, which bears *p*-chlorophenyl substituents. Intermolecular C–H···Cl interactions (Figure 2 inset shows NCI isosurface, further details SI p. S110) link neighboring porphyrins, resulting in a 1:2 (*R*,*R*)-cycHC­[6]·**4** complex along with an uncoordinated, cocrystallized (*R*,*R*)-cycHC­[6]. These interactions do not perturb Zn···O coordination but bring the porphyrins closer together, preventing formation of a 1D polymer. Despite different crystallization temperatures—ambient for (*R*,*R*)-cycHC­[6]·**4** (Figure 2 E, SI p. S79) and 3 °C for (*S*,*S*)-cycHC­[6]·**4** (SI p. S80)—both enantiomers form isomorphous and isostructural crystals.

Other metalloporphyrins (**5**, **6**, **7**) form 1D-chain polymers with cycHC[6], in which the Zn^2+^ and Mg^2+^ centers are exclusively hexa-coordinated (Figure 2F–H, SI p. S81–S85). For porphyrins **5** and **7**, isomorphous and isostructural complexes are obtained even with varying crystallization temperatures, whereas for porphyrin **6**, only a single enantiomer was crystallized.

Crystallization of the larger macrocycle, cycHC[8], under conditions analogous to those used for cycHC[6], produced a distinct set of single crystals containing porphyrins **1**–**5** and **7**, highlighting the structural adaptability and polymorphism of the host. For porphyrin **1**, two polymorphs were obtained: (*S*,*S*)-cycHC­[8]·**1**(a), crystallized from DCM/MeOH at 3 °C over 8 days, represents a discrete 1:2 complex with two porphyrins bound in a pincer-like fashion to alternating urea subunits of cycHC[8] (Figure 3A and SI p. S86). The Zn···O distances between the porphyrin **1** and the (*S*,*S*)-cycHC­[8] moieties are 0.1 Å shorter than in the (*R*,*R*)-cycHC­[6] crystals (Figure 2A), showing a larger overlap of van der Waals radii due to the lower coordination number at the zinc ion. In the second form, (*S*,*S*)-cycHC­[8]·**1**(b), crystallized from chlorobenzene/MeOH at 3 °C over 2 weeks, chlorobenzene is included in some of the cycHC[8]­s cavities, forming a 2D-square-grid coordination polymer where each (*S*,*S*)-cycHC­[8] binds four porphyrins via alternating urea subunits (Figure 3B, SI p. S87). This structure demonstrates the host’s ability to organize multiple metal centers into an extended 2D network, stabilized by both Zn···O interactions and solvent inclusion. The average Zn···O distance is the same as for a hexa-coordinated Zn^2+^ cation in the 2:3 complex of (*R*,*R*)-cycHC­[6]·**1**.

For porphyrin **2**, the crystallization yielded multiple polymorphs, illustrating the myriad of solid-state structures that could be accommodated by the system under different crystallization conditions. Using (*R*,*R*)-cycHC­[8], the following polymorphs were obtained: (*R*,*R*)-cycHC­[8]·**2**(a) (Figure 3 C and SI p. S88), crystallized from DCM/MeOH at ambient temperature over 2 days, forms a discrete 1:2 complex stabilized by solvate molecules. The other polymorphs—(*R*,*R*)-cycHC­[8]·**2**(c) (SI p. S89), obtained from DCM/MeOH with 1,4-thioxane, (*R*,*R*)-cycHC­[8]·**2**(d) (SI p. S90), from DCM/MeOH with tetrahydrofuran, and (*R*,*R*)-cycHC­[8]·**2**(e) (SI p. S91), from MeOH with (*R*)-(+)-limonene—all produced discrete complexes without incorporation of the additive into the porphyrin–cycHC assembly. For (*S*,*S*)-cycHC­[8], two polymorphs were observed: (*S*,*S*)-cycHC­[8]·**2**(a) (SI p. S92) (DCM/MeOH, 3 °C, 3 days) forms a 1:2 complex analogous to (*R*,*R*)-cycHC­[8]·**2**(a), and (*S*,*S*)-cycHC­[8]·**2**(b) (SI p. S93) (chlorobenzene/MeOH, ambient temperature, 2.5 weeks) includes chlorobenzene in the cavity.

The use of different additives (chlorobenzene, thioxane, tetrahydrofuran, and (*R*)-(+)-limonene) was intended to probe whether ternary complexes could form, i.e., host–porphyrin–additive assemblies. Only in chlorobenzene was a ternary complex formed with inclusion of the aromatic guest to cycHC[8], while maintaining the porphyrin external interaction, demonstrating that cycHC[8] strongly favors direct porphyrin coordination.

Porphyrins **3**–**5** crystallized as discrete 1:1 complexes with both enantiomers of cycHC[8] (Figure 3D–F and SI p. S94–S101). For porphyrin **3**, (*R*,*R*)-cycHC­[8]·**3**(a) and (*S*,*S*)-cycHC­[8]·**3** are isomorphous and isostructural with (*R*,*R*)-cycHC­[8]·**4**(a), forming a family of crystals with essentially identical packing and coordination environments. In contrast, (*R*,*R*)-cycHC­[8]·**3**(b) represents an alternative, nonisomorphous arrangement under slightly different crystallization conditions (with quinine as the additive). Porphyrin **4** also forms two families of crystals: (*R*,*R*)-cycHC­[8]·**4**(b) and (*S*,*S*)-cycHC­[8]·**4** are isomorphous and isostructural with one another, but distinct from the **3**(a)/**3**/**4**(a) set. Porphyrin **5** crystallizes as discrete 1:1 complexes with both (*R*,*R*)-cycHC­[8] crystallized from chlorobenzene/MeOH (chlorobenzene inside) and (*S*,*S*)-cycHC­[8] crystallized from DCM/MeOH, but these do not belong to any previously observed isomorphous families. Across all of these structures, peripheral substituents (fluoro, chloro, or CF_3_) slightly affect packing but do not alter the Zn···O coordination motif, which remains consistent in all cases.

Finally, the only porphyrin forming a 1D-chain polymer with cycHC[8] was **7** (Figure 3G). A preference of **7** to form exclusively polymeric complexes could be expected because Mg^2+^ is typically hexa-coordinated.^70,71^ The nonlinearity of carbonyl groups in cycHC­[*n*]­s leads to a helical polymer with a pitch length of approximately 61 Å including four porphyrins with four cycHC­[*n*]­s. Helix handedness is dictated by host chirality: (*R*,*R*)-cycHC­[8] induces a *P*-helix (Figure 3H), while (*S*,*S*)-cycHC­[8] forms an *M*-helix (Figure 3I).

Across porphyrins **1**–**6**, the Zn···O distances are primarily determined by coordination number: 2.38 Å for hexa-coordinated and 2.18 Å for penta-coordinated cations, in agreement with the literature values, with the only exception being ∼0.1 Å longer distances in cycHC[6]·**1** due to different substituents (ethyl vs phenyl). Mg···O distances in porphyrin **7** are 0.1–0.2 Å shorter than for comparable hexa-coordinated Zn^2+^. A general trend emerges: cycHC[6] preferentially forms 1D polymers when competing hydrogen bonding is absent, whereas cycHC[8] can form extended networks when metal binding is sufficiently strong. This combination of robust host–metal coordination and solvent-sensitive assembly provides a versatile framework for tuning solid-state chirality transfer and material properties, consistent with variations in sensing behavior observed for different porphyrin–cycHC­[*n*] complexes.^27^

### Analysis of NCIs of Complexes

NCI^57,72^ analysis (SI p. S105–S122) and IRI^58^ analysis (SI p. S123) were carried out to gain deeper insight into the interaction modes between cycHC­[*n*]­s and porphyrins. The key difference between approaches is that NCI is primarily used to visualize weak NCIs, whereas IRI allows simultaneous visualization of both covalent and NCI regions.^58,72^ The *x*-axis in the IRI scatter plots (Figure 4C,F) reflects electron density in the interaction regions, which correlates with the strength and nature of the interactions.

**Figure 4:**
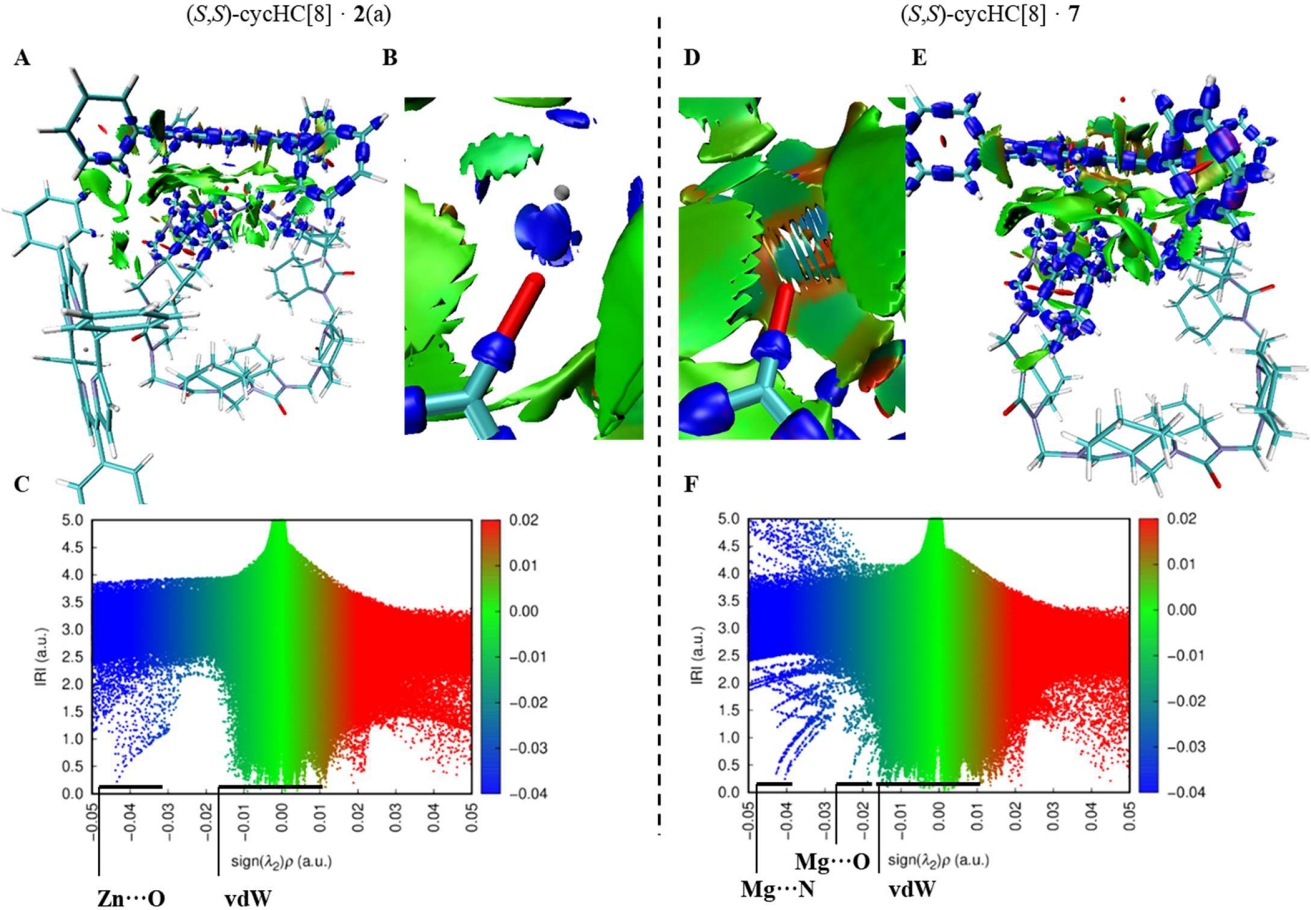
IRI isosurfaces and scatter graphs of porphyrin-cycHC[8] interactions, where grid area is taken between one porphyrin and the porphyrin facing part of cycHC[8], (A) (*S*,*S*)-cycHC­[8]·**2**(a); (B) inset of Zn···O interaction; (C) corresponding scatter graph; (D) inset of Mg···O interaction in (*S*,*S*)-cycHC­[8]·**7**; (E) (*S*,*S*)-cycHC­[8]·**7**; and (F) corresponding scatter graph. Coloring: blue—attractive forces, green—van der Waals interactions, and red—repulsive forces (SI Figure S118).

The IRI isosurfaces and scatter plots for (*S*,*S*)-cycHC­[8]·**2**(a) (Figure 4A–C) and (*S*,*S*)-cycHC­[8]·**7** (Figure 4D–F) reveal clear differences in the distribution of attractive interactions. In (*S*,*S*)-cycHC­[8]·**2**(a), the strongest contributions correspond to Zn···O coordination between the porphyrin metal center and carbonyl groups of cycHC[8] (Figure 4C, Zn···O region), indicating localized and strong metal–oxygen interactions. In contrast, in (*S,S*)-cycHC­[8]·**7**, the corresponding Mg···O interactions are more delocalized (Figure 4F, Mg···O region). This can be attributed to the stronger Lewis acidity^73^ of Zn^2+^ compared to Mg^2+^ and differences in their electron configuration. To further disentangle contributions from different interactions, region masking (by setting RDG to 100 in specific areas) was applied (SI p. S119–S122). This analysis confirmed that one of the dominant high-electron-density regions in (*S*,*S*)-cycHC­[8]·**7** corresponds to Mg···N coordination within the porphyrin core (Figure 4F, Mg···N region).

In addition to metal–oxygen coordination, secondary hydrophobic interactions were detected between the porphyrin and the hydrophobic exterior of cycHC[8] (Figure 4, **2**···cycHC­[8] and **7**···cycHC­[8]). Although weaker than the metal–O coordination, these interactions contribute to the overall stabilization of the supramolecular assembly. Furthermore, Mg^2+^ exhibits additional weak contacts with the carbonyl oxygen atoms of cycHC[8], consistent with previously observed Mg···O interactions in magnesium–carboxylate complexes.^74^

### Chiroptical Properties in Solution and the Solid State

The wide range of association constants determined in solution offers a unique opportunity to investigate the relationship between the binding strength and the chirogenic properties of porphyrins. The chirality induction is evidenced by the emergence of ECD signals in the Soret band region (λ_max_ was in the range of 408–467 nm). The absolute dissymmetry factor—the *g*-factor—was used to describe the intensity of induced circular dichroism (ICD) in order to appropriately compare solution and solid-state chiroptical properties.^75^ The values of the *g*-factors of the studied complexes are shown in Figure 5 with the corresponding ECD spectra (SI Tables S3 and S4). Among the porphyrins studied, the weakest binder, porphyrin **1** with ethyl groups, exhibited only a monosignate ICD in the Soret band region (λ_max_ = 409 nm, Figure 5A). In contrast, the tetraphenylporphyrin derivatives **2**–**5** demonstrated bisignate ICD (λ_max_ = 418 nm, Figure 5C and SI); this is apparently due to exciton coupling and the orientation of *meso*-phenyls.^76–79^ Even though the porphyrins are far apart, the exciton coupling effect has been observed over interchromophoric distances up to 50 Å.^80^ Despite the differences in the ICD shape, the *g*-factors for porphyrins **1**–**5** remained comparable, at around 3.5 × 10^–5^. Notably, the significantly stronger binding magnesium tetraphenylporphyrin **7** displayed a similar ICD shape and position (Figure 5E), with only a slightly larger *g*-factor (4.5 × 10^–5^) compared to the Zn-porphyrins **2**-**5** (Figure 5B and SI Table S3).

**Figure 5:**
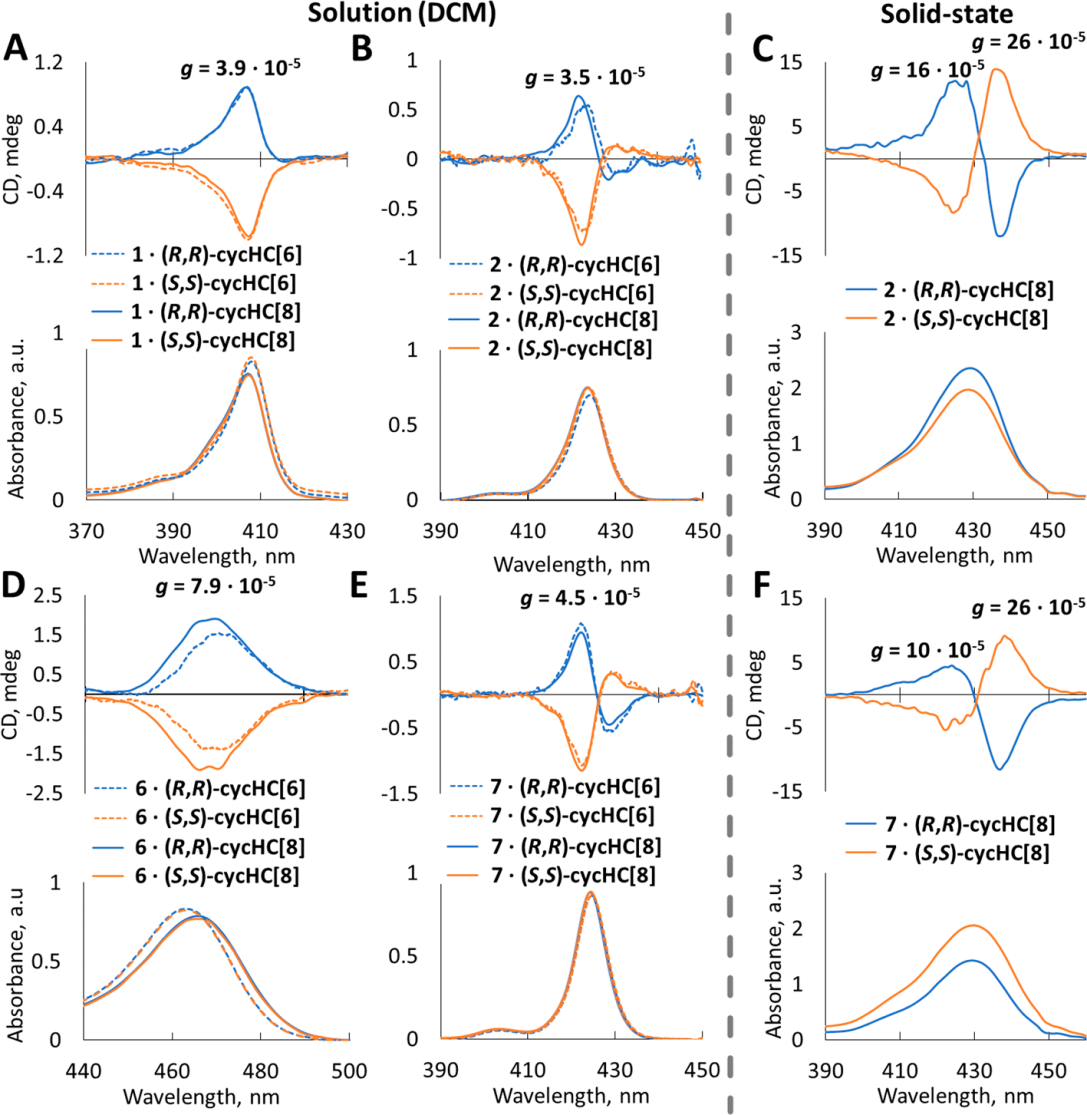
ECD spectra of enantiomeric 1:1 complexes of cycHC[6] (dashed line) and cycHC[8] (solid line) with **1** (A), **2** (B), **6** (D), and **7** (E) in DCM; ECD spectra with other porphyrins and cycHC­[*n*] complexes can be found in SI (p. S37–S51), and the values of λ_max_ and *g*-factors are listed in SI Table S3. ECD spectra of KBr pellets ground with microcrystals of cycHC[8]·**2** (C) and cycHC[8]·**7** (F) and their corresponding absorption spectra. In all the spectra, blue lines correspond to complexes with (*R*,*R*)-cycHC­[*n*], and orange lines correspond to complexes with (*S*,*S*)-cycHC­[*n*].

In contrast to other metalloporphyrins, the geometry of porphyrin **6** is significantly distorted due to β-halogenation, resulting in a red shift of its Soret band.^36,81,82^ It was anticipated that the nonplanar conformation^83,84^ of porphyrin **6** would enhance chiral induction upon complexation, thereby increasing the ICD signal. However, despite the binding strength of complex with **6** being several orders of magnitude greater (Table 1, line 6), its *g*-factor was only about twice that of the other zinc porphyrins **1**–**5** (SI Table S3).

Interestingly, the absorption band of complex **6**·cycHC­[*n*]­s was significantly broader (up to 40 nm, compared to 20 nm for the others) (Figure 5B and SI p. S38–S51). It is known that distortion of the porphyrin core geometry leads to a larger split in the energy gap of electronic transitions, and this agrees with the broader signal of **6**·cycHC­[*n*]­s, compared to other complexes.^78^ Notably, both β-substituted porphyrin **1** and distorted porphyrin **6** exhibited monosignate ICD bands, in contrast to the bisignate signals observed for the planar tetraphenylmetalloporphyrins (**2–5** and **7**). A bisignate pattern can be rationalized by the influence of phenyl group orientations in planar porphyrins,^78,79^ while in the case of monosignate porphyrin **6**, the rotation angle and symmetry of the pentafluoro-groups position in the complex deviate strongly from other phenyl-substituted porphyrins.

In conclusion, the *g*-factor is not significantly affected by the binding strength in solution. The 1:1 complexes of metalloporphyrins **1**–**7** with the corresponding enantiomers of cycHC­[*n*]­s generate specular ECD signals with patterns that reflect substantial changes in porphyrin geometry.

Although the solution measurements of 1:1 complexes indicated weak ICD, it is known that porphyrins may form aggregates with strong ICD, which can be directed by chiral guests.^85,86^ Given the formation of coordination polymers in single crystals, we anticipated differences in solid-state ICD signals compared with those in solution, particularly in the context of the chiral helical coordination polymer of cycHC[8]·**7**. To investigate chirality induction in these complexes in solid-state, we measured the CD of solids using KBr as a pellet matrix in both the UV–vis (ECD) and IR (VCD) regions.^87^ Careful measures were taken to minimize the influence of optical anisotropies, including thorough grinding of the sample using a mortar to achieve a homogeneous pellet. In addition to ECD, linear dichroism as well as linear and circular birefringence were measured, confirming that optical anisotropies remained at negligible levels (SI p. S53–S56). The ECD spectra obtained in the Soret band region for (*R*,*R*)-cycHC­[8]·**2** and (*S*,*S*)-cycHC­[8]·**2** (Figure 5E) showed corresponding mirror images, and the overall shape exhibited a bisignate feature. In contrast to the solution spectra, the UV–vis absorption maxima closely matched the zero point of the ECD spectra, indicating that the major contribution to the chirogenic effect in the solid state is exciton coupling between the neighboring porphyrin chromophores. This phenomenon was previously described in **1** upon the development of chiral aggregates.^86^ However, the Soret band was red-shifted by 5 nm, the intensity of ICD increased, and the relative proportion of the Cotton effect changed in the solid-state in comparison to the solution. Compared to the solution, the *g*-factor increased 7-fold, up to 26 × 10^–5^ in solid-state (Figure 5C). As previously mentioned, porphyrin aggregation can enhance ECD intensity; thus, we anticipated a significant increase in the ECD intensity for cycHC[8]·**7** complexes, driven by their unidirectional helical shape and the corresponding amplification of interporphyrin exciton coupling. Surprisingly, the differences between ECD in solution and solid-state for cycHC[8]·**7** were remarkably similar to those observed for the cycHC[8]·**2** complexes. The Soret band underwent a 4 nm red shift, the signs of Cotton effects remained consistent, and the *g*-factor increased up to 26 × 10^–5^ as in the complex with **2** in solid-state. This increase in ICD intensity in the solid-state compared to the solution can be attributed to chiral aggregation.

Surprisingly, the crystals of the 1:2 discrete complex (cycHC[8]·**2**) and the 1D helical polymer (cycHC[8]·**7**) displayed similar ICD signal intensities, indicating that the organization of porphyrins and cycHC­[*n*]­s within the crystal does not significantly impact ICD. We can only speculate that spatial separation of porphyrins by cycHC­[*n*] macrocycles in the crystal structure allows weak exciton-coupling interactions between the nearest porphyrins. The ECD from powder and crystal of cycHC[8]·**2** were similar in terms of the shape and position of the signals, with the *g*-factors deviating slightly (SI Tables S3 and S4).

While ECD analyzes the molecule as a whole and provides chiral information through chromophore properties, VCD focuses on localized phenomena, offering detailed insights into molecular structures.^88^ Despite limited research on VCD in supramolecular systems, notable examples include chiral porphyrins,^89,90^ porphyrin aggregates,^91,92^ and host–guest complexes involving chiral porphyrins.^19,93^ When it comes to achiral porphyrins complexing with chiral guests, most examples include simple porphyrins and biomolecules such as DNA, oligonucleotides, and peptides—with tripeptides being the smallest example.^94,95^

We were able to explore chirality induction in the IR absorption range and recorded distinct features in VCD spectra of cycHC[6] and cycHC[8] as well as their complexes with **2** and **7** in the KBr matrix (Figure 6 and SI p. S57–S62). The cycHC­[*n*]­s exhibit a relatively strong and characteristic VCD signal in the carbonyl region, and their highest *g*-factors are shown in Figure 6 and SI Table S5.

**Figure 6:**
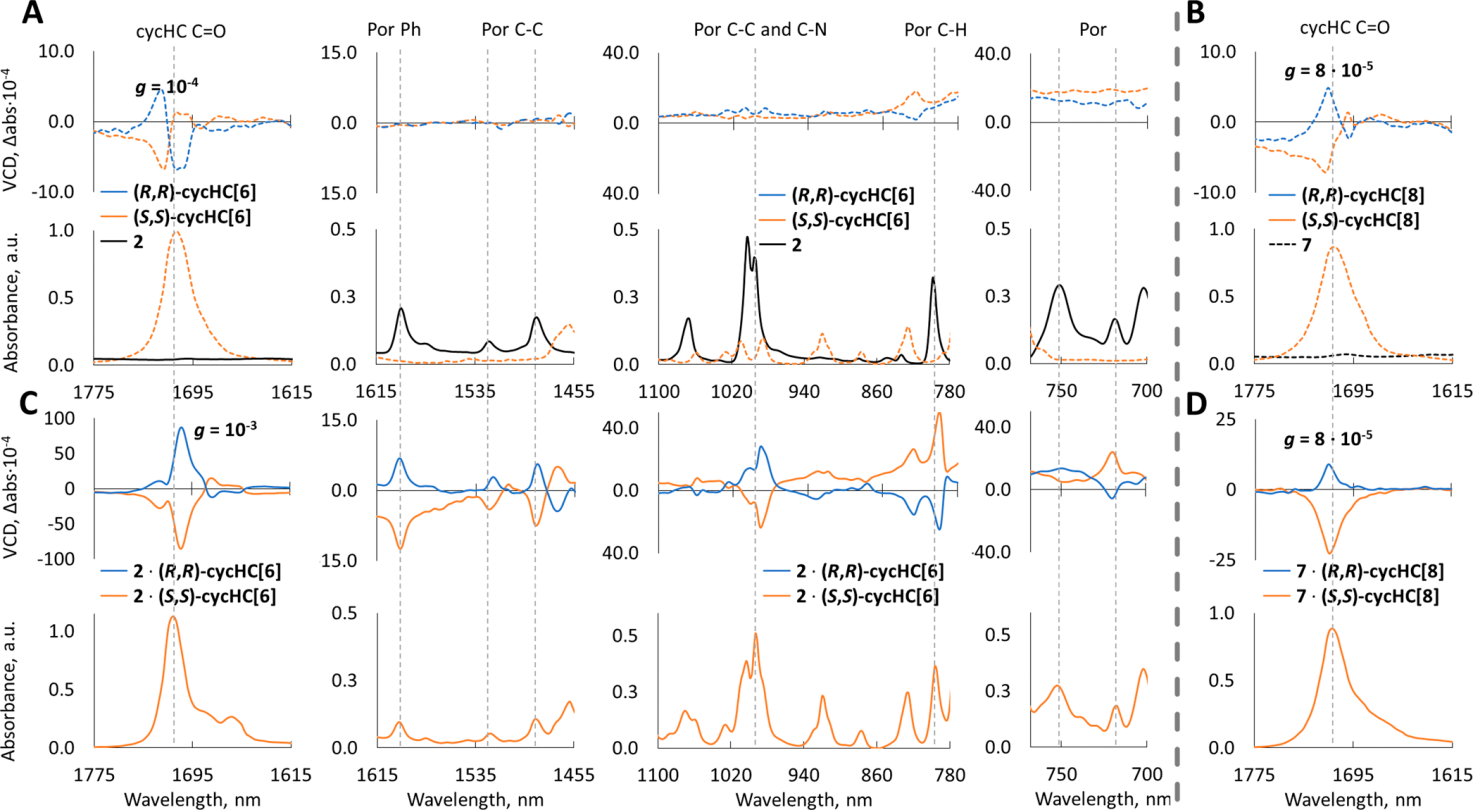
IR and VCD spectra of cycHC­[*n*] complexes measured from KBr pellet: (A) spectral fragments of uncomplexed cycHC[6] and **2**; (B) spectral fragment of uncomplexed cycHC[8] and **7** spectra; (C) spectral fragments of cycHC[6]·**2** enantiomeric complexes; (D) fragment of cycHC[8]·**7** enantiomeric complexes. On all the spectra, blue lines correspond to (*R*,*R*)-cycHC­[*n*] and orange lines to (*S*,*S*)-cycHC­[*n*].

Upon complexation of cycHC[6] with porphyrin **2**, the *g*-factor of the VCD carbonyl signal is increased 10-fold for cycHC[6]·**2**, compared to unbound cycHC[6] (Figure 6A,C). Additionally, several induced VCD signals appeared in the porphyrin **2** vibration regions due to the chiral cycHC[6]·**2** complex formation, the most prominent ones being around 800, 990, 1490, 1530, and 1600 cm^–1^, which are characteristic for pyrrole C–H, C–C, and C–N bonds and phenyl groups in **2**. Moreover, new induced VCD signals at 748 cm^–1^ on Ph and 717 cm^–1^ on C­(beta)H were observed for the cycHC[6]·**2** complex (Figure 6). In cycHC[8]·**2** and cycHC[6]·**7**, the *g*-factors of carbonyl signals were increased 2- to 3-fold compared to unbound cycHC­[*n*]­s. Contrary to cycHC[6]·**2**, the larger homologue cycHC[8] did not induce strong and distinct VCD signals outside of the cycHC[8] carbonyl region (SI p. S57–S62). Interestingly, the only complex that showed a monosignate VCD carbonyl signal and no new apparent shoulder around 1660 cm^–1^ was cycHC[8]·**7** (Figure 6D), which produces a helical 1D-chain polymer in solid-state (Figure 3G–I). CycHC[8]·**7** is also the only complex where the *g*-factor in the carbonyl region did not increase upon complexation (Figure 6C,D). It is known from the literature that the structures of host and guest are rigidified upon complexation, which can lead to a more limited conformational space and more intense VCD signals.^19^ However, induction of the VCD signal in the cycHC­[*n*]–porphyrin complexes cannot be generalized, due to strong induced VCD for cycHC[6]·**2** but only modest changes in signals for other complexes. This phenomenon warrants separate investigation in the future.

## Conclusions

This comprehensive study investigated the complexation of various porphyrins **1**–**9** with enantiomers of cycHC­[*n*]­s, focusing on the influence of the electron-withdrawing groups, metal coordination centers, and solvent media on binding affinity and chirality induction. Our findings reveal that the binding strength of various zinc tetraarylporphyrins **2–5** slightly increases with the growth of Lewis acidity; however, this increase does not significantly affect the induced CD. In contrast, the nonplanar porphyrin **6** and magnesium tetraphenylporphyrin **7** exhibited markedly stronger binding, yet only a modest increase in ICD. Iron and palladium porphyrins showed no binding to cycHC­[*n*]­s. In general, the cycHC[6] exhibited slightly stronger binding than its eight-membered counterpart.

Analysis of SC-XRD data indicated that the Zn**···**O distances for zinc porphyrins **1**–**6** are primarily influenced by the coordination number, ranging from 2.38 Å for hexacoordinated zinc cations and 2.18 Å for penta-coordinated zinc cations. Analysis of NCIs pointed to clear differences between Zn- and Mg-porphyrin binding. A particularly interesting finding is that Zn-porphyrins tend to form 1D chain polymers with cycHC[6], and Mg-porphyrin **7** gave a helical 1D-chain polymer with cycHC[8]. The handedness of the helix was determined by the chirality of cycHC[8]. This structural feature was not observed with porphyrin **2**, prompting further investigation into solid-state chirality, as no discernible differences in ICD were noted between **2** and **7** in solution. In the solid state, we observed a red shift of the Soret band and a significant increase in ICD intensity—up to 10-fold compared to solution—suggesting the formation of aggregates. However, no substantial differences were found between the ICD of complexes **2** and **7**, indicating that the helical structure had no significant influence. VCD studies revealed distinct chiral features, with cycHC[6]·**2** exhibiting the most pronounced VCD spectrum, where the *g*-factor of the carbonyl signal increased 10-fold upon complexation. Specular induced VCD signals were also observed for porphyrins complexed with opposite-handed cycHC­[*n*]­s. In summary, the *g*-factor values were notably larger in the solid state compared to the solution.

A large pool of new supramolecular chiral systems with characteristic spectroscopic and structural features was uncovered, motivating further exploration of their potential in optical sensing or other supramolecular applications.

## Supplementary Material



## Notes

Experimental details and data on titrations, binding constants and characterization of complexes by ^1^H NMR, UV–vis, ECD, VCD, SC-XRD, and computational analysis methods (PDF)
