# Supplementary material for: Chiral Self-Assembly of Zinc and Magnesium Porphyrins with Enantiopure Cyclohexanohemicucurbiturils in Solution and in Solid State
Source: Inorg Chem. 2025 Nov 24;64(48):23773–85. doi: 10.1021/acs.inorgchem.5c04969 (PMC12707794; doi:10.1021/acs.inorgchem.5c04969)
Supplement: Supplementary file 1 [file ic5c04969_si_001.pdf]

Supporting information,

Chiral Self-assembly of Zinc and Magnesium Porphyrins with Enantiopure Cyclohexanohemicucurbiturils in Solution and in Solid-State

*Marko Šakarašvili,[a] Khai-Nghi Truong,[b] Lukáš Ustrnul,[a] Nele Konrad,[a] Kristjan Siilak,[a] Tatsiana Burankova,[c] Reiko Kuroda,[d] Mathias O. Senge,[e,f] Victor Borovkov,[a] Jas S. Ward,[b] Kari Rissanen[b] and Riina Aav *[a]*

a. Department of Chemistry and Biotechnology, Tallinn University of Technology, Akadeemia tee 15, 12618 Tallinn, Estonia. riina.aav@taltech.ee

b. Department of Chemistry, University of Jyväskylä, Survontie 9B, 40014 Jyväskylä, Finland.

c. Process Analytics, Hamilton Bonaduz AG, Via Crusch 8, Bonaduz, 7402, Switzerland.

d. Chubu University, 1200 Matsumoto-cho, Kasugai-shi, Aichi 487-8501, Japan

e. School of Chemistry, Trinity Biomedical Sciences Institute, 152-160 Pearse Street, Trinity College Dublin, The University of Dublin, Dublin D02 R590, Ireland.

f. Institute for Advanced Study (TUM-IAS), Focus Group—Molecular and Interfacial Engineering of Organic Nanosystems, Technical University of Munich, Lichtenberg Str. 2a, 85748 Garching (Germany)

Table of contents

1. General information S2
 1.1 Numbering of porphyrins S2
2. Binding studies in solvent S3
 2.1 Binding in different solvents S3
 2.2 Binding studies of porphyrins 1-9 with cycHC[*n*] in DCM S8
 2.3 ^{1}H NMR titration studies of MgTPP (7) with cycHC[*n*] S33
3. Chiroptical properties (UV-Vis and CD) of complexes in DCM S37
4. Chiroptical properties in solid-state S52
 4.1 ECD spectra S52
 4.2 Vibrational circular dichroism (VCD) S57
5. X-ray Crystallographic Studies S63
 5.1 Discrete complexes and coordination polymers featuring cycHC[6]: S73
 5.2 Discrete complexes and coordination polymers featuring cycHC[8]: S86
6. Computational analysis S104
 6.1 Electrostatic potential colored molecular van der Waals surface S104
 6.2 Noncovalent interactions analysis S105
 6.3 Interaction Region Indicator analysis S123
7. References S124

1. General information

Binding studies in solvent were performed by monitoring UV-Vis absorbance on Varian Cary® 50 UV-Vis spectrophotometer (Agilent Technologies, Inc., Santa Clara, CA, USA) in a 10 mm cuvette. Samples were prepared by weighing solid samples on Radwag (Radom, Poland) MYA 11.4 microbalance and solutions were added by Hamilton Gastight® syringe and the volume was verified by weighing on Precisa (Dietikon, Switzerland) semi-micro balance HF-125SM-FR.

All the ^{1}H NMR experiments were measured on Bruker Avance III 400MHz spectrometer or Bruker Avance III 800MHz spectrometer at temperature 298 K. All NMR titrations used deuterium solvent to lock. Chemical shifts in ^{1}H NMR were referenced to deuterated solvent residual peak. The data was analyzed using the program MNova (Mestrelab) and MS excel.

The UV-Vis absorption spectra were recorded with a Jasco V-730 spectrophotometer or with Varian Cary® 50 UV-Vis spectrophotometer. The CD spectra were recorded with a Jasco J-1500 Circular dichroism spectrophotometer in the 370-430 nm range for **1**, 420-520 nm range for **6**, and 390-450 nm for other porphyrins. Measurement parameters were: scanning speed 20 nm/min; data pitch 0.1 nm; digital integration time 4 sec; bandwidth 2.00 nm. A total of 6 scans were recorded for each porphyrin cycHC[*n*] complex and average of them was taken; DCM spectra was subtracted for baseline correction. Thermostat (PTC-510) was used to keep the cell holder temperature at 20 °C. For evaluating binding the online tool Bindfit (supramolecular.org)[1,2] was used.

Solid-state ECD, LD UV-Vis, LB and CB spectra were recorded by solid-state dedicated circular dichroism (CD) spectrophotometer (J-800KCM) by mixing the sample with KBr and pressing it to a pellet.

IR and VCD measurements were carried out with Bruker spectrometer Tensor27 equipped with VCD module PMA 50. For VCD measurement a linear polarizer and a 50 kHz ZnSe photoelastic modulator (PEM) were used. The absorption signals were detected with liquid nitrogen cooled MCT infrared detector from a sample of KBr pellet with resolution of 4 cm^{-1}. Data collection time was set on 6 hours using six blocks of 60 min. For the demodulation of the signals a lock- in amplifier (Stanford Research Systems 830) was used. The PEM was adjusted for a maximum efficiency at 1600 cm^{-1} or 900 cm^{-1}, depending on the region under investigation. Multiple wavelength plate (CdS) combined with a wire grid linear polarizer was used to calibrate the phase of the lock- in amplifier. For baseline of VCD pure KBr pellet was used. Baseline signal was subtracted from original sample signal.

1.1 Numbering of porphyrins

1 – Zinc$^{(2+)}$ 2,3,7,8,12,13,17,18-octaethylporphyrin – ZnOEP
2 – Zinc$^{(2+)}$ 5,10,15,20-tetraphenylporphyrin – ZnTPP
3 – Zinc$^{(2+)}$ 5,10,15,20-Tetrakis(4-fluorophenyl)-porphyrin – Zn(*p*-F)TPP
4 – Zinc$^{(2+)}$5,10,15,20-Tetrakis(4-chlorophenyl)-porphyrin – Zn(*p*-Cl)TPP
5 – Zinc$^{(2+)}$ 5,10,15,20-Tetrakis(4-(trifluoromethyl)phenyl)-porphyrin – Zn(*p*-CF_3)TPP
6 – Zinc$^{(2+)}$ 2,3,7,8,12,13,17,18-Octabromo-5,10,15,20-tetrakis(pentafluorophenyl)-porphyrin – ZnTPFPOBP
7 – Magnesium$^{(2+)}$ 5,10,15,20-tetraphenylporphyrin – MgTPP
8 – 2,3,7,8,12,13,17,18-Octaethyl-21H,23H-porphine iron$^{(3+)}$ chloride – FeClOEP
9 – Palladium$^{(2+)}$ 2,3,7,8,12,13,17,18-octaethylporphyrin – PdOEP

2. Binding studies in solvent

Table S1 Summary table with metalloporphyrin cycHC[*n*] association constants.

No.	Guest	K_1 with guest·cycHC[6], M^{-1} *R,R / S,S*	K_1 with guest·cycHC[8], M^{-1} *R,R /S,S*	Solvent	Signal
1	**1**	2710 ± 20 / 3050 ± 20	692 ± 5 / 689 ± 9	DCM	Soret
2	**2**	-	1110 ± 40 / -	Toluene	Q-bands
3	**2**	-	1080 ± 40 / -	Chloroform	Q-bands
4	**2**	5340 ± 30 / - (ref [3])	2070 ± 40 / - (ref [3])	DCM	Q-bands
5	**3**	4530 ± 30 / 5120 ± 70	1900 ± 100 / 2530 ± 20	DCM	Soret
6	**4**	5980 ± 70 / 6040 ± 30	2880 ± 50 / 2860 ± 20	DCM	Soret
7	**5**	6630 ± 80 / 7540 ± 50	3340 ± 20 / 3340 ± 10	DCM	Soret
8	**6**	$(3.99 \pm 0.04) \cdot 10^5$ / $(4.17 \pm 0.04) \cdot 10^5$	Not fitable	DCM	Soret
9	**7**	$(1.84 \pm 0.07) \cdot 10^6$	$(1.04 \pm 0.04) \cdot 10^6$	DCM	Soret
10	**8**	-	No binding	DCM	Soret
11	**9**	-	No binding	DCM	Soret

2.1 Binding in different solvents

Inclusion complexes of electron-rich guests with cycHC[8] can be formed in MeOH;[4,5] however, binding of ZnTPP to cycHC[8] was not observed neither in MeOH (**Figure S1**) nor in 1:1 (v/v) MeOH:DCM mixture (**Figure S2**). Titration in DCM showed that MeOH binds to ZnTPP (**2**) with $K = 10.7 \pm 0.5$ M^{-1} (**Figure S3**, **Figure S4**), which is similar to the value reported by Tafeenko *et al.*[4] ($K = 7.32 \pm 0.21$ M^{-1} for binding of **2** to MeOH in $CHCl_3$) and considerably stronger than reported by Gogoll *et al.*[3] (K = 0.002 M^{-1} for binding of **2** to MeOH in $CHCl_3$). No binding was observed in THF (**Figure S5**) and DMSO (**Figure S6**). Binding strength between cycHC[8] and **2** in chloroform and toluene (**Figure S7** - **Figure S10**) was determined for comparison to previously published results measured in DCM.[3]

Porphyrin concentrations were adjusted based on intensity of Q-bands to start titration at absorbance lower than 1. In case of binding the acquired data was fitted with 2:1 statistical binding model in all cases except for porphyrin binding to methanol, where a 1:1 model was used.

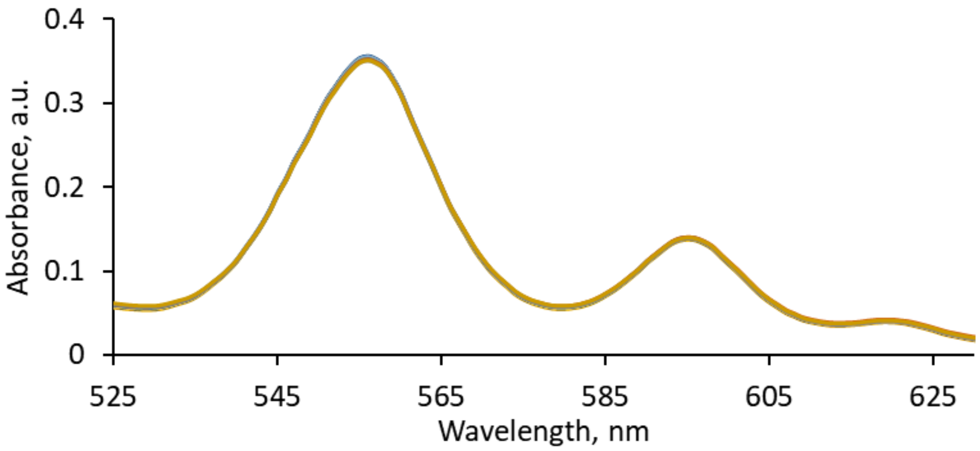

Figure S1 UV-Vis titration spectra of 17.2 × 10^{-6} M **2** solution by **(*R,R*)-cycHC[8]** (up to 128 eq.) in MeOH.

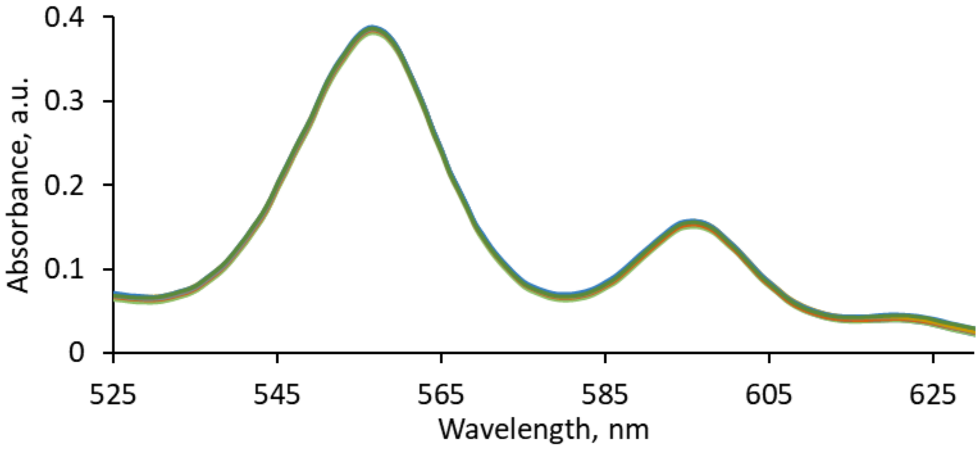

Figure S2 UV-Vis titration spectra of 17.5 × 10^{-6} M **2** solution by **(*R,R*)-cycHC[8]** (up to 162 eq.) in 1:1 MeOH/DCM (v/v).).

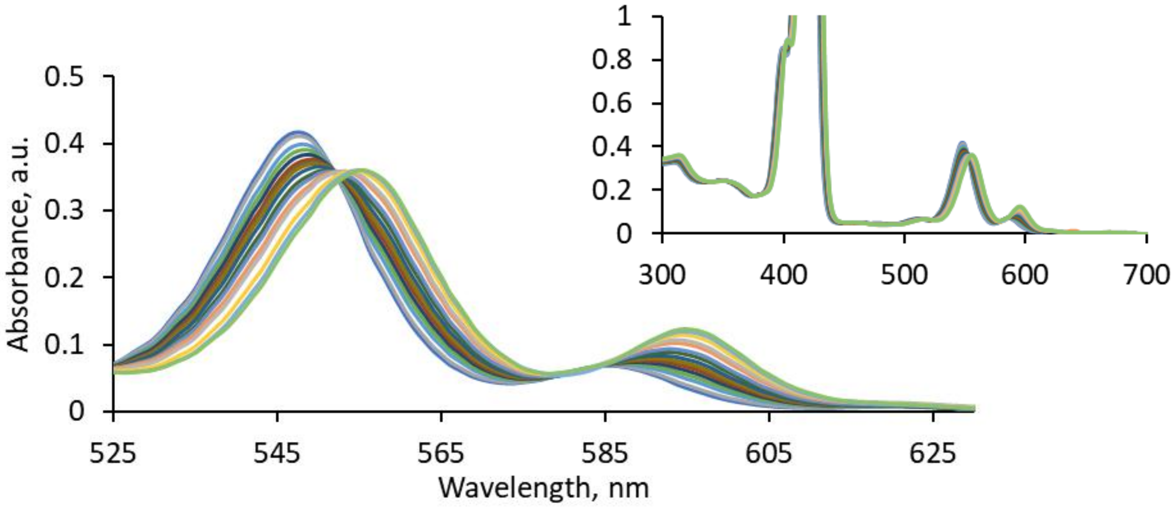

Figure S3 UV-VIS titration spectra of 17.3 × 10^{-6} M **2** solution by MeOH (up to 25702 eq.) in DCM. Full spectra is shown in to upper right corner, as the concentration was chosen for Q-band region the Soret band region is too intense and thus and the following spectra, only Q-Ban region is shown.

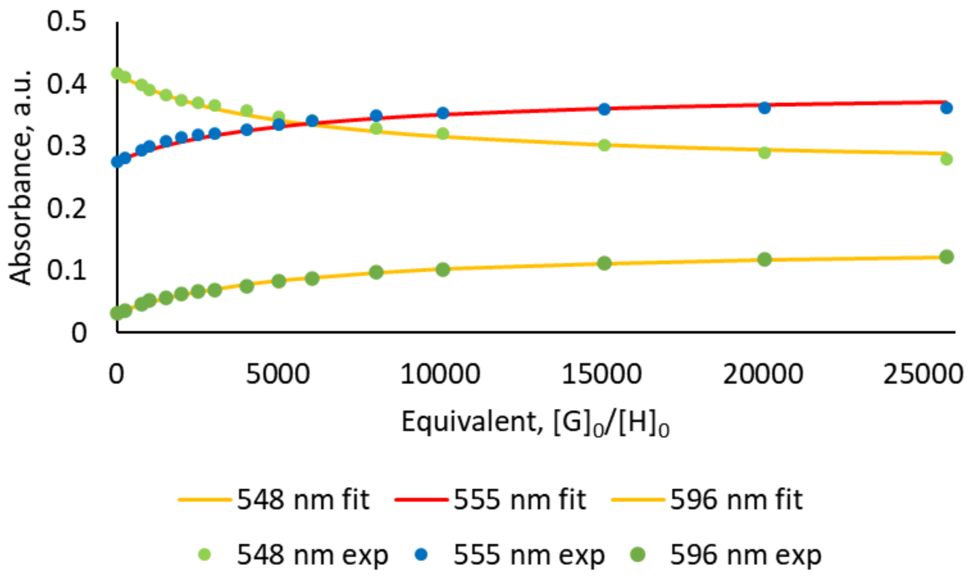

Figure S4 Evaluation of the association constants with 1:1 binding model for 17.3 × 10^{-6} M **2** titrated by MeOH (up to 25702 eq.) in DCM gave K = 10.7 ± 0.5 M^{-1} (*http://app.supramolecular.org/bindfit/view/d5f368e4-fd81-4fff-9f7c-406279c3234f.*).

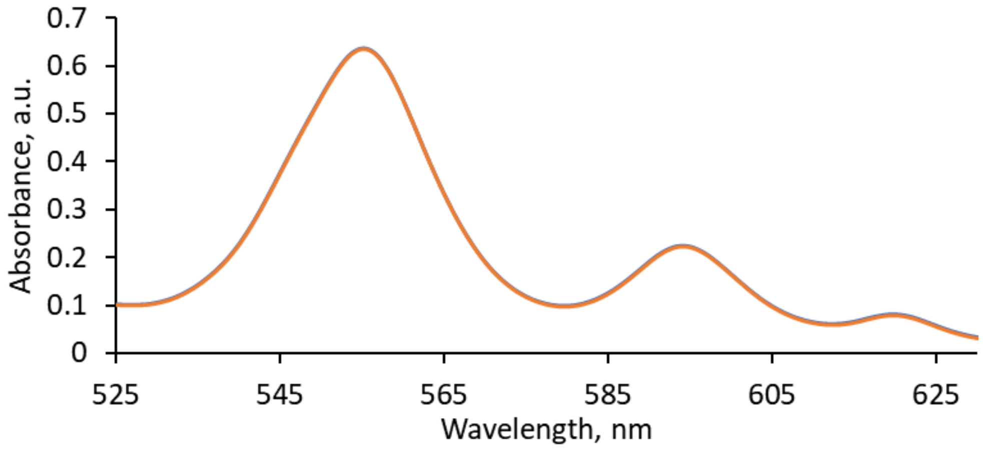

Figure S5 Single addition of 250 eq. **(*R*,*R*)-cycHC[8]** to 25.0 × 10^{-6} M **2** solution in THF; no change observed.

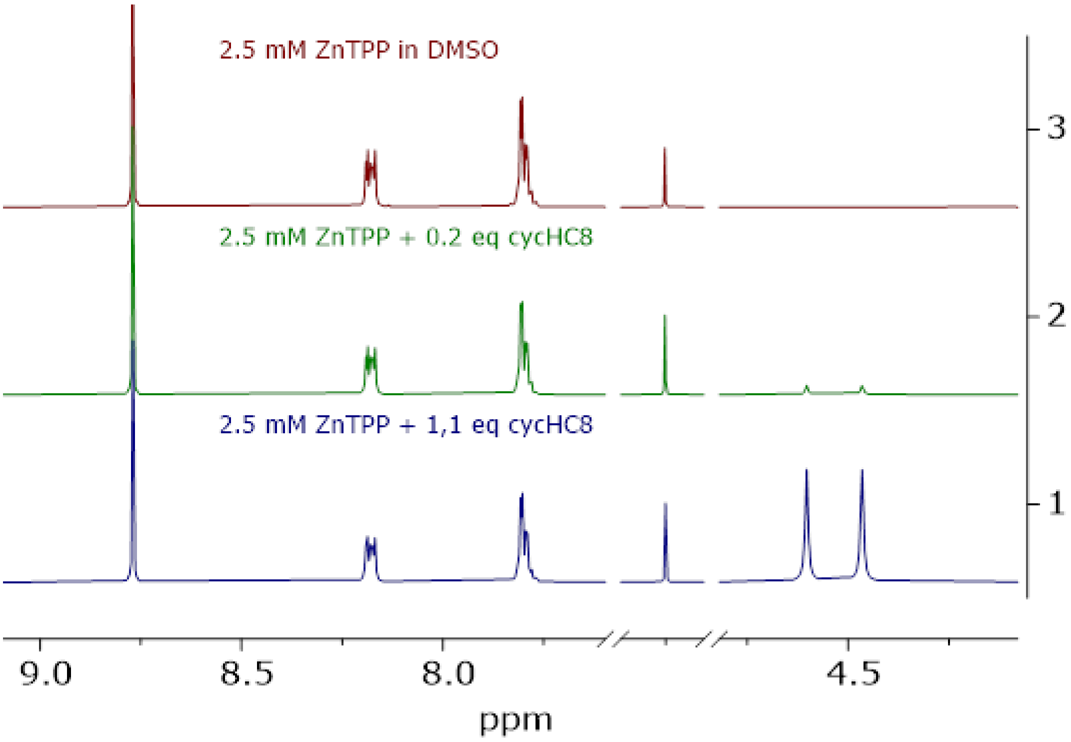

Figure S6 ^{1}H NMR of 2.5 × 10^{-3} M **2** in DMSO with addition of 0.2 equivalent and 1.1 equivalent of **(*R,R*)-cycHC[8]**; no change observed.

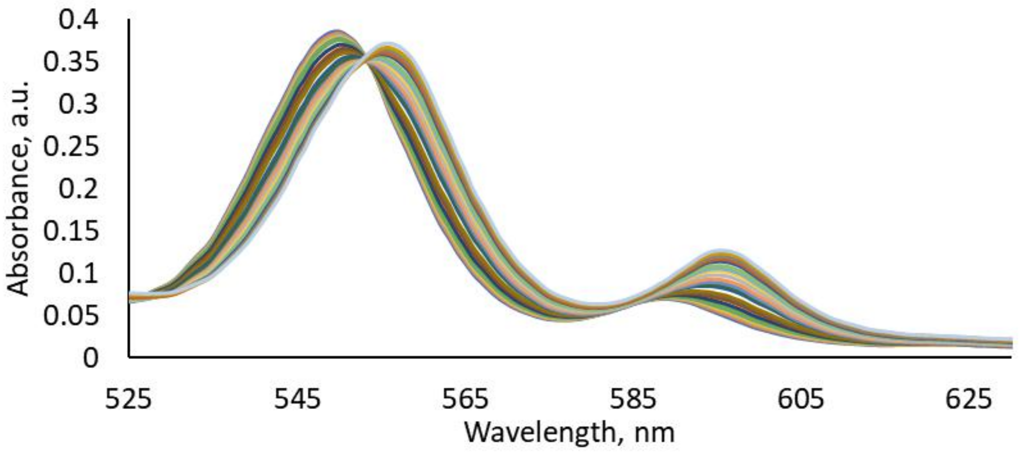

Figure S7 UV-Vis titration spectra of 17.0 × 10^{-6} M **2** solution titrated by **(*R*,*R*)-cycHC[8]** (up to 177 eq.) in toluene.

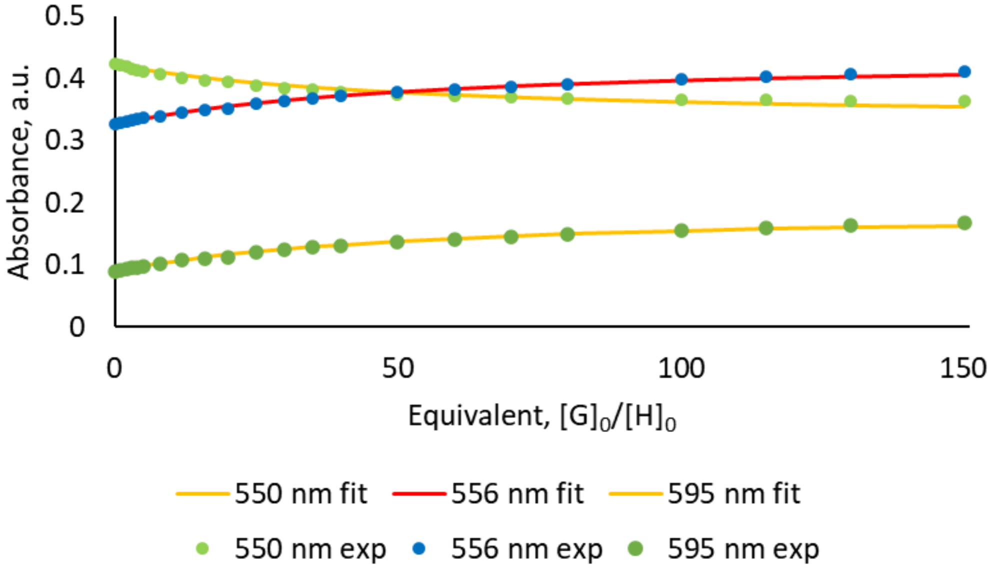

Figure S8 Evaluation of the association constants for 17.0 × 10^{-6} M **2** solution titrated by **(*R*,*R*)-cycHC[8]** (up to 177 eq.) in toluene, K_1 = 1110 ± 40 M^{-1} (*http://app.supramolecular.org/bindfit/view/b1b15955-e6c5-4e47-b12e-5fd9ad1d20a1*).

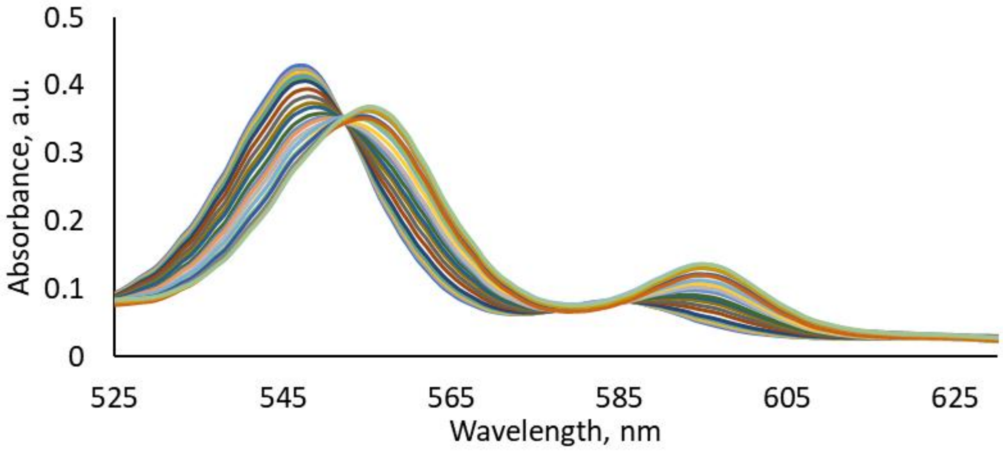

Figure S9 UV-VIS titration spectra of 17.8 × 10^{-6} M **2** solution titrated by **(*R,R*)-cycHC[8]** (up to 257 eq.) in chloroform.

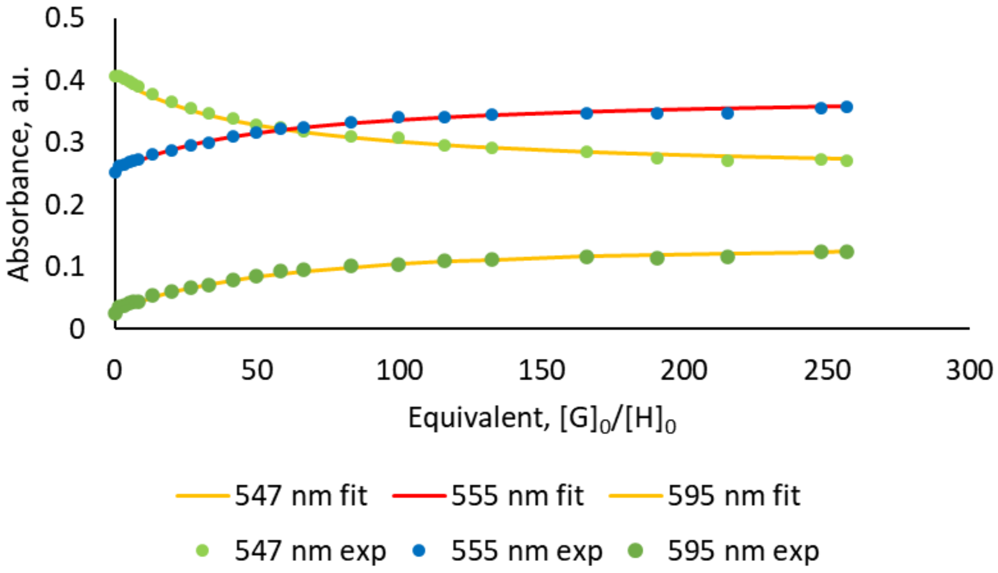

Figure S10 Evaluation of the association constants for 17.8 × 10^{-6} M **2** solution titrated by **(*R,R*)-cycHC[8]** (up to 257 eq.) in chloroform, K_1 = 1080 ± 40 M^{-1} (*http://app.supramolecular.org/bindfit/view/7ba86d34-9984-4468-8578-8090ee61a4db*).

2.2 Binding studies of porphyrins 1-9 with cycHC[*n*] in DCM

Porphyrin concentrations were adjusted to start at absorbance being close to 1 and aliquots of cycHC[*n*] dissolved in the same porphyrin solution were added step-wise. Titrations with cycHC enantiomers were considered as parallels because both enantiomers must have the same affinity to an achiral porphyrin.

The evaluation of affinity constants with 3:1 binding model demanded large amount of experimental data to provide good fits.[3] Assessment of association constants for **1**·(*R,R*)-cycHC[8], **2**·(*R,R*)-cycHC[6] and **2**·(*R,R*)-cycHC[8], with 2:1 statistical binding model from the online tool Bindfit[1,2] (www.supramolecular.org) provides similar K_1 values (Table S2) while assuming K_2 = K_1/4.

K_1 from the latter binding model was used for all host-guest systems (except **7** where 2:1 full model was used) studied in this work to provide representative comparison and good estimate of the strength of the first binding event.

Table S2 Table comparing published K_1 using 3:1 model and Bindfit 2:1 statistical model from same experimental data.[3]

System	K_1 using 3:1 model from ref [3]	K_1 by Bindfit 2:1 statistical	
**1**·(*R*,*R*)-cycHC[8]	880 M^{-1}	880 M^{-1}	Bindfit1
**1**·(*R*,*R*)-cycHC[6]	3070 M^{-1}	2980 M^{-1}	Bindfit2
**2**·(*R*,*R*)-cycHC[6]	5340 M^{-1}	5330 M^{-1}	Bindfit3
**2**·(*R*,*R*)-cycHC[8]	2070 M^{-1}	2160 M^{-1}	Bindfit4

Binding of ZnOEP (1) with cycHC[*n*]

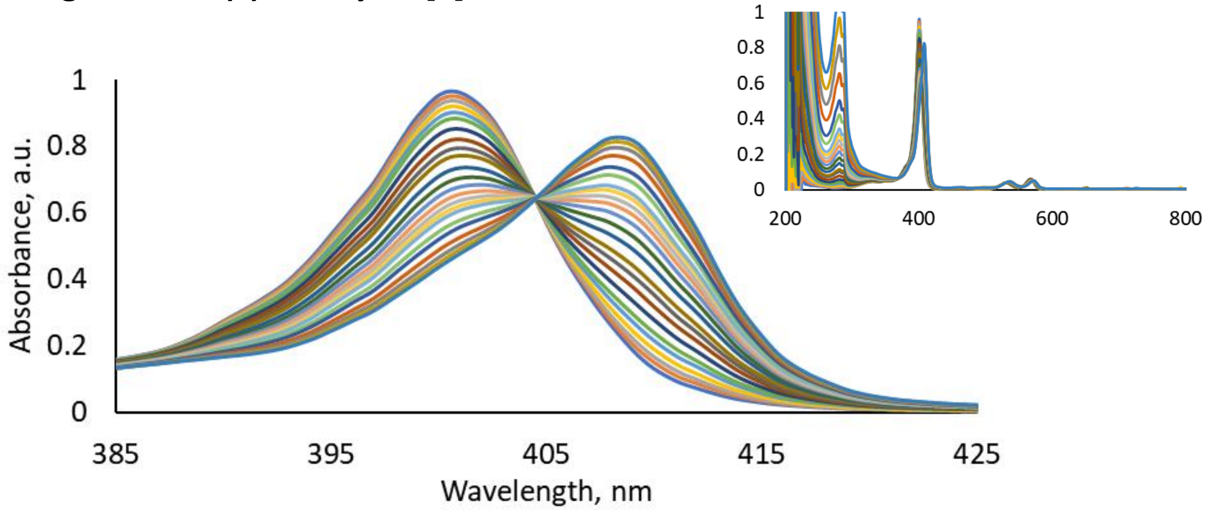

Figure S11 Titration of 2.49 × 10^{-6} M **1** by **(*R,R*)-cycHC[8]** (up to 3493 eq.) in DCM. In the upper right corner, full spectra is shown, as the concentration was chosen for Soret band region, the Q-bands have too small intensity to reliably analyze and are thus omitted from following spectra. The signal in the UV region is from cycHC's carbonyl n → π* transition.

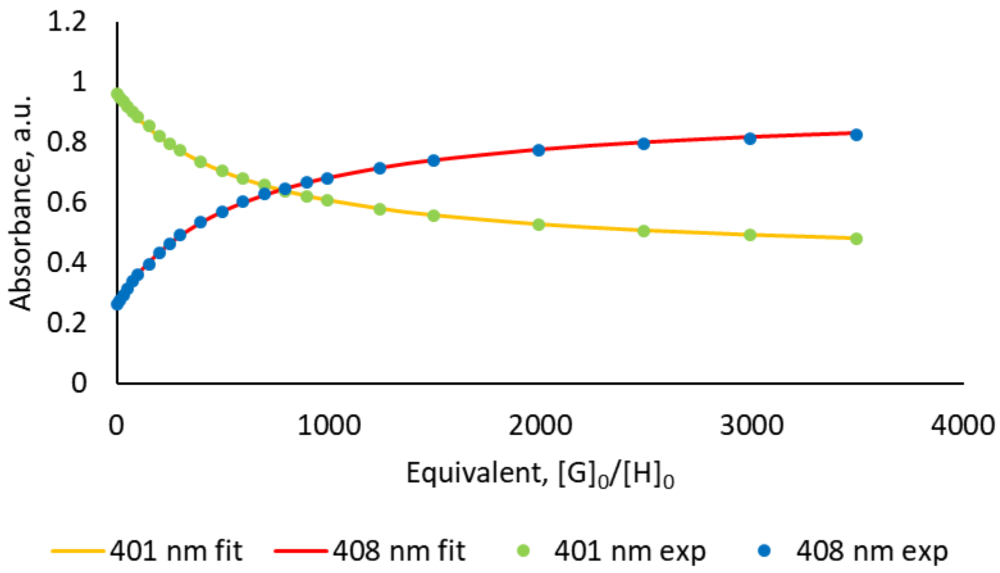

Figure S12 Evaluation of the association constants for 2.49 × 10^{-6} M **1** titrated by **(*R,R*)-cycHC[8]** (up to 3493 eq.) in DCM, K_1 = 692 ± 5 M^{-1} (*http://app.supramolecular.org/bindfit/view/27a930bd-10e4-41bf-b7b1-5f4fcb0d5101*).

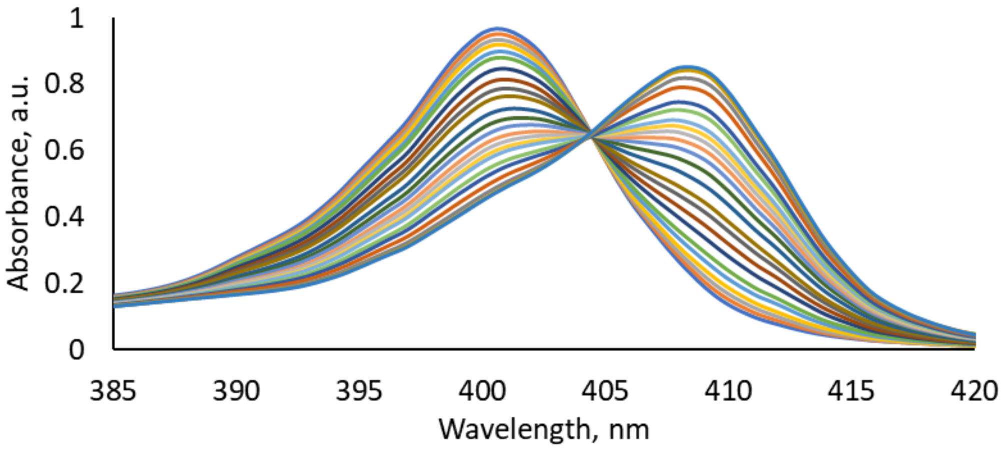

Figure S13 Titration of 2.49 × 10^{-6} M **1** by **(*S,S*)-cycHC[8]** (up to 3201 eq.) in DCM.

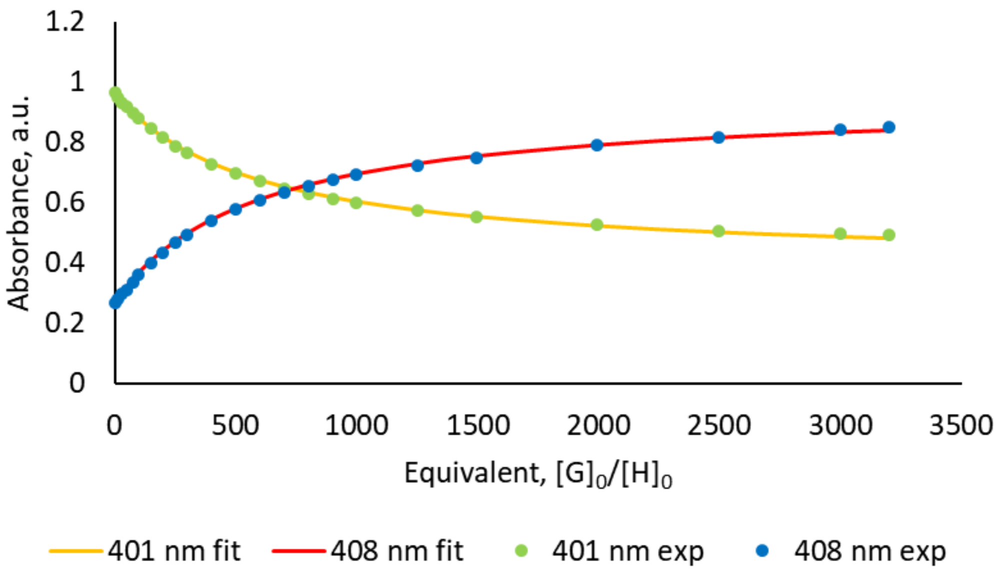

Figure S14 Evaluation of the association constants for 2.49 × 10^{-6} M **1** titrated by **(*S,S*)-cycHC[8]** (up to 3201 eq.) in DCM, K_1 = 689 ± 9 M^{-1} (*http://app.supramolecular.org/bindfit/view/f719607e-6799-4250-a219-97c5ea67eb91*).

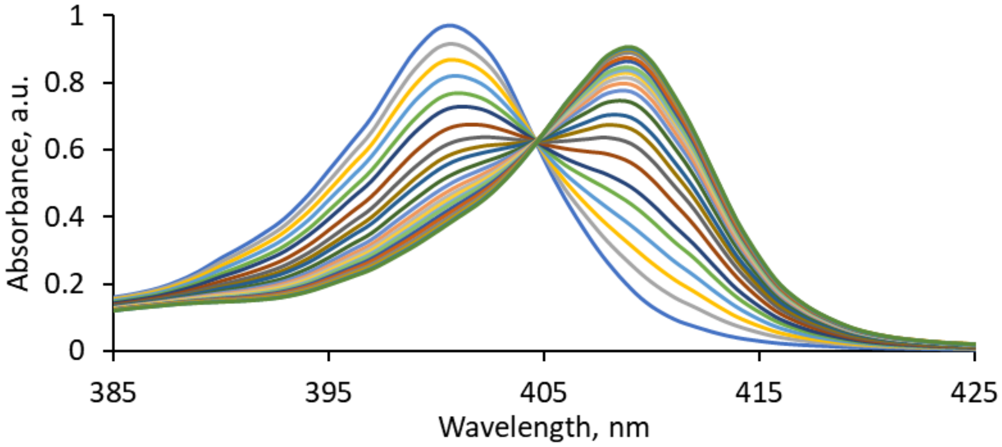

Figure S15 Titration of 2.49 × 10^{-6} M **1** by **(*R,R*)-cycHC[6]** (up to 3386 eq.) in DCM.

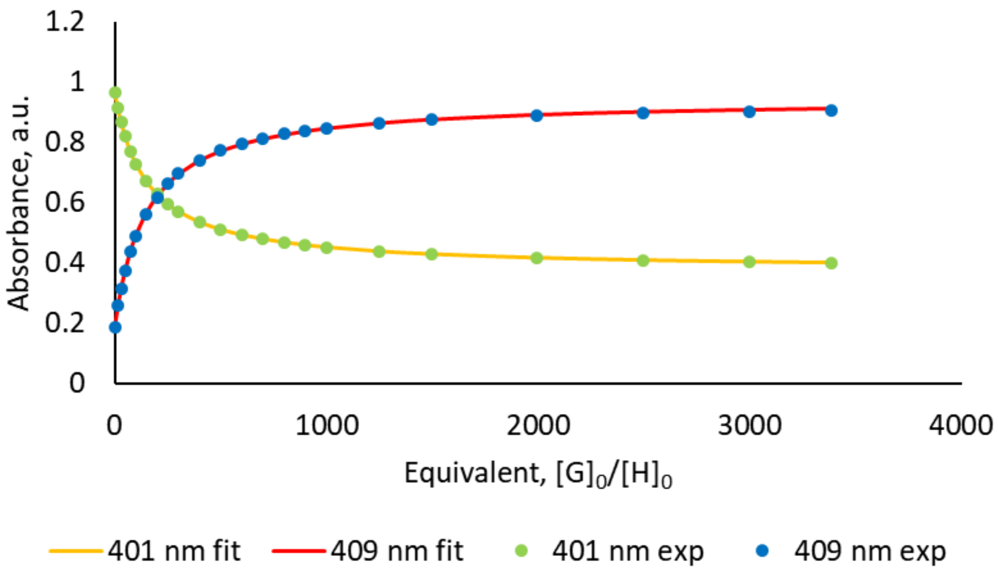

Figure S16 Evaluation of the association constants for 2.49 × 10^{-6} M **1** titrated by **(*R,R*)-cycHC[6]** (up to 3386 eq.) in DCM, K_1 = 2710 ± 20 M^{-1} (*http://app.supramolecular.org/bindfit/view/170d9349-aa39-4bc4-905a-e841c07f2772*).

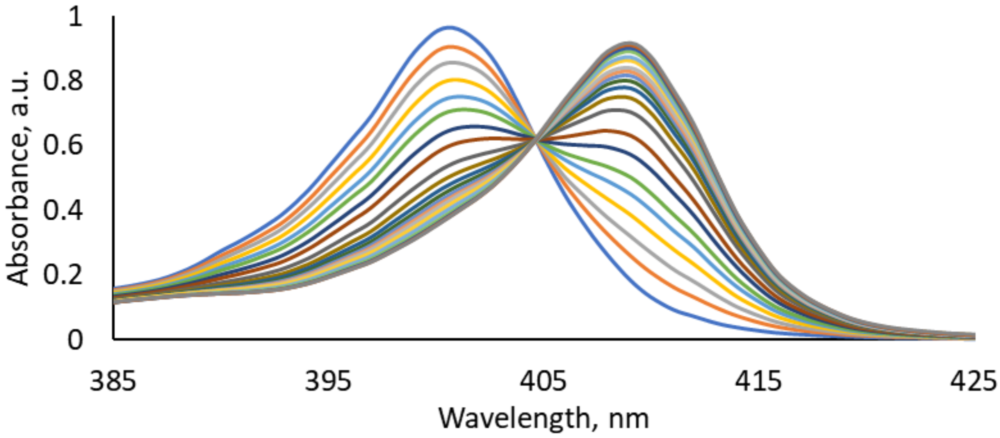

Figure S17 Titration of 2.49 × 10^{-6} M **1** by **(*S,S*)-cycHC[6]** (up to 3217 eq.) in DCM.

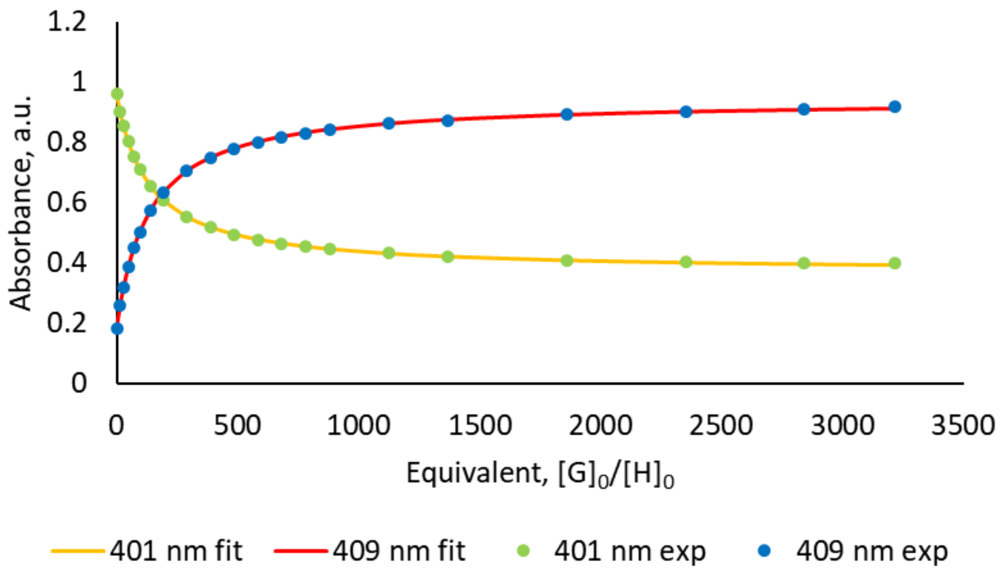

Figure S18 Evaluation of the association constants for 2.49 × 10^{-6} M titrated **1** by **(*S,S*)-cycHC[6]** (up to 3217 eq.) in DCM, K_1 = 3050 ± 20 M^{-1} (*http://app.supramolecular.org/bindfit/view/65ca2606-7e1c-492c-b73f-92e1b796b760*).

Binding of Zn(*p*-F)TPP (3) with cycHC[*n*]

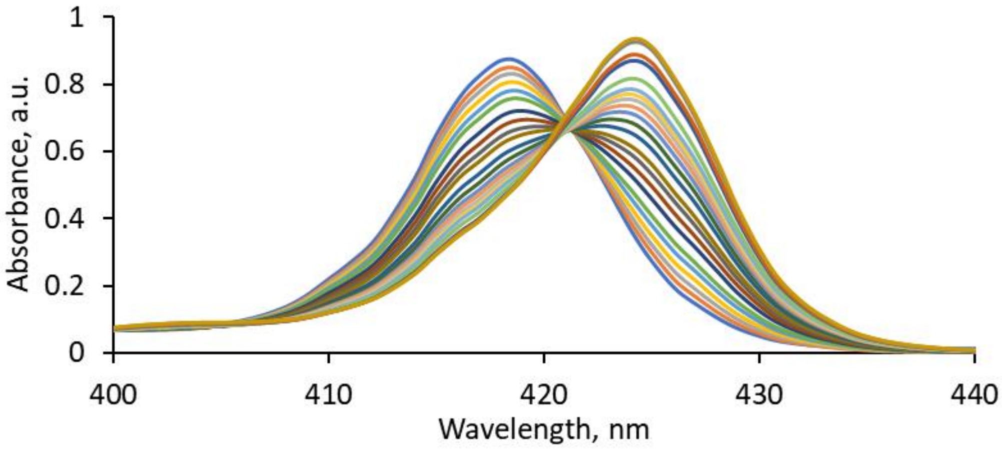

Figure S19 Titration of 1.40 × 10^{-6} M **3** by **(*R,R*)-cycHC[8]** (up to 2639 eq.) in DCM.

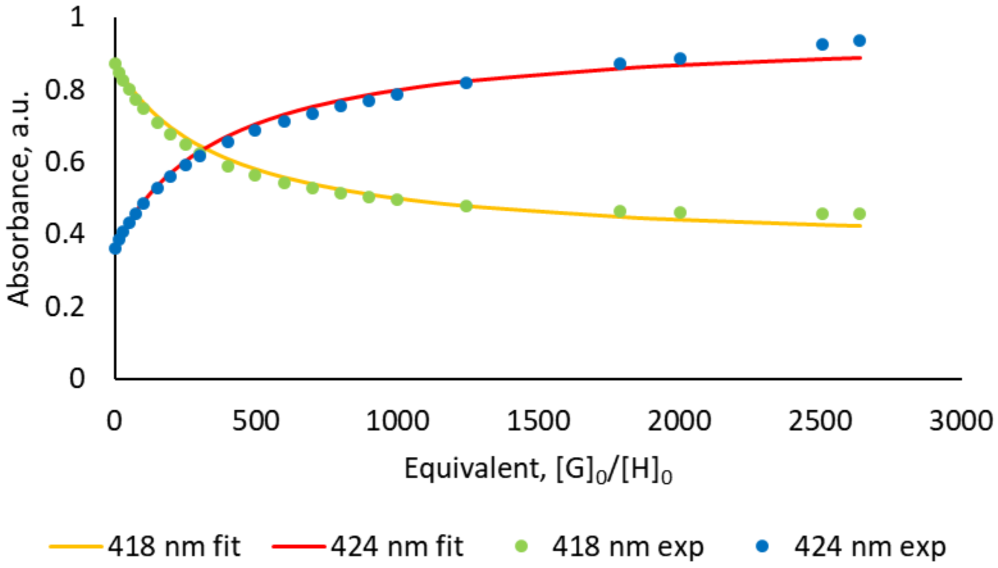

Figure S20 Evaluation of the association constants for 1.40 × 10^{-6} M **3** titrated by **(*R,R*)-cycHC[8]** (up to 2639 eq.) in DCM, K_1 = 1900 ± 100 M^{-1} and parallel experiment with K_1 = 2160 ± 10 M^{-1} (*http://app.supramolecular.org/bindfit/view/3a288f88-e2ac-426e-b7e0-fff3f0315bea* and *http://app.supramolecular.org/bindfit/view/796f3c24-c0ec-4b82-ad78-00cea503c2ad*).

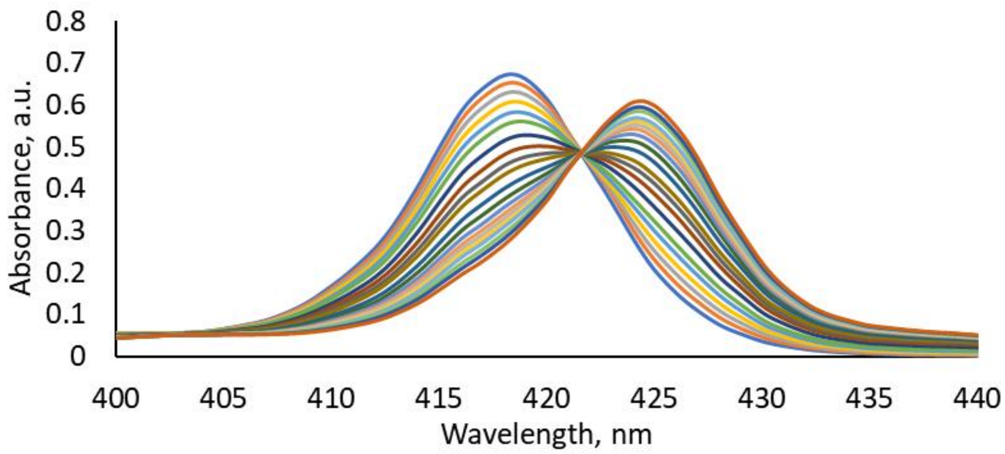

Figure S21 Titration of 1.36 × 10^{-6} M **3** by **(*S*,*S*)-cycHC[8]** (up to 2001 eq.) in DCM.

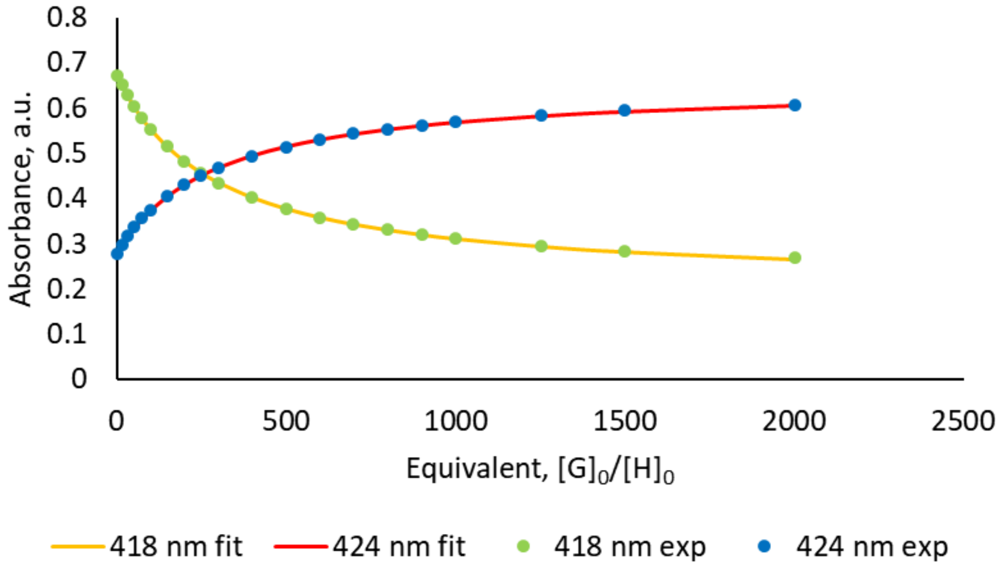

Figure S22 Evaluation of the association constants for 1.36 × 10^{-6} M **3** titrated by **(*S*,*S*)-cycHC[8]** (up to 2001 eq.) in DCM, K_1 = 2530 ± 20 M^{-1} and parallel experiment with K_1 = 1700 ± 20 M^{-1} (*http://app.supramolecular.org/bindfit/view/531cca5d-4898-4c39-93f2-0210d5d2a13f* and *http://app.supramolecular.org/bindfit/view/335964da-4b37-4383-9351-5f5f8acbac6f*).

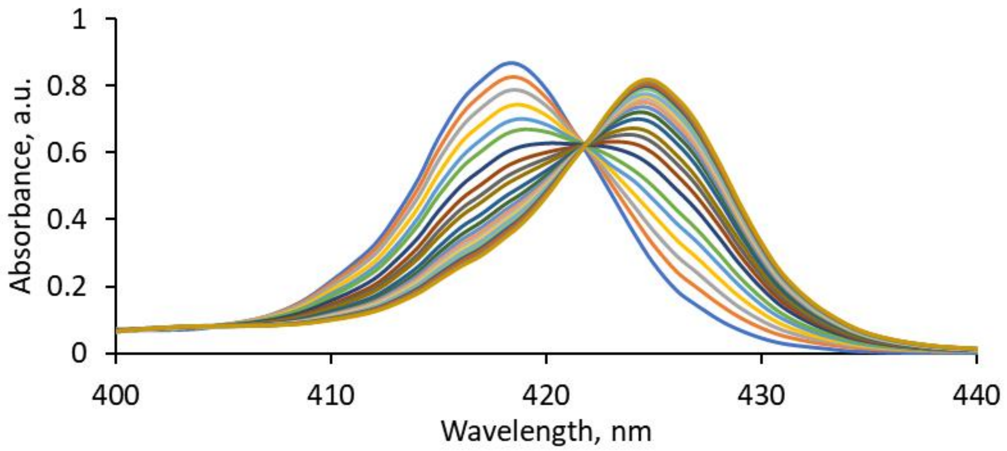

Figure S23 Titration of 1.40 × 10^{-6} M **3** by **(*R,R*)-cycHC[6]** (up to 2763 eq.) in DCM.

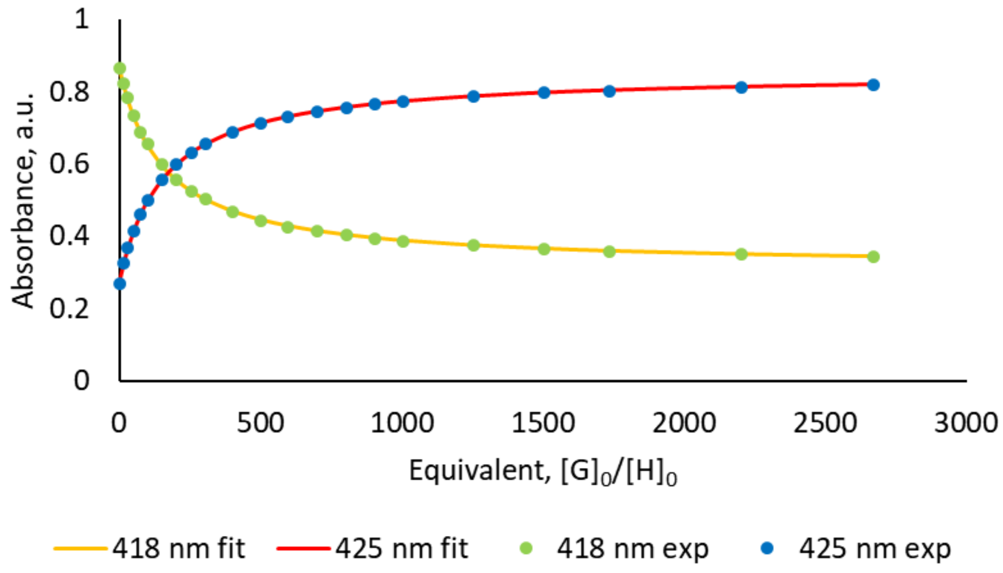

Figure S24 Evaluation of the association constants for 1.40 × 10^{-6} M **3** titrated by **(*R,R*)-cycHC[6]** (up to 2763 eq.) in DCM, K_1 = 4530 ± 30 M^{-1} and parallel experiment with K_1 = 4710 ± 40 M^{-1}(*http://app.supramolecular.org/bindfit/view/62047219-1314-409f-a213-285ded50ae9c* and *http://app.supramolecular.org/bindfit/view/618130d0-de21-4db9-abed-282f1c169438*).

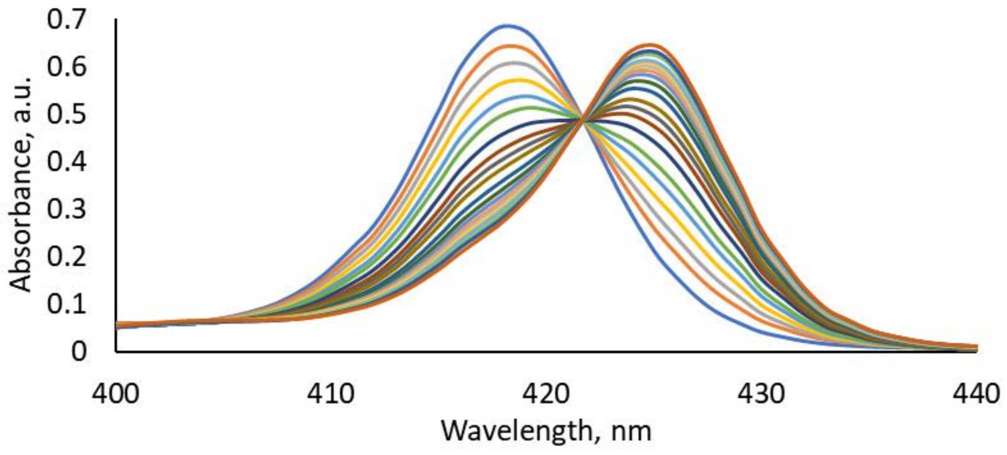

Figure S25 Titration of 1.36 × 10^{-6} M **3** by (***S,S***)**-cycHC[6]** (up to 2002 eq.) in DCM.

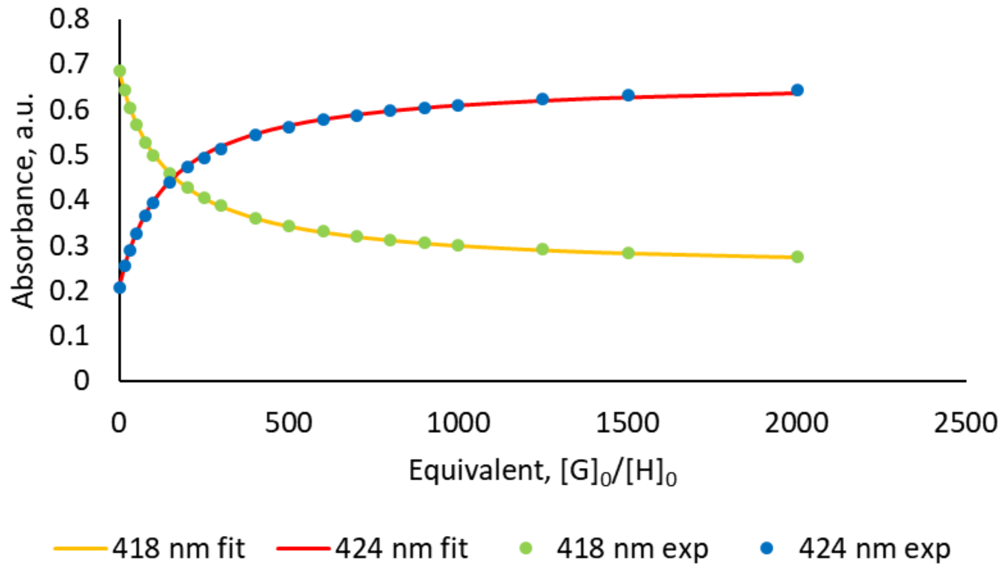

Figure S26 Evaluation of the association constants for 1.36 × 10^{-6} M **3** titrated by (***S,S***)**-cycHC[6]** (up to 2002 eq.) in DCM, K_1 = 5120 ± 70 M^{-1} and parallel experiment with K_1 = 4990 ± 30 M^{-1}(*http://app.supramolecular.org/bindfit/view/307bbf9c-17c2-4af4-8844-9dc1bba9a558* and *http://app.supramolecular.org/bindfit/view/35b29501-b4c7-4388-9207-d8793e6b81f9*).

Binding of Zn(*p*-Cl)TPP (4) with cycHC[*n*]

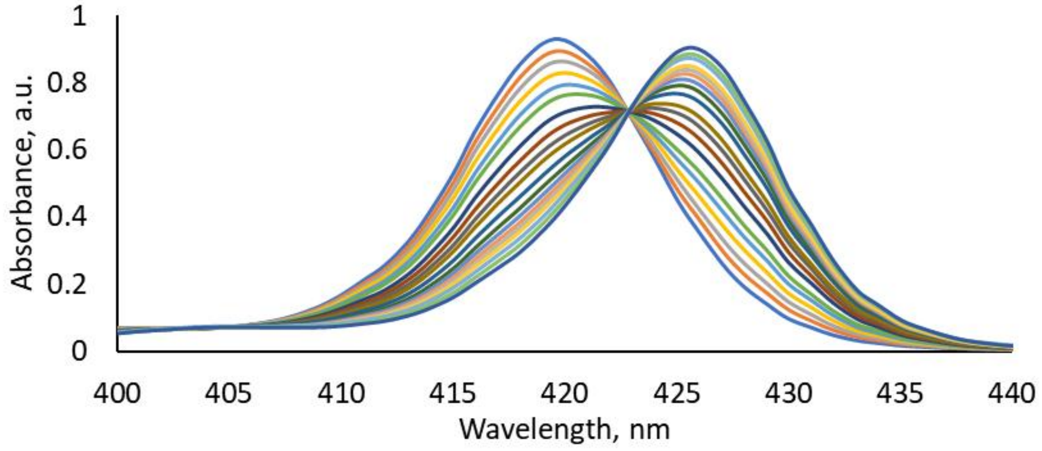

Figure S27 Titration of 1.43 × 10^{-6} M **4** by **(*R,R*)-cycHC[8]** (up to 1968 eq.) in DCM.

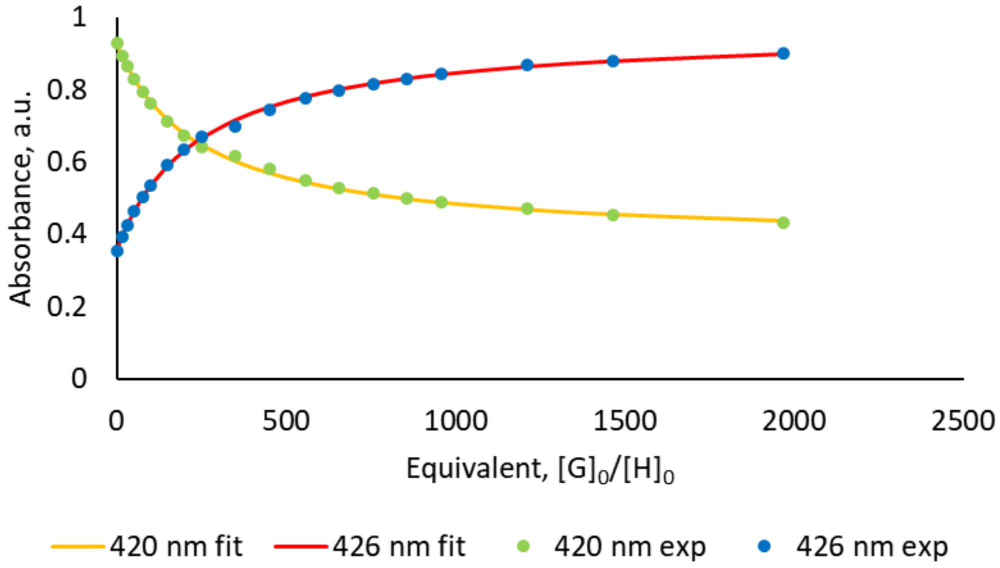

Figure S28 Evaluation of the association constants for 1.43 × 10^{-6} M **4** titrated by **(*R,R*)-cycHC[8]** (up to 1968 eq.) in DCM, K_1 = 2880 ± 50 M^{-1} *(http://app.supramolecular.org/bindfit/view/30bcb526-36c0-4df8-b216-4ba7c39f1e0d).*

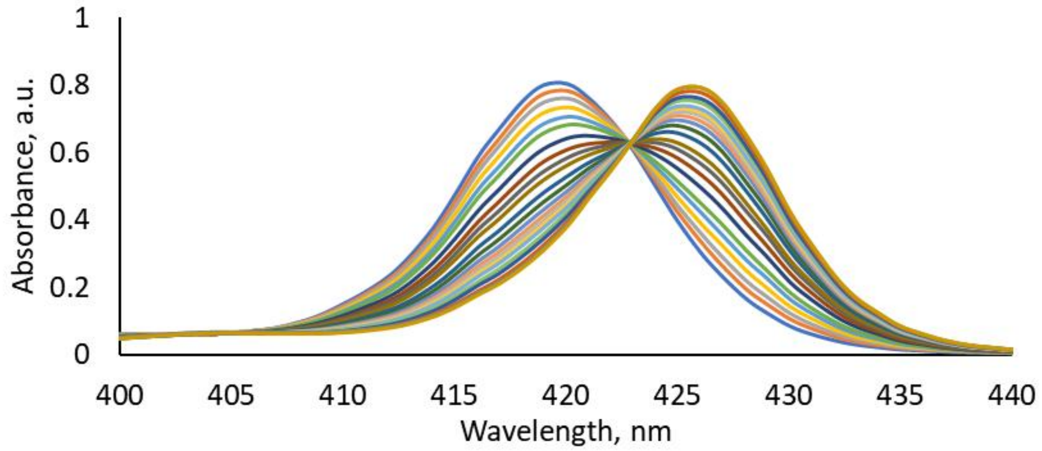

Figure S29 Titration of 1.29 × 10^{-6} M **4** by **(*S*,*S*)-cycHC[8]** (up to 2668 eq.) in DCM.

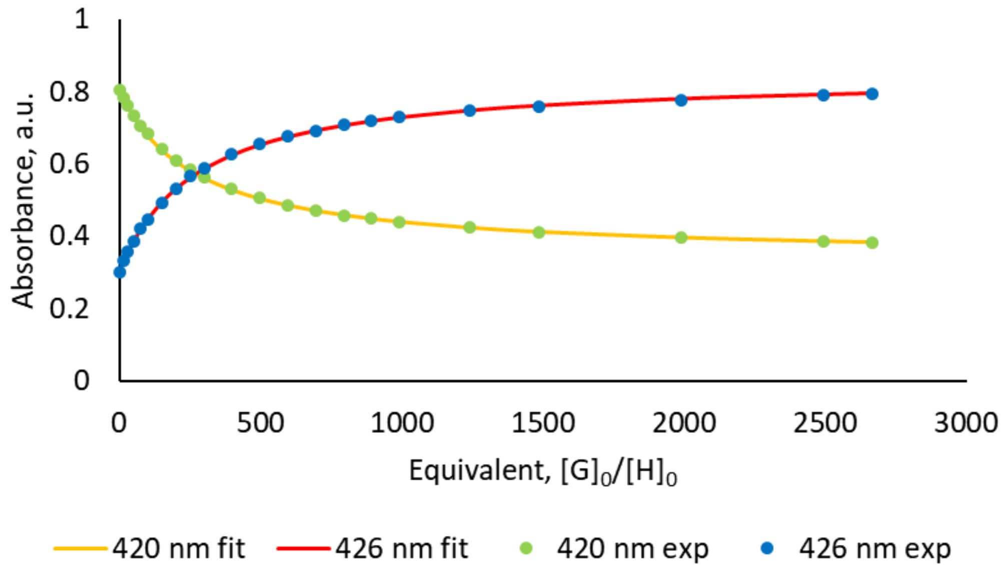

Figure S30 Evaluation of the association constants for 1.29 × 10^{-6} M **4** titrated by **(*S*,*S*)-cycHC[8]** (up to 2668 eq.) in DCM, K_1 = 2860 ± 20 M^{-1} (*http://app.supramolecular.org/bindfit/view/e388a3d4-4099-4893-a56a-1a8fd549e0c9*).

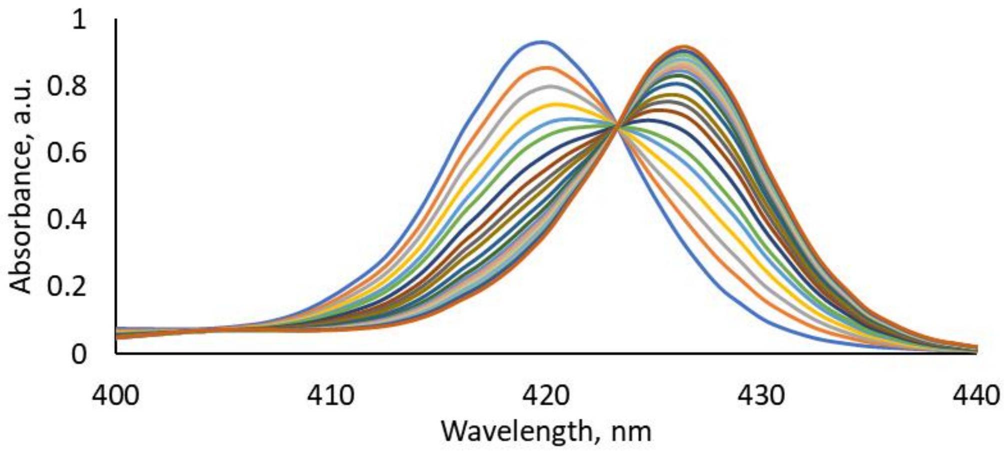

Figure S31 Titration of 1.43 × 10^{-6} M **4** by **(*R*,*R*)-cycHC[6]** (up to 2090 eq.) in DCM.

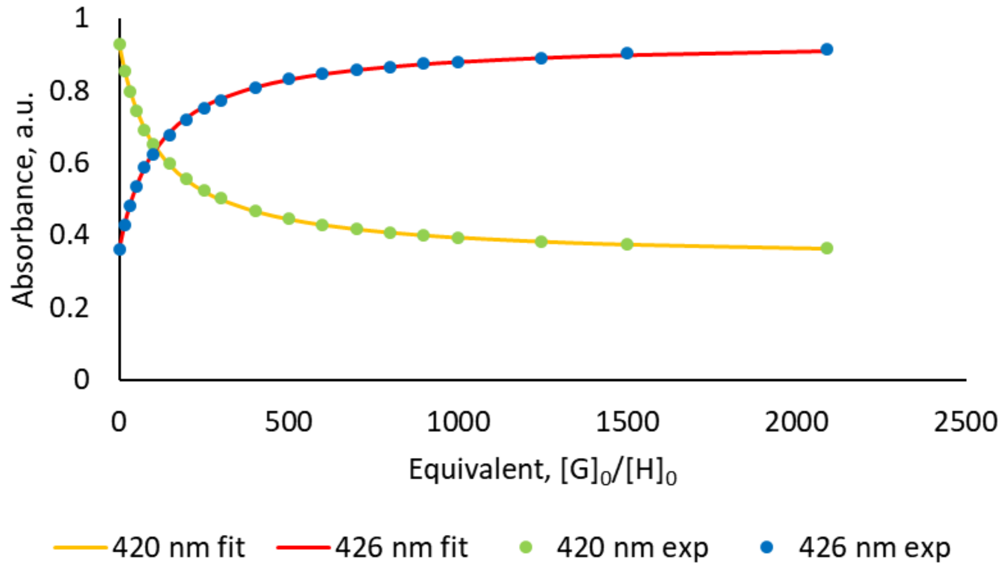

Figure S32 Evaluation of the association constants for 1.43 × 10^{-6} M **4** titrated by **(*R*,*R*)-cycHC[6]** (up to 2090 eq.) in DCM, K_1 = 5980 ± 70 M^{-1} (*http://app.supramolecular.org/bindfit/view/e66ec208-3f32-4eda-b183-e97c5d3b062e*).

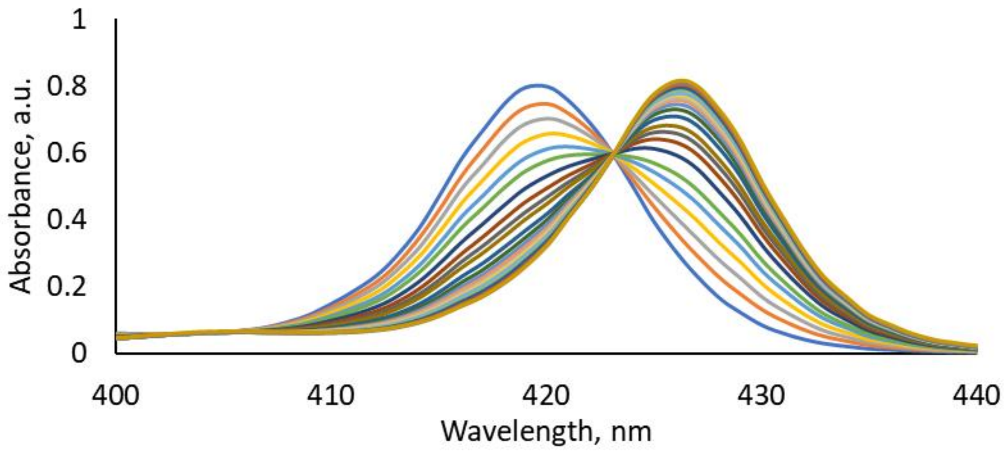

Figure S33 Titration of 1.29 × 10^{-6} M **4** by **(*S,S*)-cycHC[6]** (up to 2620 eq.) in DCM.

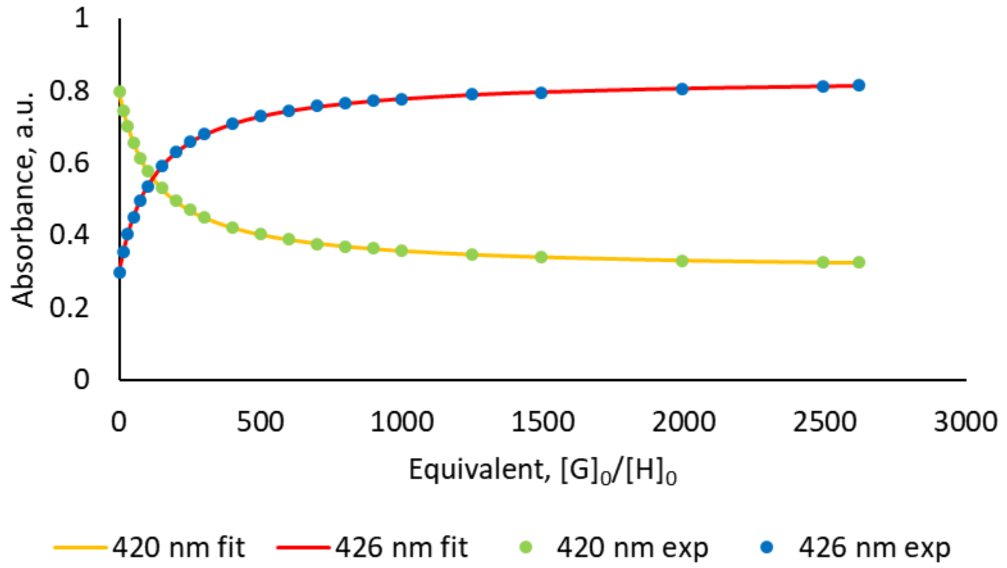

Figure S34 Evaluation of the association constants for 1.29 × 10^{-6} M **4** titrated by **(*S,S*)-cycHC[6]** (up to 2620 eq.) in DCM, K_1 = 6040 ± 30 M^{-1} (*http://app.supramolecular.org/bindfit/view/2edb0f39-1965-4d11-899c-c08b7dc9dba9*).

Binding of Zn(*p*-CF_3)TPP (5) with cycHC[*n*]

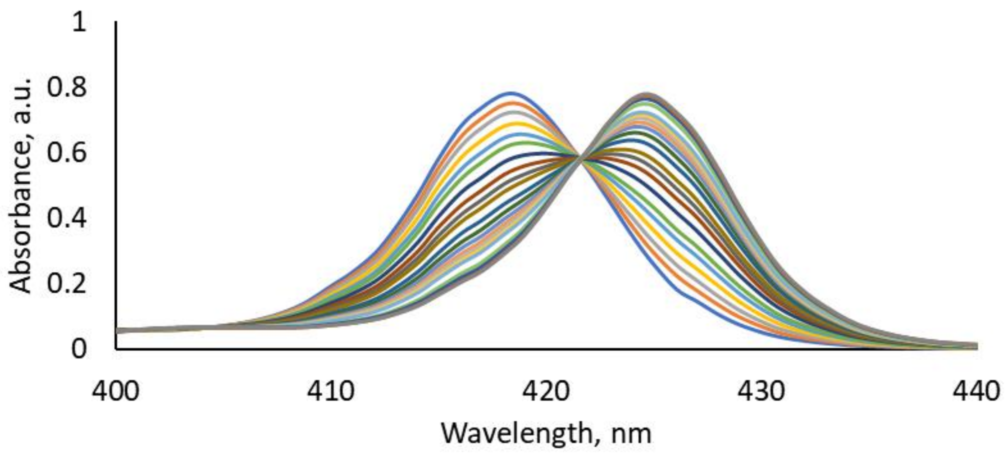

Figure S35 Titration of 1.33 × 10^{-6} M **5** by **(*R,R*)-cycHC[8]** (up to 2769 eq.) in DCM.

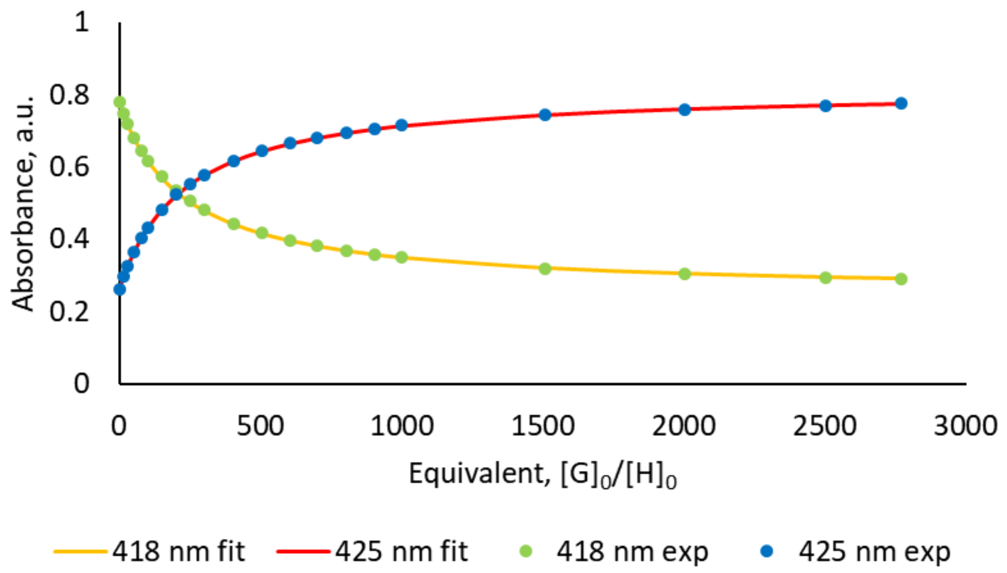

Figure S36 Evaluation of the association constants for 1.33 × 10^{-6} M **5** titrated by **(*R,R*)-cycHC[8]** (up to 2769 eq.) in DCM, K_1 = 3340 ± 20 M^{-1} (*http://app.supramolecular.org/bindfit/view/e20fbead-1121-4af7-a354-852283c2d93b*).

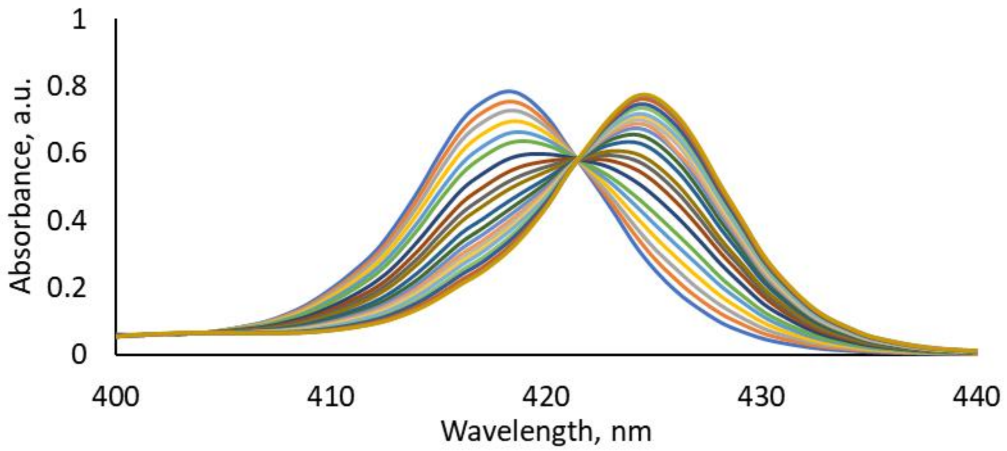

Figure S37 Titration of 1.33 × 10^{-6} M **5** by ***(S,S)*-cycHC[8]** (up to 2739 eq.) in DCM.

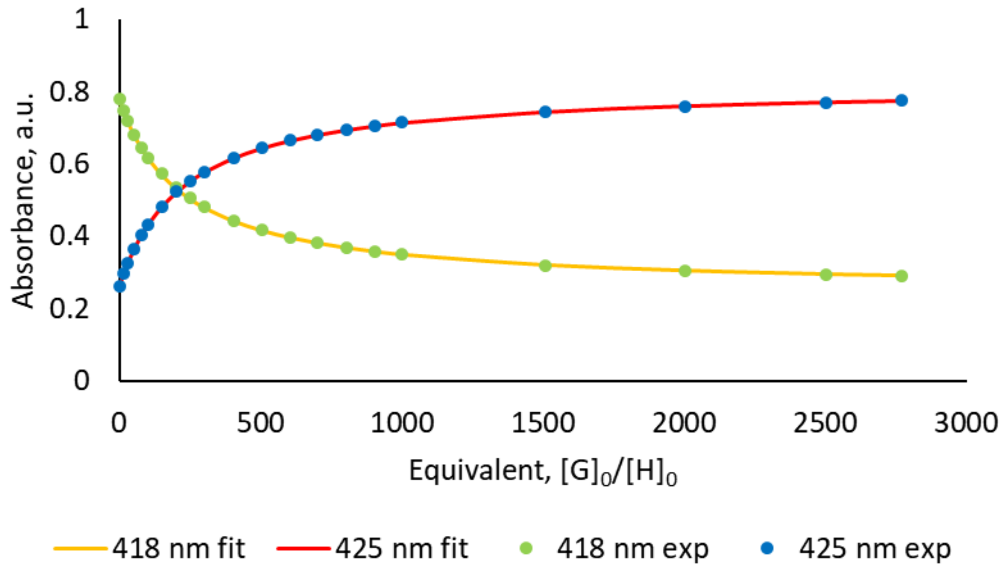

Figure S38 Evaluation of the association constants for 1.33 × 10^{-6} M **5** titrated by ***(S,S)*-cycHC[8]** (up to 2739 eq.) in DCM, K_1 = 3340 ± 10 M^{-1} (*http://app.supramolecular.org/bindfit/view/2057f151-34d2-4448-a320-8acd3bc39007*).

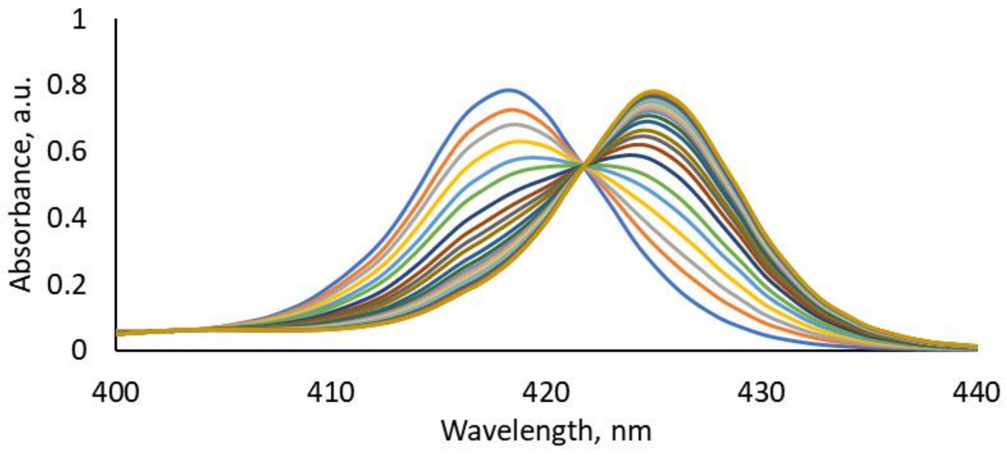

Figure S39 Titration of 1.33 × 10^{-6} M **5** by **(*R,R*)-cycHC[6]** (up to 2684 eq.) in DCM.

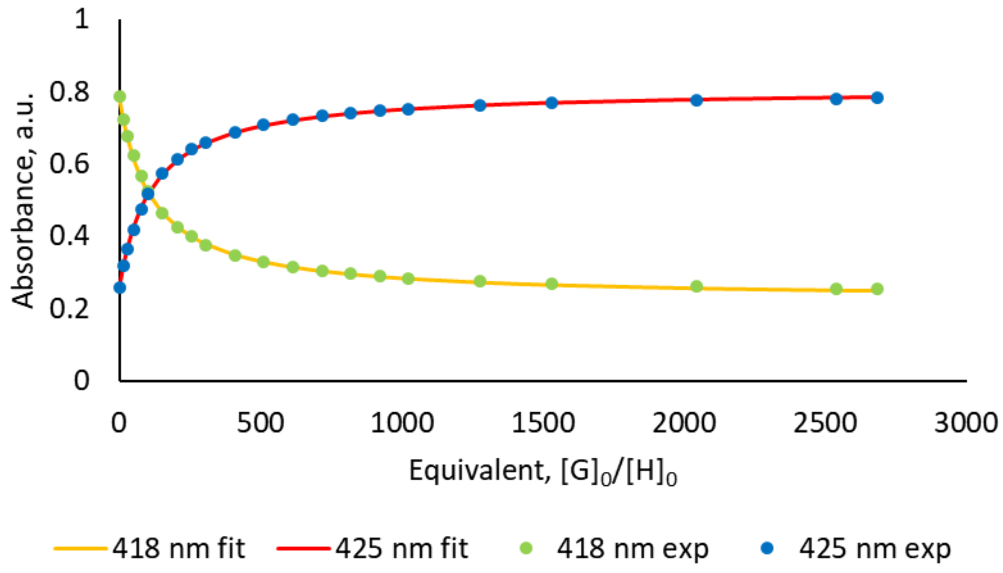

Figure S40 Evaluation of the association constants for 1.33 × 10^{-6} M **5** titrated by **(*R*,*R*)-cycHC[6]** (up to 2684 eq.) in DCM, K_1 = 6630 ± 80 M^{-1} and parallel experiment with K_1 = 7400 ± 100 M^{-1} (*http://app.supramolecular.org/bindfit/view/c3b245ac-02c4-46eb-b730-8c83304d3c8c* and *http://app.supramolecular.org/bindfit/view/858794c3-1d92-4a20-971f-f66ef204322c*).

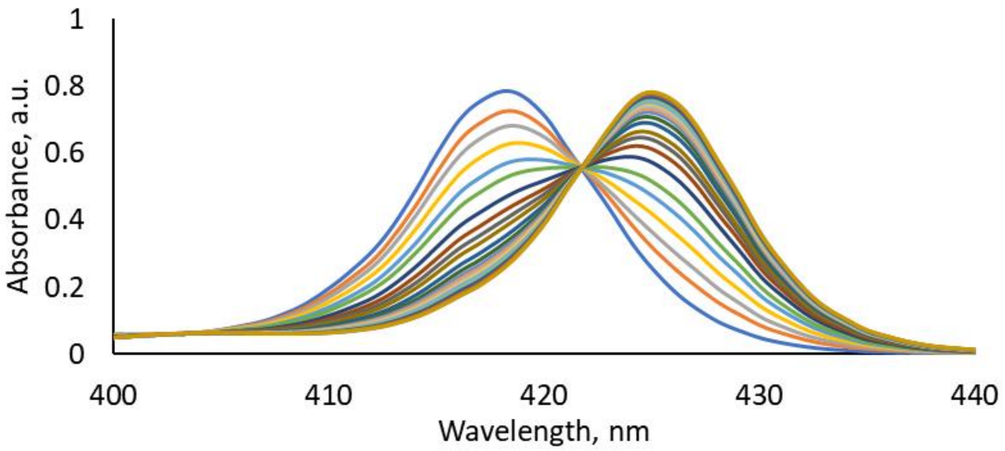

Figure S41 Titration of 1.33 × 10^{-6} M **5** by **(*S,S*)-cycHC[6]** (up to 2810 eq.) in DCM.

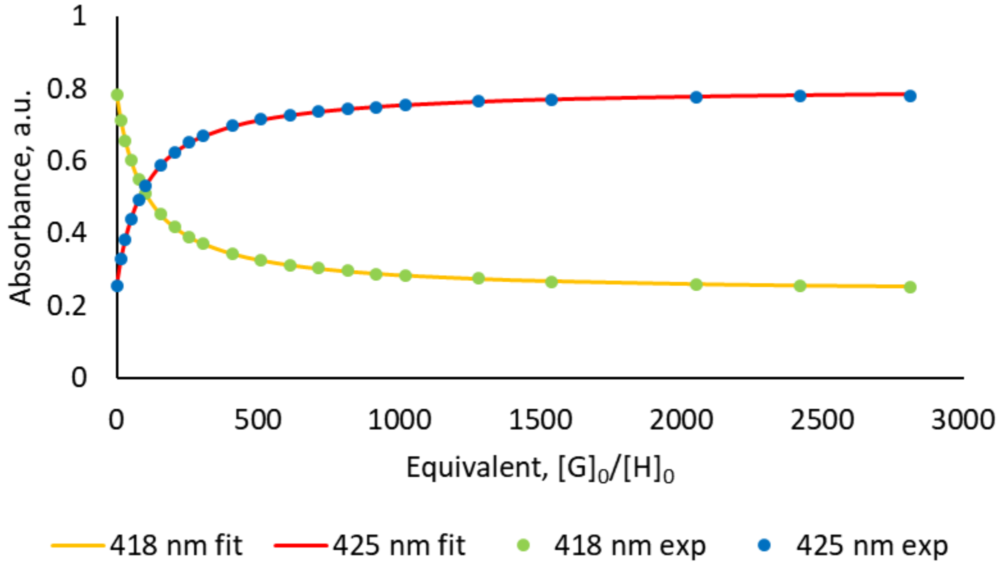

Figure S42 Evaluation of the association constants for 1.33 × 10^{-6} M **5** titrated by **(*S,S*)-cycHC[6]** (up to 2810 eq.) in DCM, K_1 = 7540 ± 50 M^{-1} and parallel experiment with K_1 = 7880 ± 90 M^{-1} (*http://app.supramolecular.org/bindfit/view/87afffc9-fa5f-45da-bd55-306887db07eb* and *http://app.supramolecular.org/bindfit/view/5987661f-983e-42c2-88a9-1779a4933432*).

Binding of ZnTPFPOBP (6) with cycHC[*n*]

The titration data for binding of **6** could not be reasonably fit using the Bindfit software, most likely due to a pronounced presence of complexes with stoichiometry higher than 2:1. However, only ca. four equivalents of macrocycle were needed to reach the plateau of titration curve in comparison with ca. 1000 equivalents needed for porphyrins **1**–**5.** This indicates orders of magnitude stronger binding for **6**.

Estimation of K_1 was obtained from fitting with 2:1 previously published binding model[3] (article table 1 line 6).

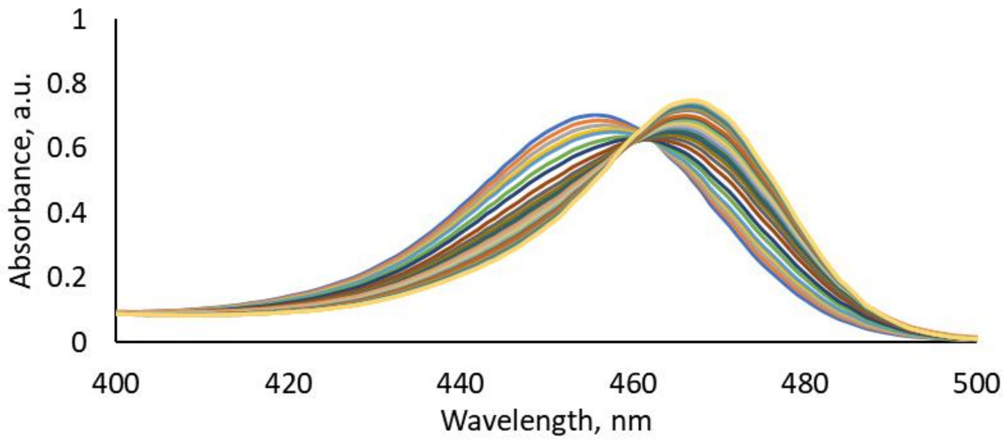

Figure S43 Titration of 3.47 × 10^{-6} M **6** by **(*R,R*)-cycHC[8]** (up to 15.45 eq.) in DCM.

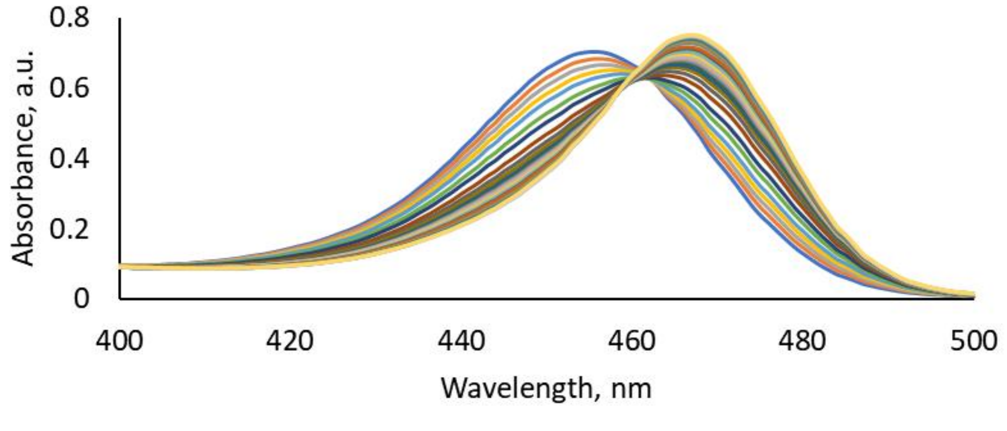

Figure S44 Titration of 3.47 × 10^{-6} M **6** by **(*S,S*)-cycHC[8]** (up to 17.31 eq.) in DCM.

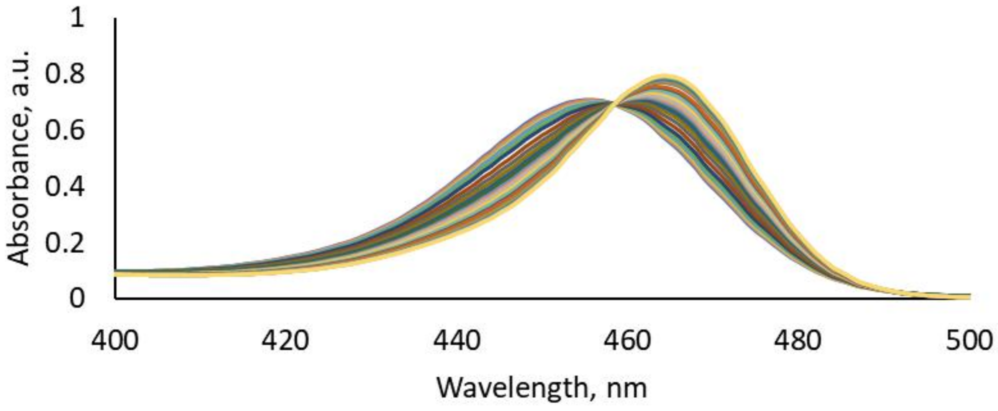

Figure S45 Titration of 3.47 × 10^{-6} M **6** by **(*R,R*)-cycHC[6]** (up to 18.24 eq.) in DCM.

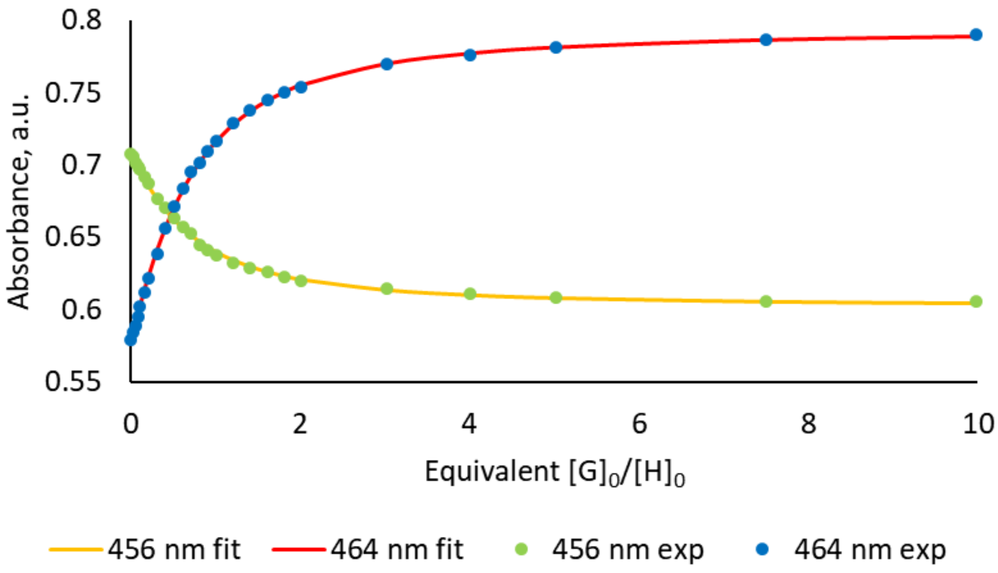

Figure S46 Evaluation of the association constants for 3.47 × 10^{-6} M **6** with addition of **(*R,R*)-cycHC[6]** (up to 10 eq.) in DCM, K_1 = (3.99 ± 0.04) · 10^5 M^{-1} (http://app.supramolecular.org/bindfit/view/afd394c3-5c99-47ab-b5ac-40ee84e939c0).

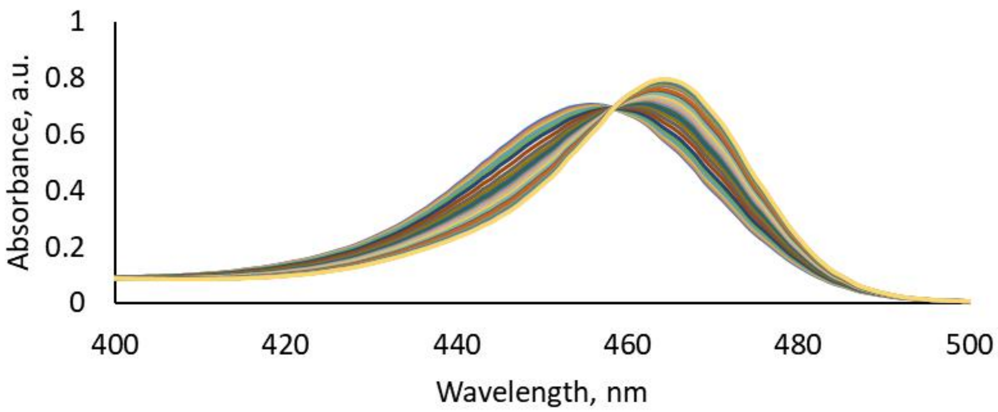

Figure S47 Titration of 3.47 × 10^{-6} M **6** by **(*S,S*)-cycHC[6]** (up to 18.37 eq.) in DCM.

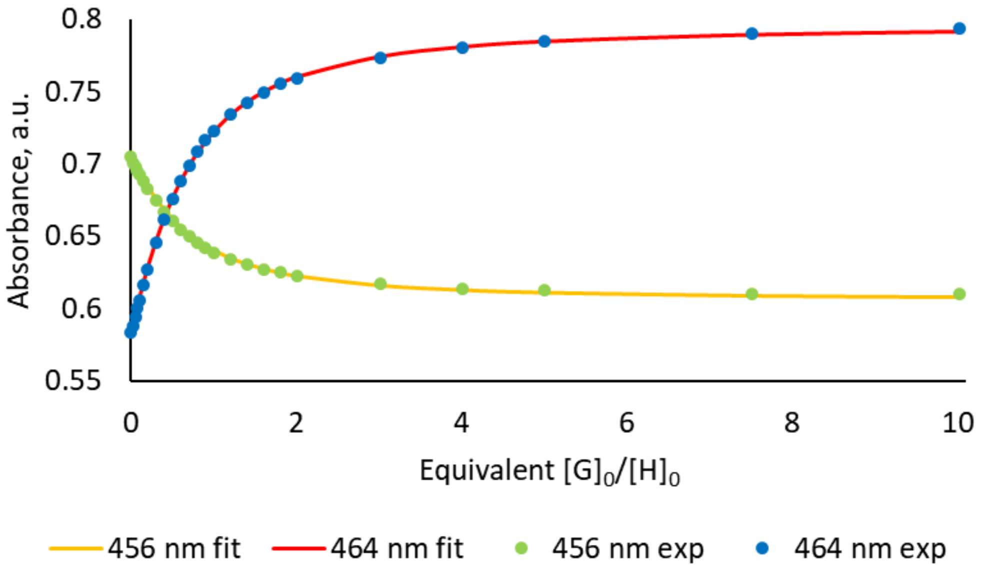

Figure S48 Evaluation of the association constants for 3.47 × 10^{-6} M **6** with addition of **(*S,S*)-cycHC[6]** (up to 10 eq.) in DCM, K_1 = (4.18 ± 0.04) · 10^5 M^{-1} (http://app.supramolecular.org/bindfit/view/a2e02e0e-82d7-4970-bbff-d28006837dbb).

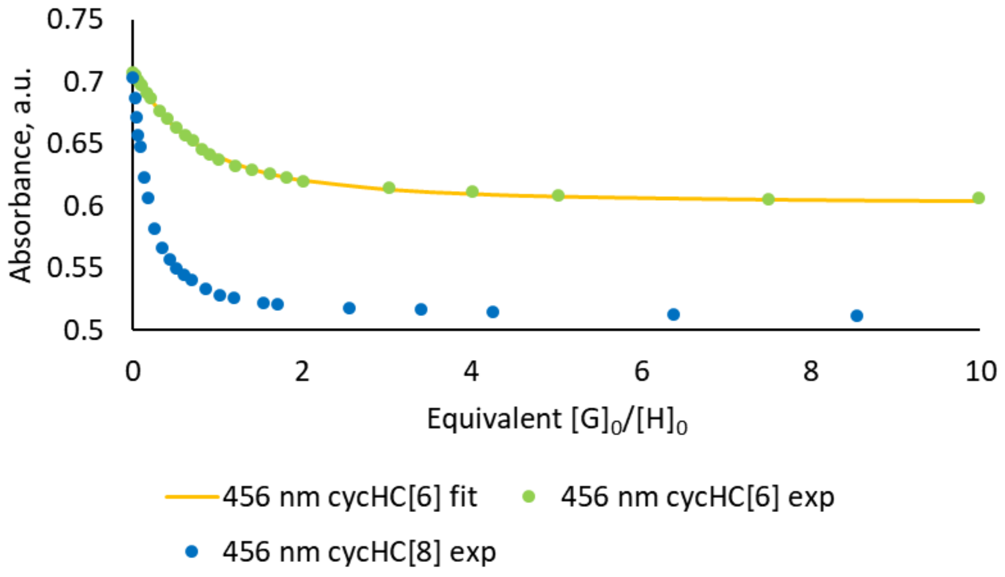

Figure S49 Experimental points of 3.47 × 10^{-6} M **6** binding to (*R*,*R*)-cycHC[8] (blue points) and to (*R*,*R*)-cycHC[6] (green points) with fitted line for the latter (yellow) at 456 nm.

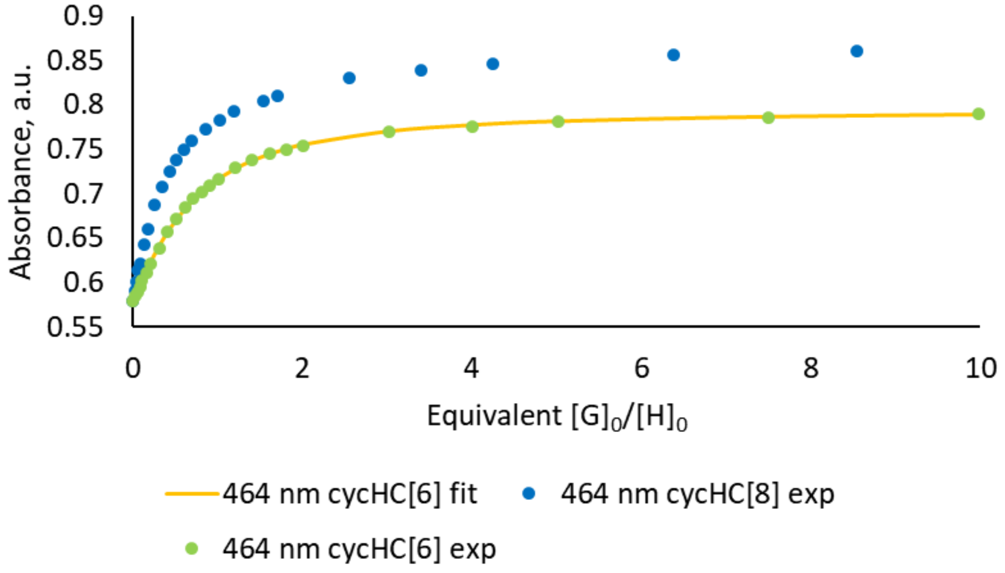

Figure S50 Experimental points of 3.47 × 10^{-6} M **6** binding to (*R*,*R*)-cycHC[8] (blue points) and to (*R*,*R*)-cycHC[6] (green points) with fitted line for the latter (yellow) at 464 nm.

Binding of MgTPP (7) with cycHC[*n*]

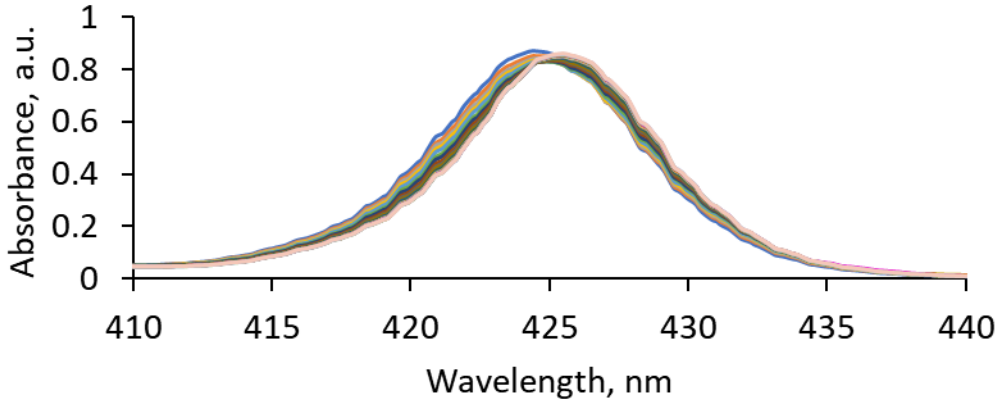

Figure S51 Titration of 1.39 × 10^{-6} M **7** by **(*R,R*)-cycHC[8]** (up to 100 eq.) in DCM.

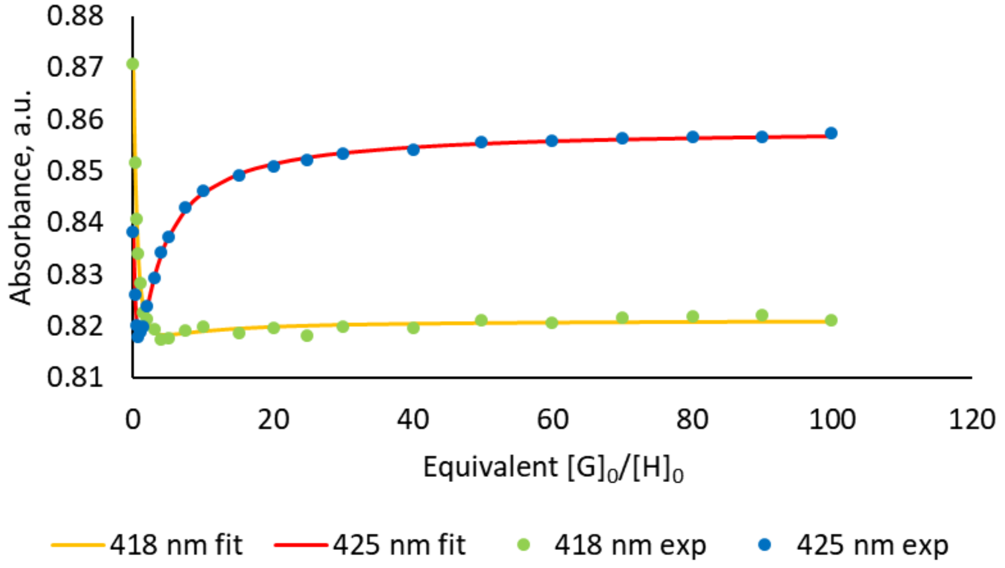

Figure S52 Evaluation of the association constants for 1.39 × 10^{-6} M **7** titrated by **(*R,R*)-cycHC[8]** (up to 100 eq.) in DCM, K_1 = (104 ± 4) · 10^4 M^{-1} K_2 = (47 ± 1) · 10^4 M^{-1} (http://app.supramolecular.org/bindfit/view/7b98311d-3ce4-4955-864a-fa6d4db7aa8d).

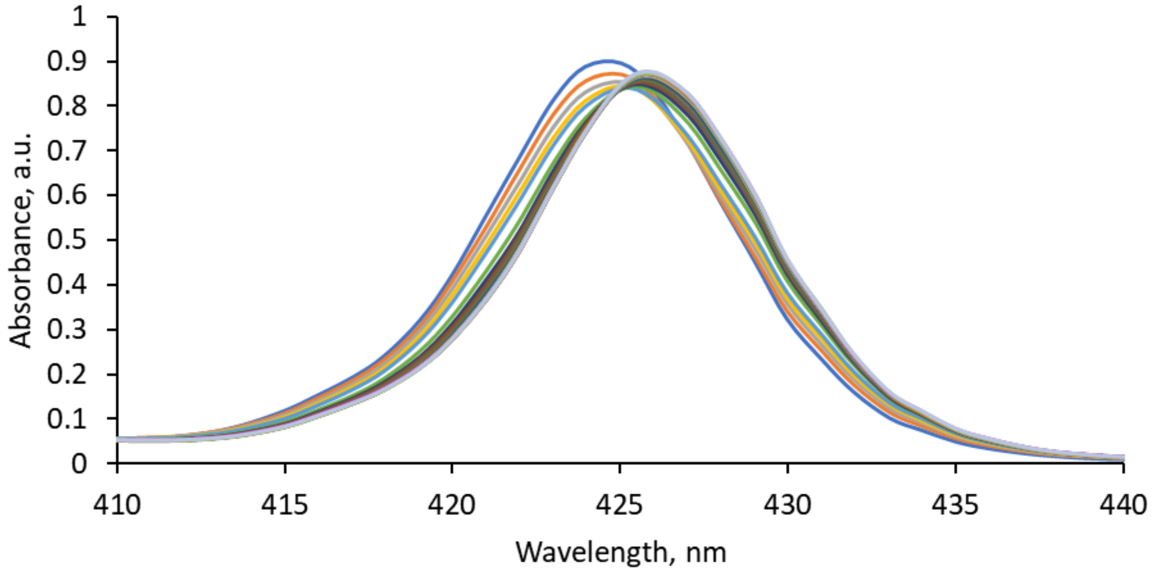

Figure S53 Evaluation of the association constants for 1.39 × 10^{-6} M **7** titrated by **(*R*,*R*)-cycHC[6]** (up to 100 eq.) in DCM.

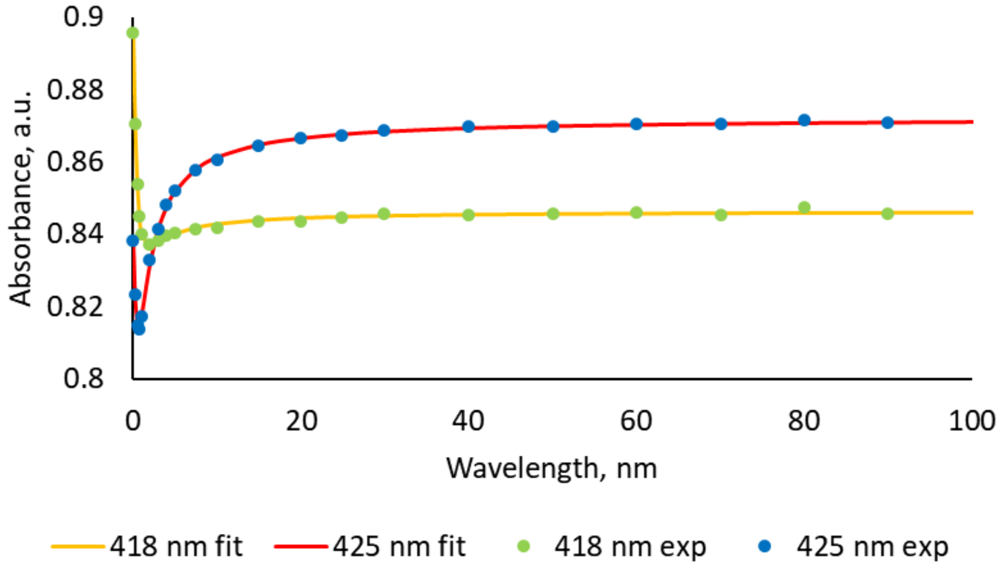

Figure S54 Evaluation of the association constants for 1.39 × 10^{-6} M **7** titrated by **(*R*,*R*)-cycHC[6]** (up to 100 eq.) in DCM, K_1 = (184 ± 7) · 10^4 M^{-1} K_2 = (47 ± 1) · 10^4 M^{-1} (http://app.supramolecular.org/bindfit/view/71af0fb5-94e9-4cc8-8649-74a0bd2ee2c1).

Titration of FeClOEP (8) with cycHC[8]

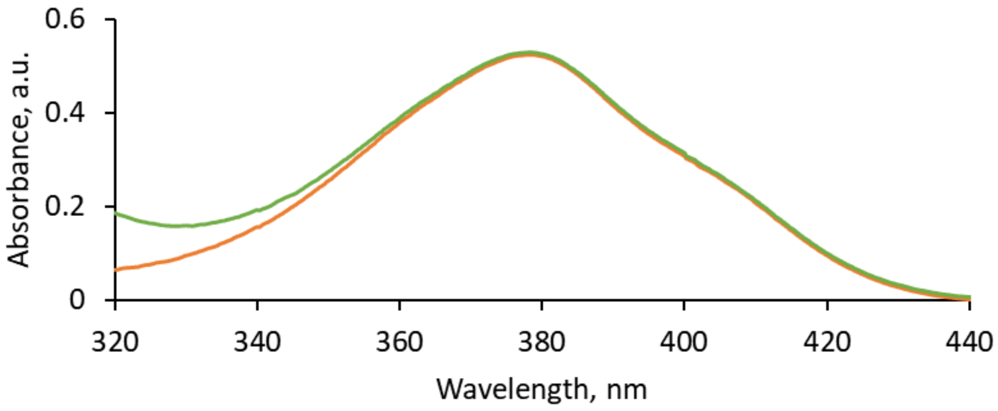

Figure S55 Spectrum of 5.5 × 10^{-6} M **8** (orange) with single addition of 895 equivalent of (*R,R*)-cycHC[8] (green) in DCM, no binding. The difference at 320 nm is due to the shoulder of (*R,R*)-cycHC[8] signal.

Titration of PdOEP (9) with cycHC[8]

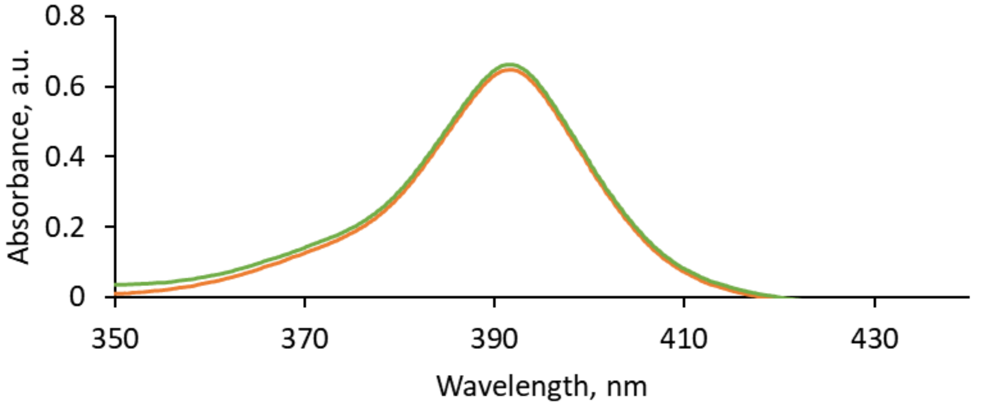

Figure S56 Spectrum of 4.5 × 10^{-6} M **9** (orange) with single addition of 1255 equivalent of (*R,R*)-cycHC[8] (green) in DCM, too weak binding to be determined.

2.3 ^{1}H NMR titration studies of MgTPP (7) with cycHC[*n*]

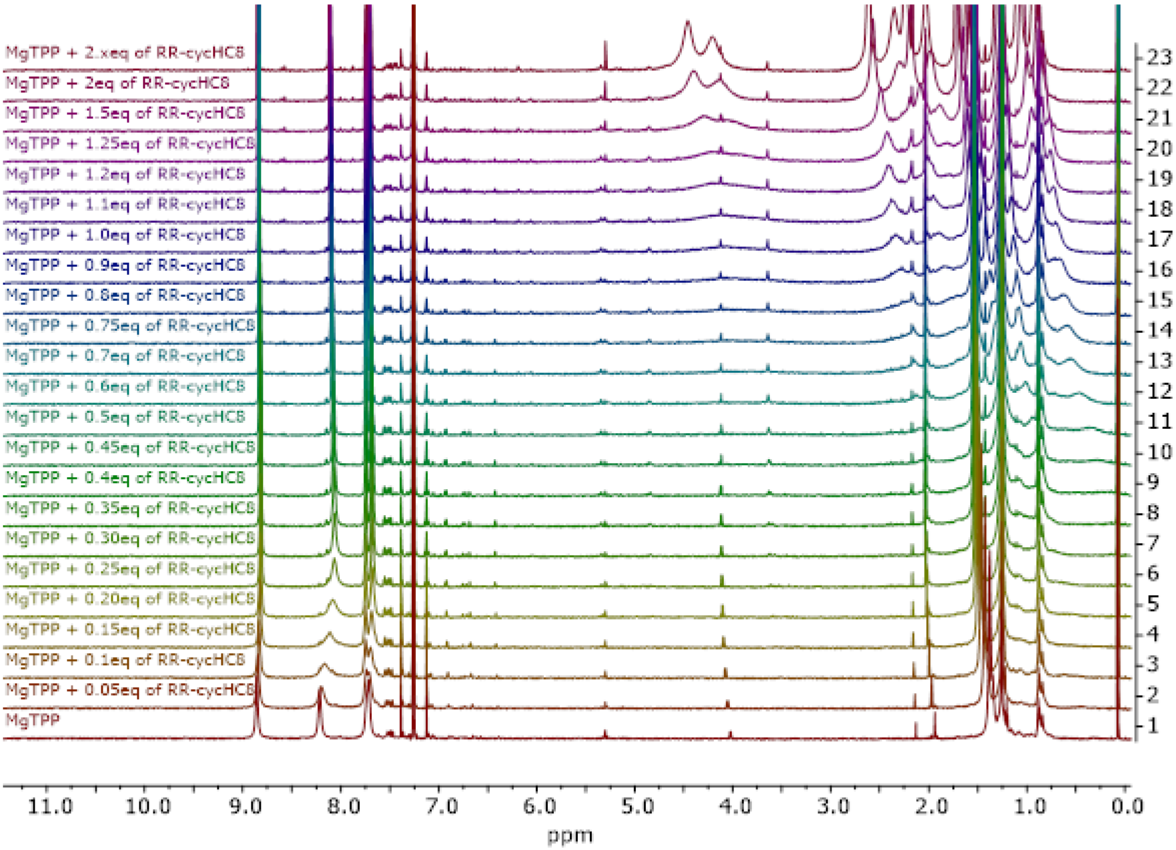

Figure S57 Full spectra of 0.626 × 10^{-3} M **7** titration by **(*R,R*)-cycHC[8]** from 0 to 2.43 eq. measured in $CDCl_3$ at 800MHz.

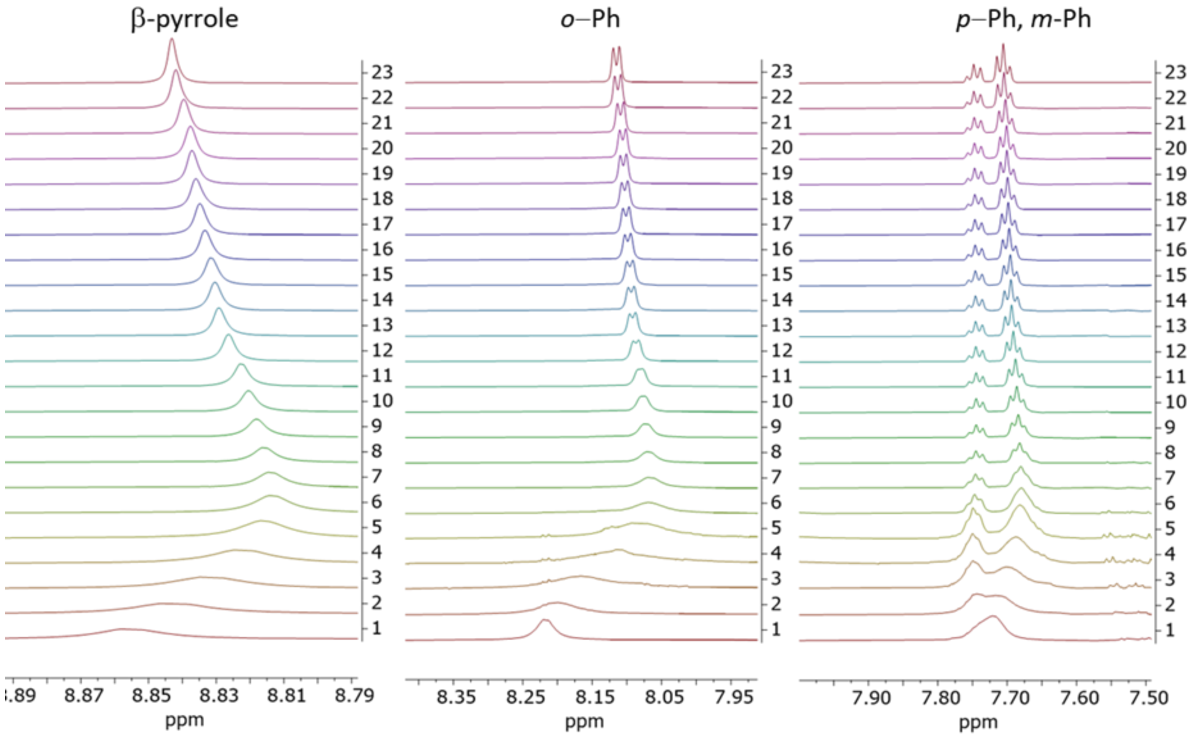

Figure S58 Change in chemical shifts of 0.626 × 10^{-3} M **7** ^{1}H NMR signals upon addition of **(*R,R*)-cycHC[8]** from 0 to 2.43 eq. measured in $CDCl_3$ at 800MHz.

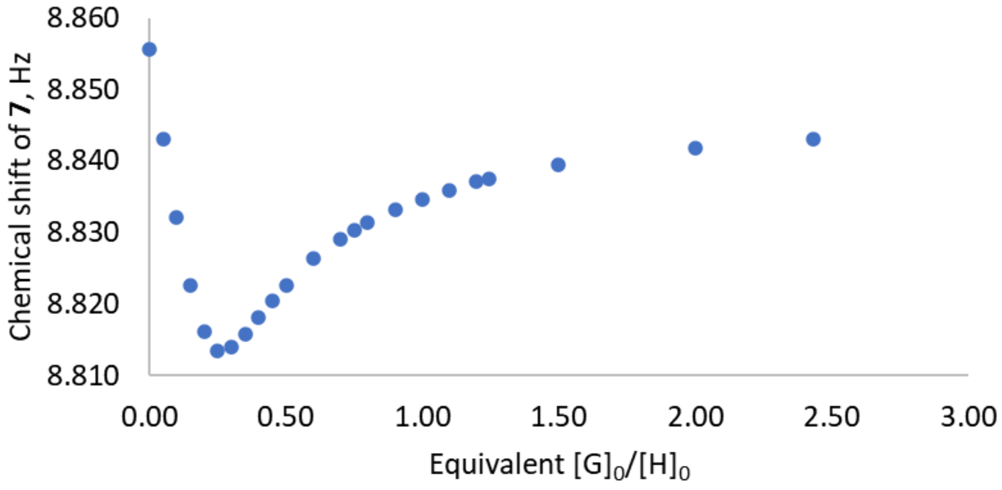

Figure S59 Change of *β*-pyrrole signals of 0.626 × 10^{-3} M **7** upon addition of **(*R,R*)-cycHC[8]** from 0 to 2.43 eq.

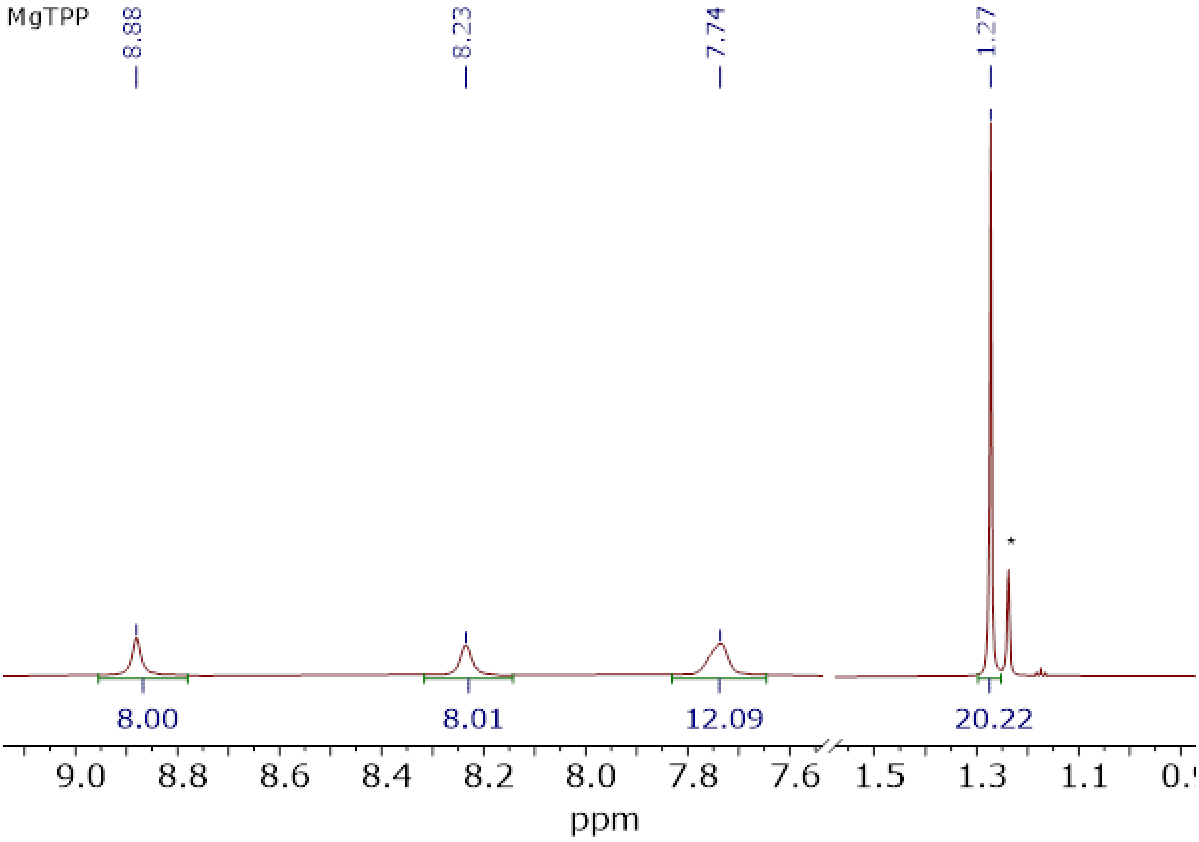

Figure S60 Integrals of 2.34 × 10^{-3} M MgTPP signals (δ 8.88, 8.23 and 7.74 ppm) show presence of ca 10 equivalents of water (1.27 ppm) in the sample in $CDCl_3$ at 800MHz. * - impurity from polyethylene.

The **MgTPP** (**7**) sample is titrated in presence of small amount of water, where **7** is fully complexed to H_2O in spectra 1 of **Figure S57**. Upon titration with **(*R,R*)-cycHC[8]**, the H_2O is exchanged by **(*R,R*)-cycHC[8]** and chemical shift of **7** is changing direction of signal shifting around 0.25 – 0.35 eq. of **(*R,R*)-cycHC[8]**, it is likely there are 3 or 4 porphyrins bound to one **(*R,R*)-cycHC[8]** macrocycle at that equivalency. Further additions of **(*R,R*)-cycHC[8]** forces it to a 1:1 complex. We fitted the data with 2:1 non-cooperative binding model, while excluding the first 7 titration points (**Figure S61**) and got apparent association constant K_1(app) = 39050 ± 780 M^{-1}. The real association constant for **(*R,R*)-cycHC[8]** for 1:1 complex of **(*R,R*)-cycHC[8]** and **7** is higher, due to competitive binding of H_2O to **7**.

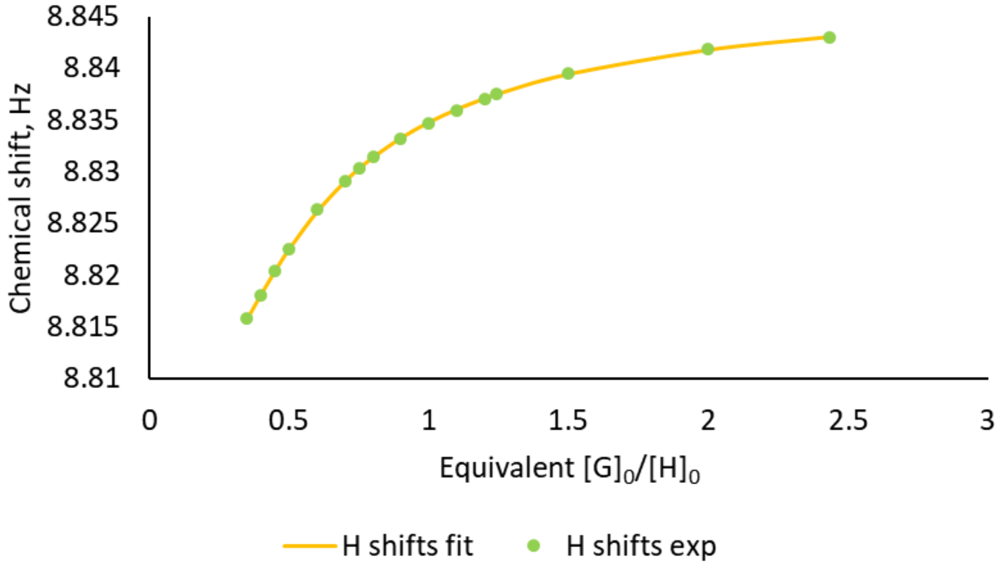

Figure S61 Evaluation of the apparent association constants for 0.626 mM **7** binding with **(*R,R*)-cycHC[8]** (from 0.35 to 2.43 eq.) in $CDCl_3$, $K_{1\ apparent}$ = (391 ± 8) 10^2 M^{-1} in presence of water.

3. Chiroptical properties (UV-Vis and CD) of complexes in DCM

CD spectra and high-tension voltage was registered right after UV-Vis absorption measurements in a 10 mm cuvette. Porphyrin concentration was adjusted to provide absorbance close to 1 and enough of a cyclohexanohemicucurbituril (cycHC[*n*]) was added to have porphyrin fully complexed. *g*-factors were calculated according to equation 1:

$$g = \frac{CD}{A \cdot 32980} \qquad (1)$$

where *g* stands for dissymmetry factor, *CD* for the recorded CD in mdeg, *A* the recorded absorbance and 32980 is a constant that comes from converting radians to degrees.

As the complexes with enantiomers were considered as parallel experiments, averages of absolute values of *g*-factors were taken and presented in **Table S3**.

Table S3 The UV-vis λ_{max}, ECD λ_{max} and *g*-factors at ECD λ_{max} values for porphyrin-cycHC[*n*] 1:1 complexes in DCM. (+ and – Cotton effects are assigned for complexes with (*R*,*R*)-cycHC[*n*] while *g*-factor is average of porphyrin complexes with both enantiomers os cycHC[*n*]

No	Complex	UV-vis λ_{max}, nm	ECD λ_{max} of complex, nm	|*g*|-factor, 10^{-5}
1	**1**·cycHC[8]	408	407 (+)	3.9
2	**1**·cycHC[6]	409	407 (+)	3.8
3	**2**·cycHC[8]	424	422 (+)/429 (-)	3.5/2.3
4	**2**·cycHC[6]	425	423 (+)/430 (-)	2.8/2.3
5	**3**·cycHC[8]	424	422 (+)/429 (-)	3.5/3.6
6	**3**·cycHC[6]	425	422 (+)/429 (-)	3.8/3.1
7	**4**·cycHC[8]	426	424 (+)/433 (-)	3.4/3.8
8	**4**·cycHC[6]	426	424 (+)/432 (-)	3.3/3.6
9	**5**·cycHC[8]	425	422 (+)/429 (-)	3.7/3.4
10	**5**·cycHC[6]	425	422 (+)/429 (-)	3.7/3.3
11	**6**·cycHC[8]	467	468 (+)	7.9
12	**6**·cycHC[6]	464	469 (+)	6.4
13	**7**·cycHC[8]	425	422 (+)/429 (-)	4.5/3.0
14	**7**·cycHC[6]	426	422 (+)/429 (-)	4.8/3.9

UV-Vis and CD of ZnOEP (1) complexes with cycHC[*n*]

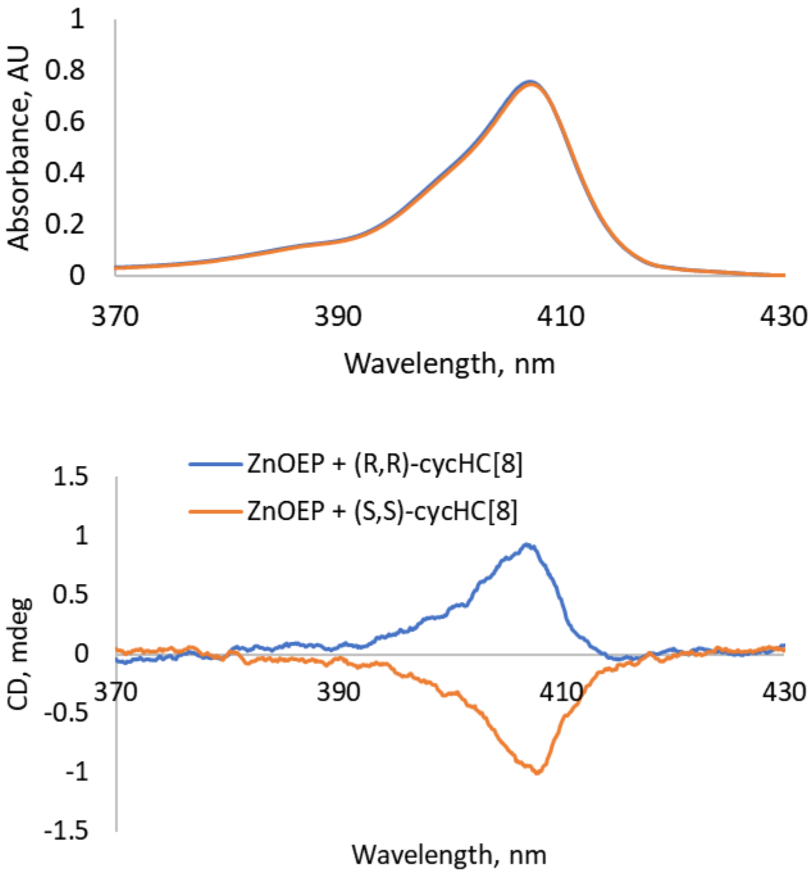

Figure S62 Absorbance and CD signal of 2.54 × 10^{-6} M **1** complex with **(*R,R*)-cycHC[8]** (3493 eq.) (blue) and with **(*S,S*)-cycHC[8]** (orange) (3200 eq.) in DCM.

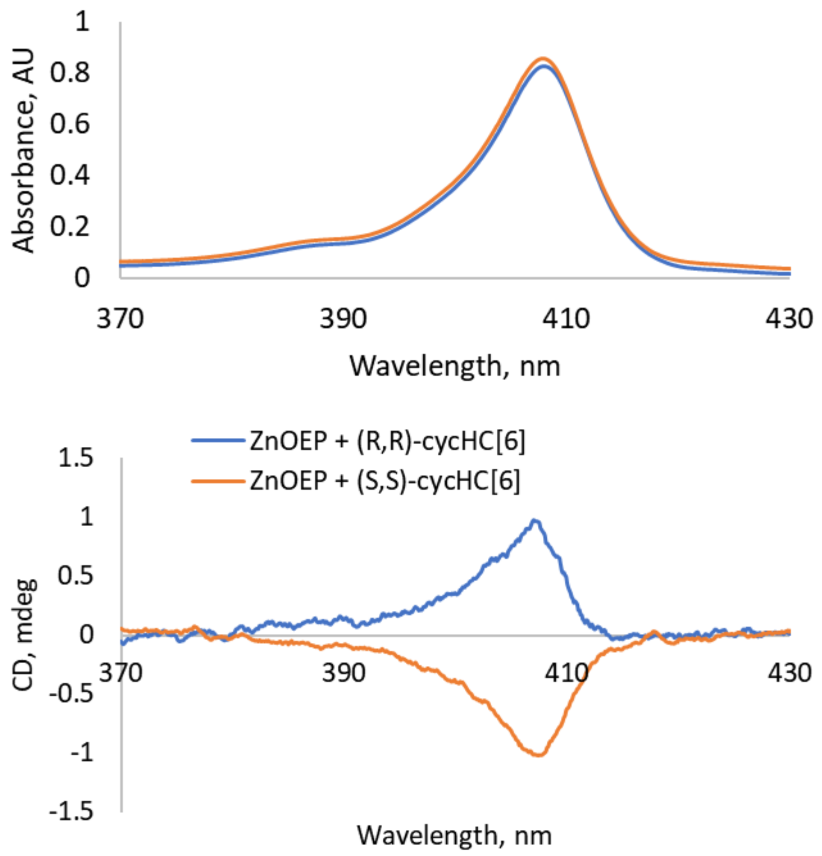

Figure S63 Absorbance and CD signal of 2.54 × 10^{-6} M **1** complex with ***(R,R)*-cycHC[6]** (3386 eq.) (blue) and with ***(S,S)*-cycHC[6]** (orange) (3216 eq.) in DCM.

UV-Vis and CD of ZnTPP (2) complexes with cycHC[*n*]

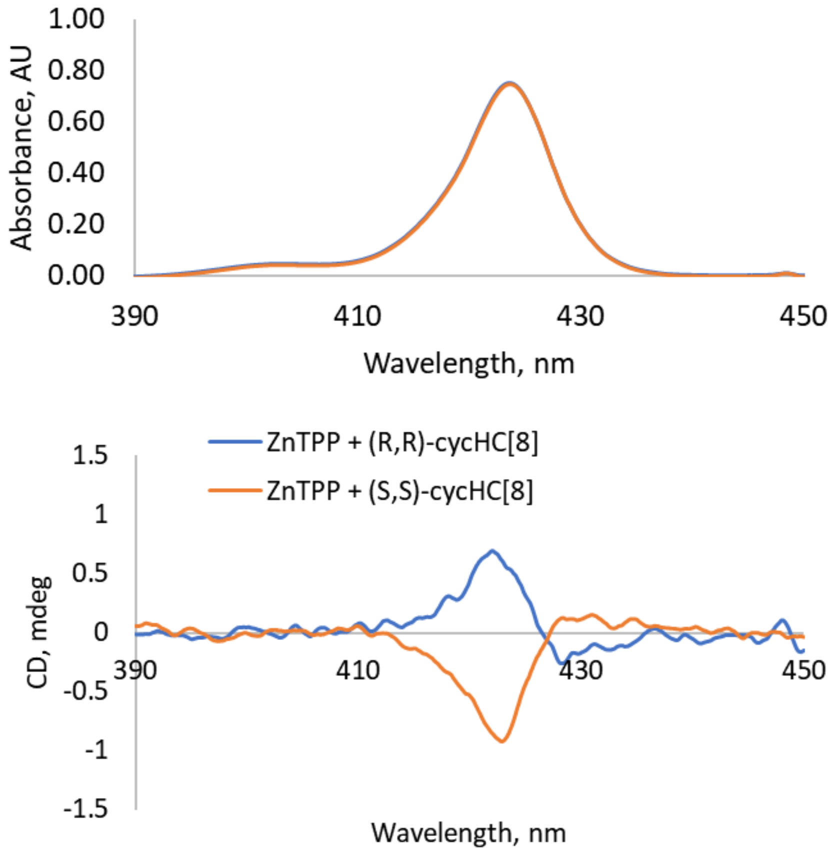

Figure S64 Absorbance and CD signal of 1.36 × 10^{-6} M **2** complex with **(*R*,*R*)-cycHC[8]** (2639 eq.) (blue) and with **(*S*,*S*)-cycHC[8]** (orange) (2001 eq.) in DCM.

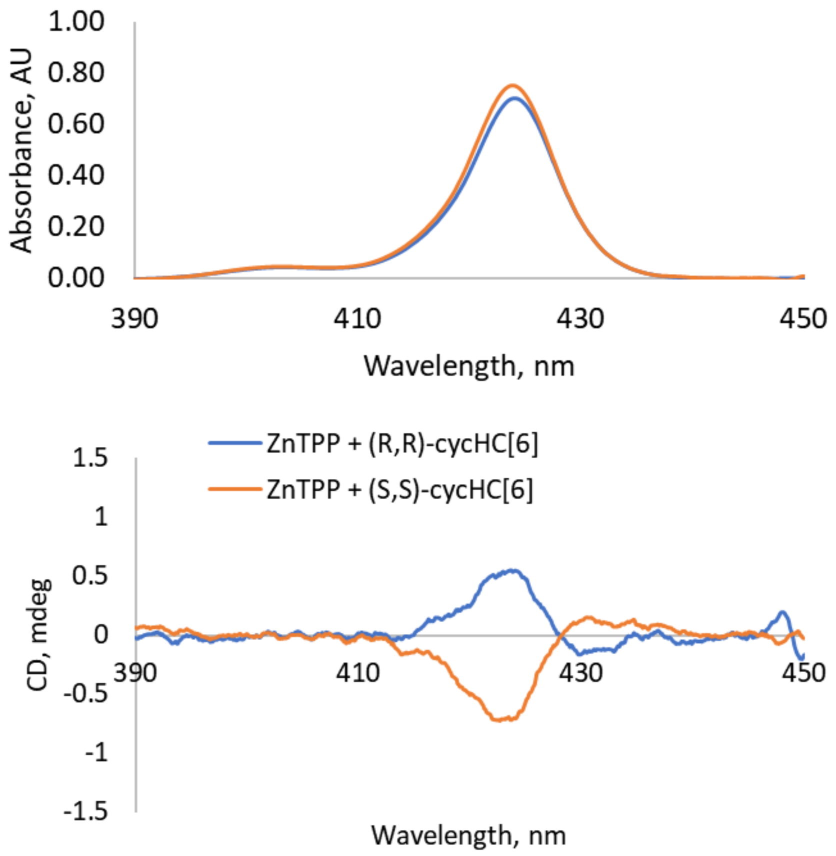

Figure S65 Absorbance and CD signal of 1.36 × 10^{-6} M **2** complex with **(*R,R*)-cycHC[6]** (2672 eq.) (blue) and with **(*S,S*)-cycHC[6]** (orange) (2002 eq.) in DCM.

UV-Vis and CD of Zn(*p*-F)TPP (3) complexes with cycHC[*n*]

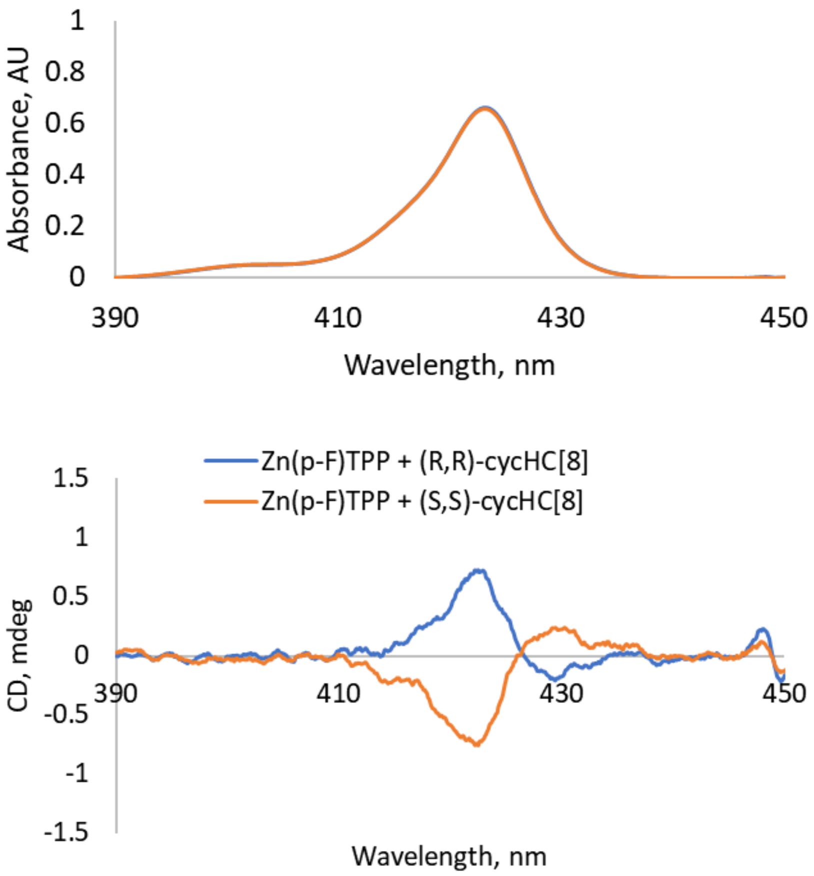

Figure S66 Absorbance and CD signal of 1.37 × 10^{-6} M **3** complex with **(*R,R*)-cycHC[8]** (2639 eq.) (blue) and with **(*S,S*)-cycHC[8]** (orange) (2001 eq.) in DCM.

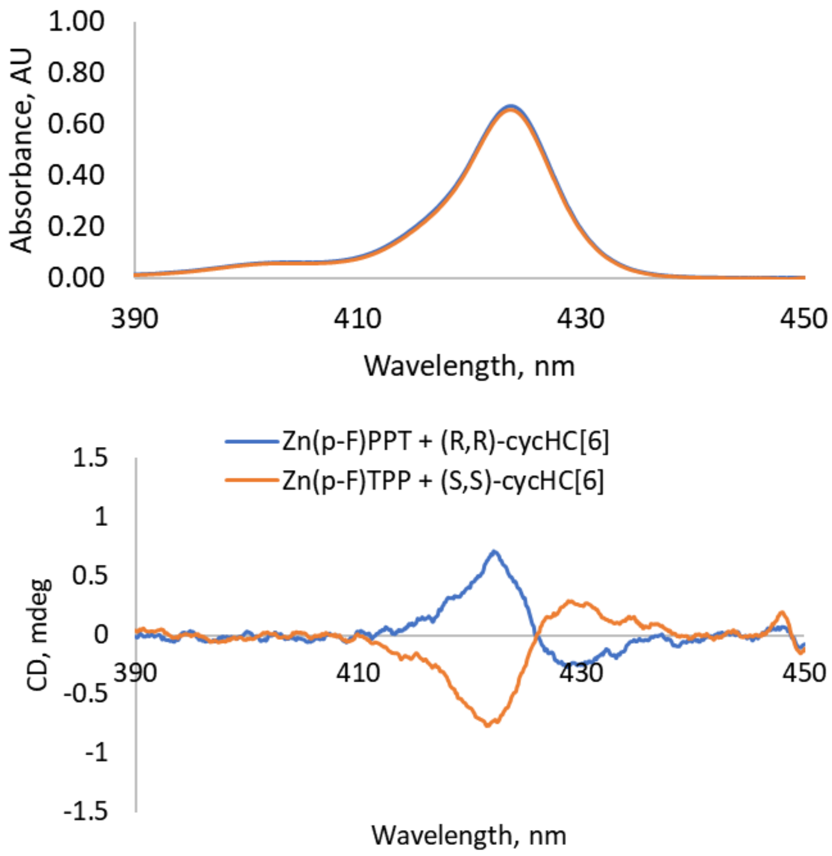

Figure S67 Absorbance and CD signal of 1.37 × 10^{-6} M **3** complex with **(*R,R*)-cycHC[6]** (2672 eq.) (blue) and with **(*S,S*)-cycHC[6]** (orange) (2002 eq.) in DCM.

UV-Vis and CD of Zn(*p*-Cl)TPP (4) complexes with cycHC[*n*]

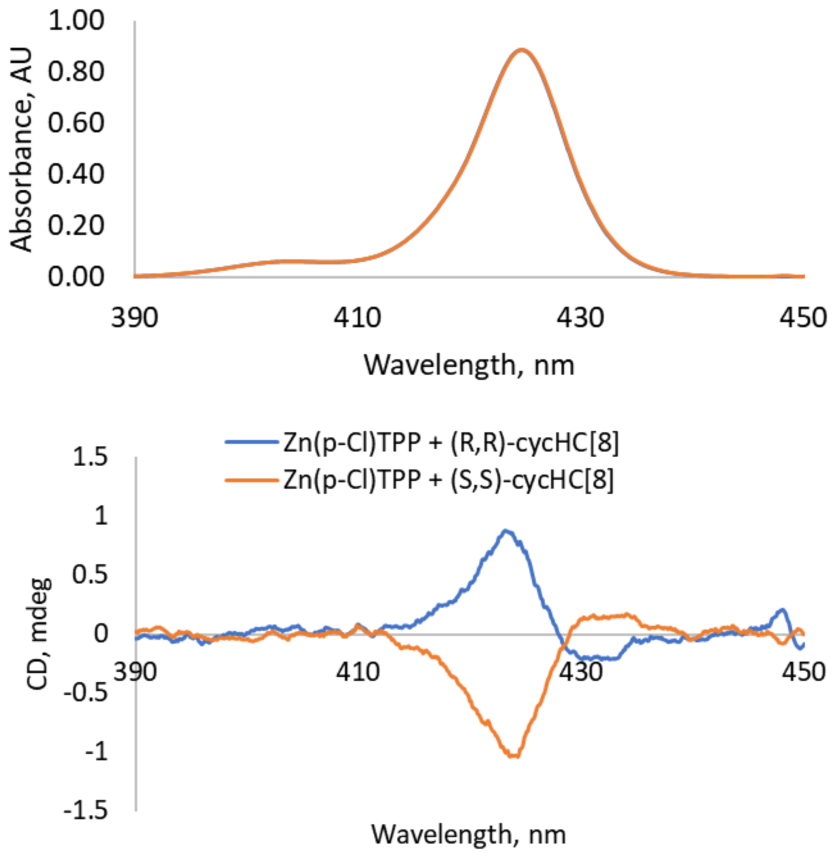

Figure S68 Absorbance and CD signal of 1.42 × 10^{-6} M **4** complex with **(*R,R*)-cycHC[8]** (1999 eq.) (blue) and with **(*S,S*)-cycHC[8]** (orange) (2667 eq.) in DCM.

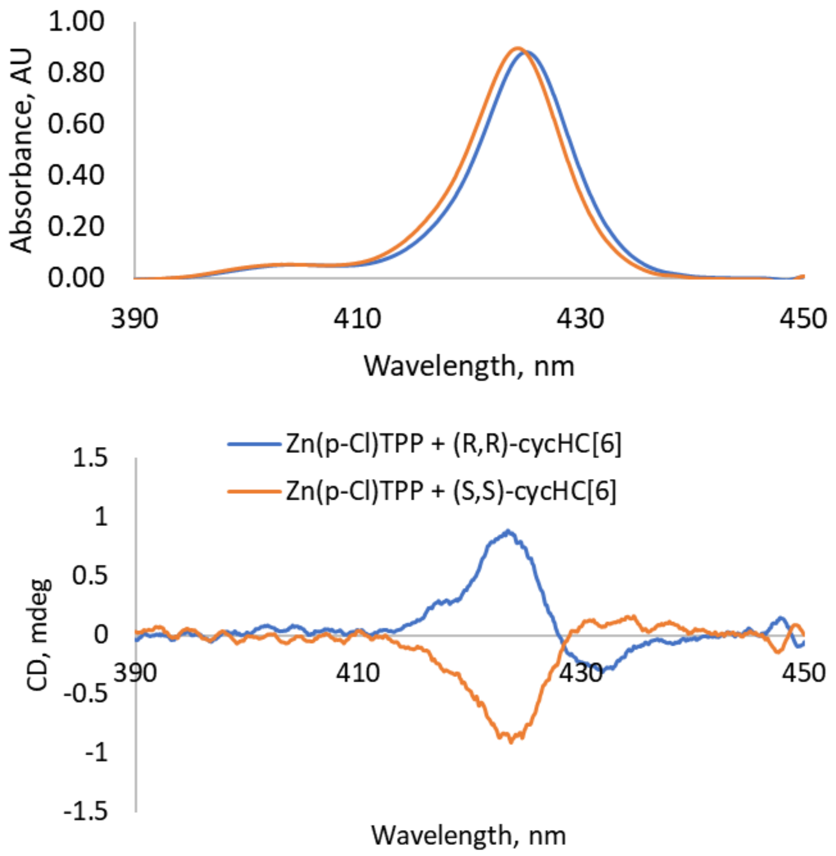

Figure S69 Absorbance and CD signal of 1.42 × 10^{-6} M **4** complex with **(*R,R*)-cycHC[6]** (2090 eq.) (blue) and with **(*S,S*)-cycHC[6]** (orange) (2620 eq.) in DCM.

UV-Vis and CD of Zn(*p*-CF_3)TPP (5) complexes with cycHC[*n*]

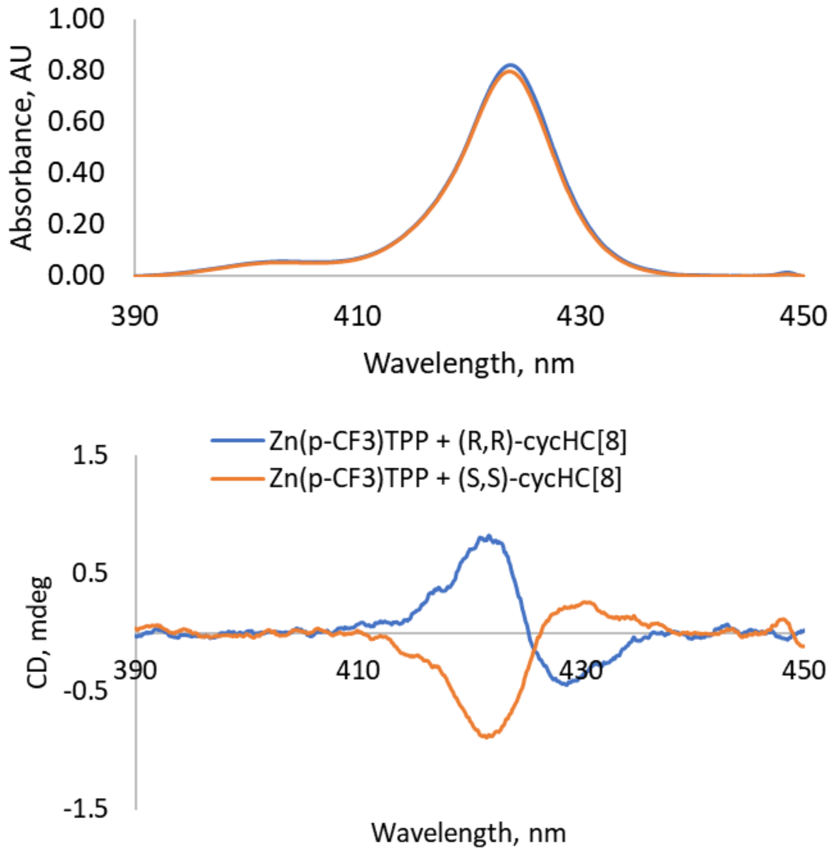

Figure S70 Absorbance and CD signal of 1.30 × 10^{-6} M **5** complex with **(*R,R*)-cycHC[8]** (2769 eq.) (blue) and with **(*S,S*)-cycHC[8]** (orange) (2738 eq.) in DCM.

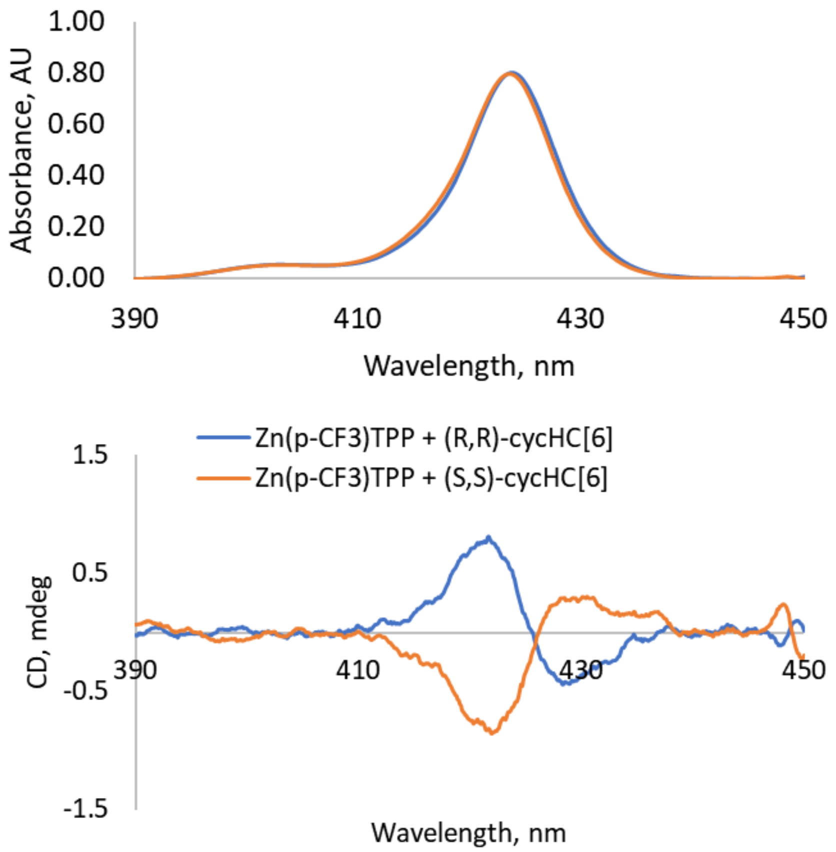

Figure S71 Absorbance and CD signal of 1.33 × 10^{-6} M **5** complex with ***(R,R)*-cycHC[6]** (2628 eq.) (blue) and with ***(S,S)*-cycHC[6]** (orange) (2751 eq.) in DCM.

UV-Vis and CD of ZnTPFPOBP (6) complexes with cycHC[*n*]

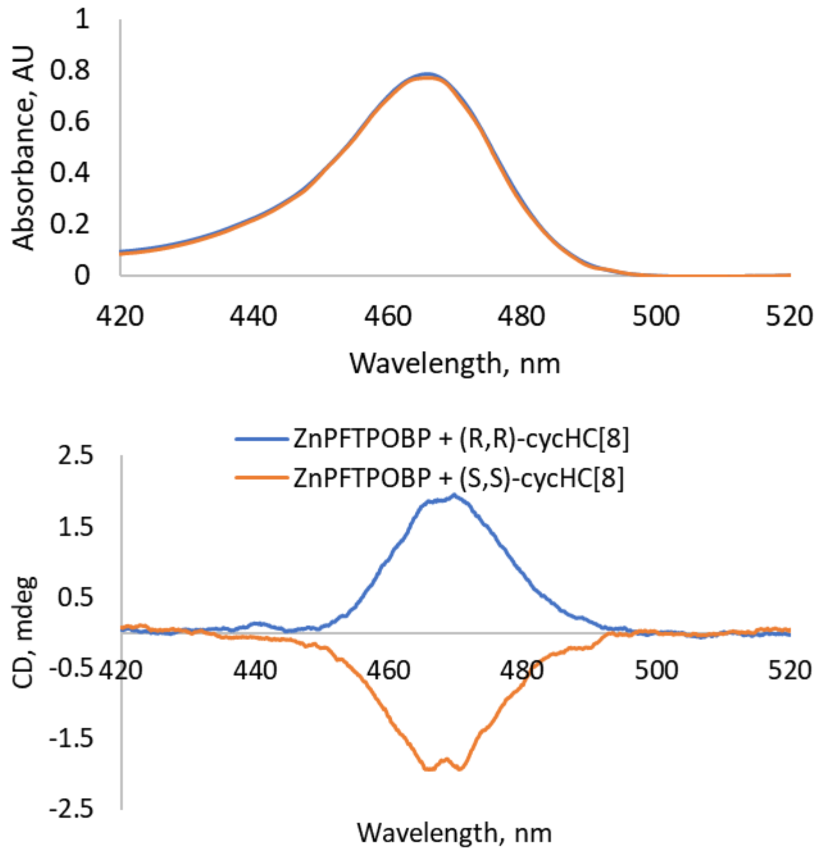

Figure S72 Absorbance and CD signal of 3.47 × 10^{-6} M **6** complex with **(*R,R*)-cycHC[8]** (15.45 eq.) (blue) and with **(*S,S*)-cycHC[8]** (orange) (17.31 eq.) in DCM.

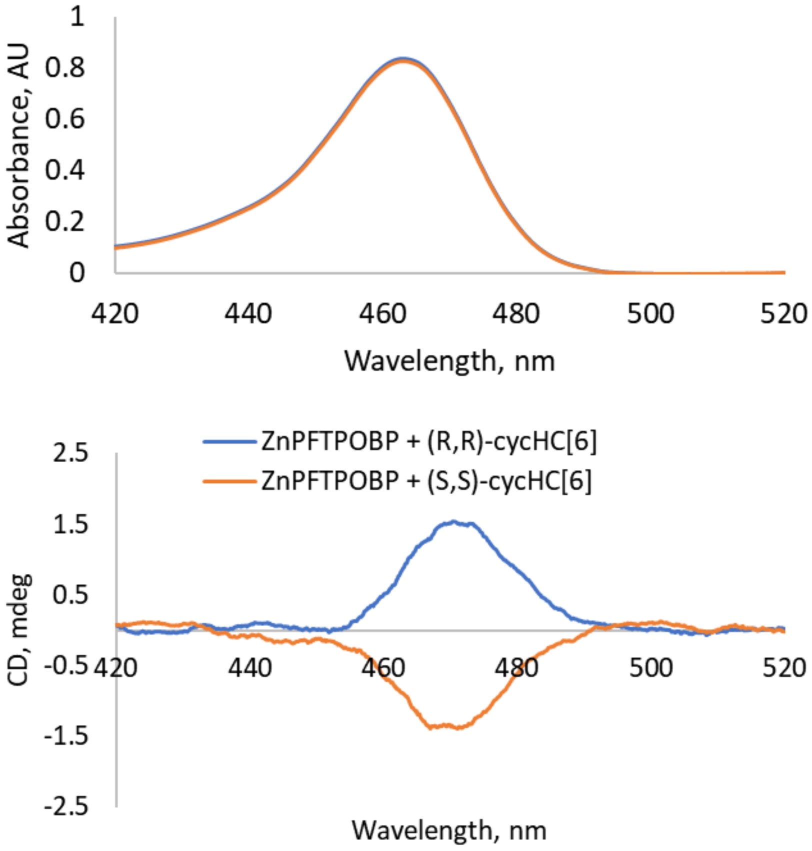

Figure S73 Absorbance and CD signal of 3.47 × 10^{-6} M **6** complex with **(*R,R*)-cycHC[6]** (18.24 eq.) (blue) and with **(*S,S*)-cycHC[6]** (orange) (18.03 eq.) in DCM.

UV-Vis and CD of MgTPP (7) complexes with cycHC[*n*]

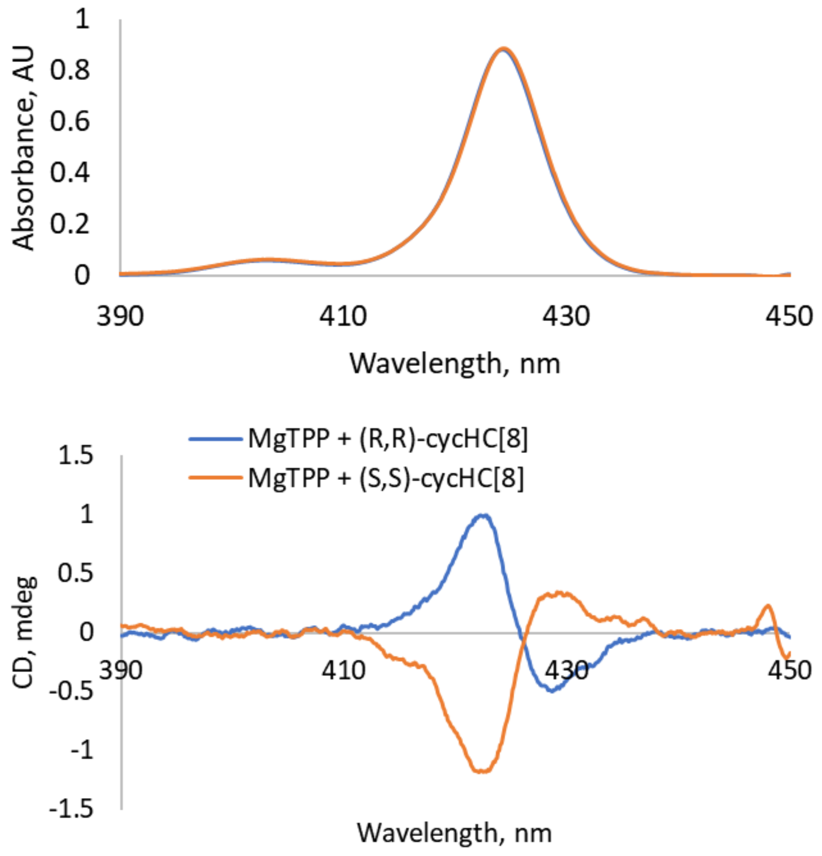

Figure S74 Absorbance and CD signal of 1.39 × 10^{-6} M **7** complex with **(*R,R*)-cycHC[8]** (blue) (263 eq.) and with **(*S,S*)-cycHC[8]** (orange) (256 eq.) in DCM.

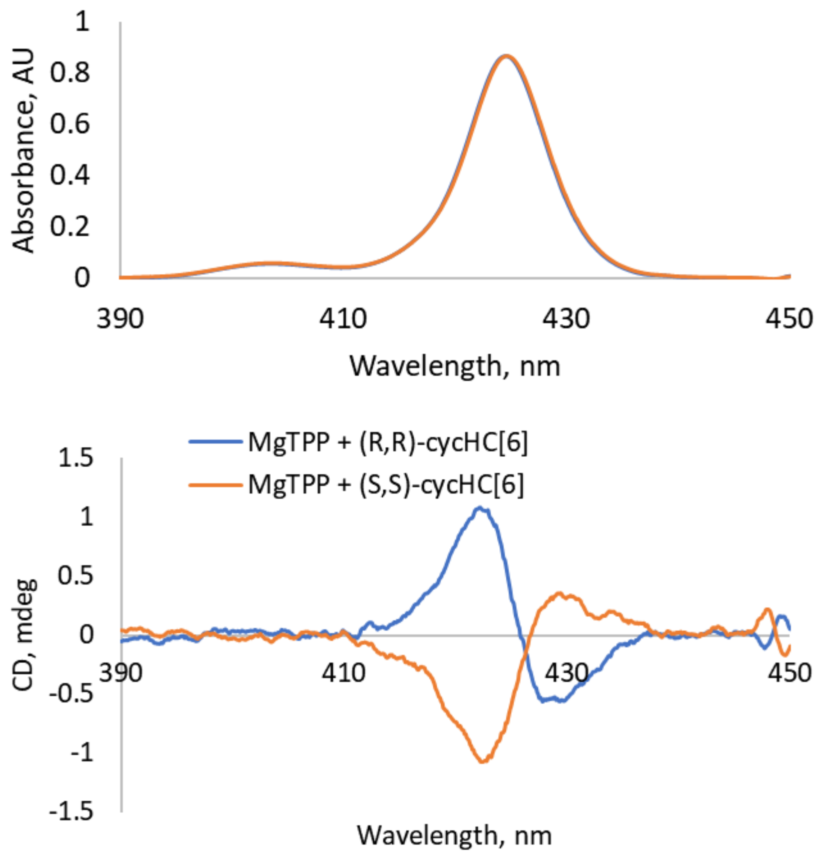

Figure S75 Absorbance and CD signal of 1.39 × 10^{-6} M **7** complex with **(*R,R*)-cycHC[6]** (blue) (295 eq.) and with **(*S,S*)-cycHC[6]** (orange) (282 eq.) in DCM.

4. Chiroptical properties in solid-state

4.1 ECD spectra

Solid-state samples were prepared by balancing 3-4 mg of porphyrin and equimolar amount of cycHC[*n*], compounds were mixed and dissolved in 1:2 MeOH/DCM mixture (v:v). The solutions were either set for crystallization (in case of **Figure S76**, **Figure S77**, **Figure S79** and **Figure S80**) or solvents evaporated rotary evaporator (**Figure S78**). The crystals or solid precipitate were mixed with 100 mg of KBr and pressed into a pellet. ECD, LD UV-Vis, LB and CB spectra of the pellets were recorded by solid-state dedicated circular dichroism (CD) spectrophotometer (J-800KCM). Similarly to solution experiments, g-factors were calculated and are summarized in **Table S4**.

Table S4 The UV-vis λ_{max}, ECD λ_{max} and *g*-factors at ECD λ_{max} values for porphyrin-cycHC[*n*] 1:1 complexes in solid-state. (+ and – Cotton effects are assigned for complexes with (*R*,*R*)-cycHC[*n*] while *g*-factor is average of porphyrin complexes with both enantiomers os cycHC[*n*]

No	Complex	UV-vis λ_{max}, nm	ECD λ_{max} of complex, nm	|*g*|-factor, 10^{-5}
1	**2**·cycHC[8] crystals	429	425 (+)/437 (-)	15.5/26.0
2	**2**·cycHC[8] percipitate	429	425 (+)/437 (-)	7.6/15.9
3	**7**·cycHC[8] crystals	430	423 (+)/438 (-)	10.4/26.4

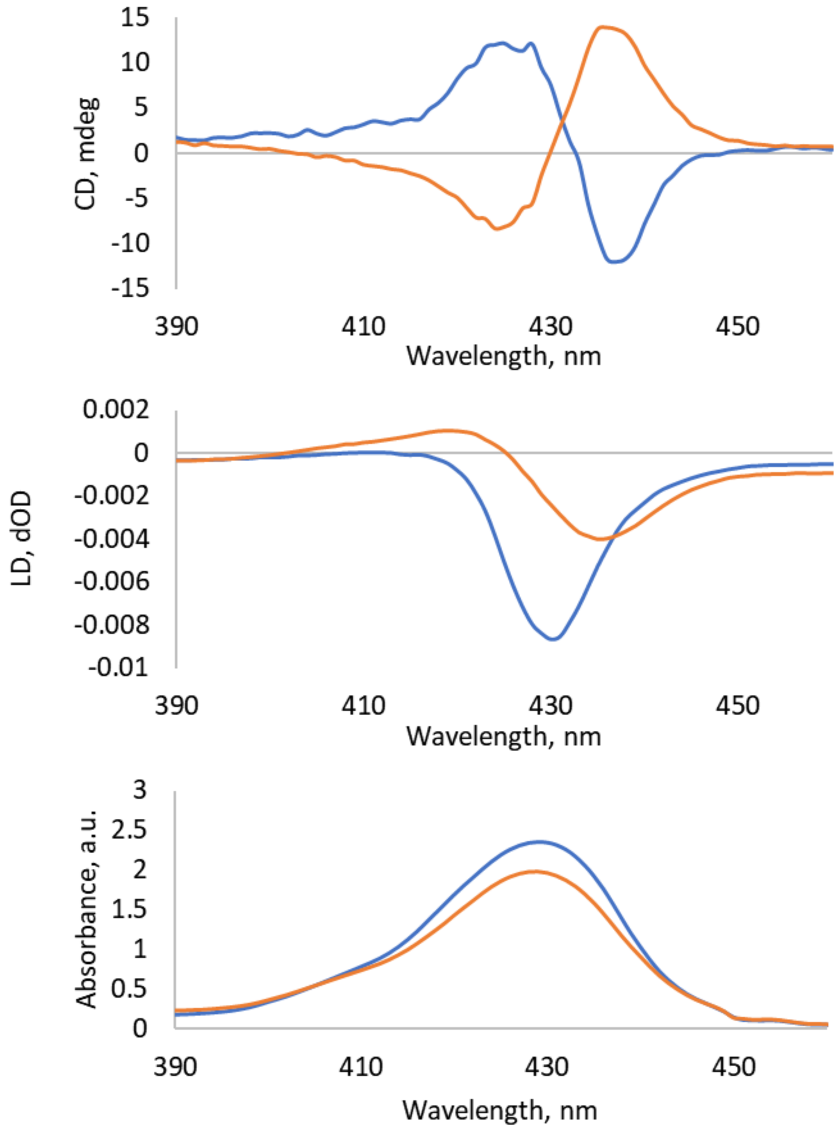

Figure S76 CD, linear dichroism (LD) and absorbance measurements of pellets made from crushed crystals of (*R,R*)-cycHC[8]·**2** (blue) and (*S,S*)-cycHC[8]·**2** (orange) in KBr matrix.

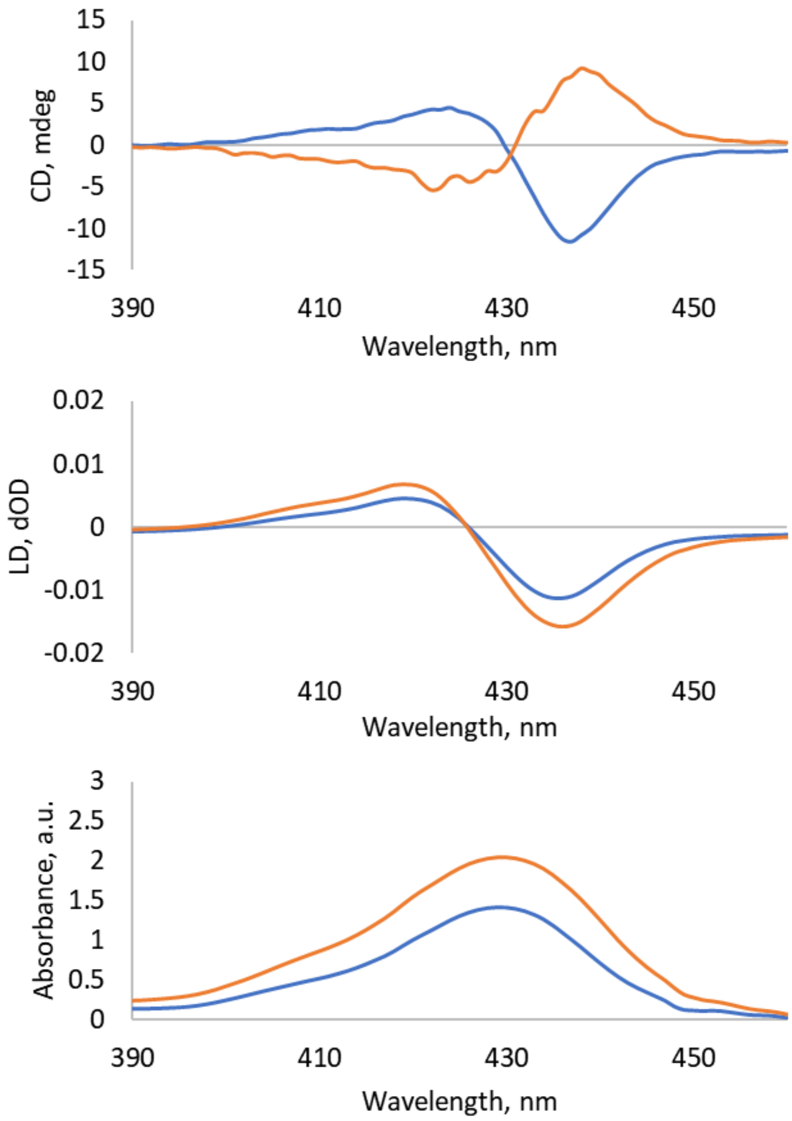

Figure S77 CD, LD and absorbance measurements of pellets made from crushed crystals of (*R,R*)-cycHC[8]·**7** (blue) and (*S,S*)-cycHC[8]·**7** (orange) in KBr matrix.

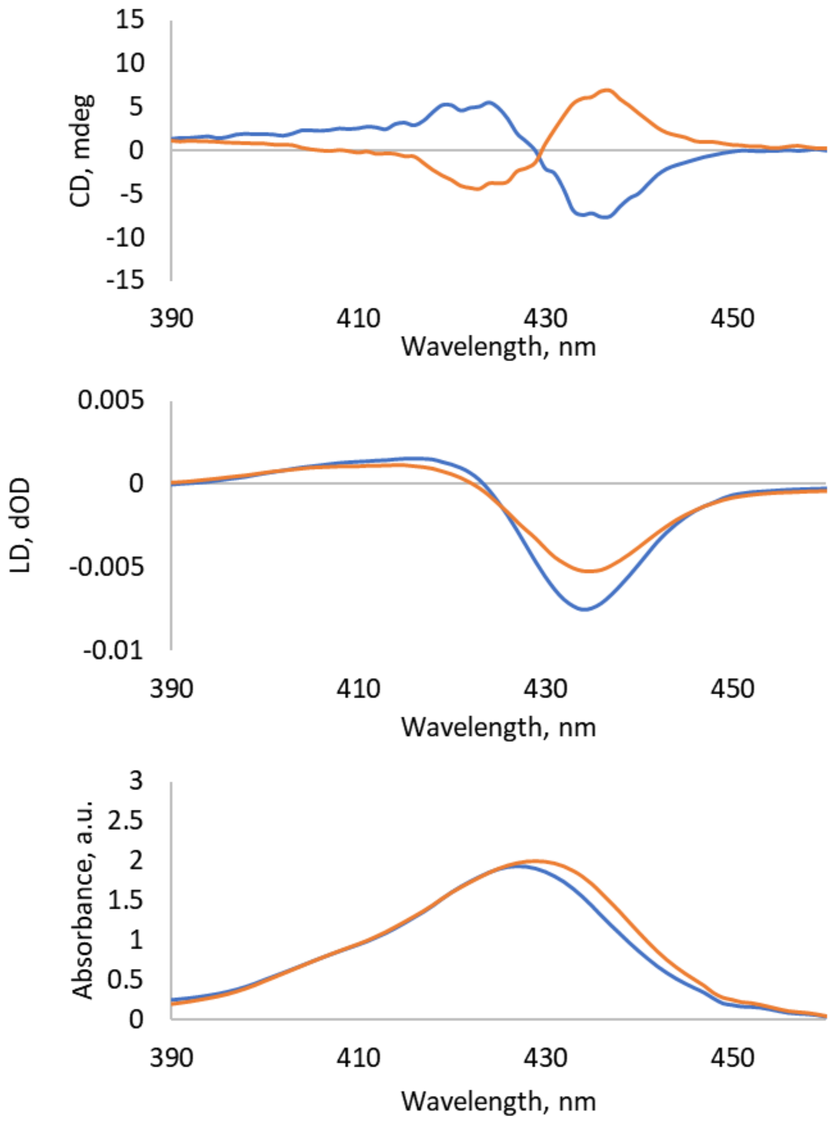

Figure S78 CD, linear dichroism (LD) and absorbance measurements of pellets made from precipitated solid containing mixture of (*R,R*)-cycHC[8]·**2** (blue) and (*S,S*)-cycHC[8]·**2** (orange) in KBr matrix.

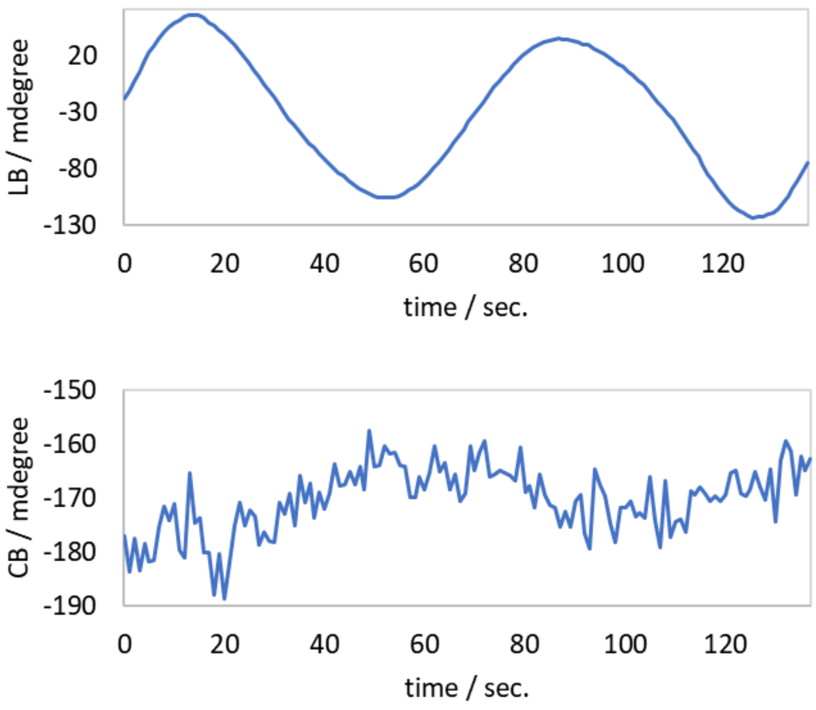

Figure S79 Linear birefringence (top) and circular birefringence (bottom) measurements of pellet made from crushed crystals of (*R,R*)-cycHC[8]·**2**.

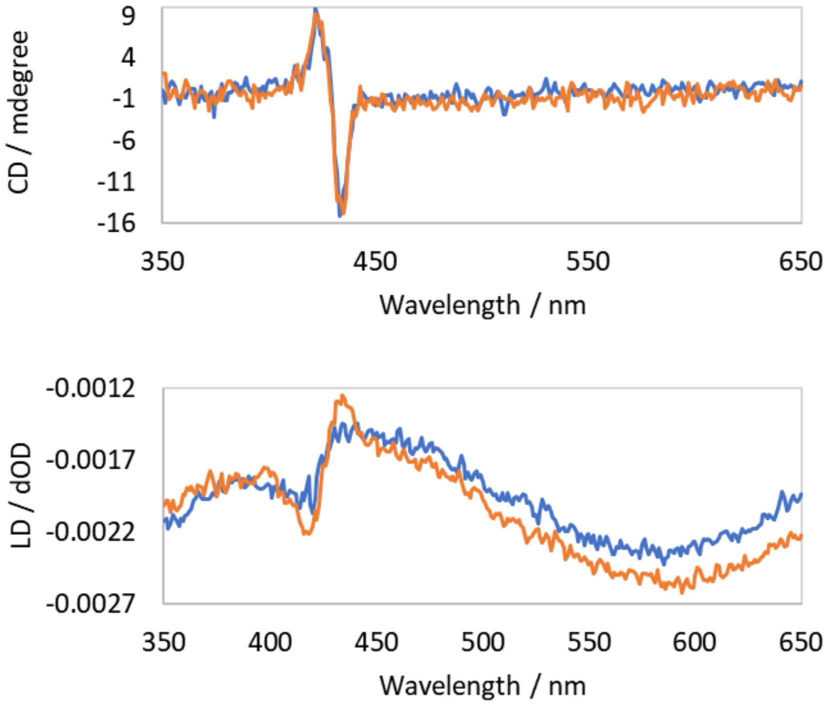

Figure S80 CD and LD measurements of pellet made from crushed crystals of (*R,R*)-cycHC[8]·**2** from front side at LB=0 (blue) and from back side at LB=0 (orange).).

4.2 Vibrational circular dichroism (VCD)

Concentration for IR and VCD spectra of complexes were from 0.7-0.8% and for pure compounds 0.4% in 1800-1300 cm^{-1} region. For 1200-700 cm^{-1} region, the concentration of complexes used was 1.9-2.4%. The *g*-factor was calculated as the average of the absolute values of the positive and negative signals.

Table S5 The characteristics of IR (range 2000-700 cm^{-1}) and VCD signals of free and complexed (*R,R*)-cycHC[*n*] with porphyrins **2** and **7**, measured in KBr pellets. The underlined bold values correspond to maxima of carbonyl signals in VCD and their g-factors. Region 1250-1200 cm^{-1} is omitted due to chiral water.

No	System	IR, cm^{-1}	VCD, cm^{-1} (sign of Cotton effect)	|*g*|-factor at maximum carbonyl VCD signals, ·10^{-5}
1	cycHC[6]	1708; 1459; 1431; 1413; 1380; 1358; 1332; 1311; 1252; 1192; 1141; 1115; 1088; 1059; 1025; 1009; 986; 920; 877; 826; 773	**1720** **(+)**; **1706** **(-)**; 1468 (-); 1458 (+); 1445 (+); 1432 (-); 1413 (+); 1385 (+); 1375 (-); 1357 (-); 1345 (-); 1328 (-); 1307 (+); 1299 (-); 1265 (+); 1187 (+); 1143 (-); 1125 (+); 1113 (-); 1060 (-); 1027 (+); 1008 (+); 985 (+); 843 (-); 833 (-); 825 (-)	**10**; **4**
2	cycHC[6]·**2**	1711; 1686; 1663; 1596; 1523; 1485; 1458; 1431; 1414; 1379; 1358; 1335; 1311; 1251; 1191; 1141; 1112; 1071; 1061; 1025; 1003; 993; 920; 877; 826; 795; 775; 752; 718; 702	**1722** **(+)**; **1704** **(+)**; **1679** **(-)**; **1657** **(-)**; 1597 (+); 1485 (+); 1469 (-); 1429 (-); 1413 (+); 1392 (-); 1375 (-); 1358 (-); 1330 (-); 1312 (-); 1273 (-); 1184 (+); 1172 (+); 1142 (-); 1125 (+); 1111 (-); 1023 (+); 1003 (+); 990 (+); 828 (-); 800 (-)	**50**; **100**; **50**; **20**
3	cycHC[6]·**7**	1711; 1669; 1597; 1522; 1486; 1461; 1438; 1359; 1333; 1310; 1260; 1250; 1137; 1123; 1069; 1059; 1013; 1004; 993; 919; 883; 830; 797; 772; 754; 719; 702	**1704** **(+)**; **1685** **(+)**; **1677** **(-)**; 1458 (+); 1445 (+); 1432 (-); 1415 (+); 1384 (+); 1374 (-); 1368 (+); 1360 (-); 1343 (-); 1329 (-); 1265 (+); 1186 (+); 1155 (+); 1123 (+); 1027 (+); 1008 (+); 988 (+); 966 (+); 929 (-); 916 (-); 895 (+); 874 (+); 826 (-)	**20**; **10**; **6**
4	cycHC[8]	1709; 1462; 1439; 1419; 1359; 1331; 1310; 1260; 1187; 1135; 1124; 1085; 1058; 1015; 987; 919; 883; 831; 773	**1716** **(+)**; **1699** **(-)**; 1460 (+); 1447 (+); 1414 (+); 1393 (+); 1385 (+); 1368 (-); 1347 (-); 1332 (-); 1326 (-); 1307 (+); 1264 (+); 1185 (+); 1141 (-); 1129 (+); 1114 (-); 1079 (+); 1067 (-); 1055 (+); 1013 (+); 988 (+); 934 (-); 920 (+); 896 (+); 831 (-)	**8**; **3**
5	cycHC[8]·**2**	1711; 1669; 1597; 1524; 1484; 1460; 1437; 1359; 1333; 1310; 1259; 1250; 1136; 1124; 1070; 1059; 1012; 1003; 993; 917; 883; 830; 797; 773; 754; 719; 702	**1723** **(-)**; **678** **(+)**; 1460 (+); 1446 (+); 1414 (+); 1394 (+); 1384 (+); 1371 (-); 1360 (+); 1346 (-); 1329 (-); 1308 (+); 1264 (+); 1197 (-); 1140 (-); 1130 (+); 1111 (-); 1068	**20**; **30**

			(-); 1013 (+); 922 (+); 894 (+); 830 (-)	
6	cycHC[8]·**7**	1711; 1598; 1517; 1479; 1460; 1437; 1419; 1360; 1332; 1310; 1259; 1136; 1124; 1070; 1060; 1009; 994; 918; 882; 830; 798; 772; 764; 755; 721; 702	**<u>1715</u> (+)**; 1459 (+); 1447 (+); 1415 (+); 1382 (+); 1371 (-); 1332 (-); 1308 (+); 1263 (+); 1194 (-); 1142 (-); 1114 (-); 1012 (+); 989 (+); 832 (-)	**<u>8</u>**

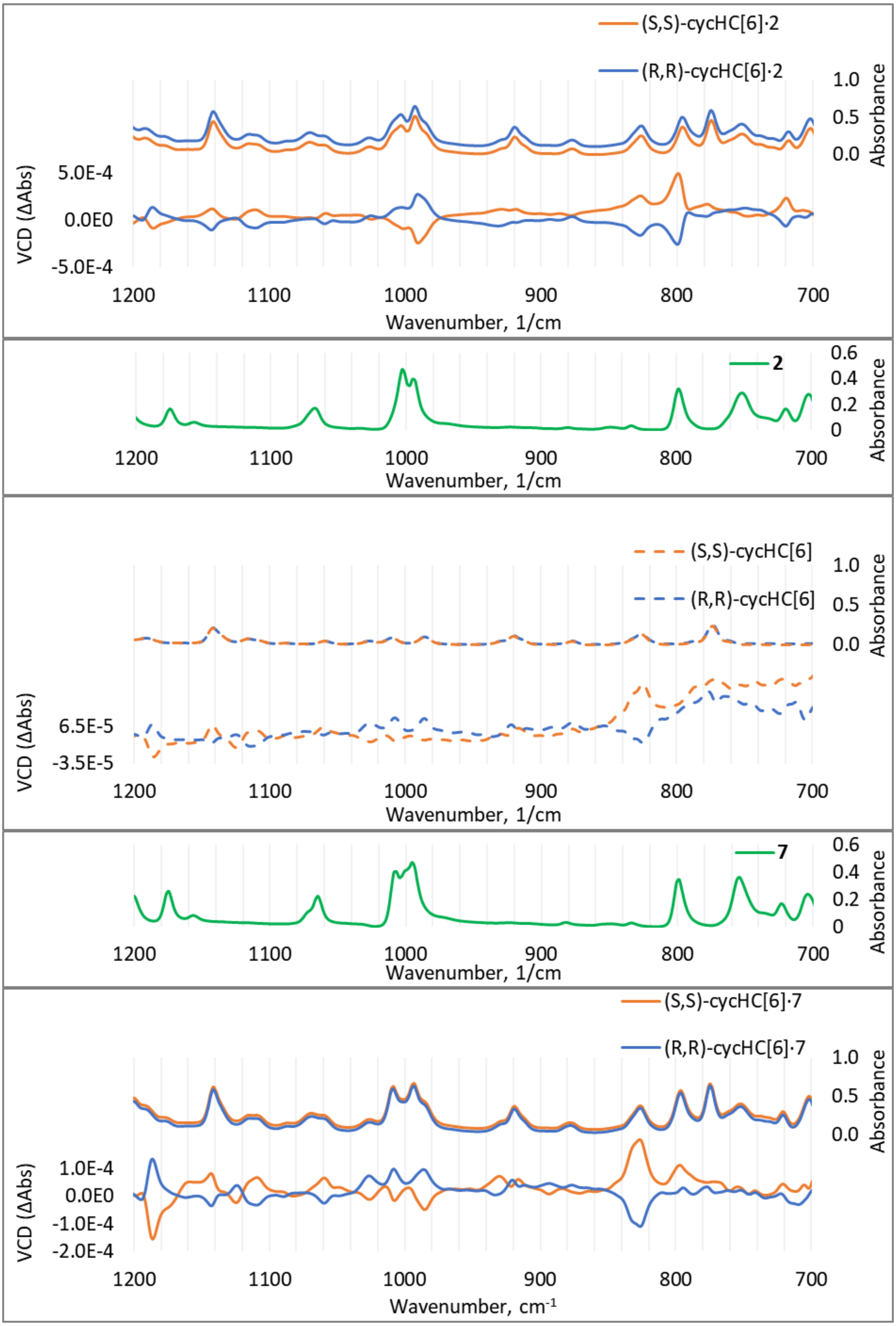

Figure S81 Measured IR of free porphyrins, IR and VCD spectra of cycHC[6] and its complexes with porphyrins in KBr in the range of 1200-700 cm^{-1}.

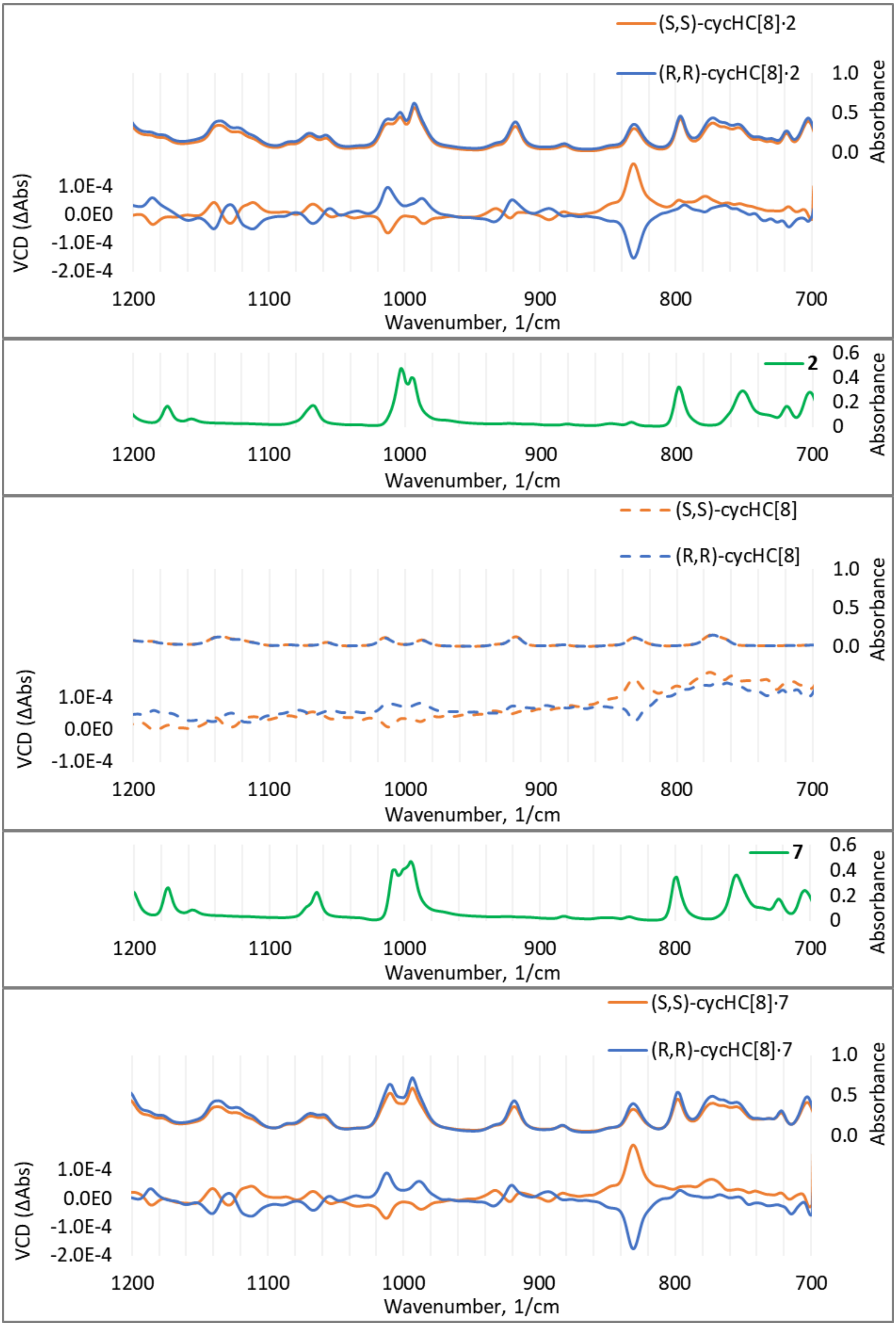

Figure S82 Measured IR of free porphyrins, IR and VCD spectra of cycHC[8] and its complexes with porphyrins in KBr in the range of 1200-700 cm^{-1}.

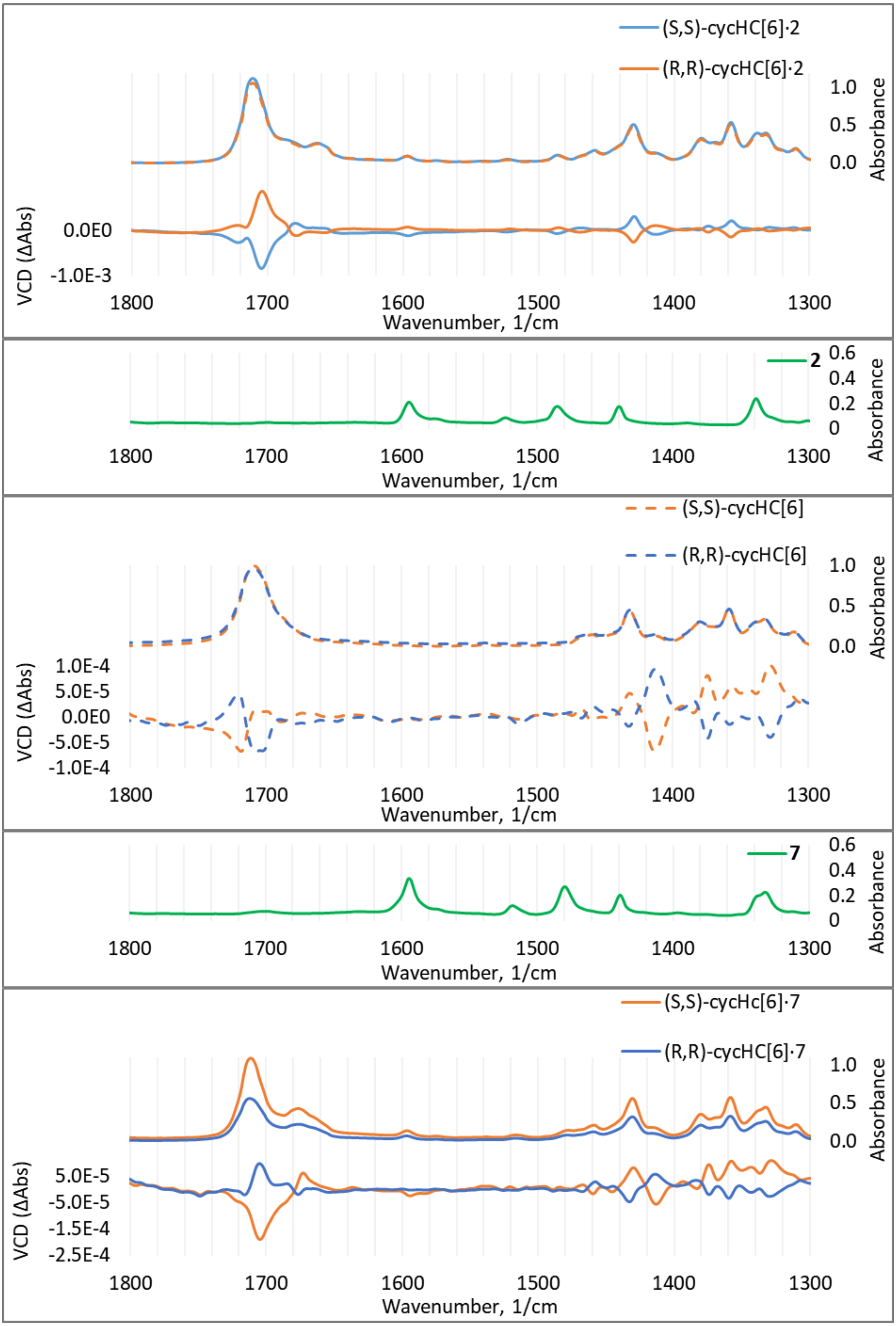

Figure S83 Measured IR of free porphyrins, IR and VCD spectra of cycHC[6] and its complexes porphyrins in KBr in the range of 1800-1300 cm^{-1}.

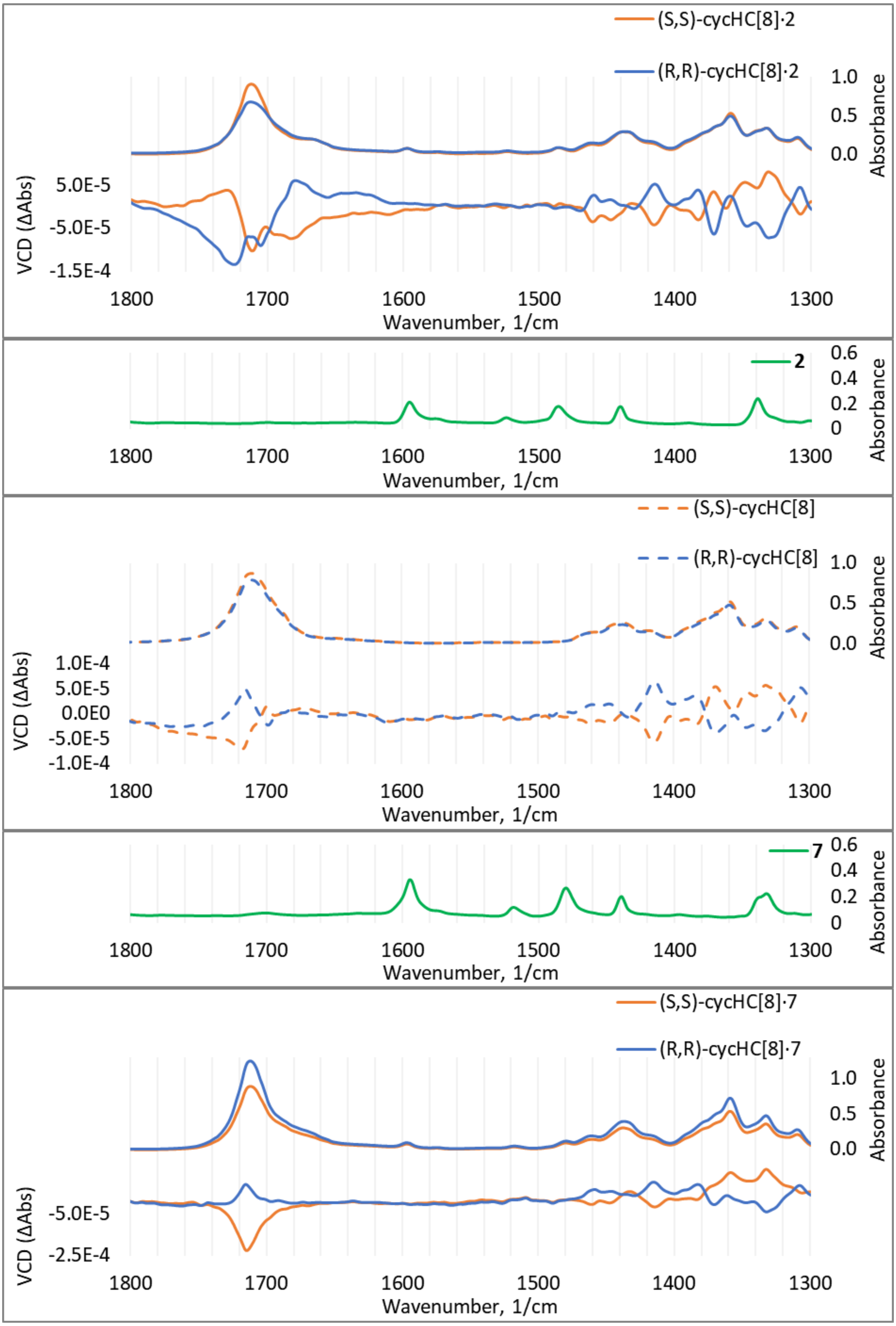

Figure S84 Measured IR of free porphyrins, IR and VCD spectra of cycHC[8] and its complexes with porphyrins in KBr in the range of 1800-1300 cm^{-1}.

5. X-ray Crystallographic Studies

The experimental and refinement details for all solid-state structures are given below. Single-crystal X-ray data of (*R,R*)-cycHC[6] · **1**, (*S,S*)-cycHC[6] · **1**, (*S,S*)-cycHC[6] · **2(a)**, (*S,S*)-cycHC[6] · **2(b)**, (*R,R*)-cycHC[6] · **3**, (*S,S*)-cycHC[6] · **3**, (*S,S*)-cycHC[6] · **4**, (*R,R*)-cycHC[6] · **5**, (*S,S*)-cycHC[6] · **5**, (*R,R*)-cycHC[6] · **6**, (*R,R*)-cycHC[6] · **7**, (*S,S*)-cycHC[6] · **7**, (*S,S*)-cycHC[8] · **1(a)**, (*S,S*)-cycHC[8] · **1(b)**, (*R,R*)-cycHC[8] · **2(a)**, (*R,R*)-cycHC[8] · **2(c)**, (*R,R*)-cycHC[8] · **2(d)**, (*R,R*)-cycHC[8] · **2(e)**, (*S,S*)-cycHC[8] · **2(a)**, (*S,S*)-cycHC[8] · **2(b)**, (*R,R*)-cycHC[8] · **3(a)**, (*R,R*)-cycHC[8] · **3(b)**, (*S,S*)-cycHC[8] · **3**, (*R,R*)-cycHC[8] · **4(a)**, (*R,R*)-cycHC[8] · **4(b)**, (*S,S*)-cycHC[8] · **4**, (*R,R*)-cycHC[8] · **5**, (*S,S*)-cycHC[8] · **5**, (*R,R*)-cycHC[8] · **7** and (*S,S*)-cycHC[8] · **7** were measured using a dual-source Rigaku SuperNova diffractometer equipped with an Atlas detector and an Oxford Cryostream cooling system using mirror-monochromated Cu-K_α radiation (λ = 1.54184 Å). Data collection and reduction for all compounds were performed using the program *CrysAlisPro*[6] and Gaussian face-index absorption correction method was applied.[6] X-ray data for (*R,R*)-cycHC[6] · **4** was measured using a Bruker-Nonius KappaCCD diffractometer with an APEX-II detector with graphite-monochromatized Mo-K_α (λ = 0.71073 Å) radiation. Data collection and reduction were performed using the program *COLLECT*[7] and *HKL DENZO AND SCALEPACK*,[8] respectively, and the intensities were corrected for absorption using *SADABS*.[9] The structures were solved with Direct Methods (*SHELXS*) and refined by full-matrix least-squares based on F^2 using *SHELXL*-2015.[10–12] Non-hydrogen atoms were assigned anisotropic displacement parameters unless stated otherwise. The hydrogen atoms bonded to oxygens were located from Fourier difference maps and refined with an O−H distance restraint of approximately 0.92 Å. Other hydrogen atoms were placed in idealized positions and included as riding. Isotropic displacement parameters for all H atoms were constrained to multiples of the equivalent displacement parameters of their parent atoms with $U_{iso}(H)$ = 1.2 $U_{eq}(CH, CH_2)$ or 1.5 $U_{eq}(CH_3, OH)$. A few reflections with large discrepancies between the calculated and observed structure factors have been omitted from the least-squares refinement as outliers. Enhanced rigid bond restraints[13,14] with non-standard uncertainties of 0.001 Å^2 were applied for several atom pairs as well as distance restraints (DFIX). Due to the size of asymmetric units, BLOC commands were used for anisotropic refinement and the model required SIMU restraints to adequately model the thermal parameters of the structure. The X-ray single crystal data and experimental details as well as CCDC numbers (CCDC 2444098 - 2444131) are given below.

Table S6 The crystallographic data for all new structures reported herein.

**Complex**	**(*R*,*R*)-cycHC[6]·1**	**(*S*,*S*)-cycHC[6]·1**	**(*S*,*S*)-cycHC[6]·2(a)**	**(*S*,*S*)-cycHC[6]·2(b)**
Figure	**Figure S85**	**Figure S86**	**Figure S87**	**Figure S88**
Formula	$C_{409}H_{554}Cl_2N_{72}O_{24}Zn_6$	$C_{244.5}H_{329}Cl_9N_{40}O_{12}Zn_4$	$C_{92}H_{100}N_{16}O_6Zn$	$C_{141}H_{174}Cl_2N_{28}O_{12}Zn$
Formula Weight (gmol^{-1})	7326.33	4600.99	1591.24	2589.34
Colour & Habit	Purple plate	Red block	Purple plate	Purple needle
Crystal Dimensions (mm)	0.05 × 0.12 × 0.14	0.25 × 0.26 × 0.28	0.07 × 0.13 × 0.37	0.02 × 0.04 × 0.30
Crystal System	Monoclinic	Triclinic	Orthorhombic	Monoclinic
Space Group	$P2_1$	$P1$	$C222_1$	$P2_1$
a (Å)	26.3928(2)	13.5387(5)	28.9308(7)	12.3448(6)
b (Å)	23.0687(2)	19.9529(6)	29.1900(8)	48.1538(19)
c (Å)	32.7554(2)	25.3189(6)	23.1944(5)	12.4691(5)
α (°)	90	68.9340(10)	90	90
β (°)	96.9300(10)	82.797(2)	90	90.233(2)
γ (°)	90	74.217(2)	90	90
V (Å)	19797.3(3)	6138.9(3)	19587.4(8)	7412.2(6)
Z	2	1	8	2
ρ_{calc} (gcm^{-3})	1.229	1.245	1.079	1.160
F(000)	7836	2445	6736	2752
μ (mm^{-1})	1.082	1.858	0.785	1.081
Temperature (K)	120.0(1)	120.0(1)	120.0(1)	120.0(1)
ϑ_{max} [°]	76.2	70.0	70.0	70.0
Total Reflections	153591	113384	97331	62005
Independent Reflections	78383	44441	18567	25046
Reflections ($I_o > 2\sigma[I_o]$)	65520	22202	11716	17727
R_{int}	0.0526	0.0595	0.0762	0.0999
Parameters	4666	2738	966	1657
Restraints	73	309	51	10
GooF (F^2)	1.012	1.021	1.039	1.040
R_1 ($I_o > 2\sigma[I_o]$)	0.0455	0.0868	0.0806	0.0741
R_1 (all reflections)	0.0600	0.1394	0.1115	0.1062
wR_2 ($I_o > 2\sigma[I_o]$)	0.0986	0.1970	0.1982	0.1549
wR_2 (all reflections)	0.1074	0.2193	0.2123	0.1715
Largest Peak (eÅ^{-3})	0.659	1.386	0.761	0.438
Largest Hole (eÅ^{-3})	-0.295	-1.331	-0.498	-0.533
CCDC Number	2444113	2444098	2444107	2444108

Continuation of **Table S6**

**Complex**	**(*R*,*R*)-cycHC[6]·3**	**(*S*,*S*)-cycHC[6]·3**	***(R,R)*-cycHC[6]·4**	**(*S*,*S*)-cycHC[6]·4**
Figure	**Figure S89**	**Figure S90**	**Figure S91**	**Figure S92**
Formula	$C_{369}H_{388}F_{16}N_{64}O_{25}Zn_4$	$C_{188}H_{206}Cl_2F_8N_{32}O_{15}Zn_2$	$C_{186}H_{196}Cl_{12}N_{32}O_{12}Zn_2$	$C_{186}H_{196}Cl_{12}N_{32}O_{12}Zn_2$
Formula Weight (gmol^{-1})	6684.89	3507.48	3627.88	3627.88
Colour & Habit	Purple plate	Purple plate	Purple plate	Purple plate
Crystal Dimensions (mm)	0.02 × 0.08 × 0.19	0.04 × 0.10 × 0.17	0.03 × 0.19 × 0.21	0.10 × 0.22 × 0.22
Crystal System	Monoclinic	Monoclinic	Triclinic	Triclinic
Space Group	$P2_1$	$P2_1$	$P1$	$P1$
a (Å)	23.4044(16)	23.2961(6)	14.3081(4)	14.2130(2)
b (Å)	29.1054(18)	15.4998(3)	15.8178(5)	15.7401(2)
c (Å)	25.121(2)	29.6748(7)	23.1376(8)	23.0071(4)
α (°)	90	90	91.125(2)	91.0790(10)
β (°)	102.186(7)	109.200(2)	105.3220(10)	105.2780(10)
γ (°)	90	90	112.634(2)	112.869(2)
V (Å)	16727(2)	10119.1(4)	4618.0(3)	4532.07(13)
Z	2	2	1	1
ρ_{calc} (gcm^{-3})	1.327	1.151	1.305	1.329
F(000)	7028	3688	1896	1896
μ (mm^{-1})	1.019	1.110	0.501	2.504
Temperature (K)	120.0(1)	120.0(1)	120.0(1)	120.0(1)
ϑ_{max} [°]	78.5	70.0	25.4	70.0
Total Reflections	128990	107427	33264	100843
Independent Reflections	67593	36260	23552	33097
Reflections ($I_o > 2\sigma[I_o]$)	25568	28190	15660	32687
R_{int}	0.1531	0.0708	0.0522	0.0262
Parameters	4282	2192	2198	2197
Restraints	385	19	120	6
GooF (F^2)	1.081	1.047	1.079	1.052
R_1 ($I_o > 2\sigma[I_o]$)	0.1679	0.0512	0.0770	0.0316
R_1 (all reflections)	0.2614	0.0723	0.1286	0.0321
wR_2 ($I_o > 2\sigma[I_o]$)	0.3895	0.1139	0.1668	0.0854
wR_2 (all reflections)	0.4772	0.1238	0.1954	0.0859
Largest Peak (eÅ^{-3})	1.265	0.436	0.354	0.458
Largest Hole (eÅ^{-3})	-1.562	-0.445	-0.466	-0.676
CCDC Number	2444114	2444101	2444115	2444102

Continuation of **Table S6**

**Complex**	**(*R*,*R*)-cycHC[6]·5**	**(*S*,*S*)-cycHC[6]·5**	**(*R*,*R*)-cycHC[6]·6**	**(*R*,*R*)-cycHC[6]·7**
Figure	**Figure S93**	**Figure S94**	**Figure S95**	**Figure S96**
Formula	$C_{192}H_{192}F_{24}N_{32}O_{12}Zn_2$	$C_{192}H_{192}F_{24}N_{32}O_{12}Zn_2$	$C_{185}H_{148}Br_{16}F_{40}N_{32}O_{13}Zn_2$	$C_{92}H_{100}MgN_{16}O_6$
Formula Weight (gmol^{-1})	3726.50	3726.50	5196.65	1550.18
Colour & Habit	Purple plate	Purple plate	Green plate	Purple block
Crystal Dimensions (mm)	0.03 × 0.10 × 0.16	0.09 × 0.24 × 0.27	0.01 × 0.04 × 0.08	0.04 × 0.08 × 0.15
Crystal System	Triclinic	Triclinic	Triclinic	Orthorhombic
Space Group	*P*1	*P*1	*P*1	$C222_1$
a (Å)	19.8043(4)	19.9874(3)	13.9211(3)	29.1542(3)
b (Å)	23.1780(6)	23.4110(4)	16.6156(4)	29.3525(3)
c (Å)	23.8651(6)	24.0257(4)	23.7795(7)	23.2437(3)
α (°)	103.744(2)	103.375(2)	83.376(2)	90
β (°)	97.4830(10)	98.2680(10)	77.191(2)	90
γ (°)	114.265(2)	114.929(2)	66.2890(10)	90
V (Å)	9374.2(4)	9540.1(3)	4908.6(2)	19890.8(4)
Z	2	2	1	8
ρ_{calc} (gcm^{-3})	1.320	1.297	1.758	1.035
F(000)	3880	3880	2566	6592
μ (mm^{-1})	1.090	1.071	5.043	0.585
Temperature (K)	120.0(1)	120.0(1)	120.0(1)	120.0(1)
ϑ_{max} [°]	70.0	70.0	70.0	76.4
Total Reflections	175113	167890	99680	195677
Independent Reflections	68278	68115	35681	20645
Reflections ($I_o > 2\sigma[I_o]$)	32233	53305	18007	16679
R_{int}	0.0817	0.0614	0.0600	0.0592
Parameters	2066	4717	1206	1032
Restraints	37	71	136	108
GooF (F^2)	1.094	1.019	1.024	1.049
R_1 ($I_o > 2\sigma[I_o]$)	0.1129	0.0686	0.0890	0.0600
R_1 (all reflections)	0.1719	0.0884	0.1358	0.0735
wR_2 ($I_o > 2\sigma[I_o]$)	0.2632	0.1697	0.2042	0.1682
wR_2 (all reflections)	0.2981	0.1840	0.2240	0.1815
Largest Peak (eÅ^{-3})	1.189	0.803	2.031	0.399
Largest Hole (eÅ^{-3})	-0.811	-0.457	-1.044	-0.409
CCDC Number	2444116	2444103	2444117	2444118

Continuation of **Table S6**

**Complex**	**(*S,S*)-cycHC[6]·7**	**(*S,S*)-cycHC[8]·1(a)**	**(*S,S*)-cycHC[8]·1(b)**	**(*R,R*)-cycHC[8]·2(a)**
Figure	**Figure S97**	**Figure S98**	**Figure S99**	**Figure S100**
Formula	$C_{185}H_{202}Cl_2Mg_2N_{32}O_{12}$	$C_{136}H_{184}N_{24}O_8Zn_2$	$C_{302}H_{393}Cl_5N_{48}O_{17.12}Zn_4$	$C_{156}H_{164}Cl_4N_{24}O_{10}Zn_2$
Formula Weight (gmol^{-1})	3185.29	2413.80	5408.27	2807.64
Colour & Habit	Blue plate	Pink plate	Pink plate	Purple needle
Crystal Dimensions (mm)	0.04 × 0.21 × 0.39	0.09 × 0.24 × 0.29	0.08 × 0.16 × 0.19	0.03 × 0.12 × 0.22
Crystal System	Orthorhombic	Triclinic	Monoclinic	Monoclinic
Space Group	$C222_1$	$P1$	$P2_1$	$P2_1$
a (Å)	28.8666(7)	14.4432(4)	15.2868(3)	17.746(2)
b (Å)	29.0639(8)	14.4555(5)	23.9951(6)	23.728(2)
c (Å)	23.1320(5)	17.2868(7)	20.3503(5)	18.111(2)
α (°)	90	79.906(3)	90	90
β (°)	90	76.087(2)	103.259(2)	95.469(10)
γ (°)	90	75.773(2)	90	90
V (Å)	19407.2(8)	3369.9(2)	7265.7(3)	7591.1(15)
Z	4	1	1	2
ρ_{calc} (gcm^{-3})	1.090	1.189	1.236	1.228
F(000)	6760	1292	2883	2952
μ (mm^{-1})	0.858	0.935	1.339	1.551
Temperature (K)	120.0(1)	120.0(1)	120.0(1)	120.0(1)
ϑ_{max} [°]	70.0	70.0	70.0	76.9
Total Reflections	76476	62119	55618	49063
Independent Reflections	17881	24511	27465	30068
Reflections ($I_o > 2\sigma[I_o]$)	9960	9182	20385	8871
R_{int}	0.0716	0.0608	0.0578	0.1571
Parameters	1130	1304	1829	1769
Restraints	135	223	115	120
GooF (F^2)	1.054	1.013	1.010	0.917
R_1 ($I_o > 2\sigma[I_o]$)	0.0767	0.1178	0.0657	0.1035
R_1 (all reflections)	0.1152	0.1681	0.0910	0.2197
wR_2 ($I_o > 2\sigma[I_o]$)	0.1733	0.2508	0.1616	0.1869
wR_2 (all reflections)	0.1851	0.2741	0.1768	0.2701
Largest Peak (eÅ^{-3})	0.736	1.375	0.933	0.692
Largest Hole (eÅ^{-3})	-0.337	-0.663	-0.451	-0.627
CCDC Number	2444104	2444105	2444106	2444120

Continuation of **Table S6**

**Complex**	**(*R*,*R*)-cycHC[8]·2(c)**	**(*R*,*R*)-cycHC[8]·2(d)**	**(*R*,*R*)-cycHC[8]·2(e)**	**(*S*,*S*)-cycHC[8]·2(a)**
Figure	**Figure S101**	**Figure S102**	**Figure S103**	**Figure S104**
Formula	$C_{472}H_{490}Cl_{30}N_{72}O_{25}Zn_6$	$C_{311}H_{320}Cl_{12}N_{48}O_{17}Zn_4$	$C_{438}H_{520}N_{80}O_{38}Zn_4$	$C_{154}H_{156}Cl_4N_{24}O_8Zn_2$
Formula Weight (gmol^{-1})	9027.05	5689.01	7774.79	2743.56
Colour & Habit	Purple plate	Purple plate	Pink plate	Purple plate
Crystal Dimensions (mm)	0.04 × 0.13 × 0.29	0.07 × 0.07 × 0.14	0.02 × 0.06 × 0.10	0.06 × 0.20 × 0.22
Crystal System	Triclinic	Triclinic	Triclinic	Triclinic
Space Group	*P*1	*P*1	*P*1	*P*1
a (Å)	17.6066(2)	17.0792(2)	18.0805(6)	17.6776(8)
b (Å)	18.0973(2)	17.5774(2)	25.0438(7)	18.0817(6)
c (Å)	36.1309(5)	26.1799(3)	25.0965(9)	24.0274(10)
α (°)	85.7850(10)	83.3030(10)	88.646(3)	88.837(3)
β (°)	88.0090(10)	73.3320(10)	71.001(3)	88.585(2)
γ (°)	89.0700(10)	83.8620(10)	87.975(2)	87.996(2)
V (Å)	11473.3(2)	7455.48(15)	10737.2(6)	7671.7(5)
Z	1	1	1	2
ρ_{calc} (gcm^{-3})	1.306	1.267	1.202	1.188
F(000)	4716	2982	4132	2880
μ (mm^{-1})	2.508	1.900	0.841	1.514
Temperature (K)	120.0(1)	120.0(1)	120.0(1)	120.0(1)
ϑ_{max} [°]	76.5	76.9	76.7	70.0
Total Reflections	215818	187435	200352	102550
Independent Reflections	88216	59202	81807	46279
Reflections ($I_o > 2\sigma[I_o]$)	74563	55242	48985	24574
R_{int}	0.0615	0.0415	0.1194	0.1229
Parameters	5365	3508	4999	3457
Restraints	427	49	151	282
GooF (F^2)	1.024	1.028	0.947	1.070
R_1 ($I_o > 2\sigma[I_o]$)	0.0819	0.0561	0.0654	0.0987
R_1 (all reflections)	0.0954	0.0605	0.1228	0.1520
wR_2 ($I_o > 2\sigma[I_o]$)	0.2149	0.1557	0.1429	0.2195
wR_2 (all reflections)	0.2279	0.1616	0.1795	0.2428
Largest Peak (eÅ^{-3})	1.263	1.751	0.701	0.834
Largest Hole (eÅ^{-3})	-1.095	-1.066	-0.664	−0.700
CCDC Number	2444122	2444123	2444124	2444099

Continuation of **Table S6**

Complex	(*S*,*S*)-cycHC[8]·2(b)	(*R*,*R*)-cycHC[8]·3(a)	(*R*,*R*)-cycHC[8]·3(b)	(*S*,*S*)-cycHC[8]·3
Figure	**Figure S105**	**Figure S106**	**Figure S107**	**Figure S108**
Formula	$C_{246}H_{277}Cl_5N_{40}O_{18}Zn_2$	$C_{222}H_{256}Cl_8F_8N_{40}O_{18}Zn_2$	$C_{108}H_{120}F_4N_{20}O_8Zn$	$C_{109}H_{122}Cl_2F_4N_{20}O_8Zn$
Formula Weight (gmol^{-1})	4390.05	4338.99	1967.60	2052.53
Colour & Habit	Purple plate	Purple plate	Purple plate	Purple block
Crystal Dimensions (mm)	0.05 × 0.15 × 0.22	0.05 × 0.11 × 0.32	0.07 × 0.16 × 0.24	0.17 × 0.20 × 0.21
Crystal System	Orthorhombic	Triclinic	Monoclinic	Triclinic
Space Group	$P2_12_12_1$	$P1$	$P2_1$	$P1$
a (Å)	17.8849(6)	15.7096(4)	17.7073(9)	15.7204(5)
b (Å)	23.5013(9)	17.4955(5)	23.8275(8)	17.4902(6)
c (Å)	27.3120(11)	23.5977(5)	26.656(2)	23.6063(8)
α (°)	90	87.101(2)	90	87.218(3)
β (°)	90	85.150(2)	104.978(6)	85.209(2)
γ (°)	90	64.879(3)	90	65.004(2)
V (Å)	11479.7(7)	5850.5(3)	10864.7(11)	5861.5(4)
Z	2	1	4	2
ρ_{calc} (gcm^{-3})	1.270	1.232	1.203	1.163
F(000)	4644	2280	4152	2160
μ (mm^{-1})	1.367	1.692	0.882	1.247
Temperature (K)	120.0(1)	120.0(1)	120.0(1)	120.0(1)
ϑ_{max} [°]	70.0	76.7	70.0	70.0
Total Reflections	46330	44535	64862	84099
Independent Reflections	21740	27498	40278	35817
Reflections ($I_o > 2\sigma[I_o]$)	13962	25488	18142	23808
R_{int}	0.0954	0.0432	0.1026	0.1220
Parameters	1432	2663	2540	2593
Restraints	13	99	181	84
GooF (F^2)	1.004	1.027	0.964	1.075
R_1 ($I_o > 2\sigma[I_o]$)	0.0657	0.0649	0.0904	0.0834
R_1 (all reflections)	0.1144	0.0696	0.1836	0.1160
wR_2 ($I_o > 2\sigma[I_o]$)	0.1249	0.1773	0.1921	0.1911
wR_2 (all reflections)	0.1482	0.1849	0.2462	0.2069
Largest Peak (eÅ^{-3})	0.768	1.767	0.992	0.835
Largest Hole (eÅ^{-3})	-0.482	-1.136	-0.511	-0.566
CCDC Number	2444100	2444126	2444127	2444109

Continuation of **Table S6**

**Complex**	**(*R*,*R*)-cycHC[8]·4(a)**	**(*R*,*R*)-cycHC[8]·4(b)**	**(*S*,*S*)-cycHC[8]·4**	**(*R*,*R*)-cycHC[8]·5**
Figure	**Figure S109**	**Figure S110**	**Figure S111**	**Figure S112**
Formula	$C_{222}H_{256}Cl_{16}N_{40}O_{18}Zn_2$	$C_{326}H_{364}Cl_{16}N_{60}O_{24}Zn_3$	$C_{326}H_{364}Cl_{16}N_{60}O_{24}Zn_3$	$C_{130}H_{135}Cl_3F_{12}N_{20}O_8Zn$
Formula Weight (gmol^{-1})	4470.59	6270.06	6270.06	2505.29
Colour & Habit	Purple needle	Purple plate	Purple plate	Purple plate
Crystal Dimensions (mm)	0.05 × 0.06 × 0.19	0.03 × 0.11 × 0.22	0.04 × 0.19 × 0.23	0.14 × 0.30 × 0.32
Crystal System	Triclinic	Monoclinic	Monoclinic	Monoclinic
Space Group	$P1$	$P2_1$	$P2_1$	$P2_1$
a (Å)	14.0546(2)	25.5193(6)	25.5567(10)	17.9398(4)
b (Å)	17.4000(3)	17.5251(3)	17.4842(5)	19.5619(6)
c (Å)	27.7241(3)	40.0876(11)	39.5961(19)	17.9629(3)
α (°)	76.1650(10)	90	90	90
β (°)	78.6430(10)	106.769(3)	104.655(3)	105.968(2)
γ (°)	66.528(2)	90	90	90
V (Å)	5999.03(17)	17165.9(7)	17117.4(12)	6060.6(3)
Z	1	2	2	2
ρ_{calc} (gcm^{-3})	1.237	1.213	1.217	1.373
F(000)	2344	6588	6588	2616
μ (mm^{-1})	2.416	1.933	1.939	1.606
Temperature (K)	120.0(1)	120.0(1)	120.0(1)	120.0(1)
ϑ_{max} [°]	76.2	70.0	70.0	70.0
Total Reflections	117590	185968	129367	41958
Independent Reflections	46382	61840	64783	18631
Reflections ($I_o > 2\sigma[I_o]$)	39591	18930	32232	14412
R_{int}	0.0478	0.1510	0.0929	0.0395
Parameters	2671	3723	3862	1567
Restraints	48	1575	435	175
GooF (F^2)	1.014	0.956	1.038	1.038
R_1 ($I_o > 2\sigma[I_o]$)	0.0570	0.1299	0.0731	0.0634
R_1 (all reflections)	0.0687	0.2403	0.1456	0.0789
wR_2 ($I_o > 2\sigma[I_o]$)	0.1443	0.3139	0.1309	0.1608
wR_2 (all reflections)	0.1540	0.4390	0.1557	0.1721
Largest Peak (eÅ^{-3})	1.006	1.159	0.517	0.904
Largest Hole (eÅ^{-3})	-0.712	-0.511	-0.442	-0.712
CCDC Number	2444128	2444129	2444110	2444130

Continuation of **Table S6**

**Complex**	**(*S*,*S*)-cycHC[8]·5**	**(*R*,*R*)-cycHC[8]·7**	**(*S*,*S*)-cycHC[8]·7**
Figure	**Figure S113**	**Figure S114**	**Figure S115**
Formula	$C_{118}H_{150}F_{12}N_{20}O_{17}Zn$	$C_{113}H_{142}Cl_2MgN_{20}O_{12}$	$C_{109}H_{126}Cl_2MgN_{20}O_8$
Formula Weight (gmol^{-1})	2413.94	2067.67	1939.50
Colour & Habit	Purple plate	Purple needle	Purple plate
Crystal Dimensions (mm)	0.03 × 0.08 × 0.16	0.05 × 0.05 × 0.25	0.03 × 0.08 × 0.36
Crystal System	Triclinic	Tetragonal	Tetragonal
Space Group	*P*1	$P4_122$	$P4_322$
a (Å)	14.2548(7)	13.99900(10)	14.0038(2)
b (Å)	14.5465(7)	13.99900(10)	14.0038(2)
c (Å)	17.3206(9)	60.6405(8)	60.6508(8)
α (°)	87.280(3)	90	90
β (°)	67.198(2)	90	90
γ (°)	70.066(2)	90	90
V (Å)	3097.6(3)	11883.8(2)	11894.0(4)
Z	1	4	4
ρ_{calc} (gcm^{-3})	1.294	1.156	1.083
F(000)	1272	4408	4120
μ (mm^{-1})	1.020	1.059	1.004
Temperature (K)	120.0(1)	120.0(1)	120.0(1)
ϑ_{max} [°]	70.0	76.6	70.0
Total Reflections	57423	72745	88846
Independent Reflections	22542	12365	11269
Reflections ($I_o > 2\sigma[I_o]$)	13965	11529	7974
R_{int}	0.1124	0.0501	0.0511
Parameters	1537	674	634
Restraints	41	1	1
GooF (F^2)	1.034	1.105	1.076
R_1 ($I_o > 2\sigma[I_o]$)	0.0661	0.0825	0.0802
R_1 (all reflections)	0.1200	0.0866	0.0975
wR_2 ($I_o > 2\sigma[I_o]$)	0.1267	0.2308	0.2030
wR_2 (all reflections)	0.1515	0.2340	0.2105
Largest Peak (eÅ^{-3})	0.516	0.898	1.030
Largest Hole (eÅ^{-3})	-0.589	-1.125	-0.927
CCDC Number	2444111	2444131	2444112

Table S7 Characteristics of cycHC[*n*]·Guest complexes in solid state.

No	Complex	Stoichiometry (cycHC[n] : por)	Nr. of pentacoordinated metals and their average distance M···O (Å)	Nr. of hexacoordinated metals and their average distance M···O	Figure(s) of the structure
1	(*R,R*)-cycHC[6]·**1**	2:3	2, 2.251 ± 0.017	1, 2.425 ± 0.070	**Figure S85**
2	(*S,S*)-cycHC[6]·**1**	1:2	2, 2.262 ± 0.013	0	**Figure S86**
3	(*S,S*)-cycHC[6]·**2(a)**[b]	1:1 (polymer)	0	1, 2.386 ± 0.045	**Figure S87**
4	(*S,S*)-cycHC[6]·**2(b)**	1 + 1:1[c]	1, 2.138	0	**Figure S88**
5	(*R,R*)-cycHC[6]·**3**	1:1 (polymer)	0	1, 2.378 ± 0.038	**Figure S89**
6	(*S,S*)-cycHC[6]·**3**	2:2	1, 2.126	1, 2.369 ± 0.009	**Figure S90**
7	cycHC[6]·**4**[a]	1 + 1:2[c]	2, 2.167 ± 0.018	0	**Figure S91** **Figure S92**
8	cycHC[6]·**5**[a]	1:1 (polymer)	0	1, 2.380 ± 0.041	**Figure S93** **Figure S94**
9	(*R,R*)-cycHC[6]·**6**	1:1 (polymer)	0	1, 2.363 ± 0.075	**Figure S96**
10	cycHC[6]·**7**[a]	1:1 (polymer)	0	1, 2.144 ± 0.084	**Figure S96** **Figure S97**
11	(*S,S*)-cycHC[8]·**1**	1:2	2, 2.127 ± 0.060	0	**Figure S98**
12	(*S,S*)-cycHC[8]·**1**	1:2 (2D polymer)	0	2, 2.420 ± 0.061	**Figure S99**
13	cycHC[8]·**2**[a,b]	1:2	2, 2.166 ± 0.023	0	**Figure S100** **Figure S104**
14	cycHC[8]·**2**[a]	1:1	1, 2.193 ± 0.056	0	**Figure S103** **Figure S105**
15	cycHC[8]·**3**[a]	1:1	1, 2.151 ± 0.016	0	**Figure S106** **Figure S107** **Figure S108**
16	cycHC[8]·**4**[a]	1:1	1, 2.155 ± 0.010	0	**Figure S109** **Figure S110** **Figure S111**
17	cycHC[8]·**5**[a]	1:1	1, 2.146 ± 0.003	0	**Figure S112** **Figure S113**
18	cycHC[8]·**7**[a]	1:1 (polymer)	0	1, 2.263 ± 0.000	**Figure S114** **Figure S115**

[a] average from crystal structures of both enantiomers, [b] include structure published in ChemComm 2019[3], [c] one cycHC[*n*] molecule is not attached to any porphyrin.

5.1 Discrete complexes and coordination polymers featuring cycHC[6]:

Crystal data for (*R,R*)-cycHC[6] · **1**, **2[2(*R,R*)-cycHC[6] · 3ZnOEP] · CH_2Cl_2 (+solvate)**: CCDC-2444113, $C_{409}H_{554}Cl_2N_{72}O_{24}Zn_6$, M = 7326.33 $gmol^{-1}$, purple plate, 0.140 × 0.119 × 0.054 mm^3, monoclinic, space group $P2_1$ (No. 4), a = 26.3928(2) Å, b = 23.0687(2) Å, c = 32.7554(2) Å, α = 90°, β = 96.9300(10)°, γ = 90°, V = 19797.3(3) $Å^3$, Z = 2, D_{calc} = 1.229 gcm^{-3}, F(000) = 7836, μ = 1.082 mm^{-1}, T = 120(2) K, θ_{max} = 75.86°, 153591 total reflections, 65520 with $I_o > 2\sigma(I_o)$, R_{int} = 0.0526, 78383 data, 4666 parameters, 73 restraints, GooF = 1.012, R_1 = 0.0455 and wR_2 = 0.0986 [$I_o > 2\sigma(I_o)$], R_1 = 0.0600 and wR_2 = 0.1074 (all reflections), 0.659 < dΔρ < -0.295 $eÅ^{-3}$, Flack parameter x = 0.018(9).

(R,R)-cycHC[6] (4.6 mg, 0.005 mmol, 1 eq.) was dissolved in DCM/MeOH (*v*:*v*, 1:1, 1 mL), and added to a pinkish solution of ZnOEP (3.0 mg, 0.005 mmol, 1 eq.) in DCM (0.5 mL). Crystals of (*R,R*)-cycHC[6] · **1** were obtained after 4 days *via* slow evaporation at -20 °C. The asymmetric unit consists of two discrete 2:3 complexes of (*R,R*)-cycHC[6] with ZnOEP, one molecule of CH_2Cl_2, and other unknown solvates. Due to the size of the asymmetric unit, BLOC commands were used for anisotropic refinement and the model required a few SIMU restraints to adequately model the structure. No sensible disordered model could be formulated for the unknown solvates which would match the observed electron density, so the computer program SQUEEZE[15] within PLATON[16] was used to account for the electron density in this region of the unit cell. The program identified solvent accessible voids totaling 1361 $Å^3$ and 246 electrons per unit cell were recovered. The formula weight, density, etc. listed above do not include any correction for the missing solvate.

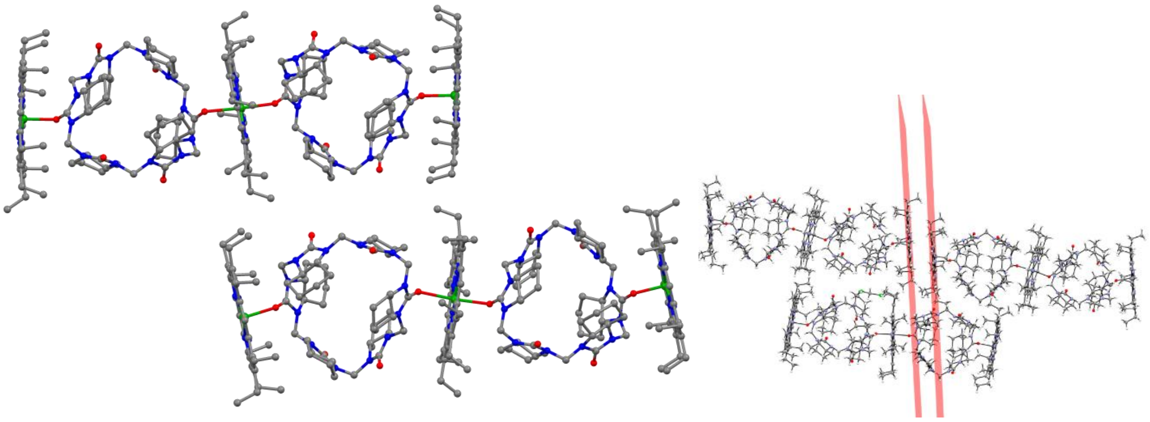

Figure S85 Ball-and-stick-model of (*R,R*)-cycHC[6] **1**. Hydrogen atoms as well as co-crystallized solvent molecules have been omitted for clarity. Distances Zn...Zn = 4.83 Å, porphyrin—porphyrin (plane to plane) 3.3 = Å. Selected interatomic distances (Å): Zn1···O1 2.243(5), Zn2···O4 2.539(6), Zn2···O7 2.361(6), Zn3···O10 2.227(5), Zn4···O13 2.270(5), Zn5···O16 2.425(6), Zn5···O19 2.375(5), Zn6···O22 2.265(5).

Crystal data for (*S,S*)-cycHC[6] · **1**, **2[(*S,S*)-cycHC[6] · 2ZnOEP] · 4.5CH₂Cl₂ (+solvate)**: CCDC-2444098, $C_{244.50}H_{329}Cl_9N_{40}O_{12}Zn_4$, M = 4600.99 gmol^{-1}, red block, 0.28 × 0.26 × 0.25 mm^3, triclinic, space group *P*1 (No. 1), a = 13.5387(5) Å, b = 19.9529(6) Å, c = 25.3189(6) Å, α = 68.9340(10)°, β = 82.797(2)°, γ = 74.217(2)°, V = 6138.9(3) Å^3, Z = 1, D_{calc} = 1.245 gcm^{-3}, F(000) = 2445, μ = 1.858 mm^{-1}, T = 120(2) K, θ_{max} = 74.37°, 113384 total reflections, 22202 with $I_o > 2\sigma(I_o)$, R_{int} = 0.0595, 44441 data, 2738 parameters, 309 restraints, GooF = 1.021, R_1 = 0.0868 and wR_2 = 0.1970 [$I_o > 2\sigma(I_o)$], R_1 = 0.1394 and wR_2 = 0.2193 (all reflections), 1.386 < dΔρ < -1.331 eÅ^{-3}, Flack parameter x = 0.059(14).

(*S,S*)-cycHC[6] (4.6 mg, 0.005 mmol, 1 eq.) was dissolved in DCM/MeOH (*v*:*v*, 1:1, 1 mL), and added to a pinkish solution of ZnOEP (3.0 mg, 0.005 mmol, 1 eq.) in DCM (1 mL). Crystals of (*S,S*)-cycHC[6] · **1** were obtained after 1.5 weeks *via* slow evaporation at -20 °C. The asymmetric unit consists of two discrete 1:2 complexes of (*S,S*)-cycHC[6] with ZnOEP, 4.5 co-crystallized DCM molecules, and other unknown solvates. The structure required a large number of restraints (DFIX, RIGU) to adequately model it. No sensible disordered model could be formulated for the unknown solvates which would match the observed electron density, so the computer program SQUEEZE[15] within PLATON[16] was used to account for the electron density in this region of the unit cell. The program identified solvent accessible voids totaling 424 Å^3 and 85 electrons per unit cell were recovered. The formula weight, density, etc. listed above do not include any correction for the missing solvate.

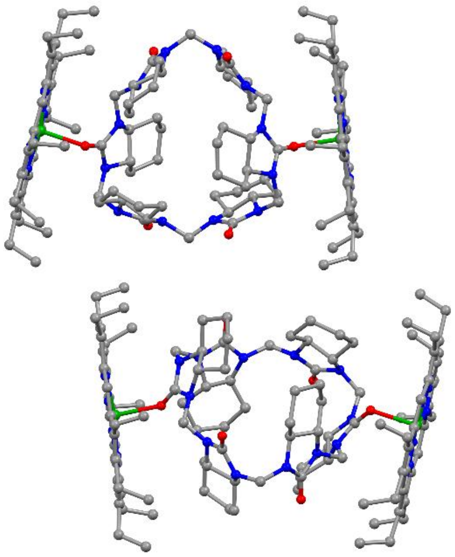

Figure S86 Ball-and-stick-model of (*S,S*)-cycHC[6] · **1**. Hydrogen atoms as well as co-crystallized solvent molecules have been omitted for clarity. Distances Zn…Zn = 4.87 Å, porphyrin—porphyrin (plane to plane) = 3.4 Å. Selected interatomic distances (Å): Zn1···O1 2.258(11), Zn2···O4 2.245(10), Zn3···O7 2.261(11), Zn4···O10 2.282(10).

Crystal data for (*S,S*)-cycHC[6] · **2(a)**, {[(***S,S***)**-cycHC[6] · ZnTPP**]}$^{1}_{\infty}$ **(+solvate)**: CCDC-2444107, $C_{92}H_{100}N_{16}O_6Zn$, M = 1591.24 gmol^{-1}, purple plate, 0.37 × 0.13 × 0.07 mm^3, orthorhombic, space group *C*222$_1$ (No. 20), a = 28.9308(7) Å, b = 29.1900(8) Å, c = 23.1944(5) Å, α = 90°, β = 90°, γ = 90°, V = 19587.4(8) Å^3, Z = 8, D_{calc} = 1.079 gcm^{-3}, F(000) = 6736, μ = 0.785 mm^{-1}, T = 120(2) K, θ_{max} = 76.24°, 97331 total reflections, 11716 with $I_o > 2\sigma(I_o)$, R_{int} = 0.0762, 18567 data, 966 parameters, 51 restraints, GooF = 1.039, R_1 = 0.0806 and wR_2 = 0.1982 [$I_o > 2\sigma(I_o)$], R_1 = 0.1115 and wR_2 = 0.2123 (all reflections), 0.761 < dΔρ < -0.498 eÅ^{-3}, Flack parameter x = 0.196(19).

(*S,S*)-cycHC[6] (4.6 mg, 0.005 mmol, 1 eq.) was dissolved in DCM/MeOH (*v*:*v*, 1:1, 1 mL), and added to a purple solution of ZnTPP (3.4 mg, 0.005 mmol, 1 eq.) in DCM (0.5 mL). Crystals of (*S,S*)-cycHC[6] · **2(a)** were obtained after 4 days *via* slow evaporation at 3 °C. The asymmetric unit consists of a monomeric unit of one-dimensional, linear chain coordination polymer of alternating (*S,S*)-cycHC[6] macrocycles and ZnTPP, and other unknown solvates. The structure required some restraints (DFIX, RIGU, FLAT) to adequately model it. No sensible disordered model could be formulated for the unknown solvates which would match the observed electron density, so the computer program SQUEEZE[15] within PLATON[16] was used to account for the electron density in this region of the unit cell. The program identified solvent accessible voids totaling 5532 Å^3 and 248 electrons per unit cell were recovered. The formula weight, density, etc. listed above do not include any correction for the missing solvate.

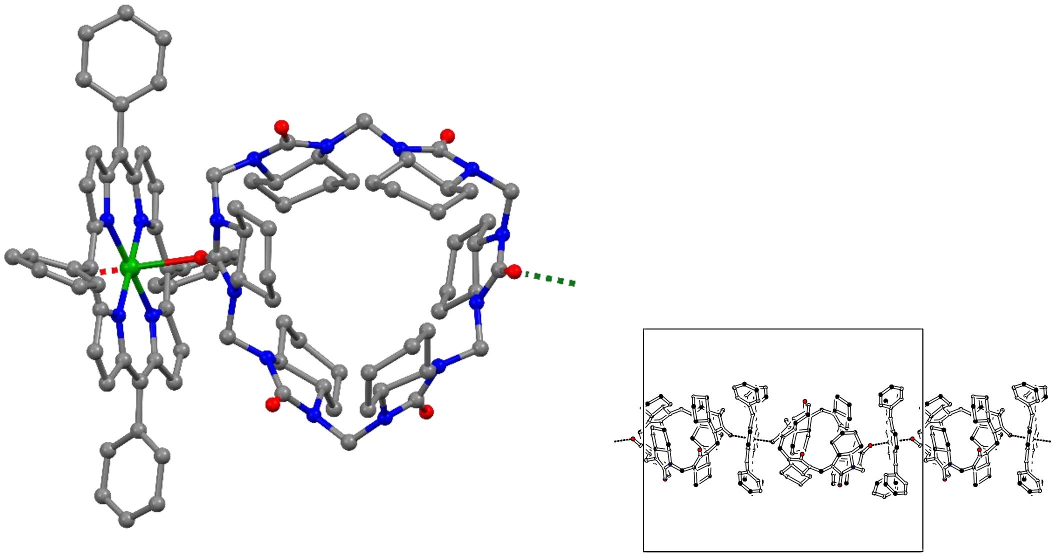

Figure S87 Ball-and-stick-model of (*S,S*)-cycHC[6] · **2(a)**. Hydrogen atoms as well as co-crystallized solvent molecules have been omitted for clarity. 1D polymeric strand along [1 0 0] direction. Selected interatomic distances (Å): Zn1···O1 2.333(5), Zn1···O4^a 2.409(5). Symmetry operator: a = 1/2−*x*, −1/2+*y*, 1/2−*z*.

Crystal data for (*S,S*)-cycHC[6] · **2(b)**, **[(*S,S*)-cycHC[6] · ZnTPP] · (*S,S*)-cycHC[6] · CH_2Cl_2 (+solvate)**: CCDC-2444108, $C_{141}H_{174}Cl_2N_{28}O_{12}Zn$, M = 2589.34 $gmol^{-1}$, purple needle, 0.301 × 0.031 × 0.017 mm^3, monoclinic, space group $P2_1$ (No. 4), a = 12.3448(6) Å, b = 48.1538(19) Å, c = 12.4691(5) Å, α = 90°, β = 90.233(2)°, γ = 90°, V = 7412.2(6) $Å^3$, Z = 2, D_{calc} = 1.160 gcm^{-3}, F(000) = 2752, μ = 1.081 mm^{-1}, T = 120(2) K, θ_{max} = 67.54°, 62005 total reflections, 17727 with $I_o > 2\sigma(I_o)$, R_{int} = 0.0999, 25046 data, 1657 parameters, 10 restraints, GooF = 1.040, R_1 = 0.0741 and wR_2 = 0.1549 [$I_o > 2\sigma(I_o)$], R_1 = 0.1062 and wR_2 = 0.1715 (all reflections), 0.438 < dΔρ < -0.533 $eÅ^{-3}$, Flack parameter x = 0.05(2).

(*S,S*)-cycHC[6] (4.6 mg, 0.005 mmol, 1 eq.) was dissolved in DCM/MeOH (*v*:*v*, 1:1, 1 mL), and added to a purple solution of ZnTPP (3.4 mg, 0.005 mmol, 1 eq.) in DCM (0.5 mL). Crystals of (*S,S*)-cycHC[6] · **2(b)** were obtained after 3 days *via* slow evaporation at ambient temperature. The asymmetric unit consists of one 1:1 complex of (*S,S*)-cycHC[6] macrocycles and ZnTPP, one uncoordinated (*S,S*)-cycHC[6], one co-crystallized DCM molecule, and other unknown solvates. The structure required a few restraints (RIGU) to adequately model it. No sensible disordered model could be formulated for the unknown solvates which would match the observed electron density, so the computer program SQUEEZE[15] within PLATON[16] was used to account for the electron density in this region of the unit cell. The program identified solvent accessible voids totaling 1327 $Å^3$ and 386 electrons per unit cell were recovered. The formula weight, density, etc. listed above do not include any correction for the missing solvate.

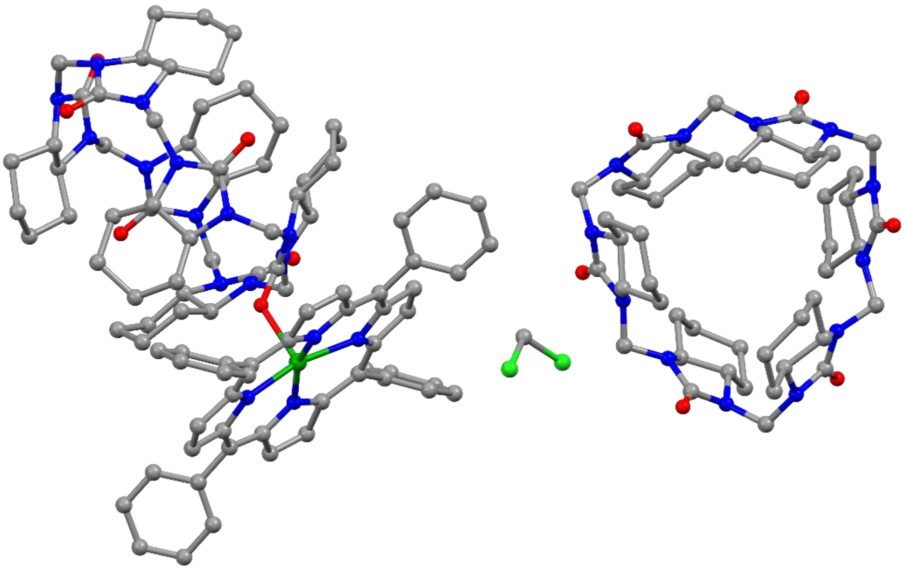

Figure S88 Ball-and-stick-model of (*S,S*)-cycHC[6] · **2(b)**. Hydrogen atoms have been omitted for clarity. Selected interatomic distances (Å): Zn1···O1 2.138(5).

Crystal data for (*R,R*)-cycHC[6] · **3**, **4{[(*R,R*)-cycHC[6] · Zn(p-F)TPP]}$^{1}_{\infty}$ · CH_3OH (+solvate)**: CCDC-2444114, $C_{369}H_{388}F_{16}N_{64}O_{25}Zn_4$, M = 6684.89 gmol^{-1}, purple plate, 0.194 × 0.078 × 0.016 mm^3, monoclinic, space group $P2_1$ (No. 4), a = 23.4044(16) Å, b = 29.1054(18) Å, c = 25.121(2) Å, α = 90°, β = 102.186(7)°, γ = 90°, V = 16727(2) Å^3, Z = 2, D_{calc} = 1.327 gcm^{-3}, F(000) = 7028, μ = 1.019 mm^{-1}, T = 120(2) K, θ_{max} = 70.39°, 128990 total reflections, 25568 with $I_o > 2\sigma(I_o)$, R_{int} = 0.1531, 67593 data, 4282 parameters, 385 restraints, GooF = 1.081, R_1 = 0.1679 and wR_2 = 0.3895 [$I_o > 2\sigma(I_o)$], R_1 = 0.2614 and wR_2 = 0.4772 (all reflections), 1.265 < dΔρ < -1.562 eÅ^{-3}, Flack parameter x = 0.11(6).

(*R,R*)-cycHC[6] (4.6 mg, 0.005 mmol, 1 eq.) was dissolved in DCM/MeOH (*v*:*v*, 1:1, 1 mL), and added to a purple solution of Zn(p-F)TPP (3.8 mg, 0.005 mmol, 1 eq.) in DCM (1 mL). Crystals of (*R,R*)-cycHC[6] · **3** were obtained after 6 days *via* slow evaporation at ambient temperature. The asymmetric unit consists of four monomeric units of one-dimensional, linear chain coordination polymers of alternating (*R,R*)-cycHC[6] molecules and Zn(p-F)TPP, one molecule of CH_3OH, and other unknown solvates. Due to the size of the asymmetric unit, BLOC commands were used for anisotropic refinement, and the structure required some restraints (DFIX, SIMU, RIGU) and constraints (EADP) to adequately model it. No sensible disordered model could be formulated for the unknown solvates which would match the observed electron density, so the computer program SQUEEZE[15] within PLATON[16] was used to account for the electron density in this region of the unit cell. The program identified solvent accessible voids totaling 1085 Å^3 and 274 electrons per unit cell were recovered. The formula weight, density, etc. listed above do not include any correction for the missing solvate.

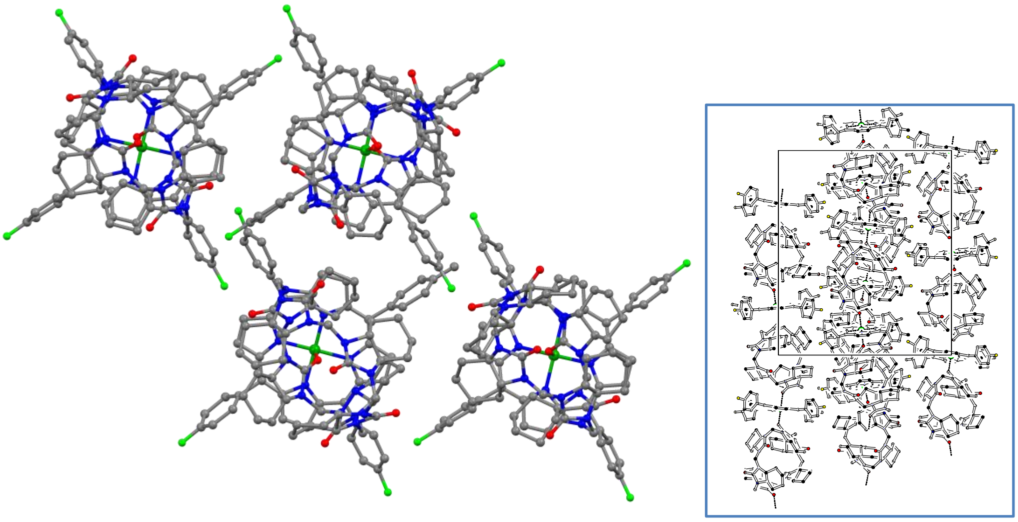

Figure S89 Ball-and-stick-model of (*R,R*)-cycHC[6] **3**. Hydrogen atoms as well as co-crystallized solvent molecules have been omitted for clarity. 1D polymeric strands along [1 0 0] direction. Selected interatomic distances (Å): Zn1···O1 2.399(17), Zn1···O4^a 2.411(15), Zn2···O7 2.356(17), Zn2···O10^b 2.372(18), Zn3···O13 2.369(17), Zn3···O16^c 2.444(15), Zn4···O19 2.309(16), Zn4···O22^d 2.367(15). Symmetry operator: a = −x, 0.5+y, 1−z; b = −x, 0.5+y, −z; c = 1−x, 0.5+y, 1−; d = 1−x, 0.5+y, 2−z.

Crystal data for (*S,S*)-cycHC[6] · **3**, **[2(*S,S*)-cycHC[6] · 2Zn(p-F)TPP] · CH_2Cl_2 · $3CH_3OH$ (+solvate)**: CCDC-2444101, $C_{188}H_{206}Cl_2F_8N_{32}O_{15}Zn_2$, M = 3507.48 gmol^{-1}, purple plate, 0.17 × 0.10 × 0.04 mm^3, monoclinic, space group $P2_1$ (No. 4), a = 23.2961(6) Å, b = 15.4998(3) Å, c = 29.6748(7) Å, α = 90°, β = 109.200(2) °, γ = 90°, V = 10119.1(4) Å^3, Z = 2, D_{calc} = 1.151 gcm^{-3}, F(000) = 3688, μ = 1.110 mm^{-1}, T = 120(2) K, θ_{max} = 75.86°, 107427 total reflections, 28190 with $I_o > 2\sigma(I_o)$, R_{int} = 0.0708, 36260 data, 2192 parameters, 19 restraints, GooF = 1.047, R_1 = 0.0512 and wR_2 = 0.1139 [$I_o > 2\sigma(I_o)$], R_1 = 0.0723 and wR_2 = 0.1238 (all reflections), 0.436 < dΔρ < -0.445 eÅ^{-3}, Flack parameter x = -0.006(12).

(*S,S*)-cycHC[6] (4.6 mg, 0.005 mmol, 1 eq.) was dissolved in DCM/MeOH (*v*:*v*, 1:1, 1 mL), and added to a purple solution of 6+Zn(p-F)TPP (3.8 mg, 0.005 mmol, 1 eq.) in DCM (1 mL). Crystals of (*S,S*)-cycHC[6] · **3** were obtained after 6 days *via* slow evaporation at 3 °C. The asymmetric unit consists of one 2:2 complex of (*S,S*)-cycHC[6] macrocycles and ZnTPP (one terminal and one bridging (*S,S*)-cycHC[6]; same with ZnTPP), one co-crystallized DCM molecule, 3 molecules of MeOH, and other unknown solvates. The structure required a few restraints (RIGU) to adequately model it. No sensible disordered model could be formulated for the unknown solvates which would match the observed electron density, so the computer program SQUEEZE[15] within PLATON[16] was used to account for the electron density in this region of the unit cell. The program identified solvent accessible voids totaling 2202 Å^3 and 761 electrons per unit cell were recovered. The formula weight, density, etc. listed above do not include any correction for the missing solvate.

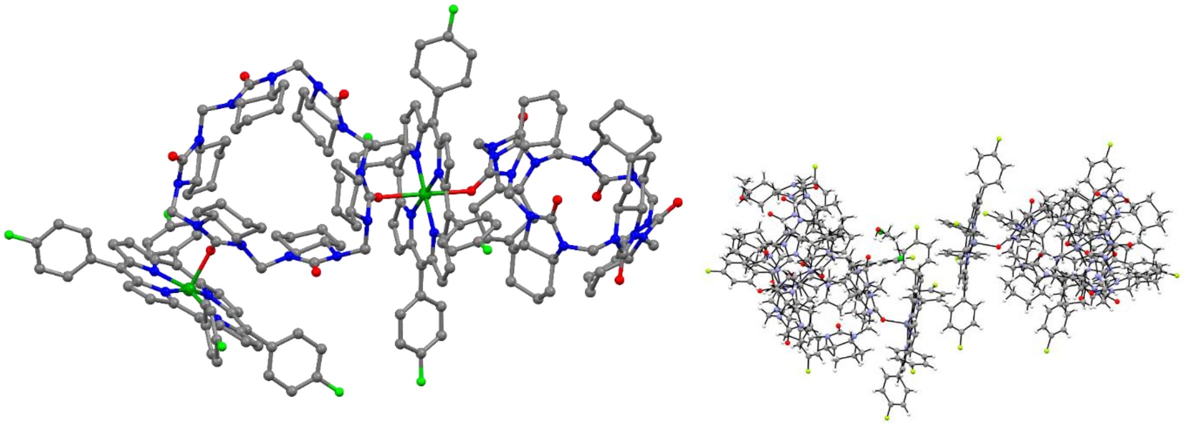

Figure S90 Ball-and-stick-model of (*S,S*)-cycHC[6] · **3**. Hydrogen atoms as well as co-crystallized solvent molecules have been omitted for clarity. Partly off-set, Zn…Zn = 8.85 Å. Selected interatomic distances (Å): Zn1···O1 2.126(4), Zn2···O3 2.360(3), Zn2···O7 2.377(3).

Crystal data for (*R,R*)-cycHC[6] · **4**, **[(*R,R*)-cycHC[6] · 2Zn(p-Cl)TPP] · (*R,R*)-cycHC[6] · 2CH$_2$Cl$_2$ (+solvate)**: CCDC-2444115, $C_{186}H_{196}Cl_{12}N_{32}O_{12}Zn_2$, M = 3627.88 gmol^{-1}, purple plate, 0.21 × 0.19 × 0.03 mm^3, triclinic, space group *P*1 (No. 1), a = 14.3081(4) Å, b = 15.8178(5) Å, c = 23.1376(8) Å, α = 91.125(2)°, β = 105.3220(10)°, γ = 112.634(2)°, V = 4618.0(3) Å^3, Z = 1, D_{calc} = 1.305 gcm^{-3}, F(000) = 1896, μ = 0.501 mm^{-1}, T = 170(2) K, θ_{max} = 28.70°, 33264 total reflections, 15660 with $I_o > 2\sigma(I_o)$, R_{int} = 0.0522, 23552 data, 2198 parameters, 120 restraints, GoF = 1.079, R_1 = 0.0770 and wR_2 = 0.1668 [$I_o > 2\sigma(I_o)$], R_1 = 0.1286 and wR_2 = 0.1954 (all reflections), 0.354 < dΔρ < -0.466 eÅ^{-3}, Flack parameter x = 0.097(18).

(*R,R*)-cycHC[6] (4.6 mg, 0.005 mmol, 1 eq.) was dissolved in DCM/MeOH (*v*:*v*, 1:1, 1 mL), and added to a purple solution of Zn(p-Cl)TPP (4.1 mg, 0.005 mmol, 1 eq.) in DCM (0.5 mL). Crystals of 6+Zn(p-Cl)TPP were obtained after 2 days *via* slow evaporation at ambient temperature. The asymmetric unit consists of one discrete 1:2 complex of (*R,R*)-cycHC[6] with Zn(p-F)TPP, one molecule of uncoordinated (*R,R*)-cycHC[6], two molecules of CH$_2$Cl$_2$, and other unknown solvates believed to be additional heavily disordered molecules of CH$_2$Cl$_2$. The SIMU and RIGU restraints were required to adequately model the structure. No sensible disordered model could be formulated for the unknown solvates which would match the observed electron density, so the computer program SQUEEZE[15] within PLATON[16] was used to account for the electron density in this region of the unit cell. The program identified solvent accessible voids totaling 429 Å^3 and 126 electrons per unit cell were recovered. The formula weight, density, etc. listed above do not include any correction for the missing solvate.

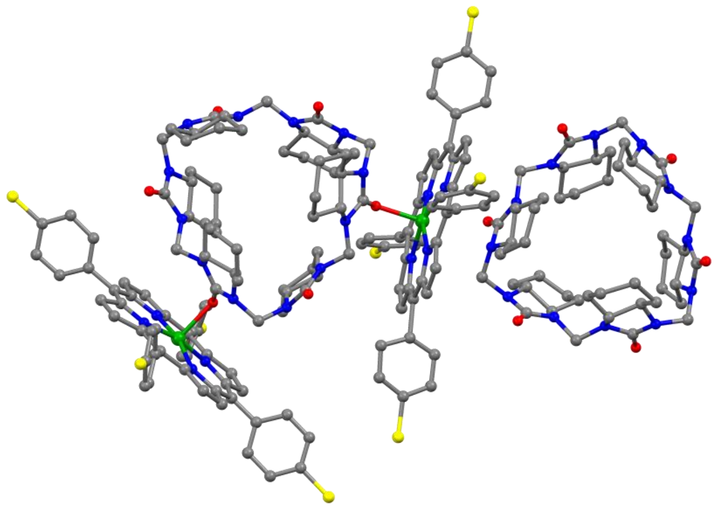

Figure S91 Ball-and-stick-model of (*R,R*)-cycHC[6] **4**. Hydrogen atoms as well as co-crystallized solvent molecules have been omitted for clarity. Selected interatomic distances (Å): Zn1···O1 2.1937(1), Zn2···O3 2.1632(1).

Crystal data for (*S,S*)-cycHC[6] · **4**, **[(*S,S*)-cycHC[6] · 2Zn(p-Cl)TPP] · (*S,S*)-cycHC[6] · 2CH₂Cl₂ (+solvate)**: CCDC-2444102, $C_{186}H_{196}Cl_{12}N_{32}O_{12}Zn_2$, M = 3627.88 gmol^{-1}, purple plate, 0.22 × 0.22 × 0.10 mm^3, triclinic, space group *P*1 (No. 1), a = 14.2130(2) Å, b = 15.7401(2) Å, c = 23.0071(4) Å, α = 91.0790(10)°, β = 105.2780(10)°, γ = 112.869(2)°, V = 4532.07(13) Å^3, Z = 1, D_{calc} = 1.329 gcm^{-3}, F(000) = 1896, μ = 2.504 mm^{-1}, T = 120(2) K, θ_{max} = 76.55°, 100843 total reflections, 32687 with $I_o > 2\sigma(I_o)$, R_{int} = 0.0262, 33097 data, 2197 parameters, 6 restraints, GooF = 1.052, R_1 = 0.0316 and wR_2 = 0.0854 [$I_o > 2\sigma(I_o)$], R_1 = 0.0321 and wR_2 = 0.0859 (all reflections), 0.458 < dΔρ < -0.676 eÅ^{-3}, Flack parameter x = 0.015(4).

(*S,S*)-cycHC[6] (4.6 mg, 0.005 mmol, 1 eq.) was dissolved in DCM/MeOH (*v*:*v*, 1:1, 1 mL), and added to a purple solution of 6+Zn(p-Cl)TPP (4.1 mg, 0.005 mmol, 1 eq.) in DCM (0.5 mL). Crystals of (*S,S*)-cycHC[6] · **4** were obtained after 4 days *via* slow evaporation at 3 °C. The asymmetric unit consists of one 1:2 complex of (*S,S*)-cycHC[6] macrocycles and Zn(p-Cl)TPP, one uncoordinated (*S,S*)-cycHC[6], two co-crystallized DCM molecule, and other unknown solvates. The structure required one restraint (RIGU) to adequately model it. No sensible disordered model could be formulated for the unknown solvates which would match the observed electron density, so the computer program SQUEEZE[15] within PLATON[16] was used to account for the electron density in this region of the unit cell. The program identified solvent accessible voids totaling 392 Å^3 and 92 electrons per unit cell were recovered. The formula weight, density, etc. listed above do not include any correction for the missing solvate.

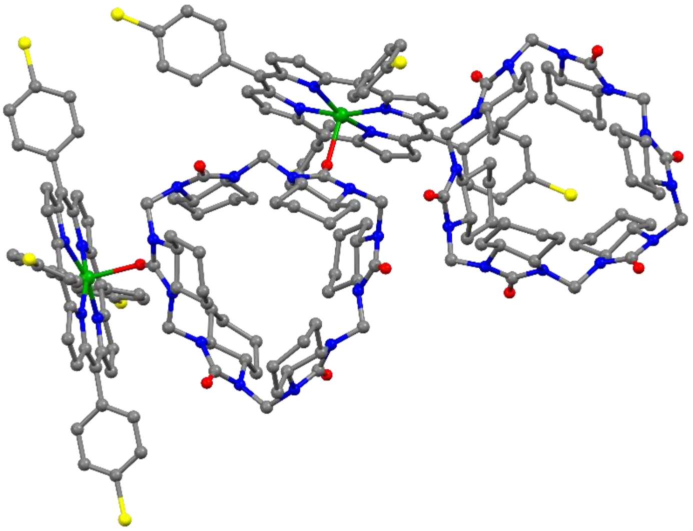

Figure S92 Ball-and-stick-model of (*S,S*)-cycHC[6] · **4**. Hydrogen atoms as well as co-crystallized solvent molecules have been omitted for clarity. Selected interatomic distances (Å): Zn1···O1 2.143(2), Zn2···O3 2.170(2).

Crystal data for (*R,R*)-cycHC[6] · **5**, $\{$**[2(*R,R*)-cycHC[6] · 2Zn(p-CF$_3$)TPP]**$\}_\infty^1$ **(+solvate)**: CCDC-2444116, $C_{192}H_{192}F_{24}N_{32}O_{12}Zn_2$, M = 3726.50 gmol^{-1}, purple plate, 0.157 × 0.097 × 0.032 mm^3, triclinic, space group *P*1 (No. 1), a = 19.8043(4) Å, b = 23.1780(6) Å, c = 23.8651(6) Å, α = 103.744(2)°, β = 97.4830(10)°, γ = 114.265(2)°, V = 9374.2(4) Å^3, Z = 2, D_{calc} = 1.320 gcm^{-3}, F(000) = 3880, μ = 1.090 mm^{-1}, T = 120(2) K, θ_{max} = 72.67°, 175113 total reflections, 32233 with $I_o > 2\sigma(I_o)$, R_{int} = 0.0817, 68278 data, 2066 parameters, 37 restraints, GooF = 1.094, R_1 = 0.1129 and wR_2 = 0.2632 [$I_o > 2\sigma(I_o)$], R_1 = 0.1719 and wR_2 = 0.2981 (all reflections), 1.189 < dΔρ < -0.811 eÅ^{-3}, Flack parameter x = -0.01(2).

(*R,R*)-cycHC[6] (4.6 mg, 0.005 mmol, 1 eq.) was dissolved in DCM/MeOH (*v*:*v*, 1:1, 1 mL), and added to a pinkish solution of Zn(p-CF$_3$)TPP (4.8 mg, 0.005 mmol, 1 eq.) in DCM (1 mL). Crystals of (*R,R*)-cycHC[6] · **5** were obtained after two weeks *via* slow evaporation at -20 °C. The asymmetric unit consists of a monomeric unit of one-dimensional, linear chain coordination polymer of alternating (*R,R*)-cycHC[6] macrocycles and Zn(p-CF$_3$)TPP, and other unknown solvates. The RIGU, DFIX, FLAT restraints were required to adequately model the structure. No sensible disordered model could be formulated for the unknown solvates which would match the observed electron density, so the computer program SQUEEZE[15] within PLATON[16] was used to account for the electron density in this region of the unit cell. The program identified solvent accessible voids totaling 1166 Å^3 and 105 electrons per unit cell were recovered. The formula weight, density, etc. listed above do not include any correction for the missing solvate.

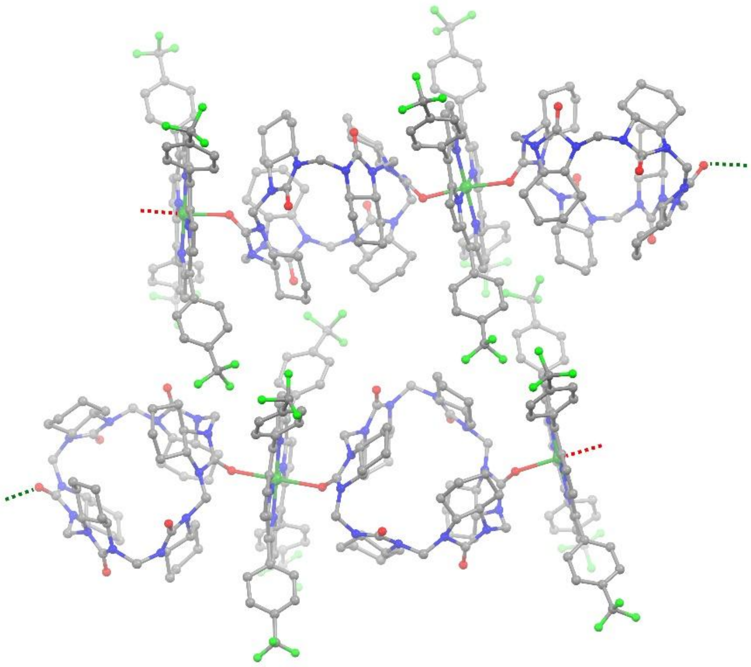

Figure S93 Ball-and-stick-model of (*R,R*)-cycHC[6] · **5**. Hydrogen atoms as well as co-crystallized solvent molecules have been omitted for clarity. Selected interatomic distances (Å): Zn1···O1 2.334(10), Zn1···O10^a 2.390(9), Zn2···O4 2.389(9), Zn2···O7 2.329(9), Zn3···O13 2.329(11), Zn3···O22^b 2.451(9), Zn4···O16, 2.423(9), Zn4···O19 2.345(9). Symmetry operator: a = −1+*x*, *y*, −1+*z*; b = 1+*x*, *y*, 1+*z*.

Crystal data for (*S,S*)-cycHC[6] · **5**, **$\{[2(S,S)\text{-cycHC[6]} \cdot 2Zn(p\text{-}CF_3)TPP]\}^{1}_{\infty}$ (+solvate)**: CCDC-2444103, $C_{192}H_{192}F_{24}N_{32}O_{12}Zn_2$, M = 3726.50 gmol^{-1}, purple plate, 0.27 × 0.24 × 0.09 mm^3, triclinic, space group *P*1 (No. 1), a = 19.9874(3) Å, b = 23.4110(4) Å, c = 24.0257(4) Å, α = 103.375(2)°, β = 98.2680(10)°, γ = 114.929(2)°, V = 9540.1(3) Å^3, Z = 2, D_{calc} = 1.297 gcm^{-3}, F(000) = 3880, μ = 1.071 mm^{-1}, T = 120(2) K, θ_{max} = 75.49°, 167890 total reflections, 53305 with $I_o > 2\sigma(I_o)$, R_{int} = 0.0614, 68115 data, 4717 parameters, 71 restraints, GooF = 1.019, R_1 = 0.0686 and wR_2 = 0.1697 [$I_o > 2\sigma(I_o)$], R_1 = 0.0884 and wR_2 = 0.1840 (all reflections), 0.803 < dΔρ < -0.457 eÅ^{-3}, Flack parameter x = 0.069(10).

(*S,S*)-cycHC[6] (4.6 mg, 0.005 mmol, 1 eq.) was dissolved in DCM/MeOH (*v*:*v*, 1:1, 1 mL), and added to a pinkish solution of 6+Zn(p-CF_3)TPP (4.8 mg, 0.005 mmol, 1 eq.) in DCM (1 mL). Crystals of (*S,S*)-cycHC[6] · **5** were obtained after 8 days *via* slow evaporation at 3 °C. The asymmetric unit consists of a monomeric unit of one-dimensional, linear chain coordination polymer of alternating (*S,S*)-cycHC[6] macrocycles and Zn(p-CF_3)TPP, and other unknown solvates. Due to the size of the asymmetric unit, BLOC commands were used for anisotropic refinement and the structure required RIGU and FLAT restraints to adequately model it. No sensible disordered model could be formulated for the unknown solvates which would match the observed electron density, so the computer program SQUEEZE[15] within PLATON[16] was used to account for the electron density in this region of the unit cell. The program identified solvent accessible voids totaling 1360 Å^3 and 255 electrons per unit cell were recovered. The formula weight, density, etc. listed above do not include any correction for the missing solvate.

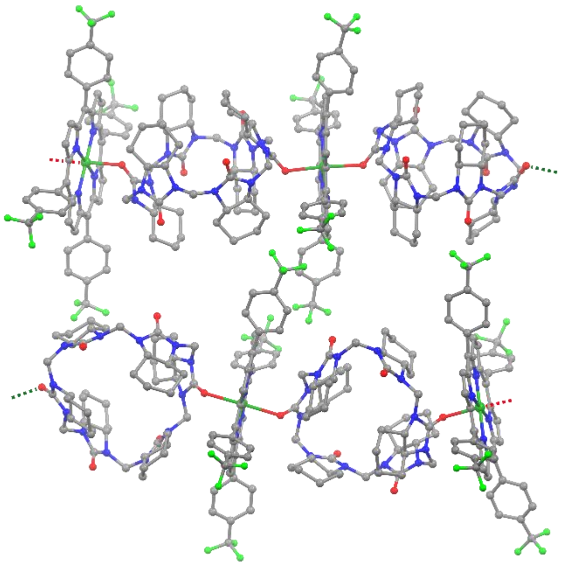

Figure S94 Ball-and-stick-model of (*S,S*)-cycHC[6] · **5**. Hydrogen atoms as well as co-crystallized solvent molecules have been omitted for clarity. Selected interatomic distances (Å): Zn1···O1 2.335(4), Zn1···O10^a 2.429(4), Zn2···O4 2.389(5), Zn2···O7 2.368(5), Zn3···O13 2.360(4), Zn3···O22^b 2.429(4), Zn4···O16 2.434(5), Zn4···O19 2.352(5). Symmetry operator: a = 1+*x*, *y*, 1+*z*; b = −1+*x*, *y*, −1+*z*.

Crystal data for (*R,R*)-cycHC[6] · **6**, $\{$**[2(*R,R*)-cycHC[6] · 2ZnOBrTPFPP]**$\}^{\mathbf{1}}_{\infty}$· **CH_3OH (+solvate)**: CCDC-2444117, $C_{185}H_{148}Br_{16}F_{40}N_{32}O_{13}Zn_2$, M = 5196.65 gmol^{-1}, green plate, 0.19 × 0.14 × 0.03 mm^3, triclinic, space group *P*1 (No. 1), a = 13.9211(3) Å, b = 16.6156(4) Å, c = 23.7795(7) Å, α = 83.376(2) °, β = 77.191(2) °, γ = 66.2890(10) °, V = 4908.6(2) Å^3, Z = 1, D_{calc} = 1.758 gcm^{-3}, F(000) = 2566, μ = 5.042 mm^{-1}, T = 120(2) K, θ_{max} = 75.18°, 99680 total reflections, 18007 with $I_o > 2\sigma(I_o)$, R_{int} = 0.0600, 35681 data, 1206 parameters, 136 restraints, GoF = 1.024, R_1 = 0.0890 and wR_2 = 0.2042 [$I_o > 2\sigma(I_o)$], R_1 = 0.1358 and wR_2 = 0.2240 (all reflections), 2.031 < dΔρ < -1.044 eÅ^{-3}, Flack parameter x = 0.018(13).

(*R,R*)-cycHC[6] (4.6 mg, 0.005 mmol, 1 eq.) was dissolved in DCM/MeOH (*v*:*v*, 1:1, 1 mL), and added to a greenish solution of ZnOBrTPFPP (8.4 mg, 0.005 mmol, 1 eq.) in DCM (0.5 mL). Crystals of (*R,R*)-cycHC[6] · **6** were obtained after 5 days *via* slow evaporation at ambient temperature. The asymmetric unit consists of a monomeric unit of one-dimensional, linear chain coordination polymer of alternating (*R,R*)-cycHC[6] macrocycles and ZnOBrTPFPP, one co-crystallized MeOH molecule, and other unknown solvates. The RIGU, DFIX, FLAT restraints were required to adequately model the structure. In order to avoid physically meaningless ADPs around porphyrin ring of ZnOBrTPFPP, split positions were assigned. The disorder was refined with almost equal split positions (59.4(8)% to 40.6(8) %) with the sum of the site occupancies of both alternative positions constrained to unity. No sensible disordered model could be formulated for the unknown solvates which would match the observed electron density, so the computer program SQUEEZE[15] within PLATON[16] was used to account for the electron density in this region of the unit cell. The program identified solvent accessible voids totaling 5532 Å^3 and 248 electrons per unit cell were recovered. The formula weight, density, etc. listed above do not include any correction for the missing solvate.

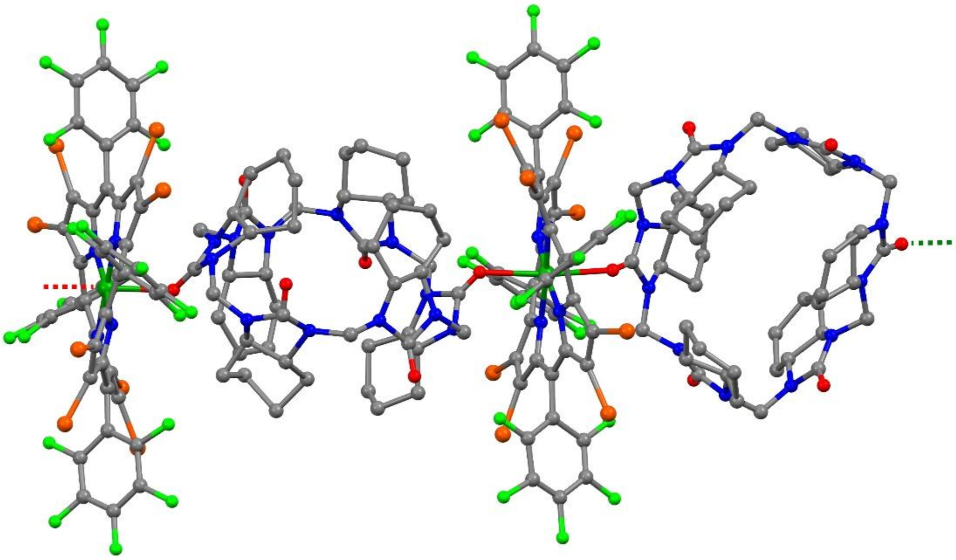

Figure S95 Ball-and-stick-model of a 1D polymeric strand of (*R,R*)-cycHC[6] · **6**. Hydrogen atoms, atom sites of minor occupancy as well as co-crystallized solvent molecules have been omitted for clarity. Selected interatomic distances (Å): Zn1···O1 2.34(3), Zn1···O10^a 2.489(15), Zn2···O4 2.33(3), Zn2···O7 2.294(13). Symmetry operator: a = −1+*x*, 1+*y*, −1+*z*.

Crystal data for (*R,R*)-cycHC[6] · **7**, {**[(*R,R*)-cycHC[6] · MgTPP]**}$^{1}_{\infty}$ **(+solvate)**: CCDC-2444118, $C_{92}H_{100}MgN_{16}O_6$, M = 1550.18 gmol^{-1}, purple block, 0.148 × 0.080 × 0.044 mm^3, orthorhombic, space group *C*222$_1$ (No. 20), a = 29.1542(3) Å, b = 29.3525(3) Å, c = 23.2437(3) Å, α = 90°, β = 90°, γ = 90°, V = 19890.8(4) Å^3, Z = 8, D_{calc} = 1.035 gcm^{-3}, F(000) = 6592, μ = 0.585 mm^{-1}, T = 120(2) K, θ_{max} = 74.18°, 195677 total reflections, 16679 with $I_o > 2\sigma(I_o)$, R_{int} = 0.0250, 20645 data, 1032 parameters, 108 restraints, GoF = 1.051, R_1 = 0.0600 and wR_2 = 0.1682 [$I_o > 2\sigma(I_o)$], R_1 = 0.0735 and wR_2 = 0.1815 (all reflections), 0.399 < dΔρ < -0.409 eÅ^{-3}, Flack parameter x = 0.48(8).

(*R,R*)-cycHC[6] (4.6 mg, 0.005 mmol, 1 eq.) was dissolved in DCM/MeOH (*v*:*v*, 1:1, 1 mL), and added to a purple solution of MgTPP (3.2 mg, 0.005 mmol, 1 eq.) in DCM (1 mL). Crystals of (*R*,*R*)-cycHC[6] · **7** were obtained after 8 days *via* slow evaporation at ambient temperature. The asymmetric unit consists of a monomeric unit of one-dimensional, linear chain coordination polymer of alternating (*R,R*)-cycHC[6] macrocycles and MgTPP, and other unknown solvates. The structure required some restraints (SIMU, RIGU), and in one instance the EADP constraint, to adequately model it. The structure was refined as an inversion twin. No sensible disordered model could be formulated for the unknown solvates which would match the observed electron density, so the computer program SQUEEZE[15] within PLATON[16] was used to account for the electron density in this region of the unit cell. The program identified solvent accessible voids totaling 5535 Å^3 and 1899 electrons per unit cell were recovered. The formula weight, density, etc. listed above do not include any correction for the missing solvate.

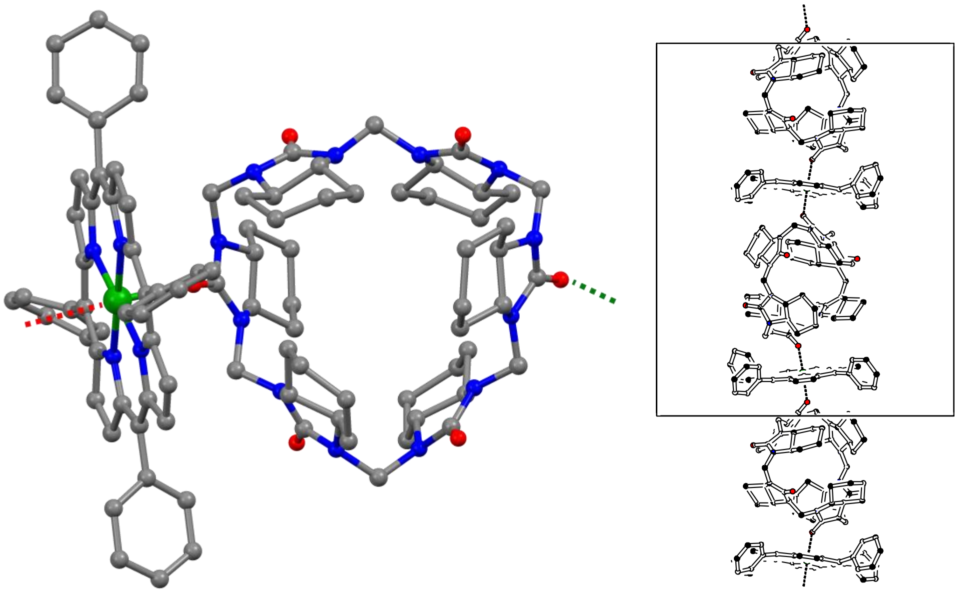

Figure S96 Ball-and-stick-model of (*R,R*)-cycHC[6] · **7**. Hydrogen atoms as well as co-crystallized solvent molecules have been omitted for clarity. 1D polymeric strand along [1 0 0] direction. Selected interatomic distances (Å): Mg1···O1 2.216(7), Mg1···O4^a 2.240(8). Symmetry operator: a = −1/2+*x*, 3/2−*y*, 1−*z*.

Crystal data for (*S,S*)-cycHC[6] · **7**, $\{[(S,S)\text{-cycHC[6]} \cdot \text{MgTPP}]\}^{1}_{\infty}$ **· CH$_2$Cl$_2$ (+solvate)**: CCDC-2444104, $C_{185}H_{202}Cl_2Mg_2N_{32}O_{12}$, M = 3185.29 gmol^{-1}, blue plate, 0.39 × 0.21 × 0.04 mm^3, orthorhombic, space group *C*222$_1$ (No. 20), a = 28.8666(7) Å, b = 29.0639(8) Å, c = 23.1320(5) Å, α = 90°, β = 90°, γ = 90°, V = 19407.2(8) Å^3, Z = 4, D_{calc} = 1.090 gcm^{-3}, F(000) = 6760, μ = 0.858 mm^{-1}, T = 120(2) K, θ_{max} = 73.10°, 76476 total reflections, 9960 with $I_o > 2\sigma(I_o)$, R_{int} = 0.0716, 17881 data, 1130 parameters, 135 restraints, GooF = 1.054, R_1 = 0.0767 and wR_2 = 0.1733 [$I_o > 2\sigma(I_o)$], R_1 = 0.1152 and wR_2 = 0.1851 (all reflections), 0.736 < dΔρ < -0.337 eÅ^{-3}, Flack parameter x = 0.30(3).

(*S,S*)-cycHC[6] (4.6 mg, 0.005 mmol, 1 eq.) was dissolved in DCM/MeOH (*v*:*v*, 1:1, 1 mL), and added to a purple solution of MgTPP (3.2 mg, 0.005 mmol, 1 eq.) in DCM (0.5 mL). Crystals of (*S,S*)-cycHC[6] · **7** were obtained after 2 days *via* slow evaporation at 3 °C. The asymmetric unit consists of a monomeric unit of one-dimensional, linear chain coordination polymer of alternating (*S,S*)-cycHC[6] macrocycles and MgTPP, one co-crystallized DCM molecule, and other unknown solvates. The structure required some restraints (DFIX, RIGU, FLAT) to adequately model it. No sensible disordered model could be formulated for the unknown solvates which would match the observed electron density, so the computer program SQUEEZE[15] within PLATON[16] was used to account for the electron density in this region of the unit cell. The program identified solvent accessible voids totaling 4177 Å^3 and 592 electrons per unit cell were recovered. The formula weight, density, etc. listed above do not include any correction for the missing solvate.

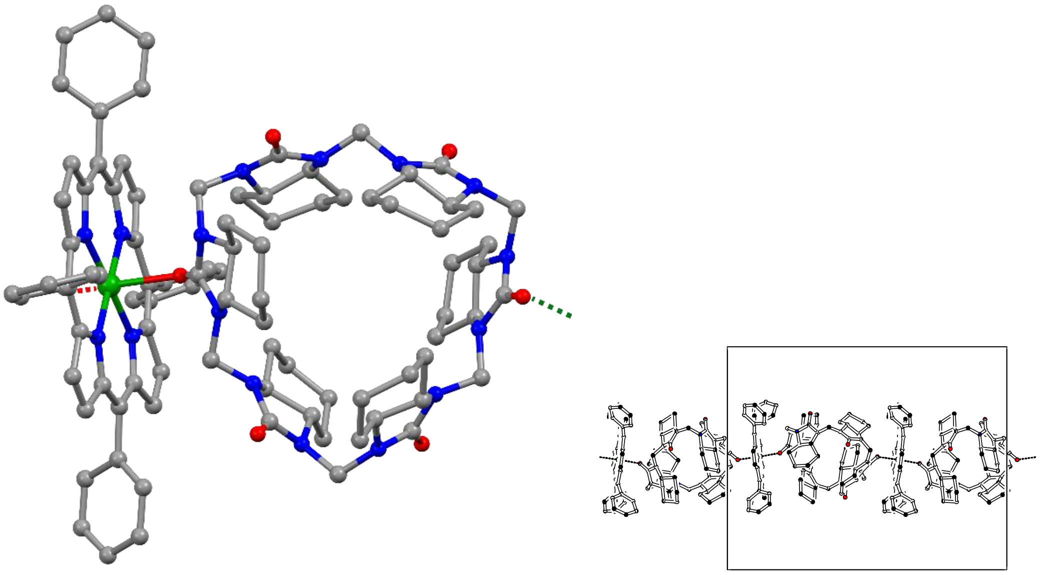

Figure S97 Ball-and-stick-model of (*S,S*)-cycHC[6] · **7**. Hydrogen atoms, atom sites with minor occupancies as well as co-crystallized solvent molecules have been omitted for clarity. 1D polymeric strand along [1 0 0] direction. Selected interatomic distances (Å): Mg1···O1 2.060(6), Mg1···O4^a 2.060(6). Symmetry operator: a = 1/2−*x*, 1/2−*y*, 1/2−*z*.

5.2 Discrete complexes and coordination polymers featuring cycHC[8]:

Crystal data for (*S,S*)-cycHC[8] · **1(a)**, **[(*S,S*)-cycHC[8] · 2ZnOEP] (+solvate)**: CCDC-2444105, $C_{136}H_{184}N_{24}O_8Zn_2$, M = 2413.80 $gmol^{-1}$, pink plate, 0.29 × 0.24 × 0.09 mm^3, triclinic, space group *P*1 (No. 1), a = 14.4432(4) Å, b = 14.4555(5) Å, c = 17.2868(7) Å, α = 79.906(3)°, β = 76.087(2)°, γ = 75.773(2)°, V = 3369.9(2) $Å^3$, Z = 1, D_{calc} = 1.189 gcm^{-3}, F(000) = 1292, μ = 0.935 mm^{-1}, T = 120(2) K, $θ_{max}$ = 74.49°, 62119 total reflections, 9182 with $I_o > 2σ(I_o)$, R_{int} = 0.0608, 24511 data, 1304 parameters, 223 restraints, GooF = 1.013, R_1 = 0.1178 and wR_2 = 0.2508 [$I_o > 2σ(I_o)$], R_1 = 0.1681 and wR_2 = 0.2741 (all reflections), 1.375 < dΔρ < -0.663 $eÅ^{-3}$, Flack parameter x = 0.03(5).

(*S,S*)-cycHC[8] (6.1 mg, 0.005 mmol, 1 eq.) was dissolved in DCM/MeOH (*v*:*v*, 1:1, 1 mL), and added to a pinkish solution of ZnOEP (3.0 mg, 0.005 mmol, 1 eq.) in DCM (1 mL). Crystals of (*S,S*)-cycHC[8] · **1(a)** were obtained after 8 days *via* slow evaporation at 3 °C. The asymmetric unit consists of a discrete 1:2 complex of (*S,S*)-cycHC[8] with ZnOEP, and other unknown solvates. The structure required a large number of restraints (DFIX, RIGU, FLAT) to adequately model it. No sensible disordered model could be formulated for the unknown solvates which would match the observed electron density, so the computer program SQUEEZE[15] within PLATON[16] was used to account for the electron density in this region of the unit cell. The program identified solvent accessible voids totaling 361 $Å^3$ and 29 electrons per unit cell were recovered. The formula weight, density, etc. listed above do not include any correction for the missing solvate.

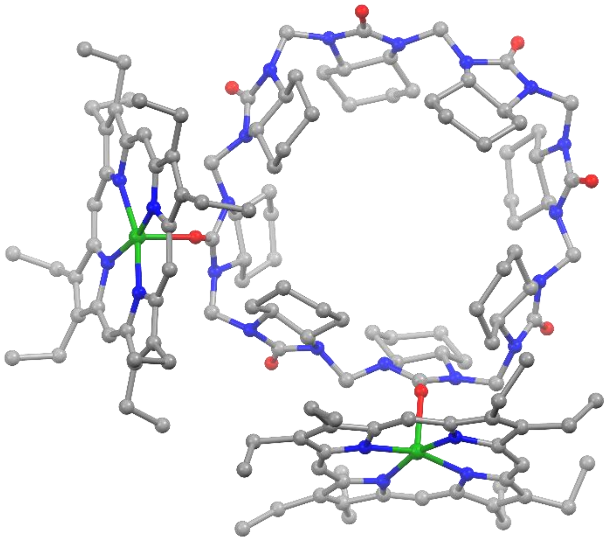

Figure S98 Ball-and-stick-model of (*S,S*)-cycHC[8] · **1(a)**. Hydrogen atoms as well as co-crystallized solvent molecules have been omitted for clarity. Selected interatomic distances (Å): Zn1···O1 2.050(8), Zn2···O3 2.204(11).

Crystal data for (*S,S*)-cycHC[8] · **1(b)**, **{[(*S,S*)-cycHC[8] · 2ZnOEP]}$^{2}_{\infty}$ · 5C$_6$H$_5$Cl · 1.124H$_2$O**: CCDC-2444106, $C_{302}H_{393}Cl_5N_{48}O_{17.12}Zn_4$, M = 5408.27 gmol^{-1}, pink plate, 0.19 × 0.16 × 0.08 mm^3, monoclinic, space group $P2_1$ (No. 4), a = 15.2868(3) Å, b = 23.9951(6) Å, c = 20.3503(5) Å, α = 90°, β = 103.259(2)°, γ = 90°, V = 7265.7(3) Å^3, Z = 1, D_{calc} = 1.236 gcm^{-3}, F(000) = 2883, μ = 1.339 mm^{-1}, T = 120(2) K, θ_{max} = 76.02°, 55618 total reflections, 20385 with $I_o > 2\sigma(I_o)$, R_{int} = 0.0578, 27465 data, 1829 parameters, 115 restraints, GooF = 1.010, R_1 = 0.0657 and wR_2 = 0.1616 [$I_o > 2\sigma(I_o)$], R_1 = 0.0910 and wR_2 = 0.1768 (all reflections), 0.933 < dΔρ < -0.451 eÅ^{-3}, Flack parameter x = 0.024(12).

(*S,S*)-cycHC[8] (6.1 mg, 0.005 mmol, 1 eq.) was dissolved in chlorobenzene/MeOH (*v*:*v*, 1:1, 0.5 mL), and added to a pinkish solution of ZnOEP (3.0 mg, 0.005 mmol, 1 eq.) in chlorobenzene (0.5 mL). Crystals of (*S,S*)-cycHC[8] · **1(b)** were obtained after 2 weeks *via* slow evaporation at 3 °C. The asymmetric unit consists of a monomeric unit of one (*S,S*)-cycHC[8] and two ZnOEPs forming a two-dimensional square-grid framework, five co-crystallized chlorobenzene and 1.124 molecules of water. The structure required a large number of restraints (DFIX, RIGU, FLAT) to adequately model it.

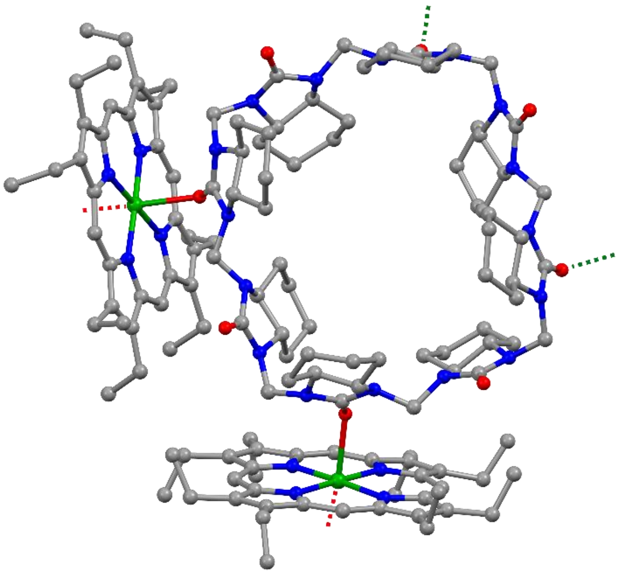

Figure S99 Ball-and-stick-model of (*S,S*)-cycHC[8] · **1(b)**. Hydrogen atoms, atom sites with minor occupancies as well as co-crystallized solvent molecules have been omitted for clarity. Selected interatomic distances (Å): Zn1···O1 2.391(5), Zn1···O7^a 2.505(5), Zn2···O3 2.340(5), Zn2···O5^b 2.442(5). Symmetry operator: a = 2−*x*, −1/2+*y*, 1−*z*; b = 1−*x*, −1/2+*y*, −*z*.

Crystal data for (*R,R*)-cycHC[8] · **2(a)**, **[(*R,R*)-cycHC[8] · 2ZnTPP] · 2CH_2Cl_2 · 2CH_3OH (+solvate)**: CCDC-2444120, $C_{156}H_{164}Cl_4N_{24}O_{10}Zn_2$, M = 2807.64 gmol^{-1}, purple plate, 0.215 × 0.117 × 0.029 mm^3, monoclinic, space group *P*2$_1$ (No. 4), a = 17.746(2) Å, b = 23.728(2) Å, c = 18.111(2) Å, α = 90°, β = 95.469(10)°, γ = 90°, V = 7591.1(15) Å^3, Z = 2, D_{calc} = 1.228 gcm^{-3}, F(000) = 2952, μ = 1.551 mm^{-1}, T = 120(2) K, θ_{max} = 72.75°, 49063 total reflections, 8871 with $I_o > 2\sigma(I_o)$, R_{int} = 0.1571, 30068 data, 1769 parameters, 120 restraints, GooF = 0.917, R_1 = 0.1035 and wR_2 = 0.1869 [$I_o > 2\sigma(I_o)$], R_1 = 0.2197 and wR_2 = 0.1869 (all reflections), 0.692 < dΔρ < -0.627 eÅ^{-3}, Flack parameter x = 0.08(4).

(*R,R*)-cycHC[8] (6.1 mg, 0.005 mmol, 1 eq.) was dissolved in DCM/MeOH (*v*:*v*, 1:1, 1 mL), and added to a purple solution of ZnTPP (3.4 mg, 0.005 mmol, 1 eq.) in DCM (0.5 mL). Crystals of (*R,R*)-cycHC[8] · **2(a)** were obtained after 2 days *via* slow evaporation at ambient temperature. The asymmetric unit consists of a discrete 1:2 complex of (*R,R*)-cycHC[8] macrocycle and ZnTPP, two co-crystallized DCM molecules, two molecules of MeOH, and other unknown solvates. The RIGU, DFIX, SIMU restraints were required to adequately model the structure. No sensible disordered model could be formulated for the unknown solvates which would match the observed electron density, so the computer program SQUEEZE[15] within PLATON[16] was used to account for the electron density in this region of the unit cell. The program identified solvent accessible voids totaling 973 Å^3 and 269 electrons per unit cell were recovered. The formula weight, density, etc. listed above do not include any correction for the missing solvate.

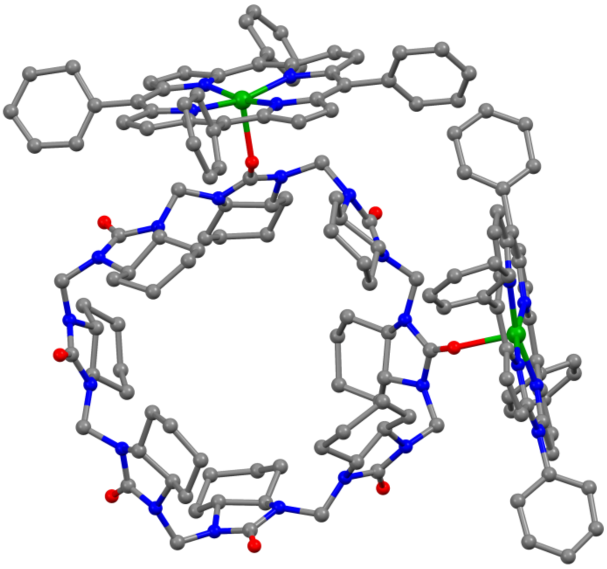

Figure S100 Ball-and-stick-model of (*R,R*)-cycHC[8] · **2(a)**. Hydrogen atoms as well as co-crystallized solvent molecules have been omitted for clarity. Selected interatomic distances (Å): Zn1···O1 2.213(15), Zn2···O3 2.120(10).

Crystal data for (*R,R*)-cycHC[8] · **2(c)**, **3[(*R,R*)-cycHC[8] · 2ZnTPP] · 15CH₂Cl₂ · CH₃OH (+solvate)**: CCDC-2444122, $C_{472}H_{490}Cl_{30}N_{72}O_{25}Zn_6$, M = 9027.05 gmol^{-1}, purple plate, 0.288 × 0.125 × 0.042 mm^3, triclinic, space group *P*1 (No. 1), a = 17.6066(2) Å, b = 18.0973(2) Å, c = 36.1309(5) Å, α = 85.7850(10)°, β = 88.0090(10)°, γ = 89.0700(10)°, V = 11473.3(2) Å^3, Z = 1, D_{calc} = 1.306 gcm^{-3}, F(000) = 4716, μ = 2.508 mm^{-1}, T = 120(2) K, θ_{max} = 75.93°, 215818 total reflections, 74563 with $I_o > 2\sigma(I_o)$, R_{int} = 0.0615, 88216 data, 5365 parameters, 427 restraints, GooF = 1.027, R_1 = 0.0819 and wR_2 = 0.2149 [$I_o > 2\sigma(I_o)$], R_1 = 0.0954 and wR_2 = 0.2279 (all reflections), 1.263 < dΔρ < -1.095 eÅ^{-3}, Flack parameter x = 0.053(14).

Purple plate-like crystals of (*R,R*)-cycHC[8] · **2(c)** were obtained from a mixture of (*R,R*)-cycHC[8] (2.4 mg), ZnTPP (2.4 mg) and 1,4-thioxane (8 mg) in DCM/MeOH (*v*:*v*, 1:1, 2 mL) at ambient temperature. The asymmetric unit consists of three discrete 1:2 complexes of (*R,R*)-cycHC[8] with ZnTPP, 15 co-crystallized DCM molecules, one molecule of MeOH, and other unknown solvates. Due to the size of the asymmetric unit, BLOC commands were used for anisotropic refinement and the structure required DFIX, DELU, SIMU, and RIGU restraints to adequately model it. For three of the disordered phenyl rings, attempts to model the atoms over multiple positions were unsuccessful and thus had to be accounted for using the EADP constraint. No sensible disordered model could be formulated for the unknown solvates which would match the observed electron density, so the computer program SQUEEZE[15] within PLATON[16] was used to account for the electron density in this region of the unit cell. The program identified solvent accessible voids totaling 913 Å^3 and 280 electrons per unit cell were recovered. The formula weight, density, etc. listed above do not include any correction for the missing solvate.

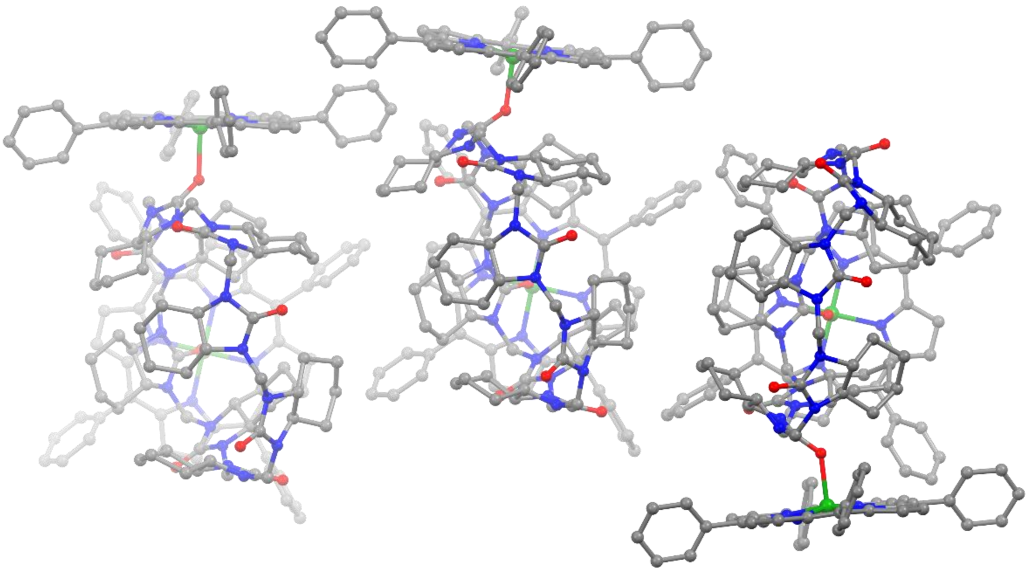

Figure S101 Ball-and-stick-model of (*R,R*)-cycHC[8] · **2(c)**. Hydrogen atoms, atom sites of minor occupancies as well as co-crystallized solvent molecules have been omitted for clarity. Selected interatomic distances (Å): Zn1···O1 2.165(5), Zn2···O3 2.139(6), Zn3···O9 2.190(6), Zn4···O11 2.163(6), Zn5···O17 2.145(5), Zn6···O19 2.142(5).

Crystal data for (*R,R*)-cycHC[8] · **2(d)**, **2[(*R,R*)-cycHC[8] · 2ZnTPP] · 6CH₂Cl₂ · CH₃OH (+solvate)**: CCDC-2444123, $C_{311}H_{320}Cl_{12}N_{48}O_{17}Zn_4$, M = 5689.01 gmol^{-1}, purple plate, 0.142 × 0.073 × 0.066 mm^3, triclinic, space group *P*1 (No. 1), a = 17.0792(2) Å, b = 17.5774(2) Å, c = 26.1799(3) Å, α = 83.3030(10)°, β = 73.3320(10)°, γ = 83.8620(10)°, V = 7455.48(15) Å^3, Z = 1, D_{calc} = 1.267 gcm^{-3}, F(000) = 2982, μ = 1.900 mm^{-1}, T = 120(2) K, θ_{max} = 76.51°, 187435 total reflections, 55242 with $I_o > 2\sigma(I_o)$, R_{int} = 0.0405, 59202 data, 3508 parameters, 49 restraints, GooF = 1.030, R_1 = 0.0561 and wR_2 = 0.1557 [$I_o > 2\sigma(I_o)$], R_1 = 0.0605 and wR_2 = 0.1616 (all reflections), 1.751 < dΔρ < -1.066 eÅ^{-3}, Flack parameter x = 0.052(12).

Purple plate-like crystals of (*R,R*)-cycHC[8] · **2(d)** were obtained from a mixture of (*R,R*)-cycHC[8] (2.4 mg), ZnTPP (2.4 mg) and tetrahydrofuran (8 mg) in DCM/MeOH (*v*:*v*, 1:1, 2 mL) at ambient temperature. The asymmetric unit consists of two discrete 1:2 complexes of (*R,R*)-cycHC[8] with ZnTPP, 6 co-crystallized DCM molecules, one molecule of MeOH, and other unknown solvates. Due to the size of the asymmetric unit, BLOC commands were used for anisotropic refinement and the structure required a few DFIX and RIGU restraints, as well as one EADP constraint, to adequately model it. No sensible disordered model could be formulated for the unknown solvates which would match the observed electron density, so the computer program SQUEEZE[15] within PLATON[16] was used to account for the electron density in this region of the unit cell. The program identified solvent accessible voids totaling 837 Å^3 and 226 electrons per unit cell were recovered. The formula weight, density, etc. listed above do not include any correction for the missing solvate.

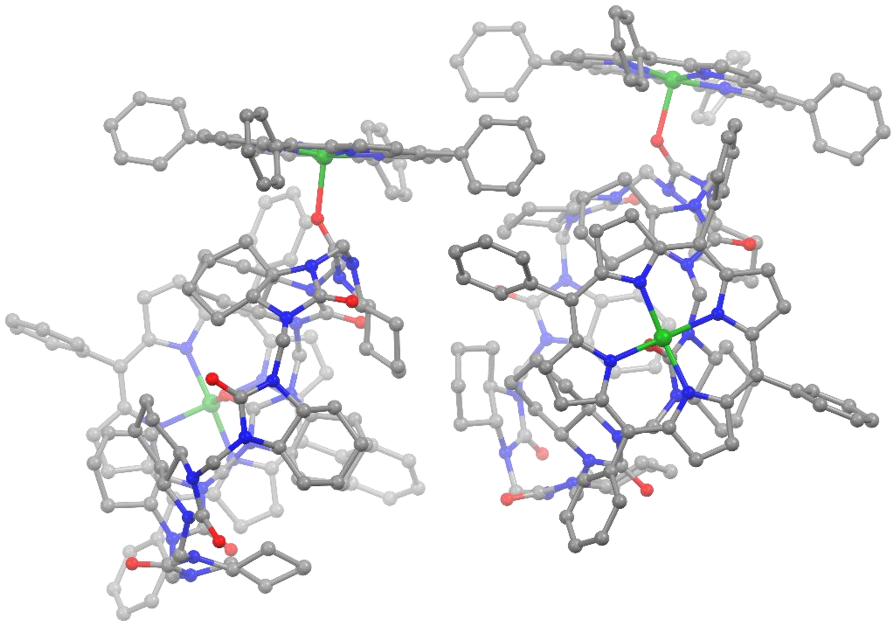

Figure S102 Ball-and-stick-model of (*R,R*)-cycHC[8] · **2(d)**. Hydrogen atoms as well as co-crystallized solvent molecules have been omitted for clarity. Selected interatomic distances (Å): Zn1···O1 2.150(4), Zn2···O3 2.172(3), Zn3···O9 2.158(3), Zn4···O11 2.162(4).

Crystal data for (*R,R*)-cycHC[8] · **2(e)**, **4[(*R,R*)-cycHC[8] · ZnTPP] · 6CH₃OH (+solvate)**: CCDC-2444124, $C_{438}H_{520}N_{80}O_{38}Zn_4$, M = 7774.79 gmol^{-1}, pink plate, 0.097 × 0.061 × 0.024 mm^3, triclinic, space group *P*1 (No. 1), a = 18.0805(6) Å, b = 25.0438(7) Å, c = 25.0965(9) Å, α = 88.646(3)°, β = 71.001(3)°, γ = 87.975(2)°, V = 10737.2(6) Å^3, Z = 1, D_{calc} = 1.202 gcm^{-3}, F(000) = 4132, μ = 0.841 mm^{-1}, T = 120(2) K, $θ_{max}$ = 75.17°, 200352 total reflections, 48985 with $I_o > 2σ(I_o)$, R_{int} = 0.1194, 81807 data, 4999 parameters, 151 restraints, GooF = 0.948, R_1 = 0.0654 and wR_2 = 0.1429 [$I_o > 2σ(I_o)$], R_1 = 0.1228 and wR_2 = 0.1795 (all reflections), 0.701 < dΔρ < -0.664 eÅ^{-3}, Flack parameter x = -0.006(15).

(*R,R*)-cycHC[8] (6.1 mg, 0.005 mmol, 1 eq.) was dissolved in MeOH (2 mL), and added to a purple solution of ZnTPP (3.4 mg, 0.005 mmol, 1 eq.) and (R)-(+)-limonene (6.8 mg, 0.05 mmol, 10 eq.) in MeOH (2 mL). Crystals of (*R,R*)-cycHC[8] · **2(e)** were obtained overnight *via* slow evaporation at ambient temperature. The asymmetric unit consists of four discrete 1:1 complexes of (*R,R*)-cycHC[8] with ZnTPP, 6 co-crystallized MeOH molecules, and other unknown solvates believed to be additional heavily disordered molecules of MeOH. Due to the size of the asymmetric unit, BLOC commands were used for anisotropic refinement and the structure required some restraints (DFIX, SIMU, RIGU) and constraints (EADP) to adequately model it. No sensible disordered model could be formulated for the unknown solvates which would match the observed electron density, so the computer program SQUEEZE[15] within PLATON[16] was used to account for the electron density in this region of the unit cell. The program identified solvent accessible voids totaling 1365 Å^3 and 247 electrons per unit cell were recovered. The formula weight, density, etc. listed above do not include any correction for the missing solvate.

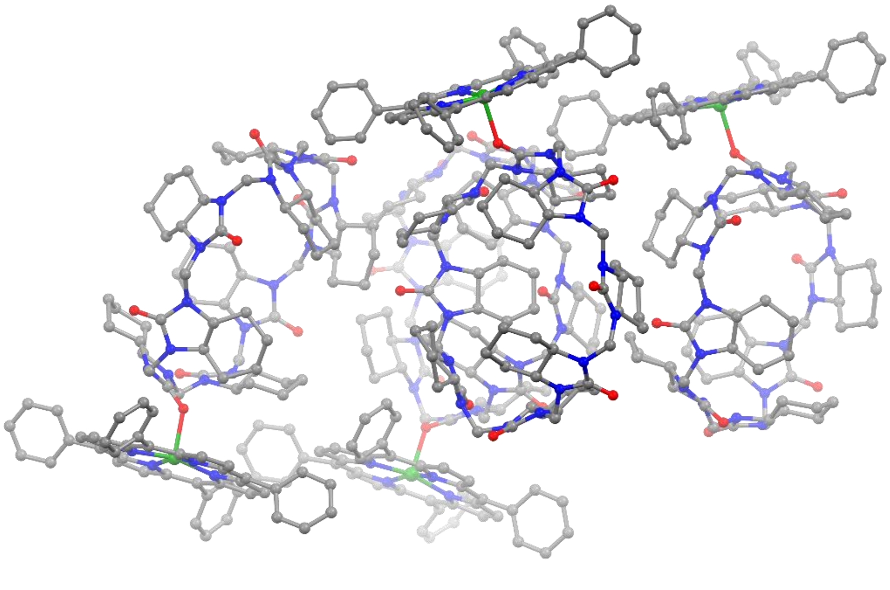

Figure S103 Ball-and-stick-model of (*R,R*)-cycHC[8] · **2(e)**. Hydrogen atoms as well as co-crystallized solvent molecules have been omitted for clarity. Selected interatomic distances (Å): Zn1···O1 2.168(5), Zn2···O9 2.157(6), Zn3···O17 2.176(5), Zn4···O25 2.192(6).

Crystal data for (*S,S*)-cycHC[8] · **2(a)**, **[(*S,S*)-cycHC[8] · 2ZnTPP] · 2CH₂Cl₂ (+solvate)**: CCDC-2444099, $C_{154}H_{156}Cl_4N_{24}O_8Zn_2$, M = 2743.56 gmol^{-1}, purple plate, 0.22 × 0.20 × 0.06 mm^3, triclinic, space group *P*1 (No. 1), a = 17.6776(8) Å, b = 18.0817(6) Å, c = 24.0274(10) Å, α = 88.837(3)°, β = 88.585(2)°, γ = 87.996(2)°, V = 7671.7(5) Å^3, Z = 2, D_{calc} = 1.188 gcm^{-3}, F(000) = 2880, μ = 1.514 mm^{-1}, T = 120(2) K, θ_{max} = 72.11°, 102550 total reflections, 24574 with $I_o > 2\sigma(I_o)$, R_{int} = 0.1229, 46279 data, 3457 parameters, 282 restraints, GooF = 1.070, R_1 = 0.0987 and wR_2 = 0.2195 [$I_o > 2\sigma(I_o)$], R_1 = 0.1520 and wR_2 = 0.2428 (all reflections), 0.834 < dΔρ < -0.700 eÅ^{-3}, Flack parameter x = 0.16(3).

(*S,S*)-cycHC[8] (6.1 mg, 0.005 mmol, 1 eq.) was dissolved in DCM/MeOH (*v*:*v*, 1:1, 1 mL), and added to a purple solution of ZnTPP (3.4 mg, 0.005 mmol, 1 eq.) in DCM (0.5 mL). Crystals of (*S,S*)-cycHC[8] · **2(a)** were obtained after 3 days *via* slow evaporation at 3 °C. The asymmetric unit consists of a discrete 1:2 complex of (*S,S*)-cycHC[8] with ZnTPP, two co-crystallized DCM molecules, and other unknown solvates. Due to the size of the asymmetric unit, BLOC commands were used for anisotropic refinement and the structure required a large number of restraints (DFIX, RIGU, FLAT) to adequately model it. No sensible disordered model could be formulated for the unknown solvates which would match the observed electron density, so the computer program SQUEEZE[15] within PLATON[16] was used to account for the electron density in this region of the unit cell. The program identified solvent accessible voids totaling 1363 Å^3 and 533 electrons per unit cell were recovered. The formula weight, density, etc. listed above do not include any correction for the missing solvate.

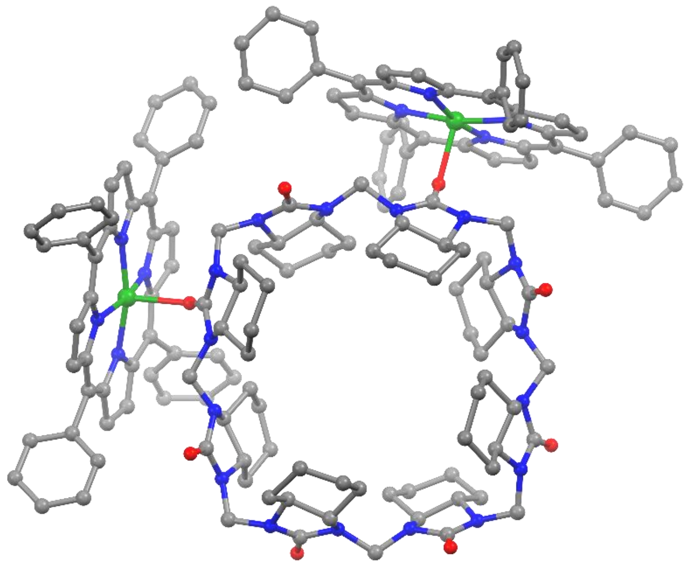

Figure S104 Ball-and-stick-model of (*S,S*)-cycHC[8] · **2(a)**. Hydrogen atoms as well as co-crystallized solvent molecules have been omitted for clarity. Selected interatomic distances (Å): Zn1···O1 2.163(9), Zn2···O3 2.187(10).

Crystal data for (*S,S*)-cycHC[8] · **2(b)**, **2[(*S,S*)-cycHC[8] · ZnTPP] · 5C_6H_5Cl · 2H_2O**: CCDC-2444100, $C_{246}H_{277}Cl_5N_{40}O_{18}Zn_2$, M = 4390.05 gmol^{-1}, purple plate, 0.22 × 0.15 × 0.05 mm^3, orthorhombic, space group $P2_12_12_1$ (No. 19), a = 17.8849(6) Å, b = 23.5013(9) Å, c = 27.3120(11) Å, α = 90°, β = 90°, γ = 90°, V = 11479.7(7) Å^3, Z = 2, D_{calc} = 1.270 gcm^{-3}, F(000) = 4644, μ = 1.367 mm^{-1}, T = 120(2) K, $θ_{max}$ = 76.02°, 46330 total reflections, 13962 with $I_o > 2σ(I_o)$, R_{int} = 0.0954, 21740 data, 1432 parameters, 13 restraints, GooF = 1.004, R_1 = 0.0657 and wR_2 = 0.1249 [$I_o > 2σ(I_o)$], R_1 = 0.1144 and wR_2 = 0.1482 (all reflections), 0.768 < dΔρ < -0.482 eÅ^{-3}, Flack parameter x = 0.009(16).

(*S,S*)-cycHC[8] (6.1 mg, 0.005 mmol, 1 eq.) was dissolved in chlorobenzene/MeOH (*v*:*v*, 1:1, 0.5 mL), and added to a purple solution of ZnTPP (3.4 mg, 0.005 mmol, 1 eq.) in chlorobenzene (0.5 mL). Crystals of (*S,S*)-cycHC[8] · **2(b)** were obtained after 2.5 weeks *via* slow evaporation at ambient temperature. The asymmetric unit consists of two discrete 1:1 complex of (*S,S*)-cycHC[8] with ZnTPP, five co-crystallized chlorobenzene and two water molecules. The structure required a few restraints (DFIX, FLAT) to adequately model it.

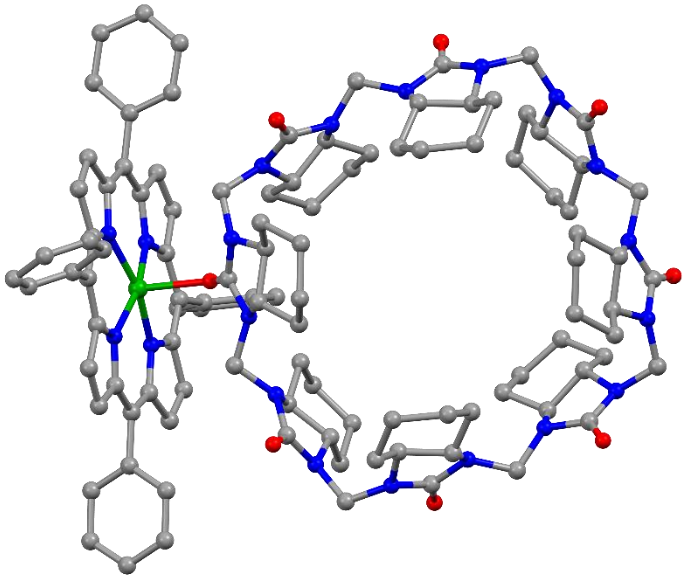

Figure S105 Ball-and-stick-model of one of the discrete complexes in (*S,S*)-cycHC[8] · **2(b)**. Hydrogen atoms as well as co-crystallized solvent molecules have been omitted for clarity. Selected interatomic distances (Å): Zn1···O1 2.137(5).

Crystal data for (*R,R*)-cycHC[8] · **3(a)**, **2[(*R,R*)-cycHC[8] · Zn(p-F)TPP] · 4CH₂Cl₂ · 2CH₃OH (+solvate)**: CCDC-2444126, $C_{222}H_{256}Cl_8F_8N_{40}O_{18}Zn_2$, M = 4338.99 gmol^{-1}, purple plate, 0.317 × 0.110 × 0.049 mm^3, triclinic, space group *P*1 (No. 1), a = 15.7096(4) Å, b = 17.4955(5) Å, c = 23.5977(5) Å, α = 87.101(2)°, β = 85.150(2)°, γ = 64.879(3)°, V = 5850.5(3) Å^3, Z = 1, D_{calc} = 1.232 gcm^{-3}, F(000) = 2280, μ = 1.692 mm^{-1}, T = 120(2) K, θ_{max} = 76.23°, 44535 total reflections, 25488 with $I_o > 2\sigma(I_o)$, R_{int} = 0.0432, 27498 data, 2663 parameters, 99 restraints, GooF = 1.029, R_1 = 0.0649 and wR_2 = 0.1773 [$I_o > 2\sigma(I_o)$], R_1 = 0.0696 and wR_2 = 0.1849 (all reflections), 1.767 < dΔρ < -1.136 eÅ^{-3}, Flack parameter x = 0.035(15).

(*R,R*)-cycHC[8] (6.1 mg, 0.005 mmol, 1 eq.) was dissolved in DCM/MeOH (*v*:*v*, 1:1, 1 mL), and added to a purple solution of Zn(p-F)TPP (3.8 mg, 0.005 mmol, 1 eq.) in DCM (1 mL). Crystals of (*R,R*)-cycHC[8] · **3(a)** were obtained after 2 days *via* slow evaporation at ambient temperature. The asymmetric unit consists of two discrete 1:1 complexes of (*R,R*)-cycHC[8] with Zn(p-F)TPP, 4 co-crystallized DCM molecules, two molecules of MeOH, and other unknown solvates. The structure required some restraints (DFIX, RIGU) and constraints (EADP) to adequately model it. No sensible disordered model could be formulated for the unknown solvates which would match the observed electron density, so the computer program SQUEEZE[15] within PLATON[16] was used to account for the electron density in this region of the unit cell. The program identified solvent accessible voids totaling 817 Å^3 and 210 electrons per unit cell were recovered. The formula weight, density, etc. listed above do not include any correction for the missing solvate.

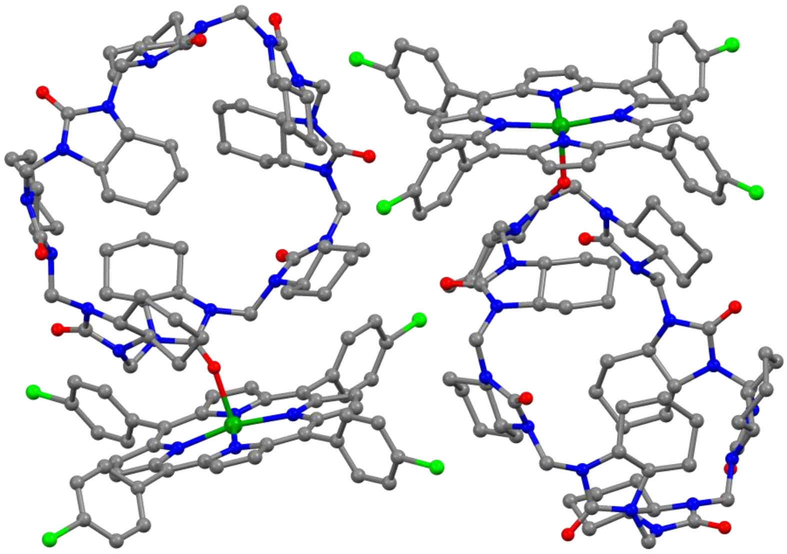

Figure S106 Ball-and-stick-model of (*R,R*)-cycHC[8] · **3(a)**. Hydrogen atoms as well as co-crystallized solvent molecules have been omitted for clarity. Selected interatomic distances (Å): Zn1···O1 2.138(3), Zn2···O9 2.167(4).

Crystal data for (*R,R*)-cycHC[8] · **3(b)**, **[(*R,R*)-cycHC[8] · Zn(p-F)TPP] (+solvate)**: CCDC-2444127, $C_{108}H_{120}F_4N_{20}O_8Zn$, M = 1967.60 gmol^{-1}, purple plate, 0.24 × 0.16 × 0.07 mm^3, monoclinic, space group *P*21 (No. 4), a = 17.7073(9) Å, b = 23.8275(8) Å, c = 26.656(2) Å, α = 90°, β = 104.978(6)°, γ = 90°, V = 10864.7(11) Å^3, Z = 4, D_{calc} = 1.203 gcm^{-3}, F(000) = 4152, μ = 0.882 mm^{-1}, T = 120(2) K, θ_{max} = 60.33°, 64862 total reflections, 18142 with $I_o > 2\sigma(I_o)$, R_{int} = 0.1026, 40278 data, 2540 parameters, 181 restraints, GooF = 0.964, R_1 = 0.0904 and wR_2 = 0.1921 [$I_o > 2\sigma(I_o)$], R_1 = 0.1836 and wR_2 = 0.2462 (all reflections), 0.992 < dΔρ < -0.511 eÅ^{-3}, Flack parameter x = 0.08(4).

(*R,R*)-cycHC[8] (6.1 mg, 0.005 mmol, 1 eq.) was dissolved in DCM/MeOH (*v*:*v*, 1:1, 1 mL), and added to a purple solution of Zn(p-F)TPP (3.8 mg, 0.005 mmol, 1 eq.) and quinine (16.2 mg, 0.05 mmol, 10 eq.) in DCM (1 mL). Crystals of (*R,R*)-cycHC[8] · **3(b)** were obtained after 3 days *via* slow evaporation at ambient temperature. The asymmetric unit consists of two discrete 1:1 complexes of (*R,R*)-cycHC[8] with Zn(p-F)TPP, and other unknown solvates. The structure required some restraints (SIMU) to adequately model it. The structure was refined as an inversion twin. No sensible disordered model could be formulated for the unknown solvates which would match the observed electron density, so the computer program SQUEEZE[15] within PLATON[16] was used to account for the electron density in this region of the unit cell. The program identified solvent accessible voids totaling 1677 Å^3 and 396 electrons per unit cell were recovered. The formula weight, density, etc. listed above do not include any correction for the missing solvate.

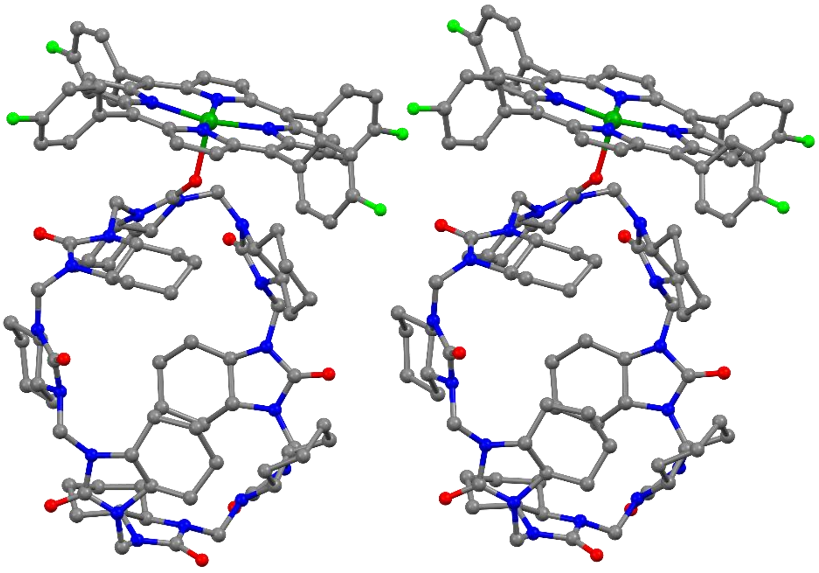

Figure S107 Ball-and-stick-model of (*R,R*)-cycHC[8] · **3(b)**. Hydrogen atoms as well as co-crystallized solvent molecules have been omitted for clarity. Selected interatomic distances (Å): Zn1···O1 2.149(7), Zn2···O9 2.155(8).

Crystal data for (*S,S*)-cycHC[8] · **3**, **[(*S,S*)-cycHC[8] · Zn(p-F)TPP] · CH_2Cl_2 (+solvate)**: CCDC-2444109, $C_{109}H_{122}Cl_2F_4N_{20}O_8Zn$, M = 2052.53 $gmol^{-1}$, purple block, 0.21 × 0.20 × 0.17 mm^3, triclinic, space group *P*1 (No. 1), a = 15.7204(5) Å, b = 17.4902(6) Å, c = 23.6063(8) Å, α = 87.218(3)°, β = 85.209(2)°, γ = 65.004(2)°, V = 5861.5(4) $Å^3$, Z = 2, D_{calc} = 1.163 gcm^{-3}, F(000) = 2160, μ = 1.247 mm^{-1}, T = 120(2) K, θ_{max} = 75.16°, 84099 total reflections, 23808 with $I_o > 2\sigma(I_o)$, R_{int} = 0.1220, 35817 data, 2593 parameters, 84 restraints, GooF = 1.075, R_1 = 0.0834 and wR_2 = 0.1911 [$I_o > 2\sigma(I_o)$], R_1 = 0.1160 and wR_2 = 0.2069 (all reflections), 0.835 < dΔρ < -0.566 $eÅ^{-3}$, Flack parameter x = 0.05(3).

(*S,S*)-cycHC[8] (6.1 mg, 0.005 mmol, 1 eq.) was dissolved in DCM/MeOH (*v*:*v*, 1:1, 1 mL), and added to a purple solution of Zn(p-F)TPP (3.8 mg, 0.005 mmol, 1 eq.) in DCM (1 mL). Crystals of (*S,S*)-cycHC[8] · **3** were obtained after 5 days *via* slow evaporation at 3 °C. The asymmetric unit consists of two discrete 1:1 complex of (*S,S*)-cycHC[8] with Zn(p-F)TPP, one co-crystallized DCM molecule, and other unknown solvates. The structure required some restraints (DFIX, RIGU, FLAT) to adequately model it. No sensible disordered model could be formulated for the unknown solvates which would match the observed electron density, so the computer program SQUEEZE[15] within PLATON[16] was used to account for the electron density in this region of the unit cell. The program identified solvent accessible voids totaling 1195 $Å^3$ and 327 electrons per unit cell were recovered. The formula weight, density, etc. listed above do not include any correction for the missing solvate.

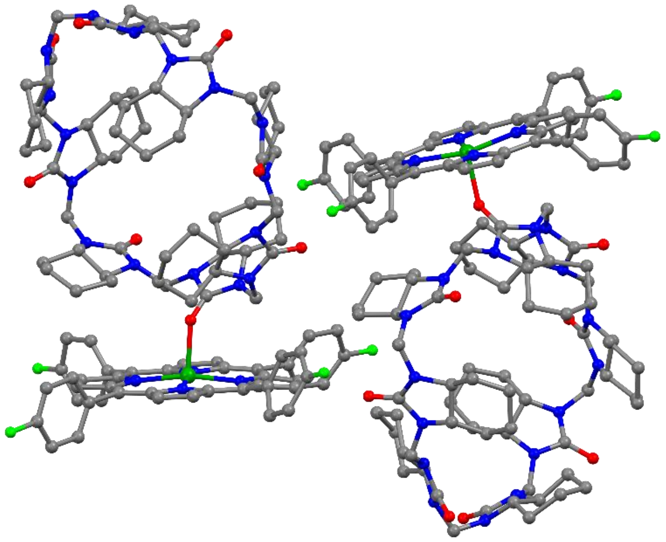

Figure S108 Ball-and-stick-model of (*S,S*)-cycHC[8] · **3**. Hydrogen atoms as well as co-crystallized solvent molecules have been omitted for clarity. Selected interatomic distances (Å): Zn1···O1 2.174(7), Zn2···O9 2.126(7).

Crystal data for (*R,R*)-cycHC[8] · **4(a)**, **2[(*R,R*)-cycHC[8] · Zn(p-Cl)TPP] · 4CH$_2$Cl$_2$ · 2CH$_3$OH (+solvate)**: CCDC-2444128, $C_{222}H_{256}Cl_{16}N_{40}O_{18}Zn_2$, M = 4470.59 gmol^{-1}, purple needle, 0.193 × 0.063 × 0.046 mm^3, triclinic, space group *P*1 (No. 1), a = 14.0546(2) Å, b = 17.4000(3) Å, c = 27.7241(3) Å, α = 76.1650(10)°, β = 78.6430(10)°, γ = 66.528(2)°, V = 5999.03(17) Å^3, Z = 1, D_{calc} = 1.237 gcm^{-3}, F(000) = 2344, μ = 2.416 mm^{-1}, T = 120(2) K, θ_{max} = 74.75°, 117590 total reflections, 39591 with $I_o > 2\sigma(I_o)$, R_{int} = 0.0478, 46382 data, 2671 parameters, 48 restraints, GooF = 1.014, R_1 = 0.0570 and wR_2 = 0.1443 [$I_o > 2\sigma(I_o)$], R_1 = 0.0687 and wR_2 = 0.1540 (all reflections), 1.006 < dΔρ < -0.712 eÅ^{-3}, Flack parameter x = 0.033(11).

(*R,R*)-cycHC[8] (6.1 mg, 0.005 mmol, 1 eq.) was dissolved in DCM/MeOH (*v*:*v*, 1:1, 1 mL), and added to a purple solution of Zn(p-Cl)TPP (4.1 mg, 0.005 mmol, 1 eq.) in DCM (0.5 mL). Crystals of (*R,R*)-cycHC[8] · **4(a)** were obtained after 6 days *via* slow evaporation at ambient temperature. The asymmetric unit consists of two discrete 1:1 complexes of (*R,R*)-cycHC[8] with Zn(p-Cl)TPP, 4 co-crystallized DCM molecules, two molecules of MeOH, and other unknown solvates. The structure required some restraints (SIMU, RIGU) and constraints (EADP) to adequately model it. The structure was refined as an inversion twin. No sensible disordered model could be formulated for the unknown solvates which would match the observed electron density, so the computer program SQUEEZE[15] within PLATON[16] was used to account for the electron density in this region of the unit cell. The program identified solvent accessible voids totaling 791 Å^3 and 204 electrons per unit cell were recovered. The formula weight, density, etc. listed above do not include any correction for the missing solvate.

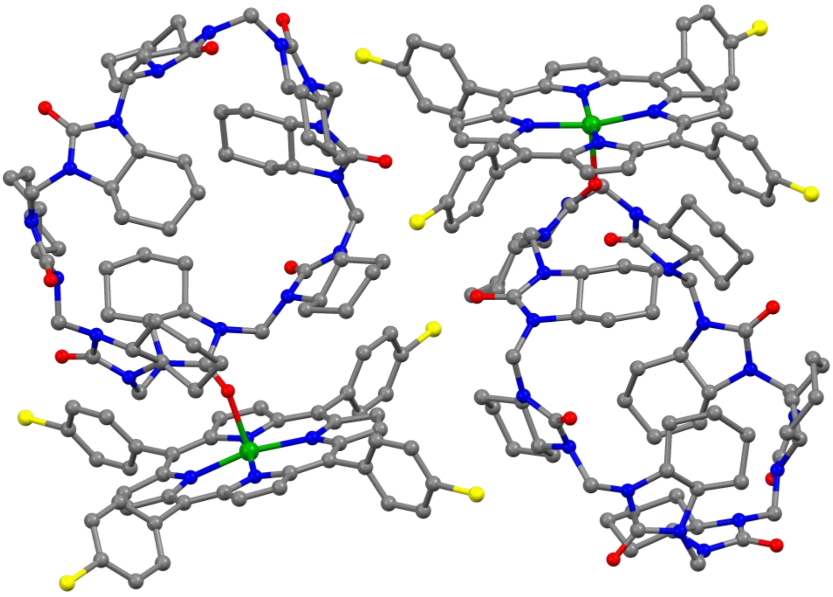

Figure S109 Ball-and-stick-model of (*R,R*)-cycHC[8] · **4(a)**. Hydrogen atoms as well as co-crystallized solvent molecules have been omitted for clarity. Selected interatomic distances (Å): Zn1···O1 2.153(4), Zn2···O9 2.151(4).

Crystal data for (*R,R*)-cycHC[8] · **4(b)**, **3[(*R,R*)-cycHC[8] · Zn(p-Cl)TPP] · 2CH_2Cl_2 (+solvate)**: CCDC-2444129, $C_{326}H_{364}Cl_{16}N_{60}O_{24}Zn_3$, M = 6270.06 $gmol^{-1}$, purple plate, 0.222 × 0.107 × 0.025 mm^3, monoclinic, space group $P2_1$ (No. 4), a = 25.5193(6) Å, b = 17.5251(3) Å, c = 40.0876(11) Å, α = 90°, β = 106.769(3)°, γ = 90°, V = 17165.9(7) $Å^3$, Z = 2, D_{calc} = 1.213 gcm^{-3}, F(000) = 6588, μ = 1.933 mm^{-1}, T = 120(2) K, θ_{max} = 60.34°, 185968 total reflections, 18930 with $I_o > 2\sigma(I_o)$, R_{int} = 0.1510, 61840 data, 3723 parameters, 1575 restraints, GooF = 0.952, R_1 = 0.1299 and wR_2 = 0.3139 [$I_o > 2\sigma(I_o)$], R_1 = 0.2403 and wR_2 = 0.4390 (all reflections), 1.159 < dΔρ < -0.511 $eÅ^{-3}$, Flack parameter x = 0.03(3).

(*R,R*)-cycHC[8] (6.1 mg, 0.005 mmol, 1 eq.) was dissolved in DCM/MeOH (*v*:*v*, 1:1, 1 mL), and added to a purple solution of Zn(p-Cl)TPP (4.1 mg, 0.005 mmol, 1 eq.) and quinine (16.2 mg, 0.05 mmol, 10 eq.) in DCM (1 mL). Crystals of (*R,R*)-cycHC[8] · **4(b)** were obtained after 3 days *via* slow evaporation at ambient temperature. The asymmetric unit consists of three discrete 1:1 complexes of (*R,R*)-cycHC[8] with Zn(p-Cl)TPP, 2 co-crystallized DCM molecules, and other unknown solvates. The structure required a large number of restraints (DFIX, SIMU, RIGU), and in one instance the EADP constraints (EADP), to adequately model it. Due to the size of the asymmetric unit, BLOC commands were used for anisotropic refinement. The structure was refined as an inversion twin. No sensible disordered model could be formulated for the unknown solvates which would match the observed electron density, so the computer program SQUEEZE[15] within PLATON[16] was used to account for the electron density in this region of the unit cell. The program identified solvent accessible voids totaling 2688 $Å^3$ and 904 electrons per unit cell were recovered. The formula weight, density, etc. listed above do not include any correction for the missing solvate.

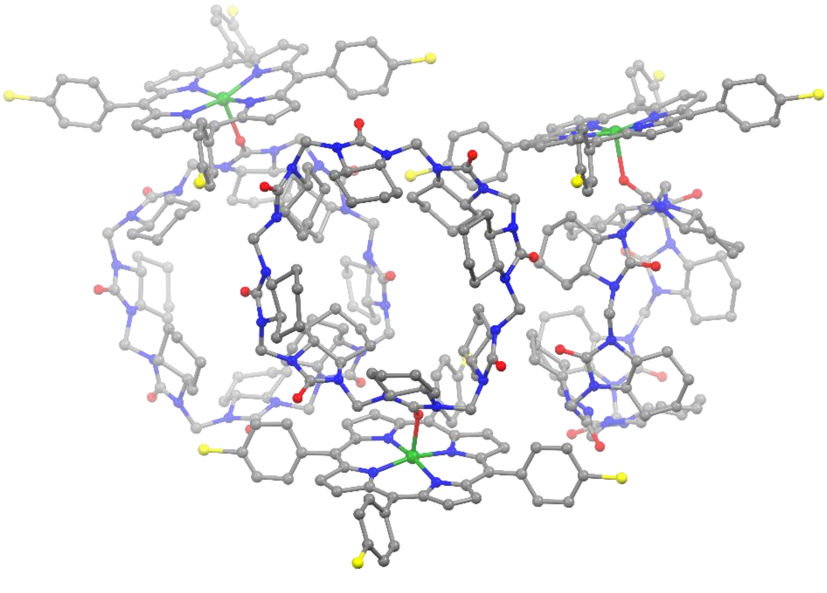

Figure S110 Ball-and-stick-model of (*R,R*)-cycHC[8] · **4(b)**. Hydrogen atoms as well as co-crystallized solvent molecules have been omitted for clarity. Selected interatomic distances (Å): Zn1···O1 2.141(15), Zn2···O9 2.146(13), Zn3···O17 2.165(17).

Crystal data for (*S,S*)-cycHC[8] · **4**, **3[(*S,S*)-cycHC[8] · Zn(p-Cl)TPP] · 2CH₂Cl₂ (+solvate)**: CCDC-2444110, $C_{326}H_{364}Cl_{16}N_{60}O_{24}Zn_3$, M = 6270.06 gmol^{-1}, purple plate, 0.23 × 0.19 × 0.04 mm^3, monoclinic, space group *P*2$_1$ (No. 4), a = 25.5567(10) Å, b = 17.4842(5) Å, c = 39.5961(19) Å, α = 90°, β = 104.655(3)°, γ = 90°, V = 17117.4(12) Å^3, Z = 2, D_{calc} = 1.217 gcm^{-3}, F(000) = 6588, μ = 1.939 mm^{-1}, T = 120(2) K, θ_{max} = 75.36°, 129367 total reflections, 32232 with $I_o > 2\sigma(I_o)$, R_{int} = 0.0929, 64783 data, 3862 parameters, 435 restraints, GooF = 1.038, R_1 = 0.0731 and wR_2 = 0.1309 [$I_o > 2\sigma(I_o)$], R_1 = 0.1456 and wR_2 = 0.1557 (all reflections), 0.517 < dΔρ < -0.442 eÅ^{-3}, Flack parameter x = 0.063(12).

(*S,S*)-cycHC[8] (6.1 mg, 0.005 mmol, 1 eq.) was dissolved in DCM/MeOH (*v*:*v*, 1:1, 1 mL), and added to a purple solution of Zn(p-Cl)TPP (4.1 mg, 0.005 mmol, 1 eq.) in DCM (0.5 mL). Crystals of (*S,S*)-cycHC[8] · **4** were obtained after 4 days *via* slow evaporation at 3 °C. The asymmetric unit consists of three discrete 1:1 complex of (*S,S*)-cycHC[8] with Zn(p-Cl)TPP, two co-crystallized DCM molecules, and other unknown solvates. Due to the size of the asymmetric unit, BLOC commands were used for anisotropic refinement and the structure required a large number of restraints (DFIX, RIGU, FLAT) to adequately model it. No sensible disordered model could be formulated for the unknown solvates which would match the observed electron density, so the computer program SQUEEZE[15] within PLATON[16] was used to account for the electron density in this region of the unit cell. The program identified solvent accessible voids totaling 2675 Å^3 and 753 electrons per unit cell were recovered. The formula weight, density, etc. listed above do not include any correction for the missing solvate.

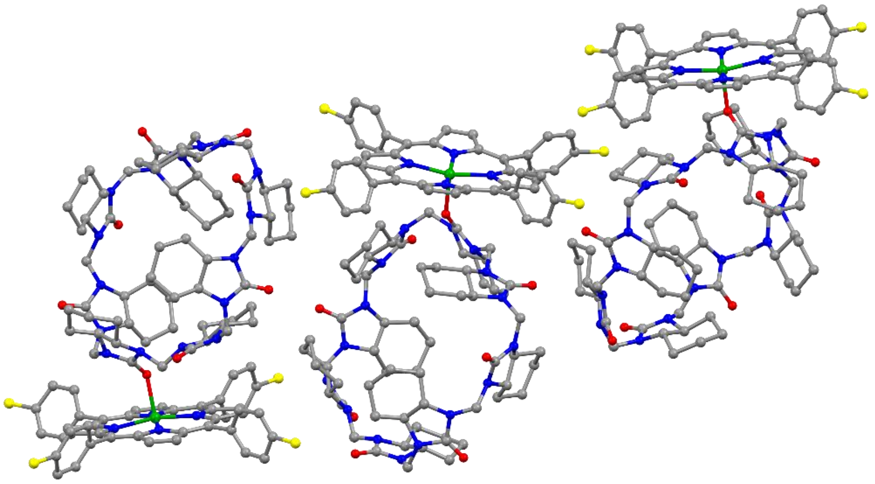

Figure S111 Ball-and-stick-model of (*S,S*)-cycHC[8] · **4**. Hydrogen atoms as well as co-crystallized solvent molecules have been omitted for clarity. Selected interatomic distances (Å): Zn1···O1 2.174(6), Zn2···O9 2.158(6), Zn3···O17 2.148(6).

Crystal data for (*R,R*)-cycHC[8] · **5**, **[(*R,R*)-cycHC[8] · Zn(p-CF_3)TPP] · 3C_6H_5Cl**: CCDC-2444130, $C_{130}H_{135}Cl_3F_{12}N_{20}O_8Zn$, M = 2505.29 gmol^{-1}, purple plate, 0.32 × 0.30 × 0.14 mm^3, monoclinic, space group $P2_1$ (No. 4), a = 17.9398(4) Å, b = 19.5619(6) Å, c = 17.9629(3) Å, α = 90°, β = 105.968(2)°, γ = 90°, V = 6060.6(3) Å^3, Z = 2, D_{calc} = 1.373 gcm^{-3}, F(000) = 2616, μ = 1.606 mm^{-1}, T = 120(2) K, θ_{max} = 75.65°, 41958 total reflections, 14412 with $I_o > 2\sigma(I_o)$, R_{int} = 0.0395, 18631 data, 1567 parameters, 175 restraints, GooF = 1.038, R_1 = 0.0634 and wR_2 = 0.1608 [$I_o > 2\sigma(I_o)$], R_1 = 0.0789 and wR_2 = 0.1721 (all reflections), 0.904 < dΔρ < -0.712 eÅ^{-3}, Flack parameter x = 0.095(10).

(*R,R*)-cycHC[8] (6.1 mg, 0.005 mmol, 1 eq.) was dissolved in chlorobenzene/MeOH (*v*:*v*, 1:1, 0.5 mL), and added to a pinkish solution of Zn(p-CF_3)TPP (4.8 mg, 0.005 mmol, 1 eq.) in chlorobenzene (0.5 mL). Crystals of (*R,R*)-cycHC[8] · **5** were obtained after 1.5 weeks *via* slow evaporation at ambient temperature. The asymmetric unit consists of one discrete 1:1 complex of (*R,R*)-cycHC[8] with Zn(p-CF_3)TPP and three co-crystallized chlorobenzene molecules. A large number of restraints (RIGU, DFIX, FLAT) were required to adequately model the structure.

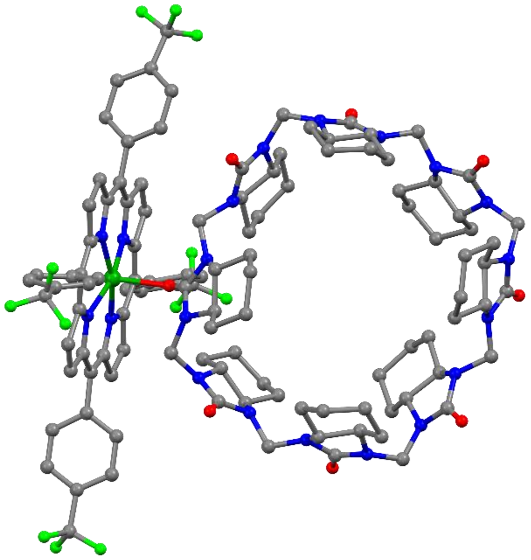

Figure S112 Ball-and-stick-model of (*R,R*)-cycHC[8] · **5**. Hydrogen atoms as well as co-crystallized solvent molecules have been omitted for clarity. Selected interatomic distances (Å): Zn1···O1 2.148(4).

Crystal data for (*S,S*)-cycHC[8] · **5**, **[(*S,S*)-cycHC[8] · Zn(p-CF$_3$)TPP] · 6CH$_3$OH · 3H$_2$O (+solvate)**: CCDC-2444111, $C_{118}H_{150}F_{12}N_{20}O_{17}Zn$, M = 2413.94 gmol^{-1}, purple plate, 0.16 × 0.08 × 0.03 mm^3, triclinic, space group *P*1 (No. 1), a = 14.2548(7) Å, b = 14.5465(7) Å, c = 17.3206(9) Å, α = 87.280(3)°, β = 67.198(2)°, γ = 70.066(2)°, V = 3097.6(3) Å^3, Z = 1, D_{calc} = 1.294 gcm^{-3}, F(000) = 1272, μ = 1.020 mm^{-1}, T = 120(2) K, θ_{max} = 73.96°, 57423 total reflections, 13965 with $I_o > 2\sigma(I_o)$, R_{int} = 0.1124, 22542 data, 1537 parameters, 41 restraints, GooF = 1.034, R_1 = 0.0661 and wR_2 = 0.1267 [$I_o > 2\sigma(I_o)$], R_1 = 0.1200 and wR_2 = 0.1515 (all reflections), 0.516 < dΔρ < -0.589 eÅ^{-3}, Flack parameter x = 0.01(3).

(*S,S*)-cycHC[8] (6.1 mg, 0.005 mmol, 1 eq.) was dissolved in DCM/MeOH (*v*:*v*, 1:1, 1 mL), and added to a pinkish solution of Zn(p-CF$_3$)TPP (4.8 mg, 0.005 mmol, 1 eq.) in DCM (1 mL). Crystals of (*S,S*)-cycHC[8] · **7** were obtained after 7 days *via* slow evaporation at 3 °C. The asymmetric unit consists of one discrete 1:1 complex of (*S,S*)-cycHC[8] with Zn(p-CF$_3$)TPP, six co-crystallized MeOH molecules, three molecules of water, and other unknown solvates. The structure required some restraints (DFIX, RIGU) to adequately model it. No sensible disordered model could be formulated for the unknown solvates which would match the observed electron density, so the computer program SQUEEZE[15] within PLATON[16] was used to account for the electron density in this region of the unit cell. The program identified solvent accessible voids totaling 127 Å^3 and 11 electrons per unit cell were recovered. The formula weight, density, etc. listed above do not include any correction for the missing solvate.

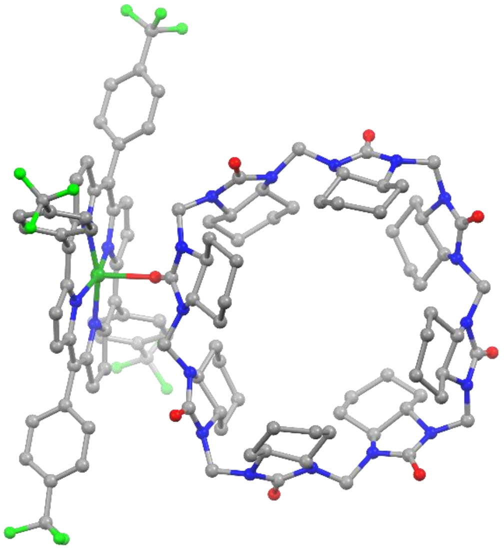

Figure S113 Ball-and-stick-model of (*S,S*)-cycHC[8] · **5**. Hydrogen atoms as well as co-crystallized solvent molecules have been omitted for clarity. Selected interatomic distances (Å): Zn1···O1 2.143(5).

Crystal data for (*R,R*)-cycHC[8] · **7**, **{[(*R,R*)-cycHC[8] · MgTPP]}$^{1}_{\infty}$ · CH_2Cl_2 · $4CH_3OH$(+solvate)**: CCDC-2444131, $C_{113}H_{142}Cl_2MgN_{20}O_{12}$, M = 2067.67 $gmol^{-1}$, purple needle, 0.250 × 0.047 × 0.045 mm^3, tetragonal, space group *P*4$_1$22 (No. 91), a = 13.99900(10) Å, b = 13.99900(10) Å, c = 60.6405(8) Å, α = 90°, β = 90°, γ = 90°, V = 11883.8(2) Å^3, Z = 4, D_{calc} = 1.156 gcm^{-3}, F(000) = 4408, μ = 1.059 mm^{-1}, T = 120(2) K, θ_{max} = 75.37°, 72745 total reflections, 11529 with $I_o > 2\sigma(I_o)$, R_{int} = 0.0322, 12365 data, 674 parameters, 1 restraints, GooF = 1.105, R_1 = 0.0825 and wR_2 = 0.2308 [$I_o > 2\sigma(I_o)$], R_1 = 0.0866 and wR_2 = 0.2340 (all reflections), 0.898 < dΔρ < -1.125 eÅ^{-3}, Flack parameter x = 0.10(6).

(*R,R*)-cycHC[8] (6.1 mg, 0.005 mmol, 1 eq.) was dissolved in DCM/MeOH (*v*:*v*, 1:1, 1 mL), and added to a purple solution of MgTPP (3.2 mg, 0.005 mmol, 1 eq.) in DCM (0.5 mL). Crystals of (*R,R*)-cycHC[8] · **7** were obtained after 3 days *via* slow evaporation at ambient temperature. The asymmetric unit consists of a monomeric unit of one-dimensional, twisted chain coordination polymer of alternating (*R,R*)-cycHC[8] macrocycles and MgTPP, one co-crystallized DCM molecule, 4 molecules of MeOH, and other unknown solvates. The model required a single DFIX restraint to adequately model the hydroxyl group of a methanol molecule. The structure was refined as an inversion twin. No sensible disordered model could be formulated for the unknown solvates which would match the observed electron density, so the computer program SQUEEZE[15] within PLATON[16] was used to account for the electron density in this region of the unit cell. The program identified solvent accessible voids totaling 1995 Å^3 and 549 electrons per unit cell were recovered. The formula weight, density, etc. listed above do not include any correction for the missing solvate.

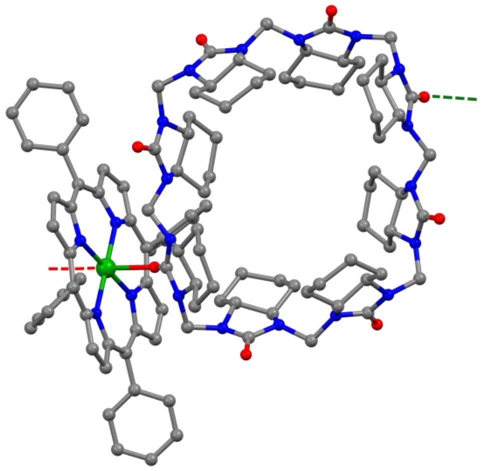

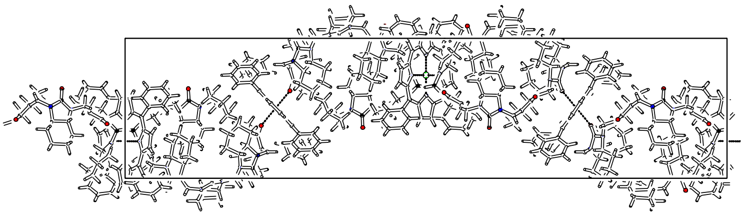

Figure S114 Ball-and-stick-model of (*R,R*)-cycHC[8] · **7**. Hydrogen atoms as well as co-crystallized solvent molecules have been omitted for clarity. 1D polymeric strand along [1 0 0] direction. Selected interatomic distances (Å): Mg1···O1 2.263(4), Mg1···O1^a 2.263(4). Symmetry operator: a = 1−*x*, *y*, 1−*z*.

Crystal data for (*S,S*)-cycHC[8] · **7**, $\{$**[(*S,S*)-cycHC[8] · MgTPP]**$\}^{1}_{\infty}$ · **CH_2Cl_2 (+solvate)**: CCDC-2444112, $C_{109}H_{126}Cl_2MgN_{20}O_8$, M = 1939.50 gmol^{-1}, purple plate, 0.36 × 0.08 × 0.03 mm^3, tetragonal, space group $P4_322$ (No. 95), a = 14.0038(2) Å, b = 14.0038(2) Å, c = 60.6508(8) Å, α = 90°, β = 90°, γ = 90°, V = 11894.0(4) Å^3, Z = 4, D_{calc} = 1.083 gcm^{-3}, F(000) = 4120, μ = 1.004 mm^{-1}, T = 120(2) K, θ_{max} = 74.96°, 88846 total reflections, 7974 with $I_o > 2\sigma(I_o)$, R_{int} = 0.0511, 11269 data, 634 parameters, 1 restraints, GooF = 1.076, R_1 = 0.0802 and wR_2 = 0.2030 [$I_o > 2\sigma(I_o)$], R_1 = 0.0975 and wR_2 = 0.2105 (all reflections), 1.030 < dΔρ < -0.927 eÅ^{-3}, Flack parameter x = 0.147(10).

(-*S,S*)-cycHC[8] (6.1 mg, 0.005 mmol, 1 eq.) was dissolved in DCM/MeOH (*v*:*v*, 1:1, 1 mL), and added to a purple solution of MgTPP (3.2 mg, 0.005 mmol, 1 eq.) in DCM (0.5 mL). Crystals of (*S,S*)-cycHC[8] · **7** were obtained after 3 days *via* slow evaporation at 3 °C. The asymmetric unit consists of a monomeric unit of one-dimensional, twisted chain coordination polymer of alternating (*S,S*)-cycHC[8] macrocycles and MgTPP, one co-crystallized DCM molecule, and other unknown solvates. The model required a single DFIX restraint to adequately model the hydroxyl group of a methanol molecule. In the final refinement cycles, an isotropic extinction parameter according to Larson converged to a value of 0.00058(7).[17] No sensible disordered model could be formulated for the unknown solvates which would match the observed electron density, so the computer program SQUEEZE[15] within PLATON[16] was used to account for the electron density in this region of the unit cell. The program identified solvent accessible voids totaling 2967 Å^3 and 39 electrons per unit cell were recovered. The formula weight, density, etc. listed above do not include any correction for the missing solvate.

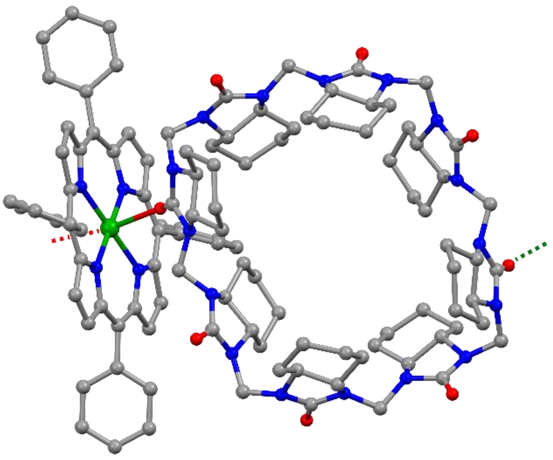

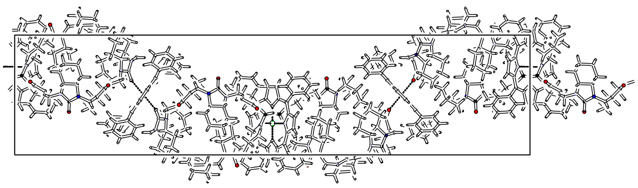

Figure S115 Ball-and-stick-model of (*S,S*)-cycHC[8] · **7**. Hydrogen atoms as well as co-crystallized solvent molecules have been omitted for clarity. 1D polymeric strand along [1 0 0] direction. Selected interatomic distances (Å): Mg1···O1 2.263(4), Mg1···O1^a 2.263(4). Symmetry operator: a = 1−*x*, *y*, 1−*z*.

6. Computational analysis

For all the computations in this work single-point energy calculations were performed with the ORCA[18–21] program package using the B3LYP functional and the def2-SVP basis set. The crystallographic coordinates were used as input geometries without further optimization. Tight SCF convergence criteria and ORCA's Grid5 integration grid were employed.

Multiwfn[22,23] was used to perform quantitative molecular surface analysis for electrostatic potential surface coloring,[24] noncovalent interactions (NCI, a.k.a reduced density gradient) analysis[25] and Interaction region indicator (IRI) analysis[26]. VMD[27] software was used for visualization of the NCI and IRI color-filled isosurface graphs. Gnuplot program (http://www.gnuplot.info) was used for generating color mapped NCI and IRI scatter maps.

6.1 Electrostatic potential colored molecular van der Waals surface

All the figures in this paragraph are from the same complex – (*S*,*S*)-cycHC[8]·**7** – only varying the shown isosurfeces and their texture. In all cases, RGB coloring method was used (lower and upper limits of color scale are -50 and 50 kcal/mol), where red means negative ESP, green means almost no ESP and blue means positive ESP.

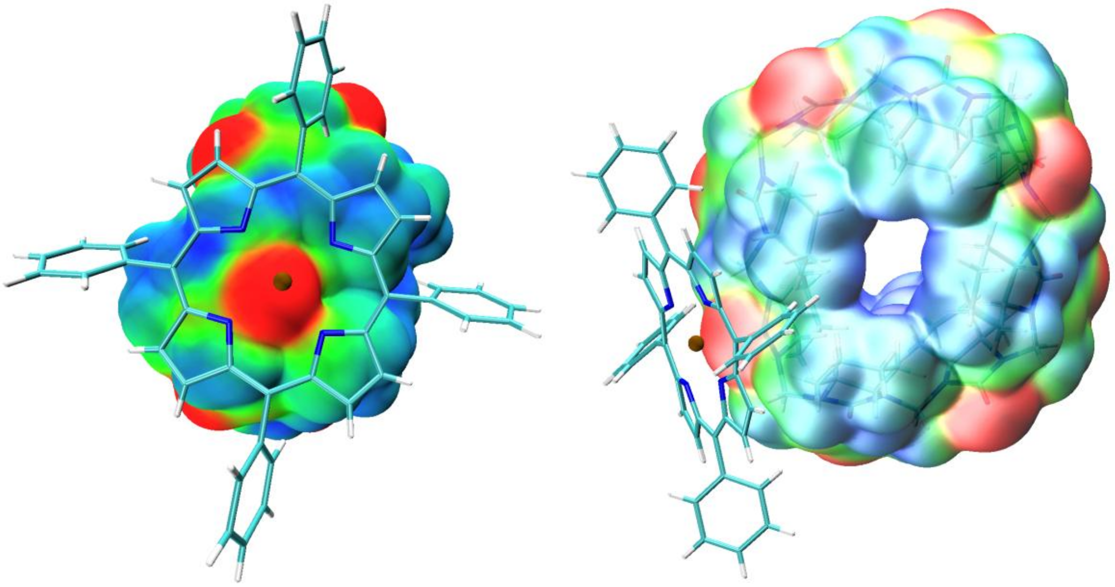

Figure S116 Strucure of **7** is shown in licorice method, while (*S*,*S*)-cycHC[8] is shown as ESP surface.

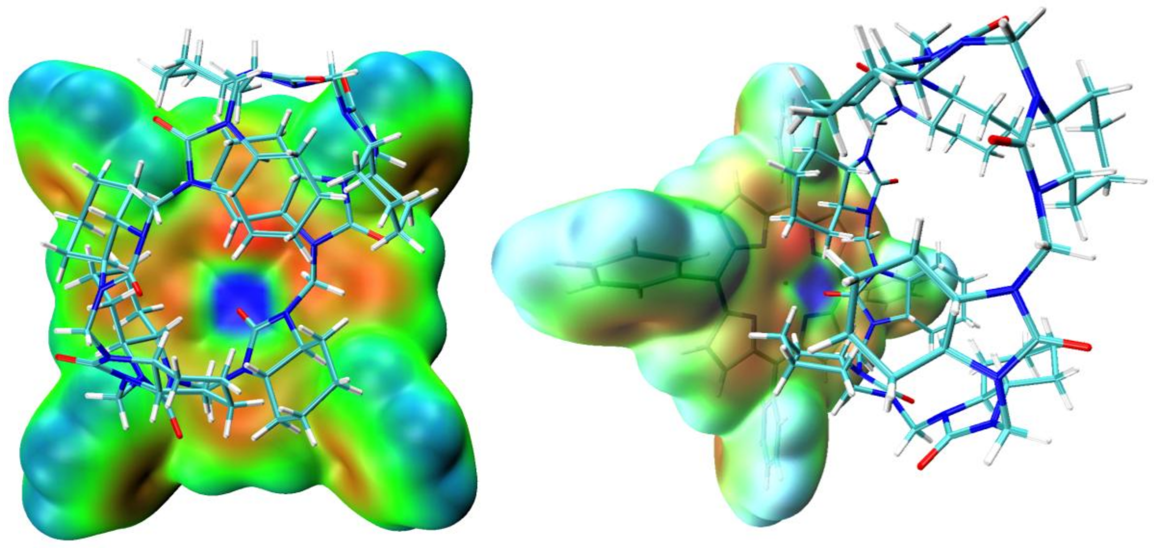

Figure S117 Strucure of (*S*,*S*)-cycHC[8] is shown in licorice method in the front, while **7** is shown as ESP surface in the back.

6.2 Noncovalent interactions analysis

With the simple instructions of Multiwfn software we were able to visualize noncovalent interactions between cycHC[*n*]'s and porphyrins based on reduced density gradient (RDG):

$$RDG(r) = \frac{1}{2(3\pi^2)^{1/3}} \frac{|\nabla\rho(r)|}{\rho(r)^{4/3}} \tag{2}$$

where ρ is electron density and r is coordinate vector.[22,23,25] Another important function for NCI is $sign(\lambda_2)\rho$ - product of the sign of the second largest eigenvalue of electron density Hessian.[22,23] Coloring used in all the NCI and IRI graphs is shown in **Figure S118**. Spatial ranges of grid data were selected such that the porphyrin and nearby part of macrocycle would be included and grid spacing was selected as 0.15 Bohr.

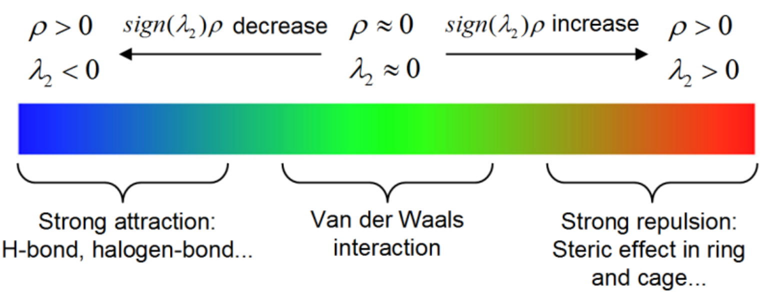

Figure S118 Labelled color bar for used colors in NCI isosurface graphs and scatter maps (provided by Multiwfn[22,23]).

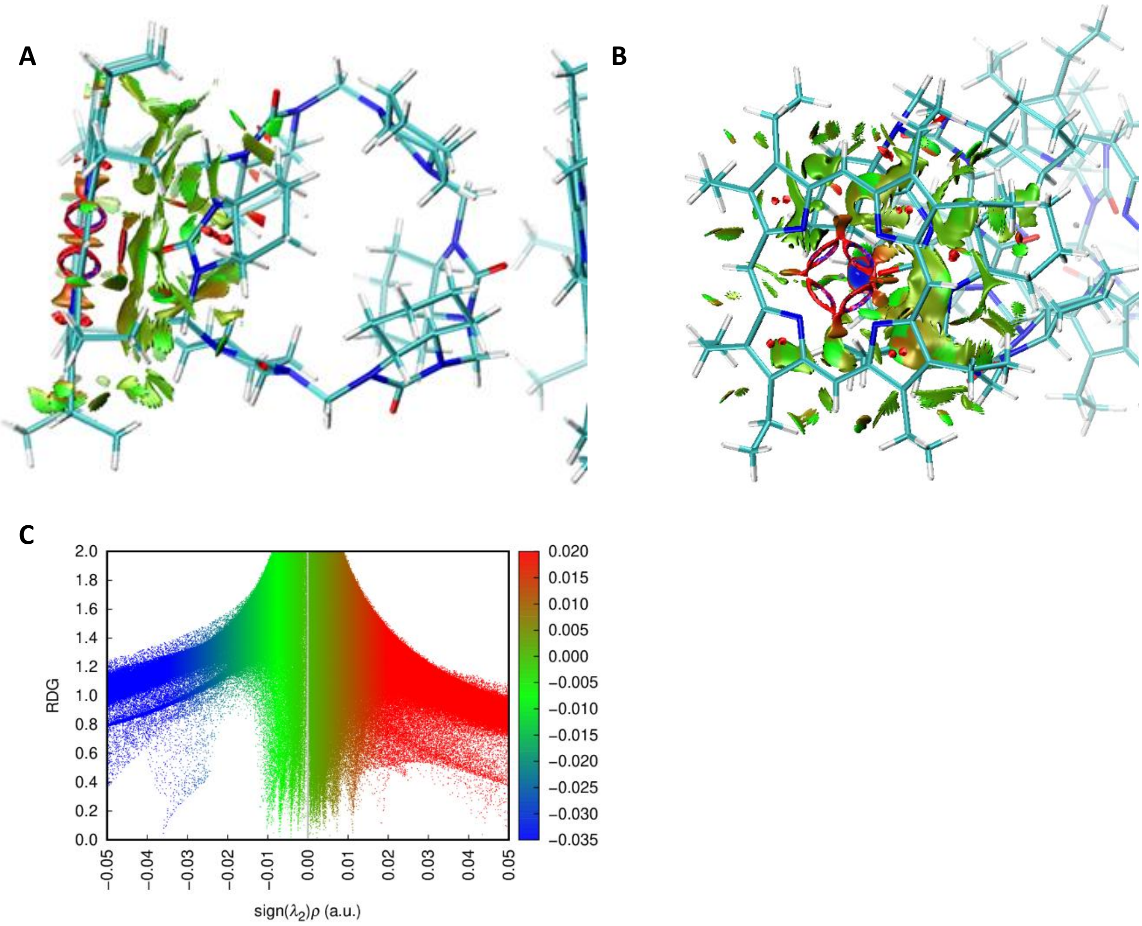

Figure S119 RDG isosurface maps of (*R*,*R*)-cycHC[6]·**1**, where grid data is selected between (*R*,*R*)-cycHC[6] and penta-coordinated peripheral porphyrin. A) view from the side, B) same map viewed diagonally, C) scatter graph

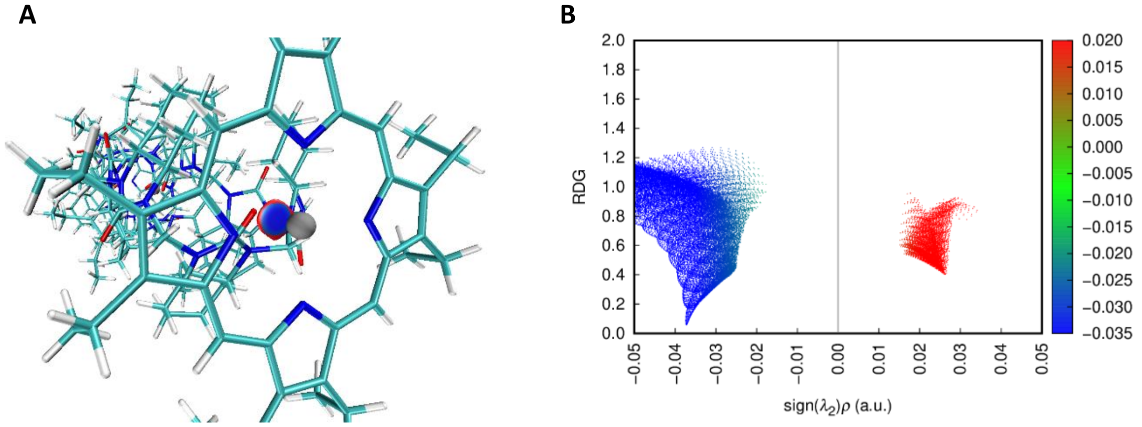

Figure S120 A) RDG isosurface map of (*R*,*R*)-cycHC[6]·**1**, where grid data is selected between the carbonyl oxygen of (*R*,*R*)-cycHC[6] and penta-coordinated zinc between the peripheral porphyrin **1**. B) corresponding scatter graph

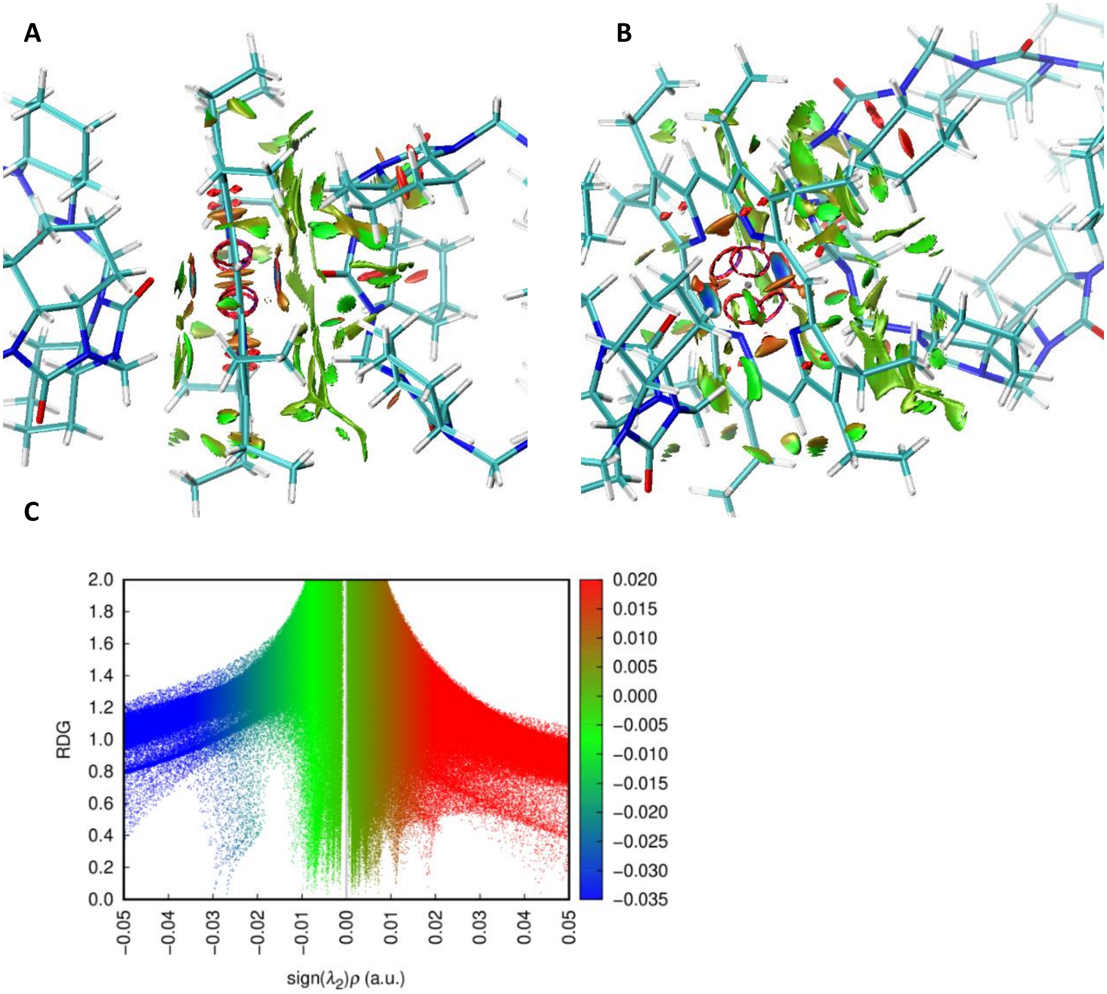

Figure S121 RDG isosurface maps of (R,R)-cycHC[6]·**1**, where grid data is selected between (R,R)-cycHC[6] and hexa-coordinated sandwiched porphyrin in the middle of two cycHC's. A) view from the side, B) same map viewed diagonally, C) scatter graph

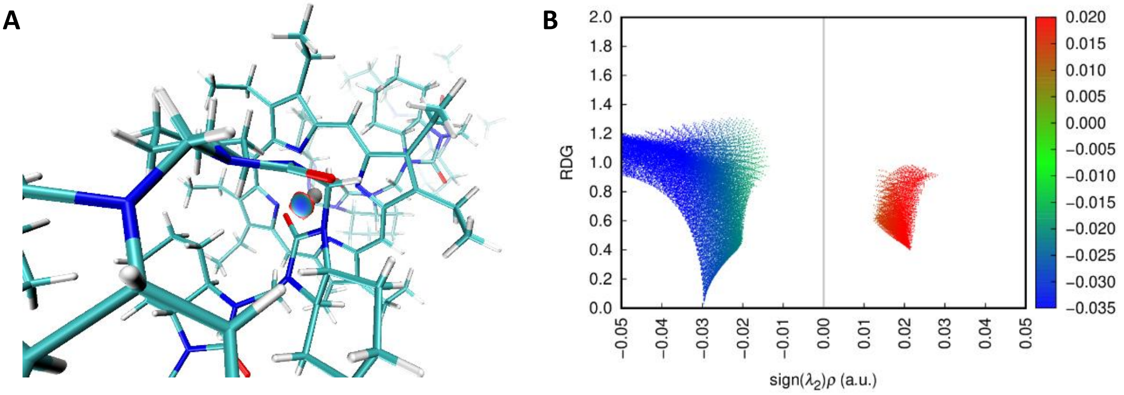

Figure S122 A) RDG isosurface map of (R,R)-cycHC[6]·**1**, where grid data is selected between the carbonyl oxygen of (R,R)-cycHC[6] and hexa-coordinated zinc between the sandwiched porphyrin **1**. B) corresponding scatter graph

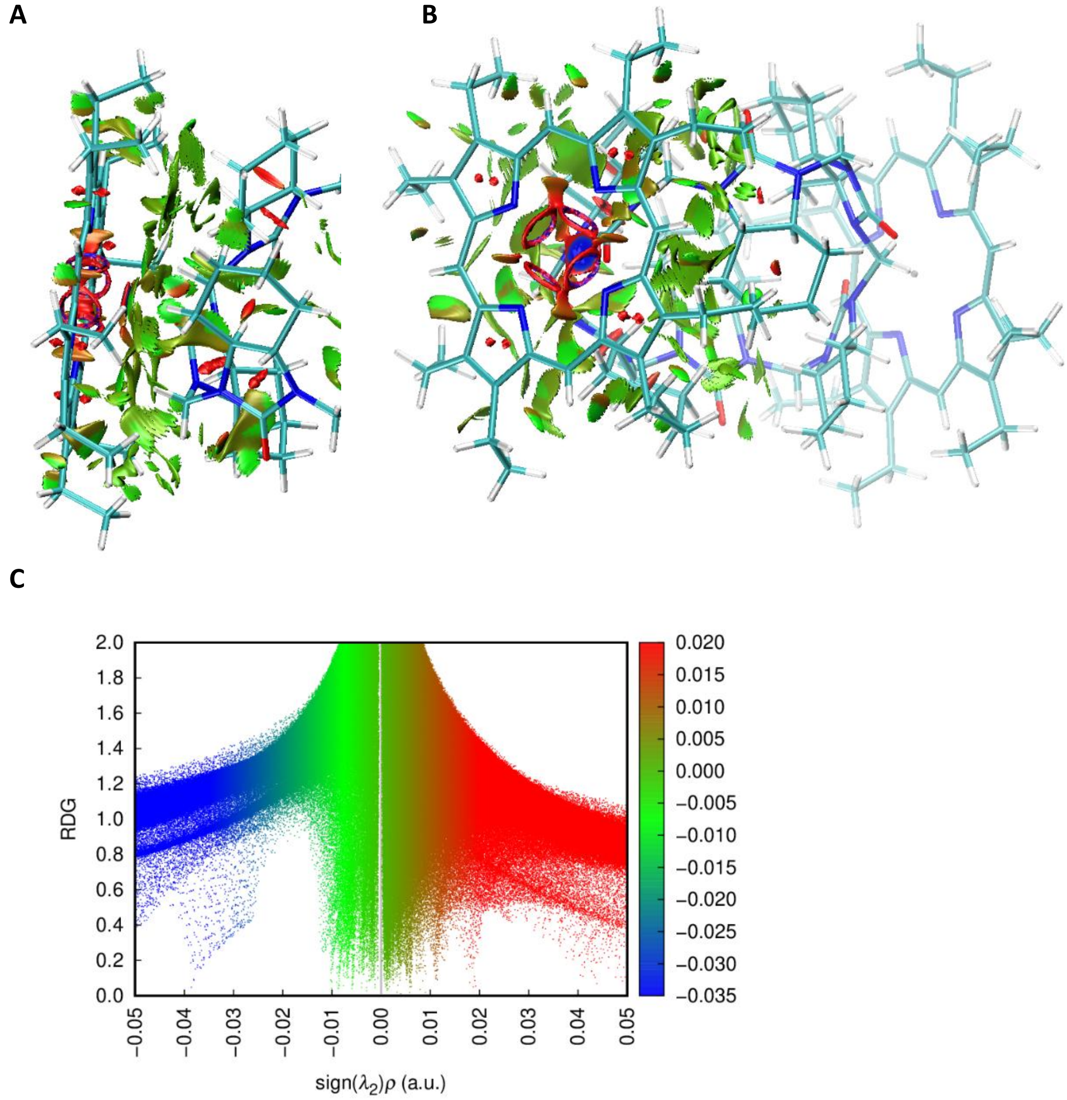

Figure S123 RDG isosurface maps of (*S*,*S*)-cycHC[6]·**1**, where grid data is selected between (*S*,*S*)-cycHC[6] and penta-coordinated peripheral porphyrin. A) view from the side, B) same map viewed diagonally, C) scatter graph

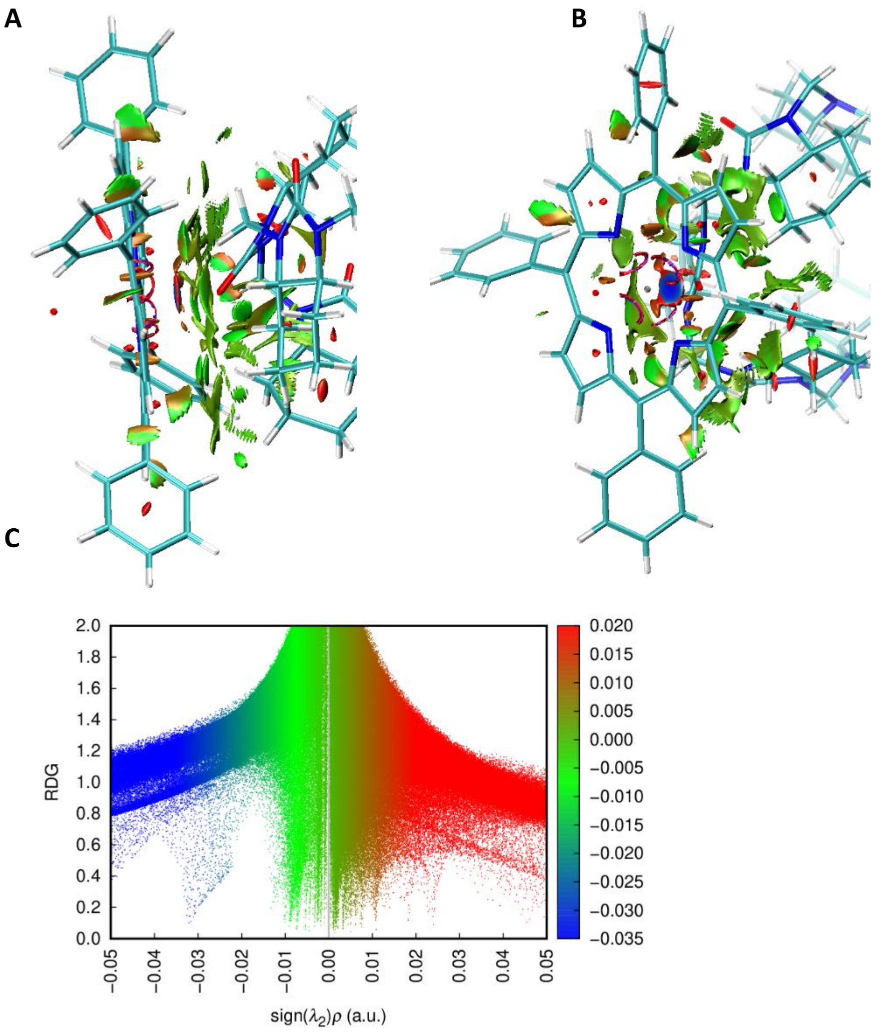

Figure S124 RDG isosurface maps of (*S*,*S*)-cycHC[6]·**2**, where grid data is selected between (*S*,*S*)-cycHC[6] and porphyrin **2**. A) view from the side, B) same map viewed diagonally, C) scatter graph

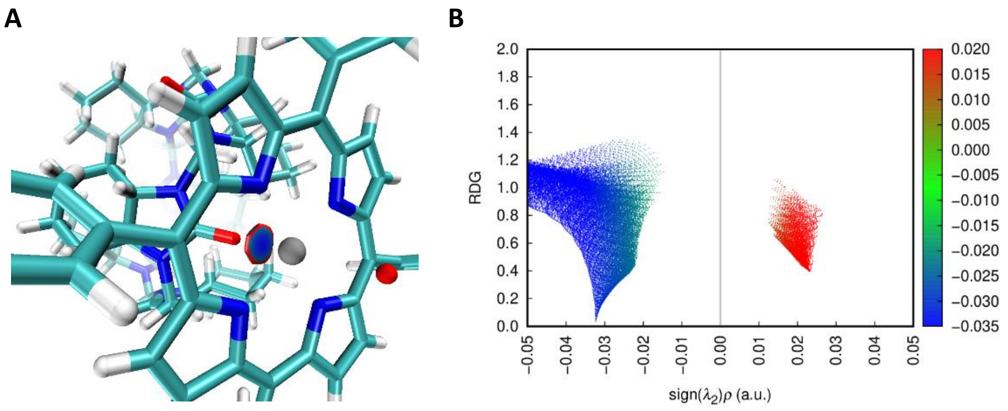

Figure S125 A) RDG isosurface map of (*S*,*S*)-cycHC[6]·**2**, where grid data is selected between the carbonyl oxygen of (*S*,*S*)-cycHC[6] and hexa-coordinated zinc between porphyrin **2**. B) corresponding scatter graph

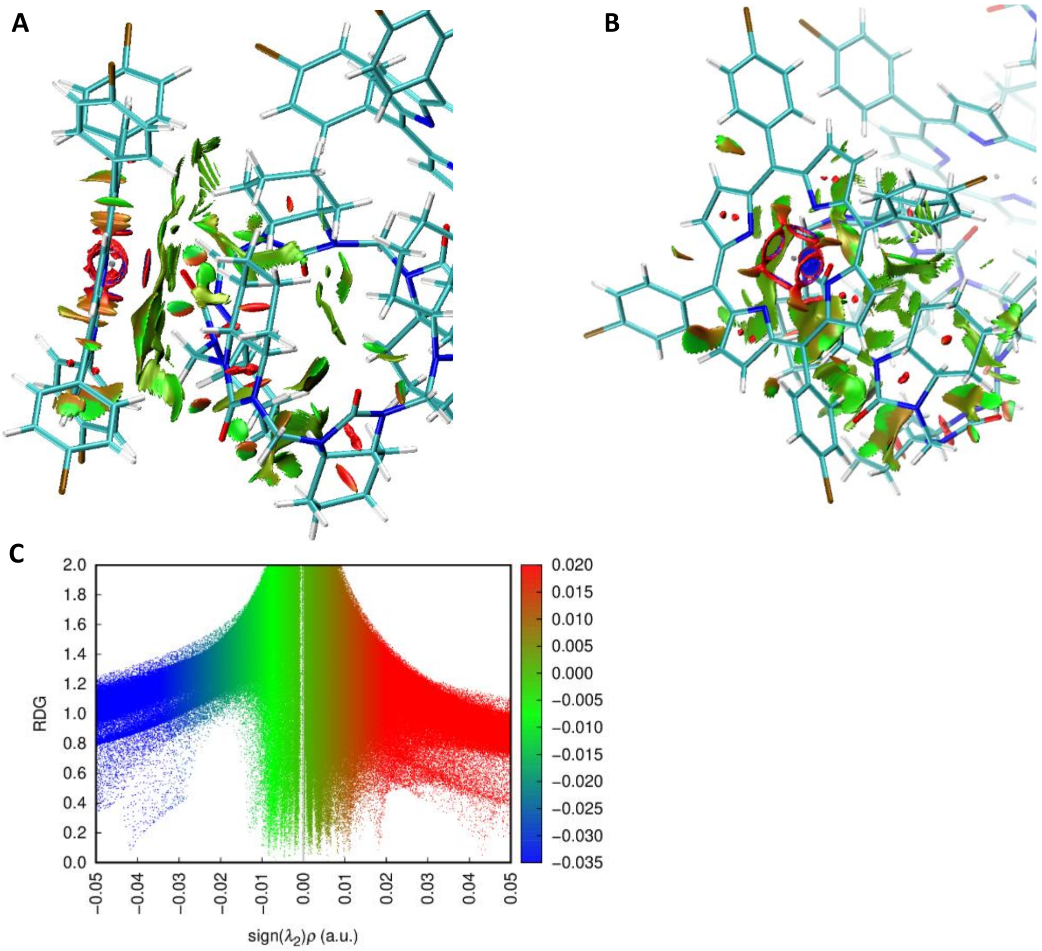

Figure S126 RDG isosurface maps of (*R*,*R*)-cycHC[6]·**4**, where grid data is selected between (*R*,*R*)-cycHC[6] and peripheral porphyrin **4**. A) view from the side, B) same map viewed diagonally, C) scatter graph

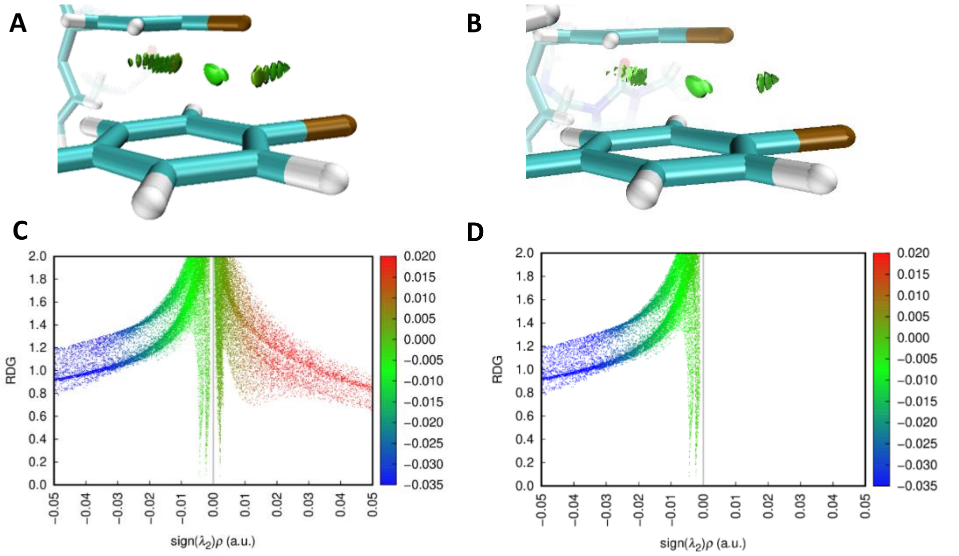

Figure S127 RDG isosurface maps of (*R*,*R*)-cycHC[6]·**4**, where grid data is selected between chloroaryls of the neighbouring porphyrins **4**. A) full range, B) only negative *sign*(λ_2)ρ values, C) scatter graph of A, D) scatter graph of B

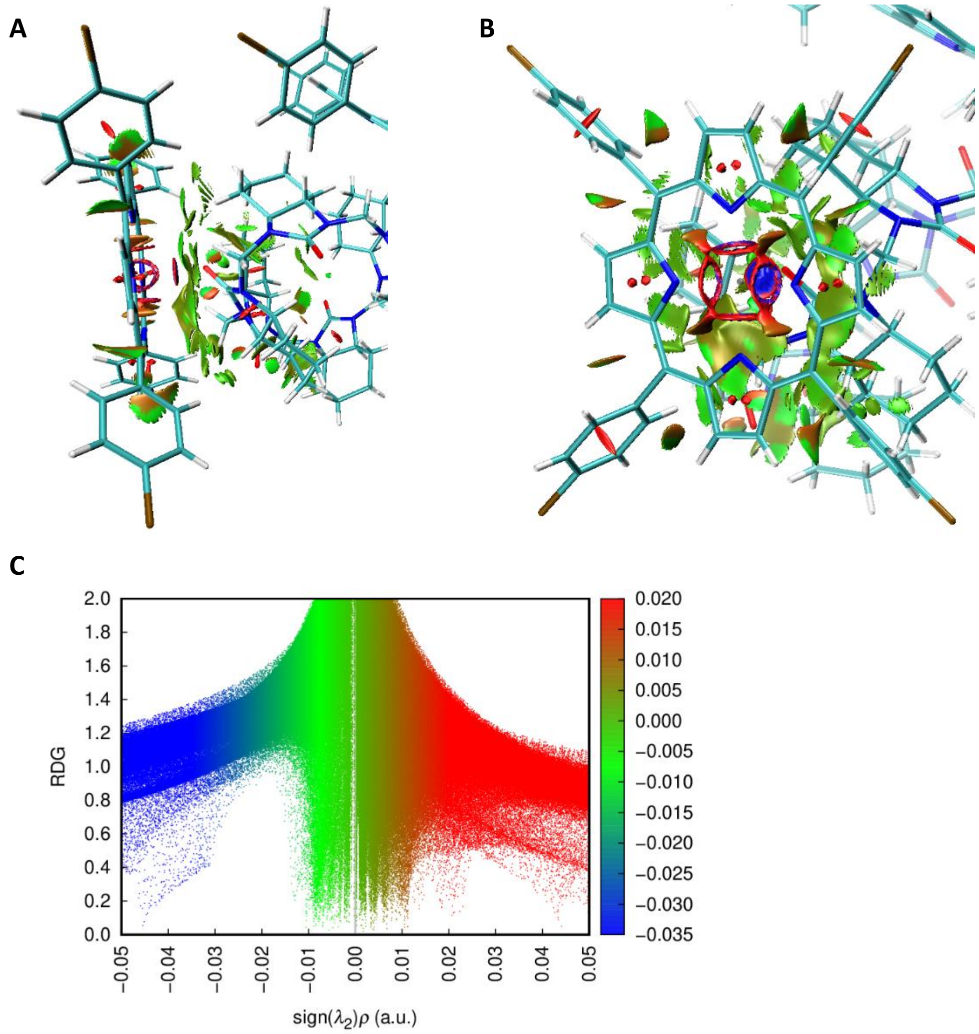

Figure S128 RDG isosurface maps of (*S*,*S*)-cycHC[6]·**4**, where grid data is selected between (*S*,*S*)-cycHC[6] and peripheral porphyrin **4**. A) view from the side, B) same map viewed diagonally, C) scatter graph

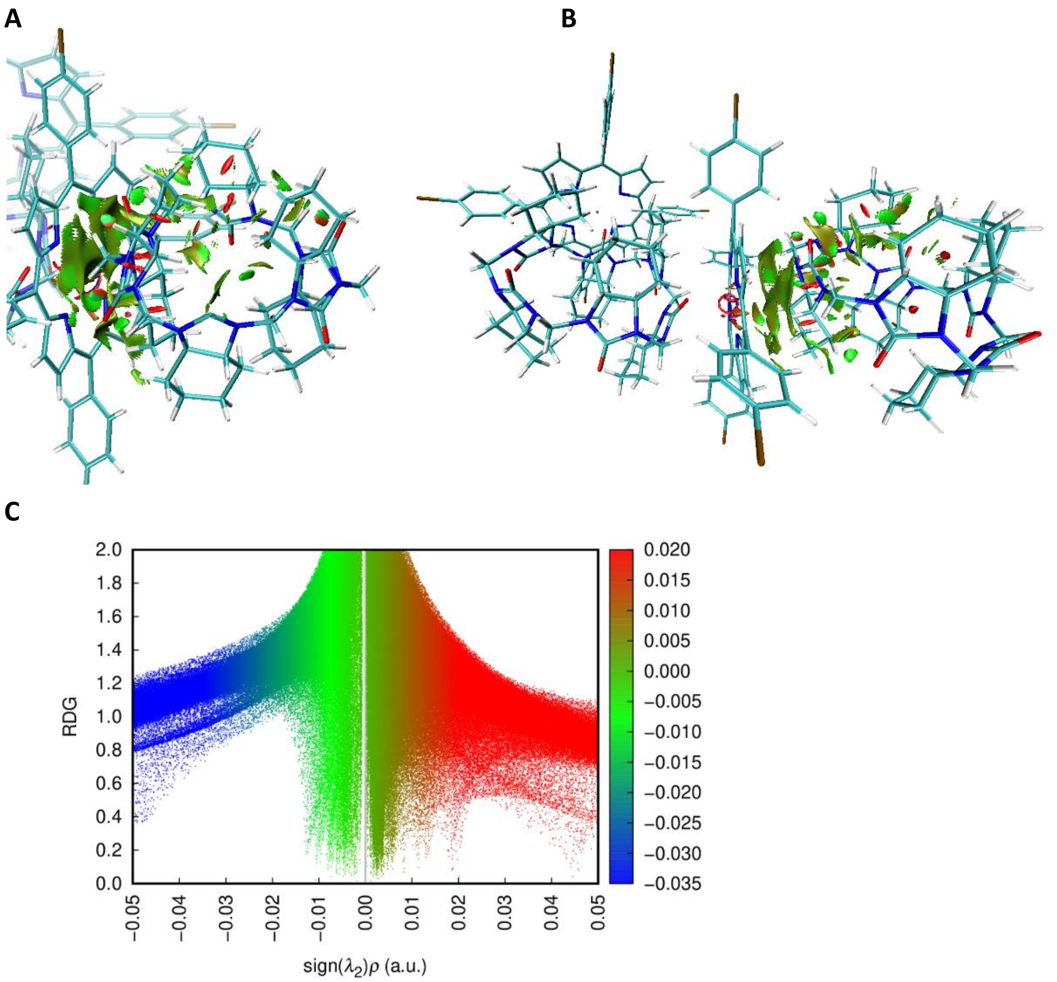

Figure S129 RDG isosurface maps of (*S*,*S*)-cycHC[6]·**4**, where grid data is selected between complex porphyrin **4** and uncomplexed (*S*,*S*)-cycHC[6] nearby. A) diagonal view, B) same map viewed from the side, C) scatter graph

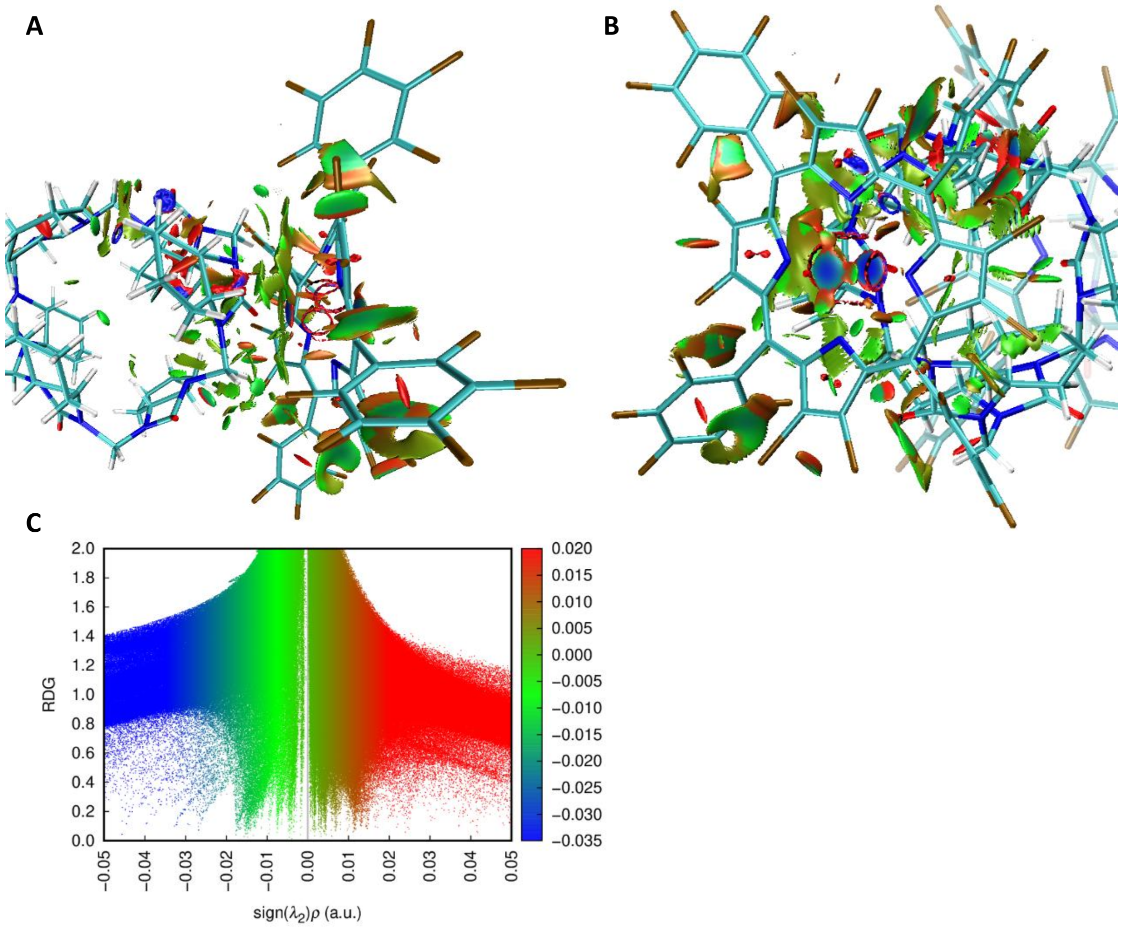

Figure S130 RDG isosurface maps of (*R*,*R*)-cycHC[6]·**6**, where grid data is selected between (*R*,*R*)-cycHC[6] and porphyrin **6**. A) view from the side, B) same map viewed diagonally, C) scatter graph

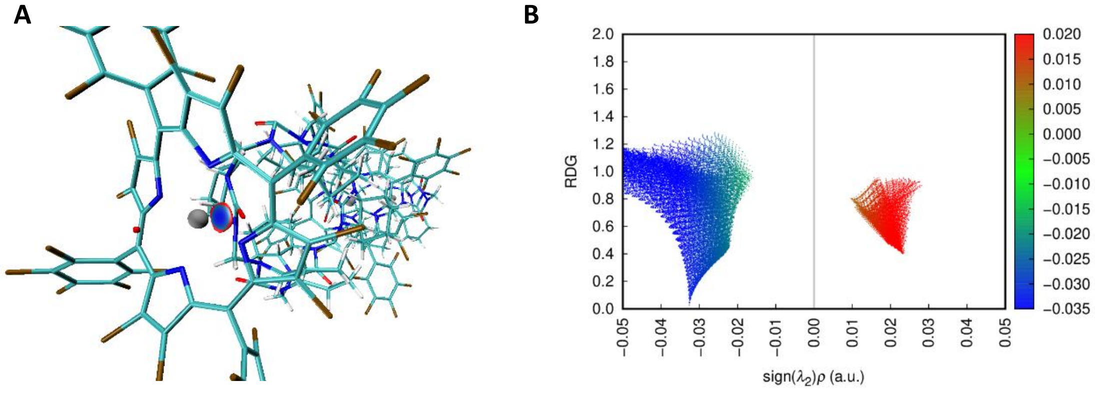

Figure S131 A) RDG isosurface map of (*R*,*R*)-cycHC[6]·**6**, where grid data is selected between the carbonyl oxygen of (*R*,*R*)-cycHC[6] and hexa-coordinated zinc between porphyrin **6**. B) corresponding scatter graph

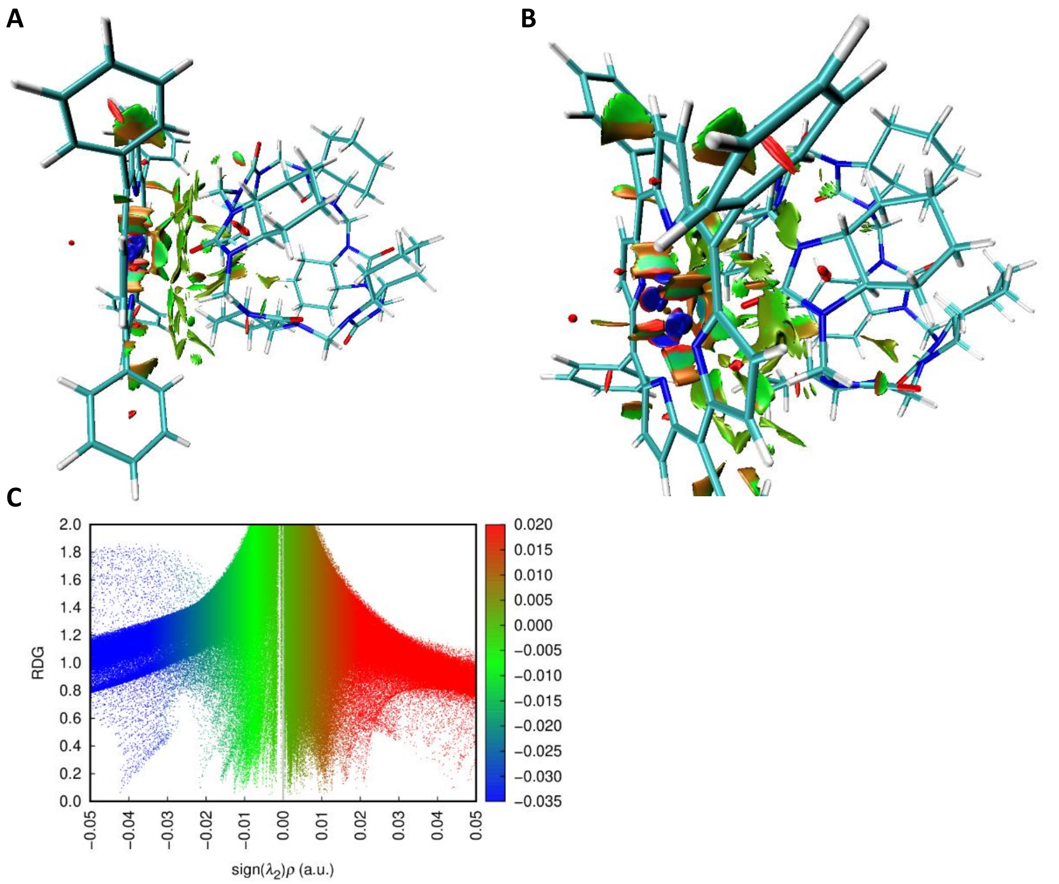

Figure S132 RDG isosurface maps of (R,R)-cycHC[6]·**7**, where grid data is selected between (R,R)-cycHC[6] and porphyrin **7**. A) view from the side, B) same map viewed diagonally, C) scatter graph

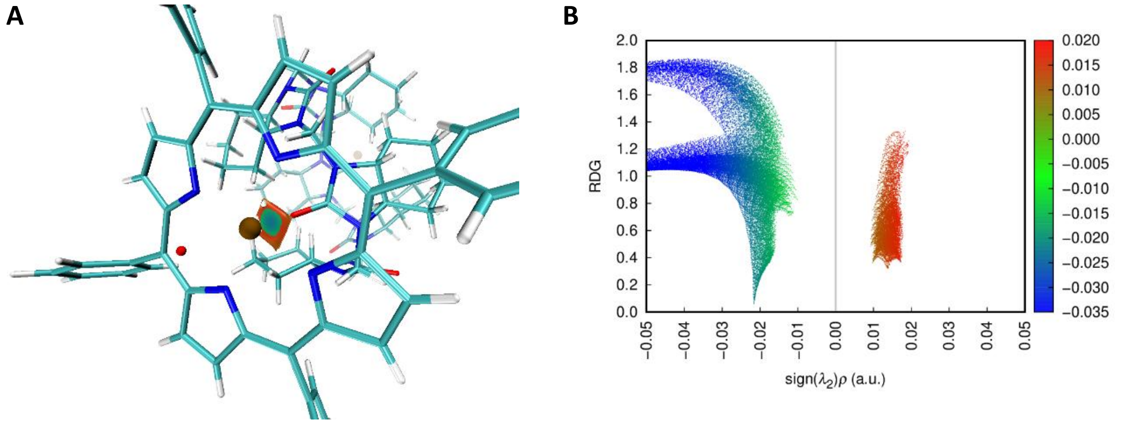

Figure S133 A) RDG isosurface map of (R,R)-cycHC[6]·**7**, where grid data is selected between the carbonyl oxygen of (R,R)-cycHC[6] and hexa-coordinated magnesium between porphyrin **7**. B) corresponding scatter graph

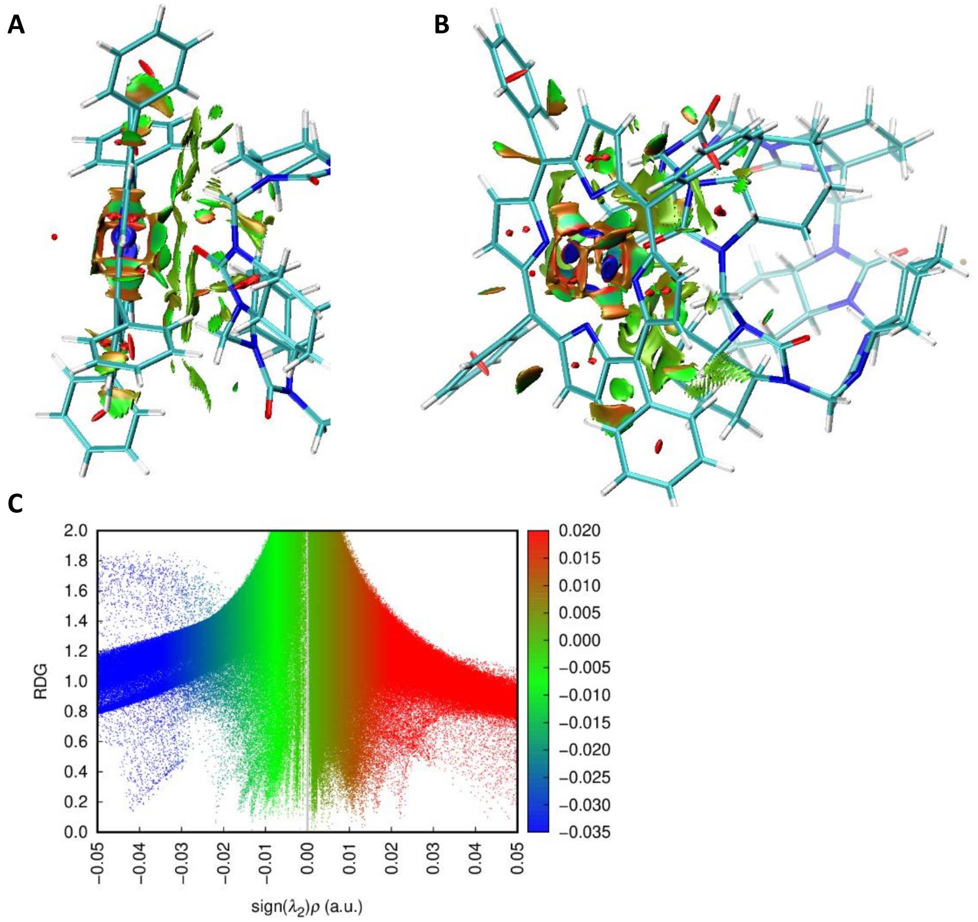

Figure S134 RDG isosurface maps of (*S*,*S*)-cycHC[6]·**7**, where grid data is selected between (*S*,*S*)-cycHC[6] and porphyrin **7**. A) view from the side, B) same map viewed diagonally, C) scatter graph

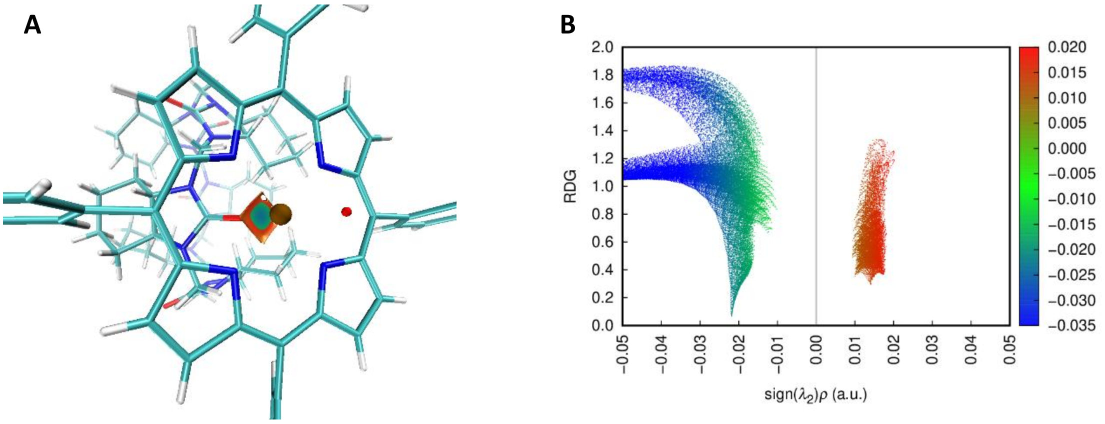

Figure S135 A) RDG isosurface map of (*S*,*S*)-cycHC[6]·**7**, where grid data is selected between the carbonyl oxygen of (*S*,*S*)-cycHC[6] and hexa-coordinated magnesium between porphyrin **7**. B) corresponding scatter graph

A B C

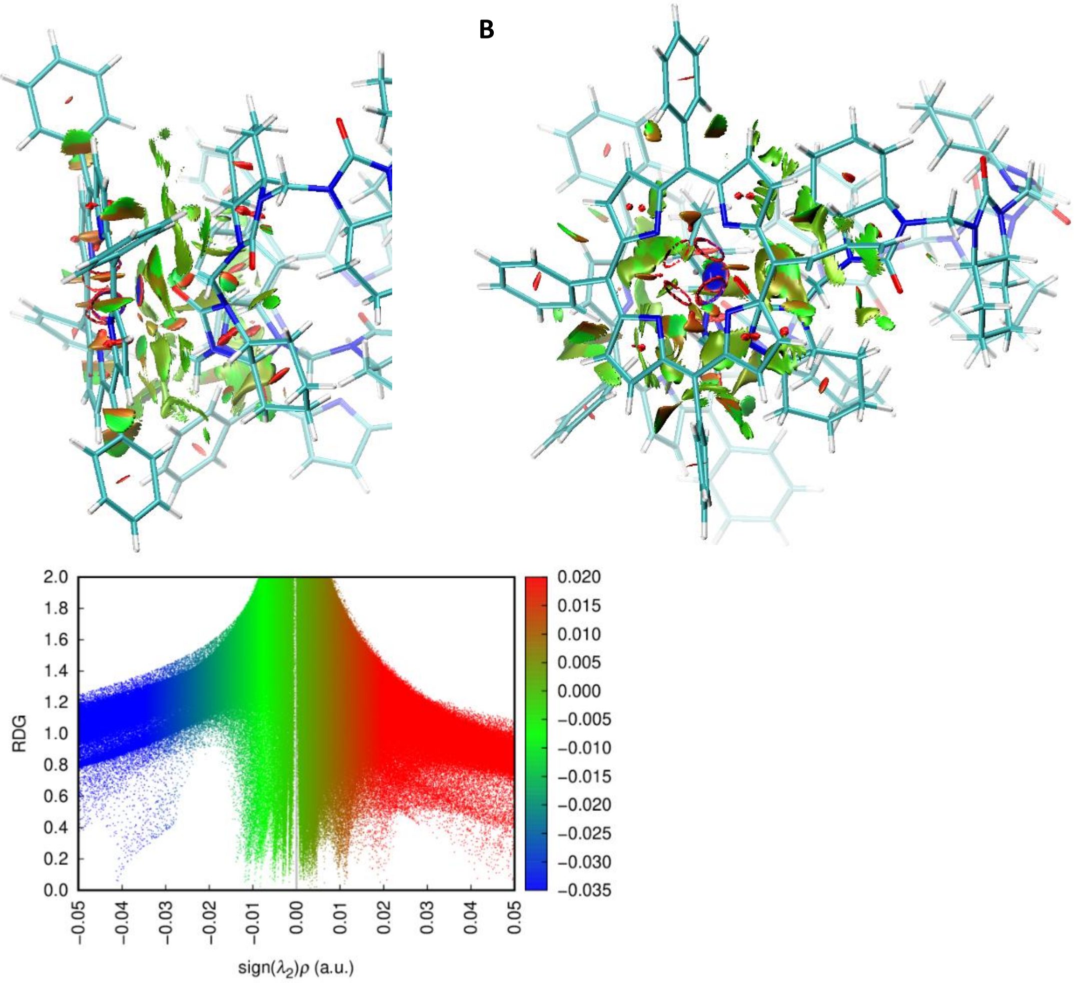

Figure S136 RDG isosurface maps of (*R*,*R*)-cycHC[8]·**2(a)**, where grid data is selected between (*R*,*R*)-cycHC[8] and peripheral porphyrin **2(a)**. A) view from the side, B) same map viewed diagonally, C) scatter graph

A B

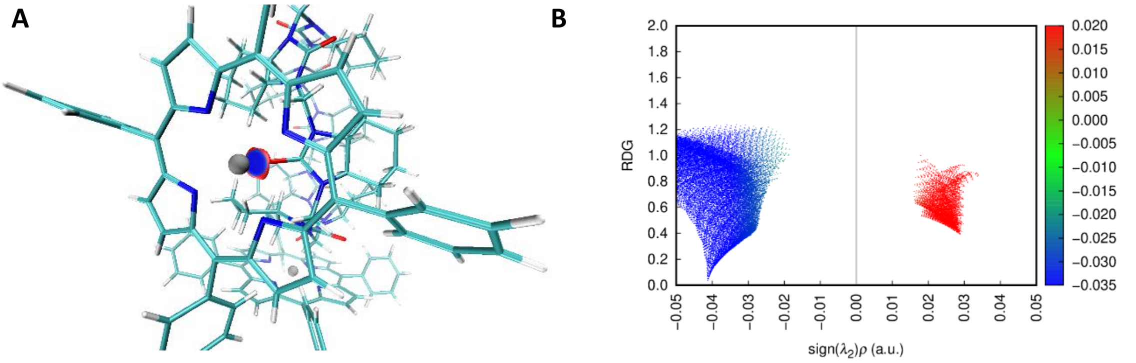

Figure S137 A) RDG isosurface map of (*R*,*R*)-cycHC[8]·**2(a)**, where grid data is selected between the carbonyl oxygen of (*R*,*R*)-cycHC[8] and penta-coordinated zinc between porphyrin **2**. B) corresponding scatter graph

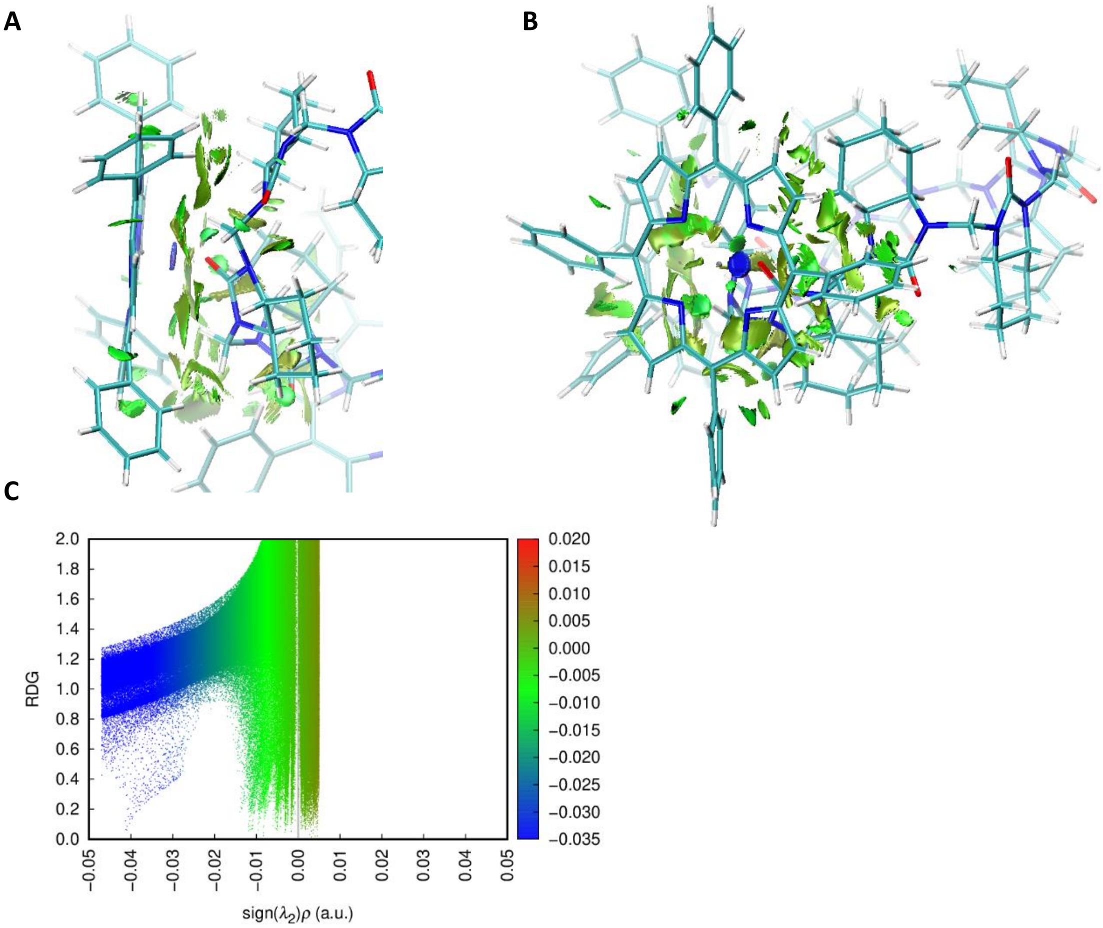

Figure S138 RDG isosurface maps of (*R*,*R*)-cycHC[8]·**2(a)** (same as **Figure S136** but with repulsive interactions removed). A) view from the side, B) same map viewed diagonally, C) scatter graph

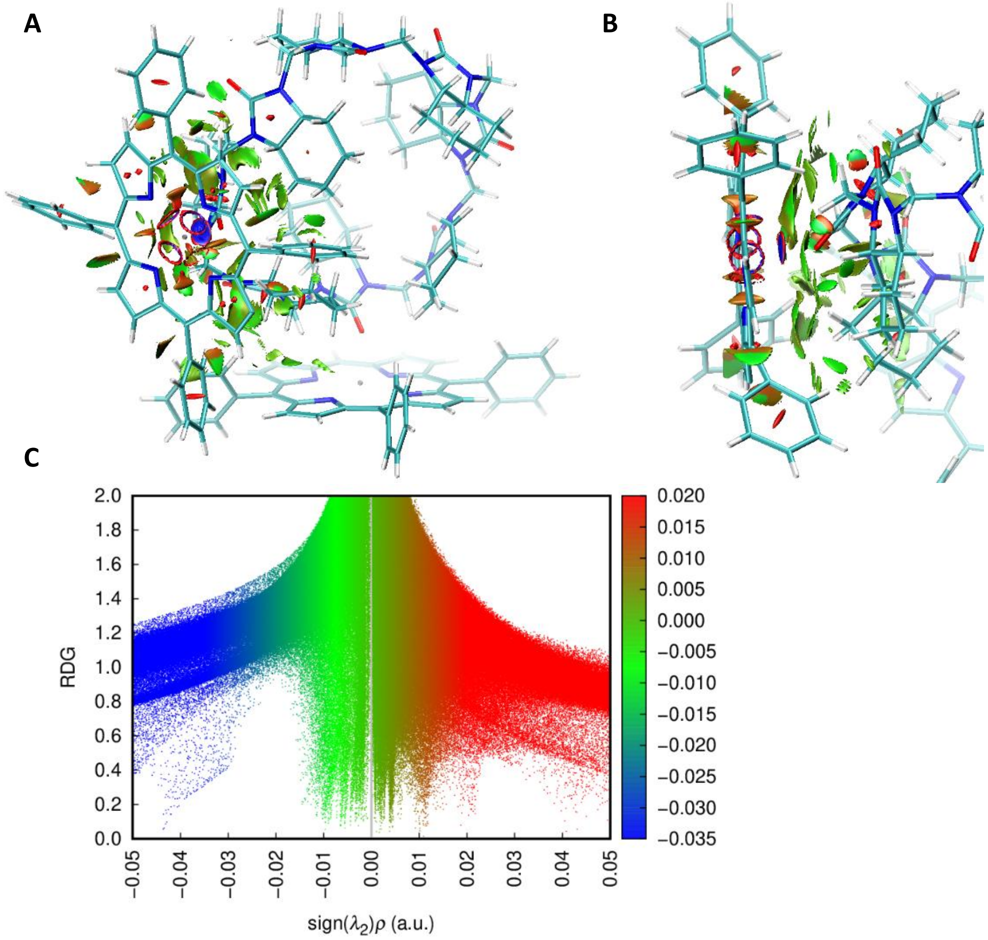

Figure S139 RDG isosurface maps of (*S*,*S*)-cycHC[8]·**2(a)**, where grid data is selected between (*S*,*S*)-cycHC[8] and porphyrin **2(a)**. A) diagonal view, B) same map viewed from the side, C) scatter graph

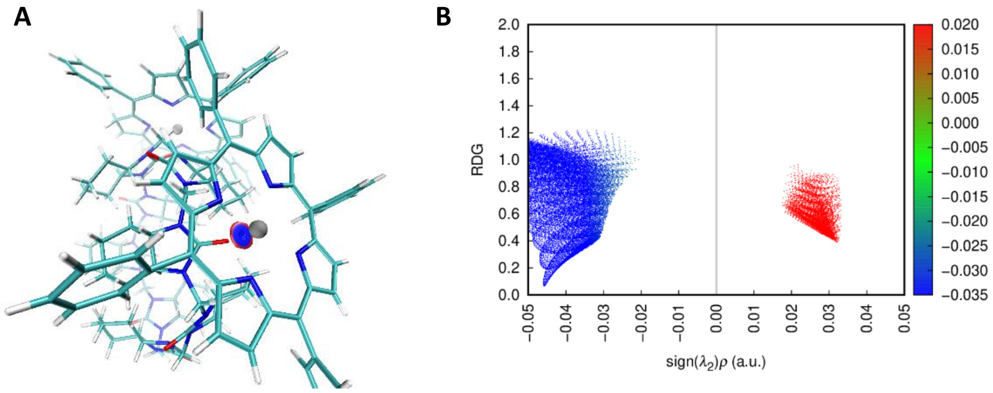

Figure S140 A) RDG isosurface map of (*S*,*S*)-cycHC[8]·**2(a)**, where grid data is selected between the carbonyl oxygen of (*S*,*S*)-cycHC[8] and penta-coordinated zinc between porphyrin **2**. B) corresponding scatter graph

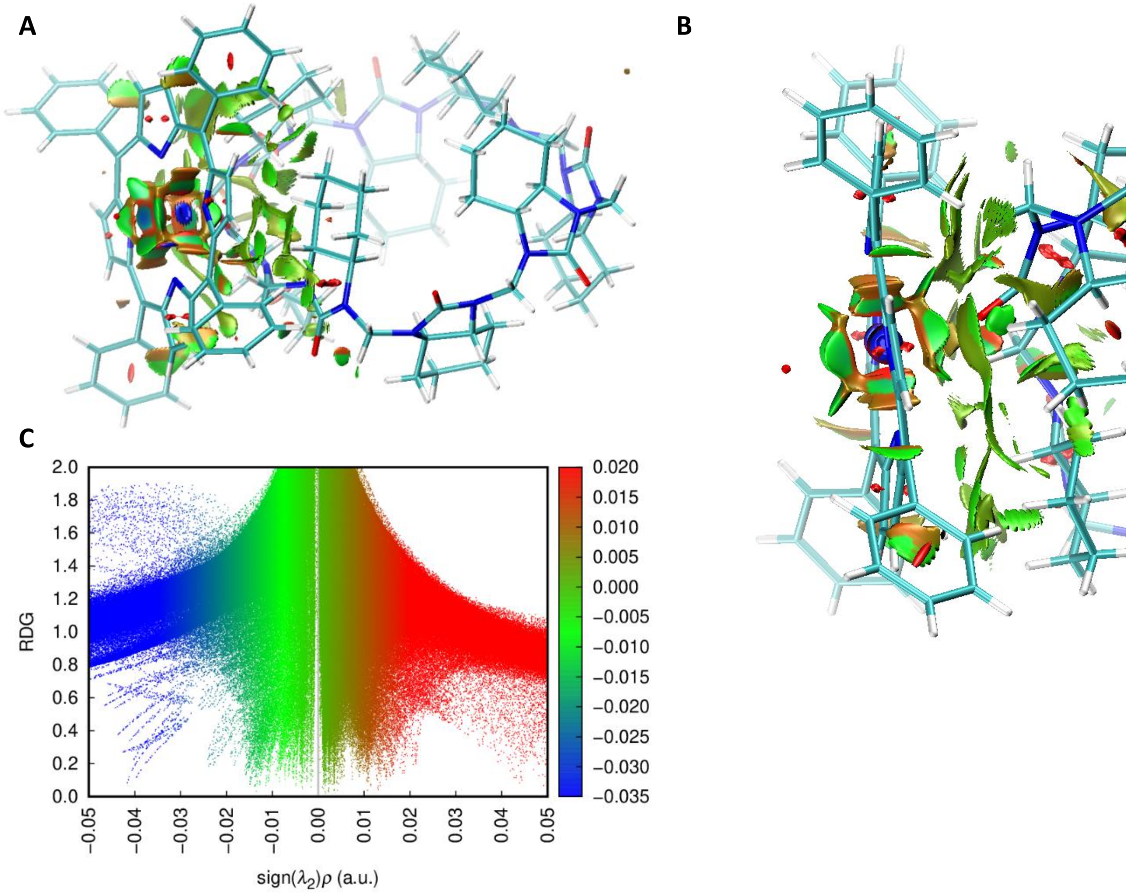

Figure S141 RDG isosurface maps of (*R*,*R*)-cycHC[8]·**7**, where grid data is selected between (*R*,*R*)-cycHC[8] and porphyrin **7**. A) diagonal view, B) same map viewed from the side, C) scatter graph

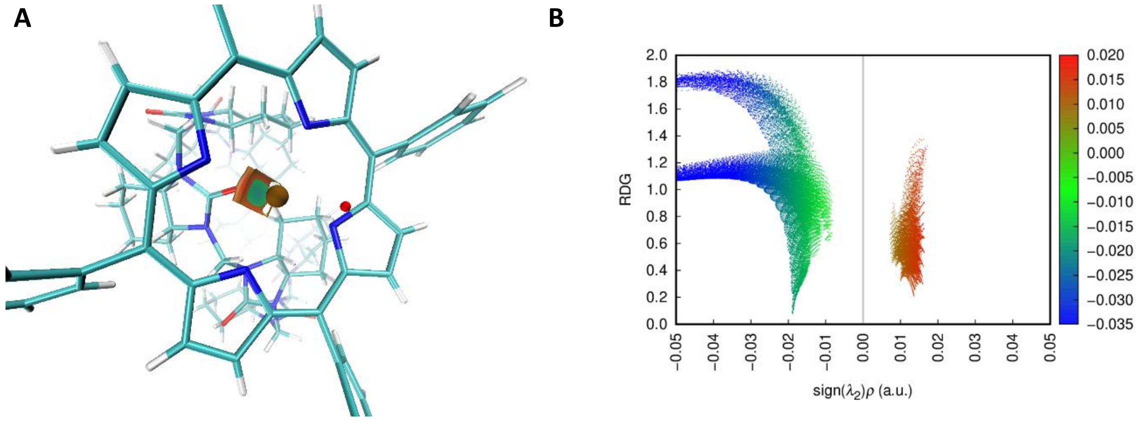

Figure S142 A) RDG isosurface map of (*R*,*R*)-cycHC[8]·**7**, where grid data is selected between the carbonyl oxygen of (*R*,*R*)-cycHC[8] and hexa-coordinated magnesium between porphyrin **7**. B) corresponding scatter graph

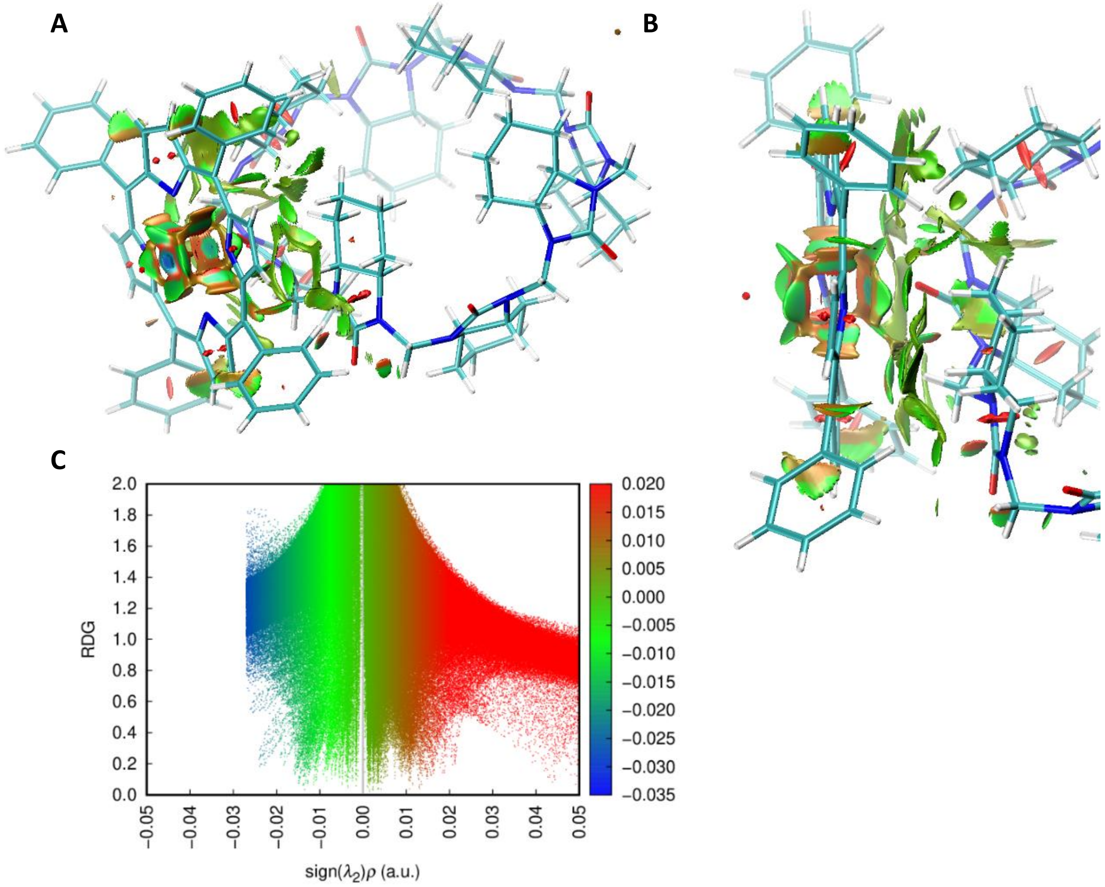

Figure S143 RDG isosurface maps of (*R*,*R*)-cycHC[8]·**7** (same as **Figure S141** but with -0.5 to -0.27 region removed, region corresponds to porphyrin Mg-N interactions). A) diagonal view, B) same map viewed from the side, C) scatter graph

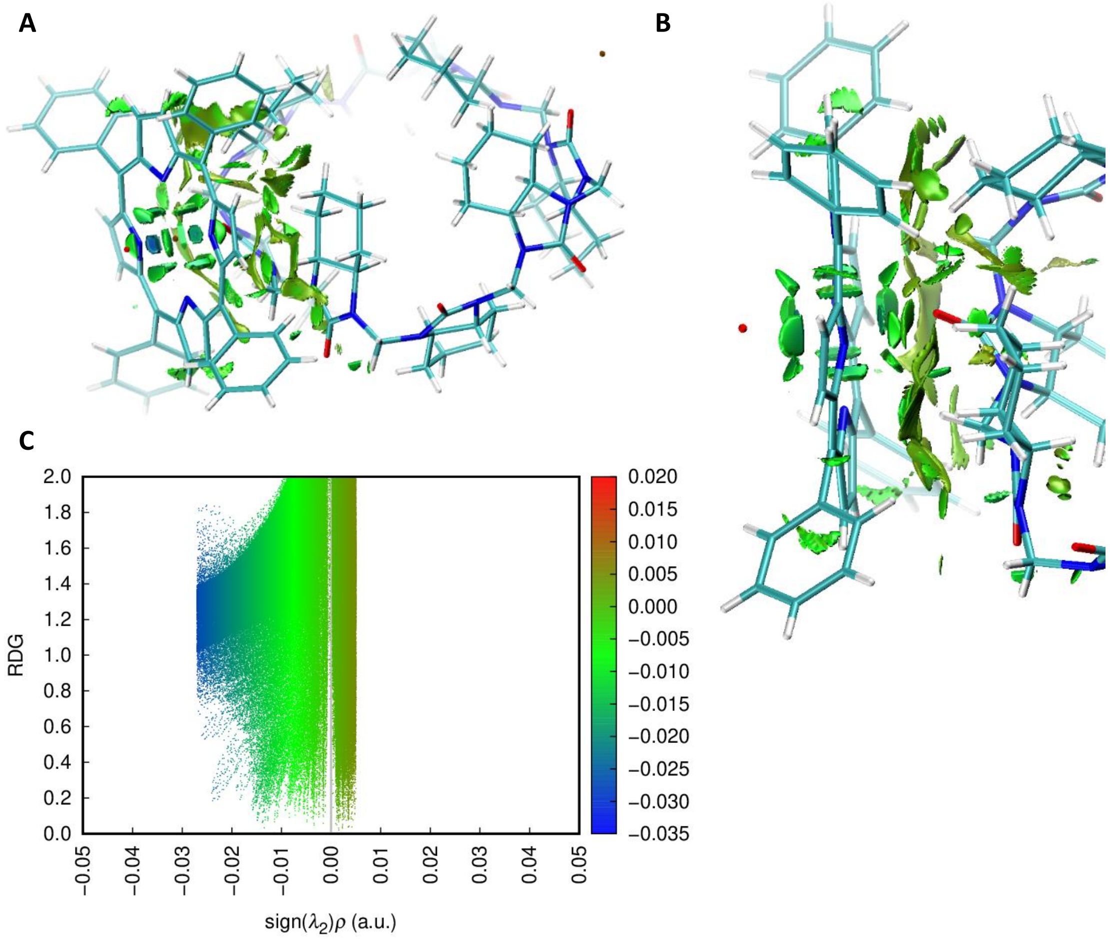

Figure S144 RDG isosurface maps of (*R*,*R*)-cycHC[8]·**7** (same as **Figure S143** but additionally also repulsive force region is removed). A) diagonal view, B) same map viewed from the side, C) scatter graph

A

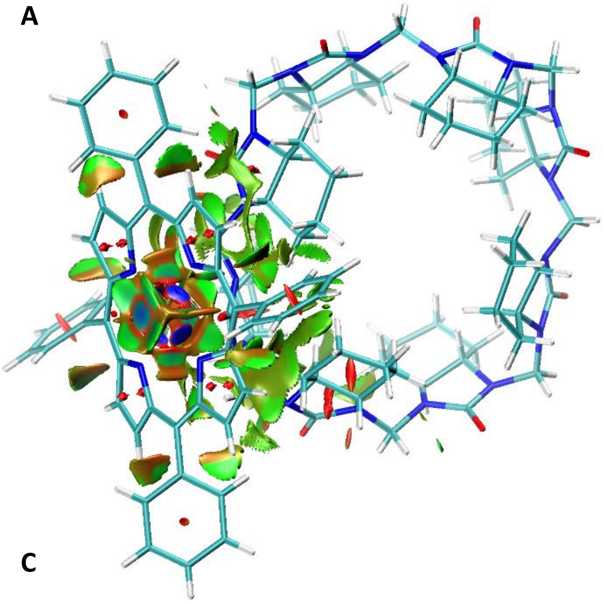

B

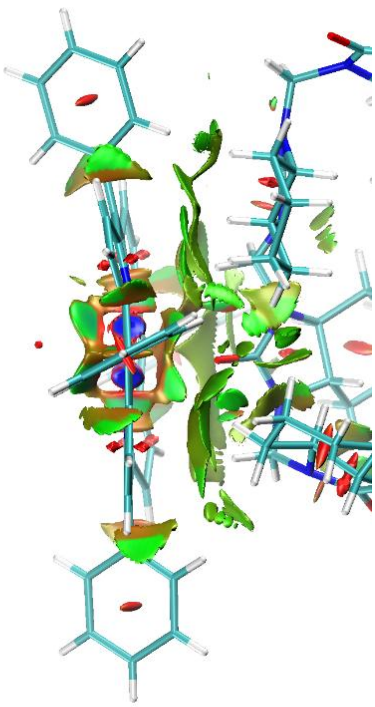

C

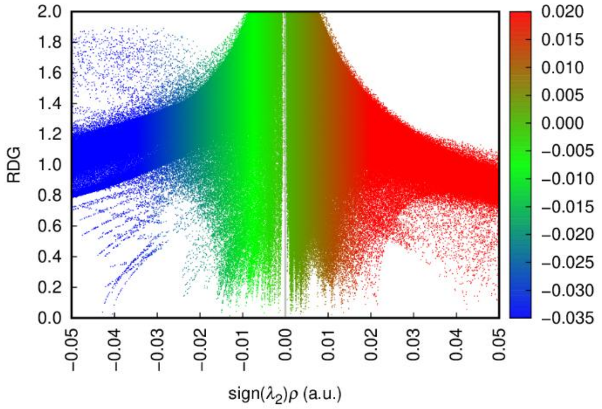

Figure S145 RDG isosurface maps of (*S*,*S*)-cycHC[8]·**7**, where grid data is selected between (*S*,*S*)-cycHC[8] and porphyrin **7**. A) diagonal view, B) same map viewed from the side, C) scatter graph

A

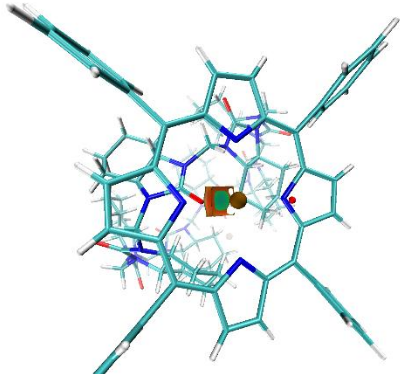

B

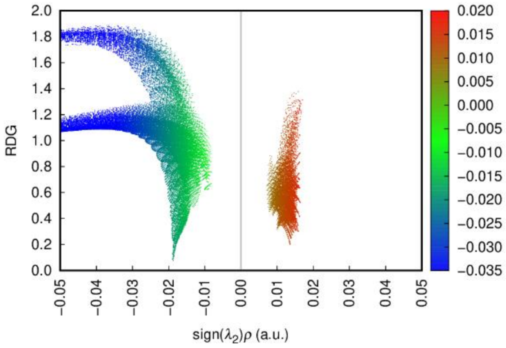

Figure S146 A) RDG isosurface map of (*S*,*S*)-cycHC[8]·**7**, where grid data is selected between the carbonyl oxygen of (*S*,*S*)-cycHC[8] and hexa-coordinated magnesium between porphyrin **7**. B) corresponding scatter graph

6.3 Interaction Region Indicator analysis

The IRI grid data was selected to include the porphyrin and the adjacent cycHC atoms. The coloring scheme follows the same convention as in the NCI representation (**Figure S118**). In addition, unlike NCI, the IRI visualization also highlights stronger interactions such as covalent bonds in blue.

Table S8 Table comparing IRI isosrufaces and scatter graphs of (*S*,*S*)-cycHC[8]·**2(a)** and (*S*,*S*)-cycHC[8]·**7**. First row are complex names, second row are full views of interaction surfaces, third row is cole-up of metal-oxygen interactio and final row isscatter graph

(*S*,*S*)-cycHC[8]·**2**(a)	(*S*,*S*)-cycHC[8]·**7**
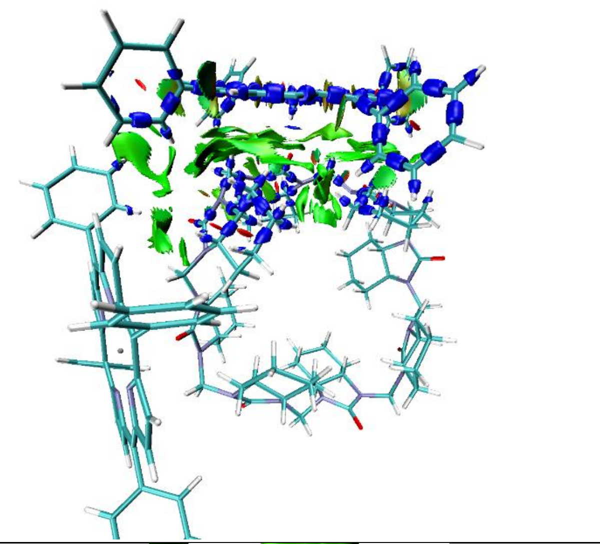	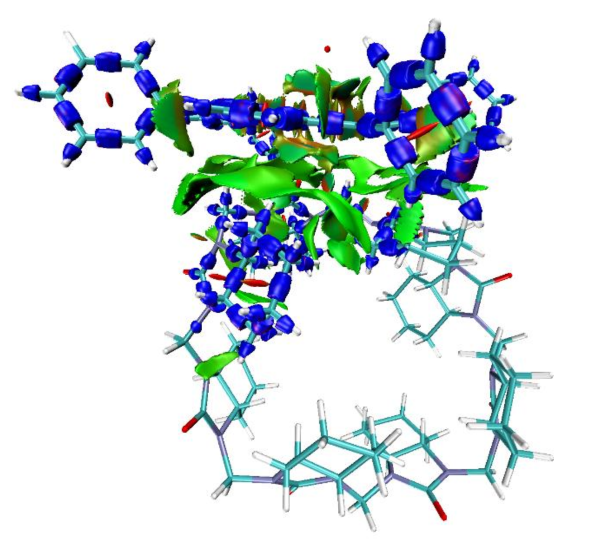
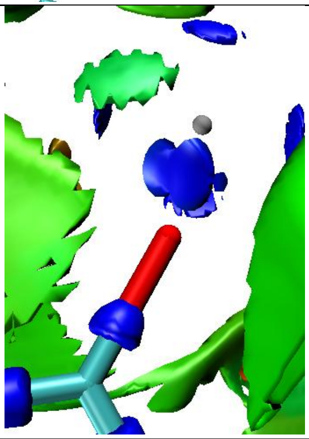	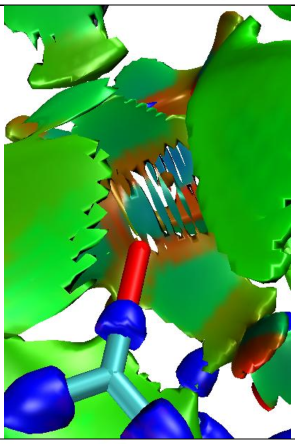
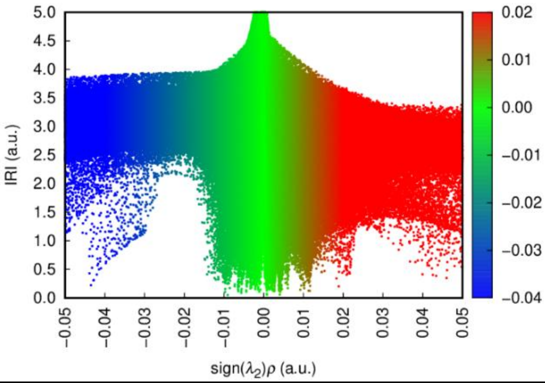	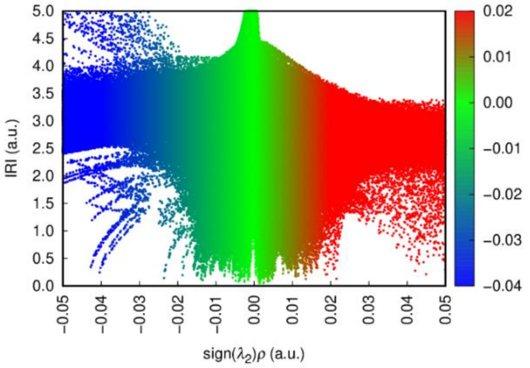

7. References

(1) Thordarson, P. Determining Association Constants from Titration Experiments in Supramolecular Chemistry. *Chem. Soc. Rev.* **2011**, *40* (3), 1305–1323.

(2) Brynn Hibbert, D.; Thordarson, P. The Death of the Job Plot, Transparency, Open Science and Online Tools, Uncertainty Estimation Methods and Other Developments in Supramolecular Chemistry Data Analysis. *Chem. Commun.* **2016**, *52* (87), 12792–12805.

(3) Ustrnul, L.; Kaabel, S.; Burankova, T.; Martõnova, J.; Adamson, J.; Konrad, N.; Burk, P.; Borovkov, V.; Aav, R. Supramolecular Chirogenesis in Zinc Porphyrins by Enantiopure Hemicucurbit[*n*]Urils (*n* = 6, 8). *Chem. Commun.* **2019**, *55* (96), 14434–14437.

(4) Shalima, T.; Mishra, K. A.; Kaabel, S.; Ustrnul, L.; Bartkova, S.; Tõnsuaadu, K.; Heinmaa, I.; Aav, R. Cyclohexanohemicucurbit[8]Uril Inclusion Complexes With Heterocycles and Selective Extraction of Sulfur Compounds From Water. *Front. Chem.* **2021**, *9*, Article 786746.

(5) Kaabel, S.; Adamson, J.; Topić, F.; Kiesilä, A.; Kalenius, E.; Öeren, M.; Reimund, M.; Prigorchenko, E.; Lõokene, A.; Reich, H. J.; Rissanen, K.; Aav, R. Chiral Hemicucurbit[8]Uril as an Anion Receptor: Selectivity to Size, Shape and Charge Distribution. *Chem. Sci.* **2017**, *8* (3), 2184–2190.

(6) CrysAlisPro Software System, 2017.

(7) Hooft, R. W. W. COLLECT, 1998.

(8) Otwinowski, Z.; Minor, W. Processing of X-Ray Diffraction Data Collected in Oscillation Mode. In *Methods in Enzymology*; Macromolecular Crystallography Part A; Academic Press, 1997; Vol. 276, pp 307–326.

(9) G.M. Sheldrick. SADABS, 1996.

(10) Sheldrick, G. M. A Short History of *SHELX*. *Acta Crystallogr A Found Crystallogr* **2008**, *64* (1), 112–122.

(11) G.M. Sheldrick. SHELXL13. Program Package for Crystal Structure Determination from Single Crystal Diffraction Data, 2013.

(12) Sheldrick, G. M. Crystal Structure Refinement with *SHELXL*. *Acta Crystallogr C Struct Chem* **2015**, *71* (1), 3–8.

(13) Hirshfeld, F. L. Can X-Ray Data Distinguish Bonding Effects from Vibrational Smearing? *Acta Cryst A* **1976**, *32* (2), 239–244.

(14) Thorn, A.; Dittrich, B.; Sheldrick, G. M. Enhanced Rigid-Bond Restraints. *Acta Crystallogr A Found Crystallogr* **2012**, *68* (4), 448–451.

(15) P. van der Sluis, A.L. Spek. BYPASS: An Effective Method for the Refinement of Crystal Structures Containing Disordered Solvent Regions. *Acta Cryst.* **1990**, No. A46, 194–201.

(16) Spek, A. L. Structure Validation in Chemical Crystallography. *Acta Crystallogr D Biol Crystallogr* **2009**, *65* (2), 148–155.

(17) A.C. Larson. In *Crystallographic Computing*; Copenhagen: Munksgaard, 1970; pp 291–294.

(18) Neese, F. Software Update: The ORCA Program System—Version 5.0.

(19) Neese, F. An Improvement of the Resolution of the Identity Approximation for the Formation of the Coulomb Matrix.

(20) Garcia-Ratés, M.; Neese, F. Effect of the Solute Cavity on the Solvation Energy and Its Derivatives within the Framework of the Gaussian Charge Scheme.

(21) Neese, F. The SHARK Integral Generation and Digestion System.

(22) Lu, T. A Comprehensive Electron Wavefunction Analysis Toolbox for Chemists, Multiwfn. *J. Chem. Phys.* **2024**, *161* (8), 082503.

(23) Lu, T.; Chen, F. Multiwfn: A Multifunctional Wavefunction Analyzer. *J Comput Chem* **2012**, *33* (5), 580–592.

(24) Zhang, J.; Lu, T. Efficient Evaluation of Electrostatic Potential with Computerized Optimized Code. *Phys. Chem. Chem. Phys.* **2021**, *23* (36), 20323–20328.

(25) Johnson, E. R.; Keinan, S.; Mori-Sánchez, P.; Contreras-García, J.; Cohen, A. J.; Yang, W. Revealing Noncovalent Interactions. *J. Am. Chem. Soc.* **2010**, *132* (18), 6498–6506.
(26) Lu, T.; Chen, Q. Interaction Region Indicator: A Simple Real Space Function Clearly Revealing Both Chemical Bonds and Weak Interactions**. *Chem.-Methods* **2021**, *1* (5), 231–239.
(27) Humphrey, W.; Dalke, A.; Schulten, K. VMD: Visual Molecular Dynamics. *Journal of Molecular Graphics* **1996**, *14* (1), 33–38.
